# A genomic perspective of the aging human and mouse lung with a focus on immune response and cellular senescence

**DOI:** 10.1186/s12979-023-00373-5

**Published:** 2023-11-06

**Authors:** Meng He, Jürgen Borlak

**Affiliations:** Centre for Pharmacology and Toxicology, Hannover Medical School, Carl-Neuberg-Str. 1, 30625 Hannover, Germany

**Keywords:** Comparative genomics, Aging mouse and human lung, ECM remodeling, Inflammaging, Immunosenescence, Pulmonary surfactant, Single cell RNA sequencing

## Abstract

**Background:**

The aging lung is a complex process and influenced by various stressors, especially airborne pathogens and xenobiotics. Additionally, a lifetime exposure to antigens results in structural and functional changes of the lung; yet an understanding of the cell type specific responses remains elusive. To gain insight into age-related changes in lung function and inflammaging, we evaluated 89 mouse and 414 individual human lung genomic data sets with a focus on genes mechanistically linked to extracellular matrix (ECM), cellular senescence, immune response and pulmonary surfactant, and we interrogated single cell RNAseq data to fingerprint cell type specific changes.

**Results:**

We identified 117 and 68 mouse and human genes linked to ECM remodeling which accounted for 46% and 27%, respectively of all ECM coding genes. Furthermore, we identified 73 and 31 mouse and human genes linked to cellular senescence, and the majority code for the senescence associated secretory phenotype. These cytokines, chemokines and growth factors are primarily secreted by macrophages and fibroblasts. Single-cell RNAseq data confirmed age-related induced expression of marker genes of macrophages, neutrophil, eosinophil, dendritic, NK-, CD4^+^, CD8^+^-T and B cells in the lung of aged mice. This included the highly significant regulation of 20 genes coding for the CD3-T-cell receptor complex. Conversely, for the human lung we primarily observed macrophage and CD4^+^ and CD8^+^ marker genes as changed with age. Additionally, we noted an age-related induced expression of marker genes for mouse basal, ciliated, club and goblet cells, while for the human lung, fibroblasts and myofibroblasts marker genes increased with age. Therefore, we infer a change in cellular activity of these cell types with age. Furthermore, we identified predominantly repressed expression of surfactant coding genes, especially the surfactant transporter Abca3, thus highlighting remodeling of surfactant lipids with implications for the production of inflammatory lipids and immune response.

**Conclusion:**

We report the genomic landscape of the aging lung and provide a rationale for its growing stiffness and age-related inflammation. By comparing the mouse and human pulmonary genome, we identified important differences between the two species and highlight the complex interplay of inflammaging, senescence and the link to ECM remodeling in healthy but aged individuals.

**Graphical Abstract:**

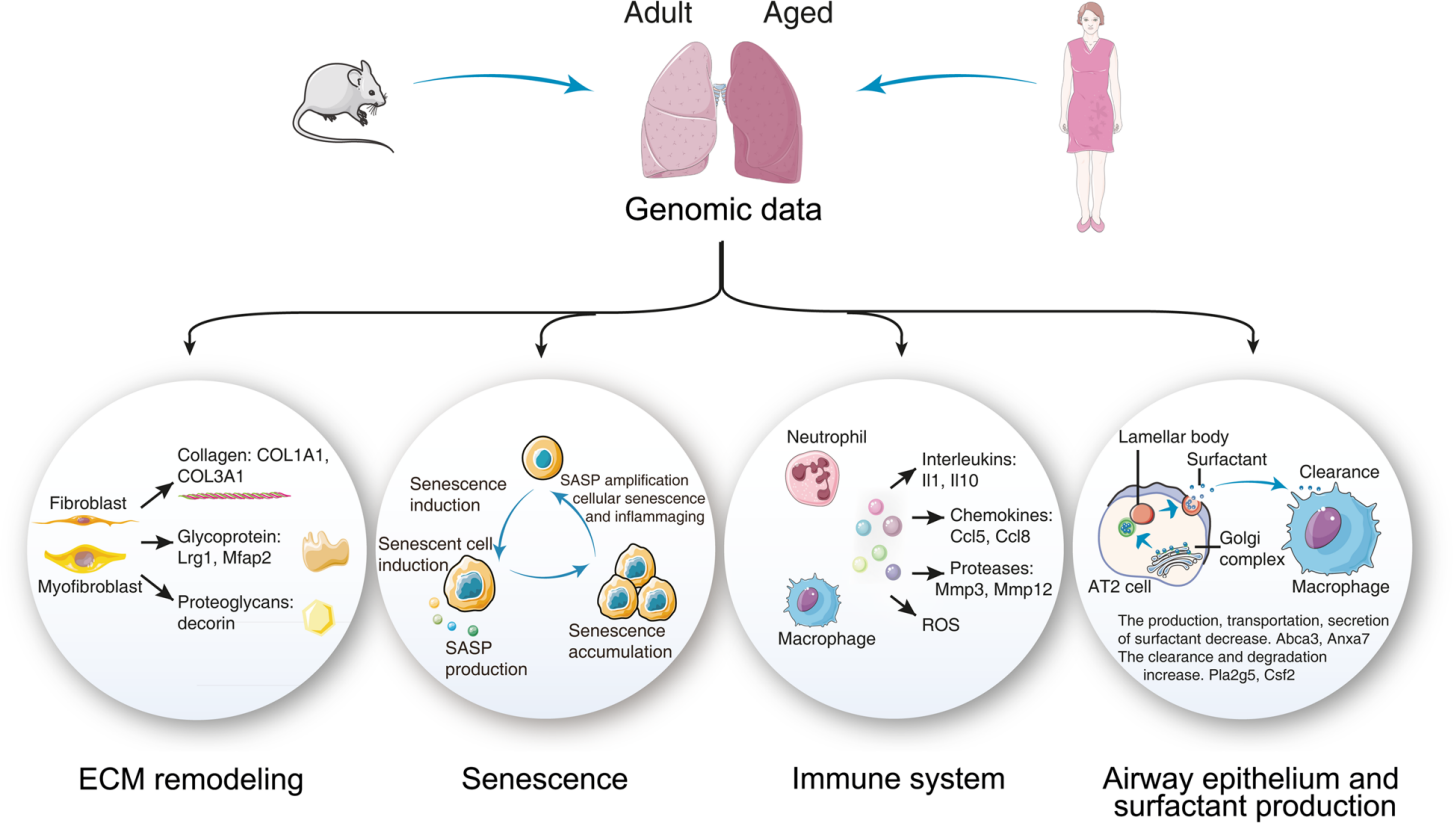

**Supplementary Information:**

The online version contains supplementary material available at 10.1186/s12979-023-00373-5.

## Background

**Fig. 1:**
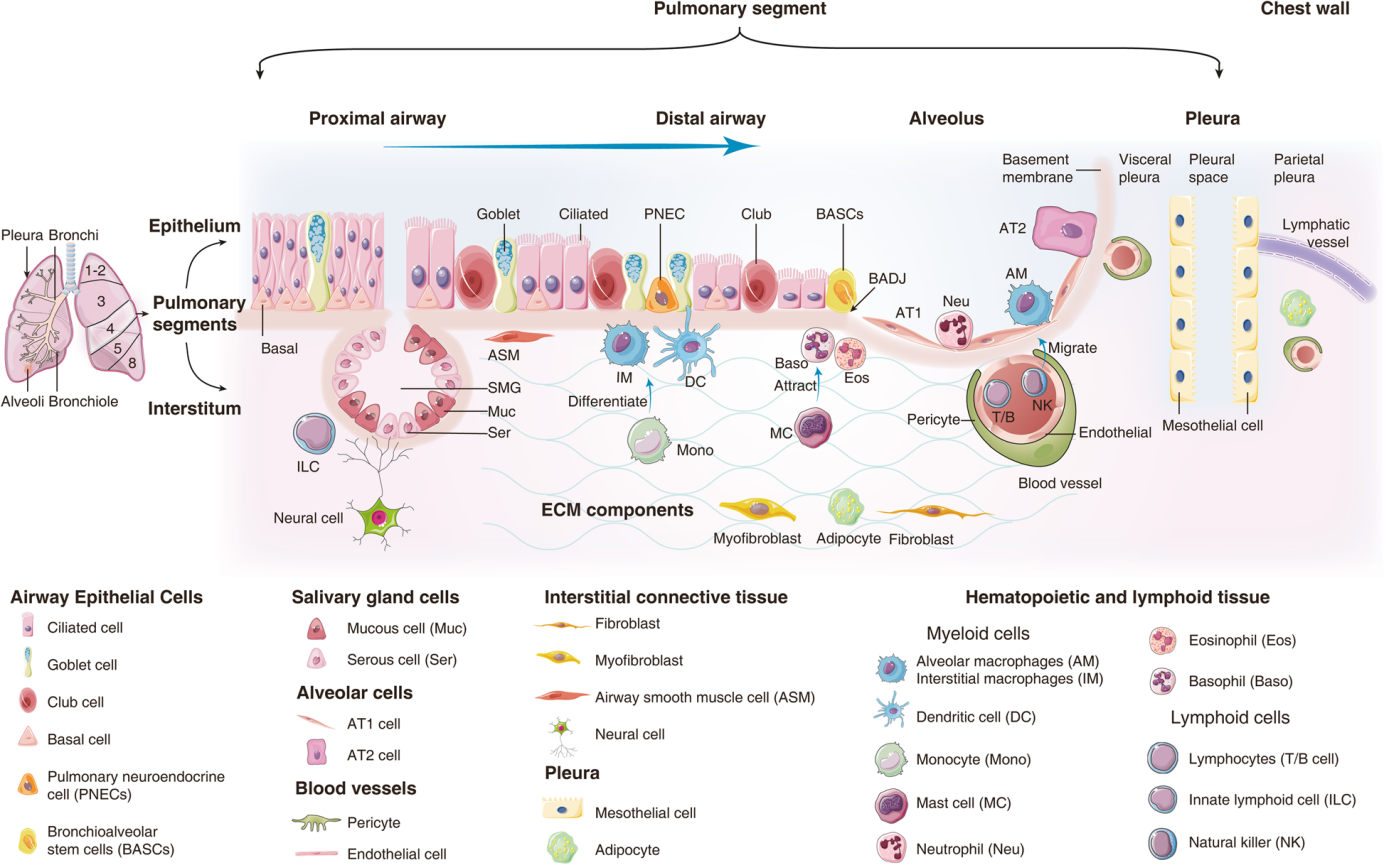
The landscape of pulmonary resident cells. According to their morphology and function, resident cells of the respiratory tract are divided into airway epithelium, alveoli and salivary gland of the bronchi, interstitial connective tissue, blood vessel and cells of hematopoietic and lymphoid origin, the pleura as well as poorly defined cells including progenitor cells

The importance of gene-environment interactions in lung disease is well recognized [1], and given that the lung is a major port of entry for xenobiotics and pathogens into the systemic circulation, its barrier function at the alveolar septa is of critical importance. As with other organs, the lung ages with time, and this process is associated with structural and functional changes [2] to ultimately limit organ functions. The changes are governed by the complex interplay of diverse cellular components, and in the seminal review of Franks et al. [3], the approximately 40 different cells of the lung have been classified by their morphology and function. Correspondingly, resident cells of the respiratory tract are divided into airway epithelium, alveoli, salivary gland of the bronchi, interstitial connective tissue, blood vessel and cells of hematopoietic and lymphoid origin, the pleura as well as poorly defined cells including progenitor cells (Fig. 1).

In terms of quantity, alveolar cells are by far the largest group of cells, and are classified as type I (AT1) and type II pneumocytes (AT2) which differ in their functions. Estimates suggest 480 million epithelial cells in the alveoli of adult human lung [4] which strikingly amounts to a surface area of about 70 m^2^ [5]. AT1 cells cover > 95% of the alveolar surface area and serve as barrier in gas exchange while AT2 cells produce surfactant and therefore maintain the surface tension of the alveoli [6]. Lamellar bodies hallmark AT2 cells and typically are seen as single cells among layers of AT1 cells. In addition, AT2 cells can differentiate into AT1 ones, especially during injury and regeneration and are classified as progenitor cells [7].

The airway epithelium is composed of various epithelial cells with distinct morphological features [8], and the immune responses by airway epithelia have been the subject of a review [9]. Usually the cells localize along the respiratory tree, that is from the nasal cavity to the bronchi in form of pseudostratified columnar ciliated epithelium as well as simple columnar and cuboidal epithelium of bronchioles [10]. The basal cells are specialized airway epithelium and retain the ability to differentiate into distinct cell types, such as ciliated and club cells and function in lung homeostasis, regeneration and tissue repair [11]. Intermingled are goblet cells which stem from basal cells and secrete mucus for moisture and to trap pathogens or particulate matter for destruction and removal [12].

A distinct group of cells of the respiratory tract functions in blood vessels [13]. Here, the intima is composed of a tiny layer of endothelial cells with complex functions in vascular biology and immune response [14]. Expectedly, the surface area of alveoli is covered by about 70% of blood capillaries.

Given that the lung serves as a port of entry for environmental pathogens and toxins, a wide range of innate and adaptive immune cells can be found and this includes alveolar macrophages, dendritic cells, circulating monocytes, T and B lymphocytes and granulocytes [15]. Among the immune cells, alveolar macrophages are the first line of defense in the clearance of particulate matter and pathogens [16], and they can be found in the pulmonary interstitium where they reside in the parenchyma between the microvascular endothelium as well as alveolar macrophages, which are in close contact with type I and type II pneumocytes. Furthermore, monocytes can differentiate into polarized macrophages, based on their response to different cytokines [17] and play decisive roles in the immune response [18].

Additionally, the interstitial connective tissue of the lung provides structural support and is composed of extracellular matrix (ECM) proteins which are cross-linked. Of note, interstitial lung disease (ILD) refers to the scaring of connective tissue and comprises a range of conditions including chronic obstructive pulmonary disease (COPD) and idiopathic pulmonary fibrosis (IPF) [19]. ECM components which are produced by fibroblasts such as type III collagen, elastin and proteoglycans become activated during wound repair. Their accumulation contributes to stiffness, i.e. a key feature of ILD [20].

Further, the pleura is layered by a simple squamous epithelium (mesothelium), and mesothelial cells play a vital role in lung development and immune response [21]. Finally, the poorly defined cells subsume multiple stem/progenitor populations in different regions of the adult lung [22].

Importantly, with the advent of single cell genomics, it is possible to study genomes of distinct cell types of the lung, for example, surfactant producing AT2 cells [23] or mucin producing bronchial epithelial cells [24]. Correspondingly, single cell transcriptomes enabled the tracing of specific transcriptomes of a particular cell type to environmental changes and exposures to toxin and pathogens [25].

So far only a few studies investigated the complex age-related changes of the pulmonary genome [5, 26–30], and there is unmet need to better comprehend the molecular events associated with the aging of the lung and its immune responses. Based on genomic data, we aimed at identifying genomic responses linked to the aging lung by considering whole lung and single cell transcriptomic data. We were particularly interested in an understanding of the age-related changes associated with stiffness and immune responses and investigated cellular senescence and surfactant biology. Finally, we compared genomic responses of the aging mouse and human lung and considered genomic variations among resident cells of the respiratory tract.

## Results

**Fig. 2:**
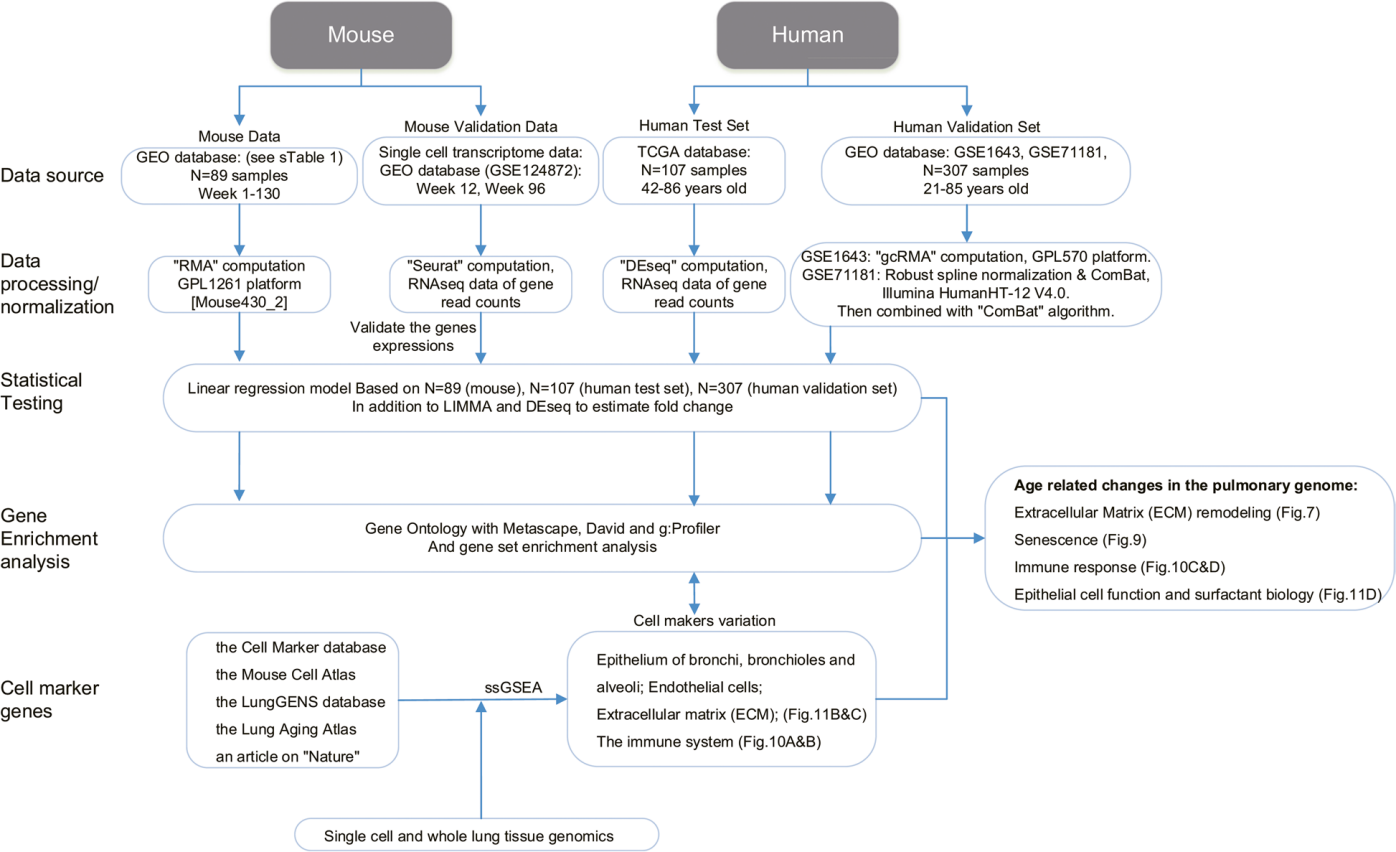
CONSORT flow diagram of genomic data analysis for the mouse and human pulmonary genomes. We structured the process into data retrieval, normalization, linear regression and other statistical testing to obtain significant DEGs (DeSeq, LIMMA), gene ontology and gene enrichment analysis and validation of results by single cell RNAseq

Figure 2 depicts the workflow of the data analysis. The process is divided into data retrieval, normalization, statistical testing for DEGs, gene enrichment analysis and single cell RNAseq. Meanwhile, we divided the mouse and human lung genomic data into a test and validation set and performed independent computational analysis.

### Mouse genomic data sets

We considered 89 individual mouse genomic data sets for in-depth evaluation (GEO database GSE83594, GSE66721, GSE55162, GSE34378, GSE38754, GSE23016, GSE25640, GSE18341, GSE15999, GSE14525, GSE11662, GSE10246, GSE9954, GSE6591, GSE3100) and show the distribution of genomic data according to sex and age in supplementary Figure S1 and supplementary Table S1.

Based on *N* = 43 individual male and *N* = 46 female mice genomic data sets (supplementary Table S1), we searched for time dependent gene expression changes. The linear regression model fitted 105 significantly regulated genes. Note, we only considered DEGs which fulfilled the criteria: FDR adjusted p-value of 0.05 and a goodness of fit of R2 > 0.4. We also considered sex differences, and this revealed 690 (305 up, 385 down) and 170 (144 up, 26 down) genes specifically regulated among male and female mice. Note, 68 genes are common to both sex. We performed gene enrichment analysis, and for males enriched terms are hemostasis, positive regulation of cell adhesion, positive regulation of cell migration, regulation of MAPK cascades, platelet activation, ECM organization, chemotaxis, blood vessel development, adaptive immune system. Likewise, for females enriched terms are inflammatory response, leukocyte chemotaxis, cell activation, nerutrophil degranulation, regulation of ERK1 and ERK2 cascade. Although we observed sex dependent gene expression changes with age, the gene ontology enriched terms are similar and alert to inflammation, immune response, ECM remodeling, cell adhesion among others. The Metascape enriched terms specifically for up- and down-regulated genes are shown supplementary Figure S2.

We also evaluated time dependent gene expression changes by excluding animals aged < 5 weeks. Independent of sex, the regression model fitted 90 DEGs whose expression changed with time. Moreover, we compared DEGs resulting from the two regression models, i.e. with and without animals aged 1–5 weeks and found 76% to be in common. Therefore, from the very beginning of life, 80 pulmonary genes (68 up, 12 repressed genes) are continuously regulated with age, and we show examples of significantly regulated genes (*p* < 0.05, R2 > 0.4) in supplementary Figure S3.

Additionally, we analysed the genomic data with the LIMMA package (= Linear Models for Microarray Data) and compared animals aged 1–5 weeks (*N* = 15), with animals aged 6–26 (*N* = 52) and 52–130 (*N* = 22) weeks. This revealed 120 and 134 genes, respectively as continuously up- and down-regulated with age. Furthermore, 38 genes are common when the results from the linear regression model and LIMMA were compared.

### Human lung genomic test set data

We retrieved RNA-Seq data of 107 histologically proven normal lung tissue samples from the TCGA repository, i.e. resection material from lung cancer patients (supplementary Table S2). The cohort consisted of individuals aged 42–86 years and based on normalized counts, the linear regression model fitted 237 significantly regulated genes (27 up and 210 down). We also analysed the RNAseq data with the DESeq2 package and compared individuals aged 42–50 (*N* = 5) to 71–86 (*N *= 40) years. This defined 1430 genes (716 up, 714 down) with a FC ≥ 1,5 and an FDR adjusted *p*-value < 0.05. Note, 56% of genes coming from the linear regression model overlap with significantly regulated genes as defined by the DESeq2 method (supplementary Figure S4).

### Human lung genomic validation data set

We retrieved genomic data sets of histologically normal lung tissue from 23 human sudden death donors (GSE1643) in addition to 284 individuals who underwent lobectomy for lung adenocarcinoma (GSE71181). We only considered genomic data of histologically proven normal lung parenchyma. Together, the validation set consisted of 307 individual genomic data sets (supplementary Table S2). Based on linear regression analysis the model fitted 857 up- and 816 down-regulated genes.

Moreover, we used LIMMA to identify significantly regulated genes and compared two age groups, i.e. 21–50 years (*N* = 27) and 71–85 years (*N* = 89). We applied the criteria FC ≥ 1,5 and an FDR adjusted *p*-value < 0.05 and this defined 20 and 4 genes, respectively as significantly up- and down-regulated. Furthermore, all genes identified by LIMMA are significant in the linear regression model.

Lastly, we compared the human test and validation set, and based on the regression model findings 26 genes are commonly regulated.

### Gene ontology and gene set enrichment analysis (GSEA)

**Fig. 3:**
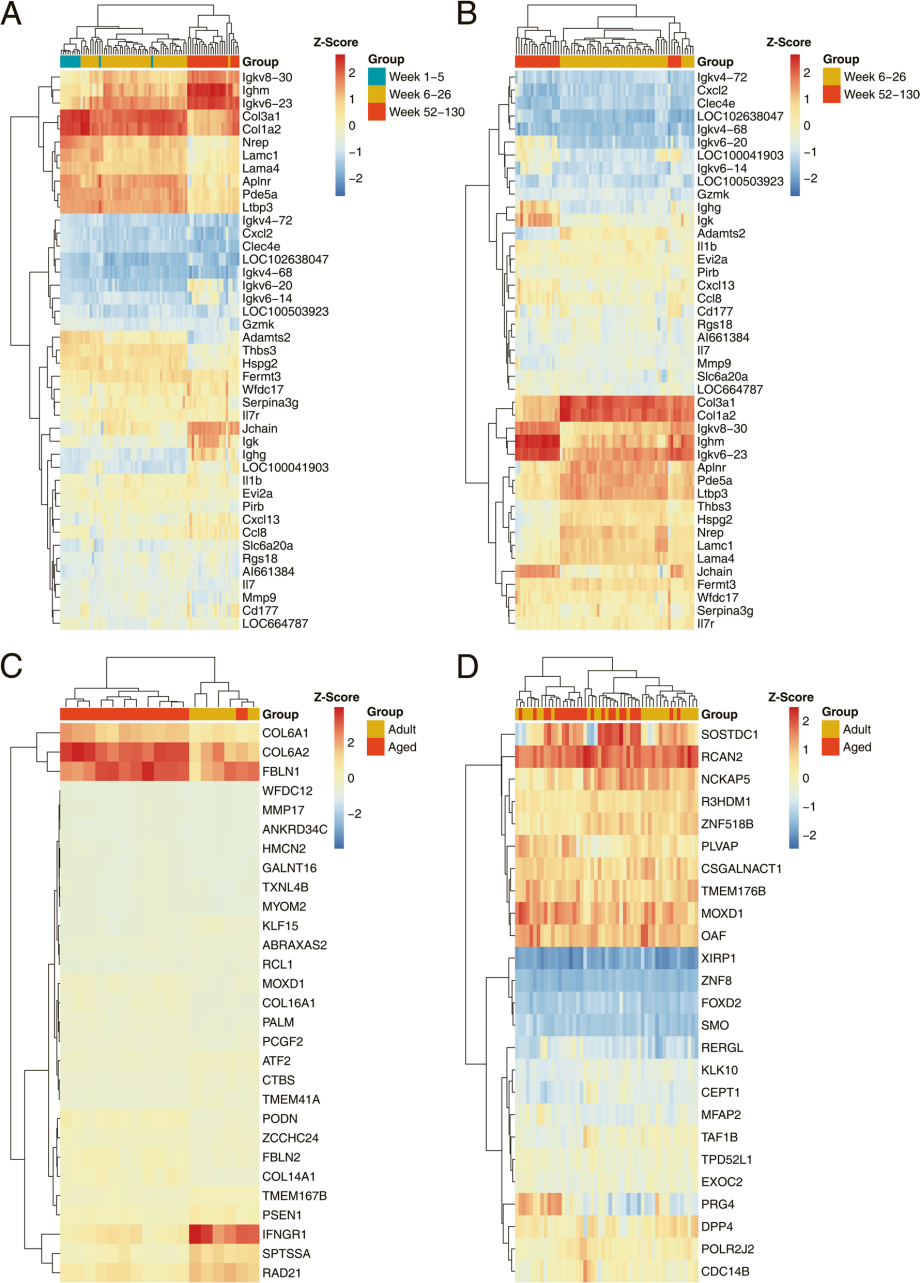
Heatmaps of age-related gene expression changes in the mouse and human lung. **A** Examples of significantly regulated genes from the linear regression model. The data are based on 89 individual mouse samples and the dendrogram separates the different age groups. **B** Examples of significantly regulated genes from the linear regression model. The data are based on 74 mice aged 6–26 and 52–130 weeks, and the dendrogram separates the different age groups. **C** Examples for significantly regulated genes from the linear regression model for the human test set. Note, we selected 17 individuals based on the maximum difference in age, i.e. < 50 years and > 80 years. **D** Examples for significantly regulated genes from the linear regression model for the human validation set. Note, we selected 51 individuals based on the maximum difference in age, i.e. < 50 years and > 80 years

We used two different approaches to group DEGs based on gene cluster analysis and gene ontology terms. Depicted in Fig. 3A and B are the Z-scores for all 89 data sets and for 74 mice aged 6–26 and 52–130 weeks. We show significantly regulated genes obtained by the linear regression model and discuss their relevance for aging below. Similar, Fig. 3C and 3D are heatmaps for a subset of human test (*N* = 17) and validation sets (*N* = 51). With mice and the human test set, the data are mostly separated by age while for the human validation set some adult lung samples are intermingled with the aged ones.

**Fig. 4:**
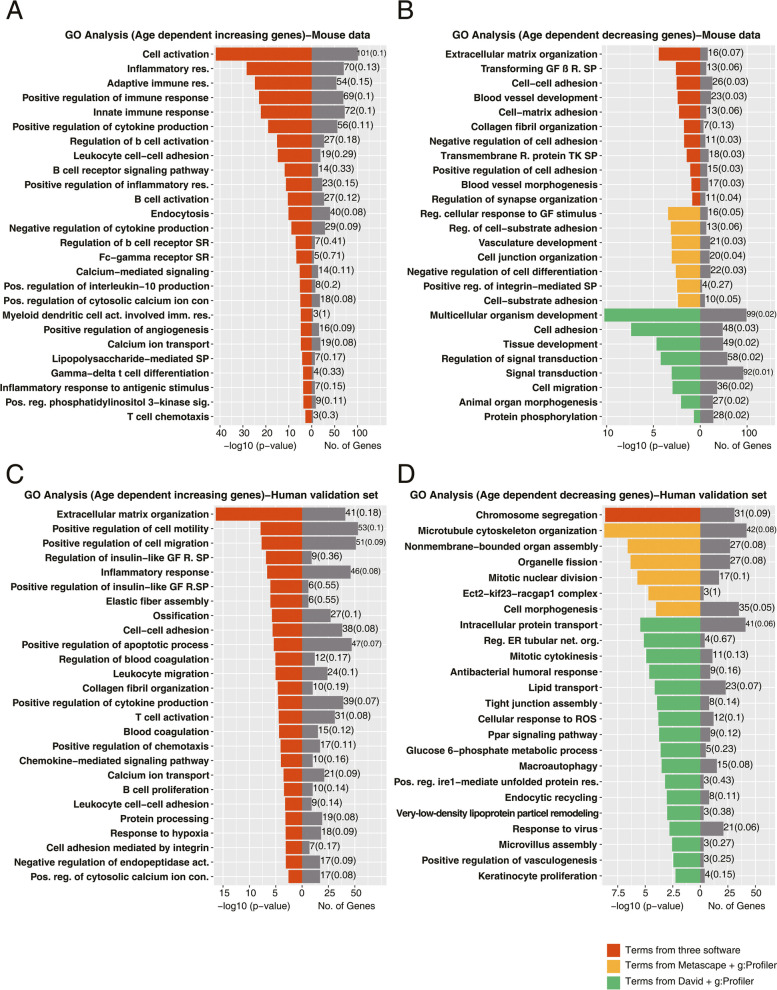
Enrichment analysis of age-related gene expression changes. Based on the linear regression model we identified significantly regulated genes in the aging lung of mice and humans. **A** Shown are significantly enriched GO terms for upregulated genes of *N* = 74 individual mice aged 6–130 weeks. **B** Shown are significantly enriched GO terms for down-regulated genes of *N* = 74 individual mice. **C** Shown are significantly enriched GO terms for upregulated genes of *N* = 307 individual human donors aged 21 – 85 years. **D** Shown are significantly enriched GO terms for down-regulated genes of *N* = 307 individual human donors aged 21 – 85 years. Abbreviations: SP (signaling pathway), GF (growth factor), R.(receptor), res. (response), act.(activation), con. (concentration), pos. (positive), reg. (regulation), sig. (signaling), imm. (immune), org. (organization), net. (network)

Depicted in Fig. 4A&4B are enriched GO terms for mice aged 6–130 weeks. We separated up- and down-regulated genes and analyzed the data with Metascape, David and g:Profiler. We only considered enriched terms with a FDR adjusted *p*-value < 0.05. Based on 579 age increased gene expression changes as determined by the regression model (*p* < 0.05, R2 > 0.2), we identified 26 overlapped terms between the Metascape, David and g:Profiler (Fig. 4A). 579 upregulated genes mapped to the terms cell activation, inflammatory response, regulation of cytokine production, endocytosis, calcium-mediated signaling, positive regulation of angiogenesis, lipopolysaccharide-mediated signaling pathway (Fig. 4A). Similar, we mapped 223 repressed genes (Fig. 4B) to GO terms, and although less than 10% overlapped (supplementary Figure S5) most terms were similar. Among the common GO terms, we wish to highlight ECM organization, cell adhesion, blood vessel development, collagen fibril organization, transmembrane receptor protein tyrosine kinase signaling pathway and transforming growth factor beta receptor signaling pathway. Moreover, we considered GO terms overlapped by two different annotation tools, and given the focus of our study, i.e. genes regulated in the aging lung, we selected top GO terms linked to lung biology (Fig. 4B). About 102 and 140 down-regulated genes, respectively overlapped between Metascape and g:Profiler and David and g:Profiler and significantly enriched terms are regulation of cellular response to growth factor stimulus, regulation of cell-substrate adhesion, vasculature development, positive regulation of integrin/mediated signaling pathway, cell-substrate adhesion, cell junction organization, regulation of signal transduction and cell migration.

Additionally, we performed gene enrichment analysis for the human validation set of *N* = 307 individuals. The linear regression model fitted 857 up and 816 down-regulated genes. Significantly enriched GO terms for age-dependent increased gene expression changes include extracellular matrix organization, positive regulation of cell motility and cell migration, leukocyte migration, inflammatory response, elastic fiber assembly, collagen fibril organization and positive regulation of apoptotic response (Fig. 4C). Likewise, for repressed genes and based on DAVID annotations, enriched terms are tight junction assembly, cellular response to reactive oxygen species, PPAR signaling pathway and lipid transport (Fig. 4D).

#### Mouse gene set enrichment analysis

Apart from GO terms, we performed gene set enrichment analysis (GSEA) for 89 mouse lung genomic data sets. The basic algorithm is described in [31] and based on the Kolmogorov–Smirnov test, which assesses the distribution of gene markers by comparing their position and distribution (rank order) in individual lung samples from young and aged animals. We report the normalized enrichment score (NES), p-value and adjusted *p*-value in supplementary Table S3, and according to NES, cell cycle, cell adhesion, collagen-fibril organization, extracellular matrix structural constituent and morphogenesis of epithelium are enriched in animals aged 1–5 (*N* = 15) as compared to 6–26 (*N* = 52) weeks. We likewise compared animals aged 6–26 (*N* = 52) vs 52–130 (*N* = 22) weeks, and the GSEA defined blood vessel development, basal lamina, integrin binding, cellular response to vascular endothelial growth factor stimulus, positive regulation of autophagy as significantly enriched. Conversely, fatty acid transport, platelet activation, scavenger receptor activity, extracellular space, B-cell activation and antigen processing and antigen processing and presentation of exogenous peptide antigen via MHC class II were enriched terms for aged animals.

### Time resolved gene expression changes in the aged lung of mice

**Fig. 5:**
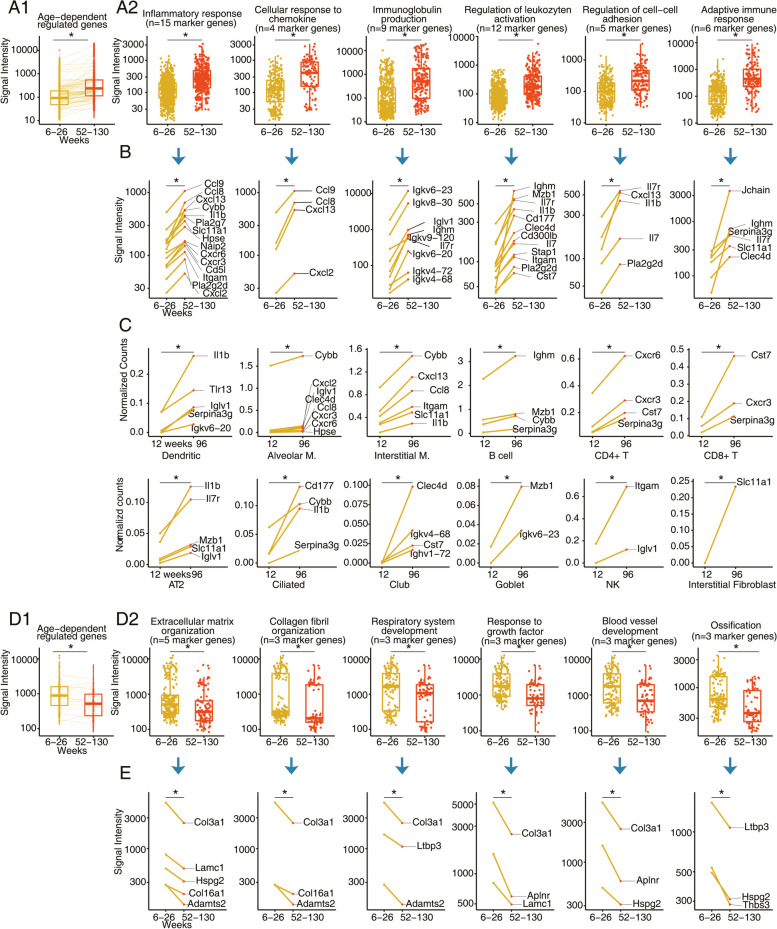
Continuous gene expression changes of the aging mouse lung. Based on the linear regression model for *N* = 74 individual mouse genomic data sets (mice aged 6–130 weeks), we identified 77 and 13 genes, respectively whose expression increased or declined with age (*p* < 0.05, R2 > 0.4). We independently confirmed their regulation by evaluating single cell RNAseq data of alveolar and interstitial macrophages, interstitial fibroblast, dendritic cells, CD4^+^ and CD8^+^ T-cells, B-cells as well as AT2, ciliated, club, goblet and NK cells. The full genes names are given in supplementary Table S4. Panel A1: Boxplot of 77 DEGs with increased expression over time. Panel A2: Boxplots of 37 uniquely upregulated genes coding for immune response. **B** Shown are individual immune gene regulation with age. The genes refer to the data shown in the boxplots of A2 for the various ontology terms. **C** Findings from RNAseq to independently validate the regulation of immune genes in resident cells of the lung. Panel D1: Boxplots of down-regulated genes. Panel D2: Boxplot for significantly down-regulated DEGs based on enriched gene ontology terms. **E** Regulation of 13 uniquely down-regulated genes with age. The genes refer to the data shown in the boxplots of D2 for the various ontology terms. Statistical testing is based on the “Kruskal–Wallis” test. *p < 0.05

We searched for continuous gene expression changes and based on a linear regression model for 74 individual pulmonary genomic data sets (ages 6 – 130 weeks) we identified 77 up- and 13 down-regulated genes whose expression changed with age (Fig. 5A1 and 5D1 and supplementary Table S4). The results for all 89 individual mouse genomic data sets (ages 1–130 weeks) are summarized in supplementary Figure S6. The boxplots shown in Fig. 5A2 depict significantly increased gene expression changes, and the genes are grouped based on their functions. For instance, we observed 6 genes coding for adaptive immune response among 74 individual animals whose expression continuously increased with time (Fig. 5A2). Therefore, the boxplot comprises a total of 444 individual gene expression changes (= 74 animals × 6 genes). Strikingly, 49% of the continuously upregulated genes code for immune response, and apart from adaptive immune and inflammatory responses, we observed an age-related increased expression of genes coding for cellular response to chemokine and immunoglobulin production. Furthermore, the ontology terms highlighted leukocyte cell activation and regulation of cell adhesion, and shown in Fig. 5B are individual gene expression changes. To independently confirm the results, we interrogated single cell RNAseq data, and the results are summarized in Fig. 5C. Here we compared 12 week old animals with aged mice (= 96 weeks) and assessed the expression pattern of 37 immune response genes (Fig. 5C) in alveolar and interstitial macrophages, interstitial fibroblast, dendritic cells, CD4^+^ and CD8^+^ T-cells, B-cells as well as AT2, ciliated, club, goblet and NK cells. We confirmed an age-related induced expression of 27 genes and therefore validated 73% of immune response genes in RNAseq data of single cells isolated from lung tissue of mice. Note, the single cell RNAseq data comprises an average of 500–1000 cells (see GSE124872). The genomic data signify upregulation of gene markers linked to alveolar and interstitial macrophages, B-, CD4^+^ and CD8^+^ T cells. Indeed, a recent review highlighted the importance of age-related changes in the pulmonary adaptive and innate immune system [32].

Based on the David tool, 83% of the significantly upregulated genes can be grouped according to gene ontology terms. An additional 4% of upregulated genes were assigned to ontology terms defined by Metascape and the g:profiler, and the associated DEGs code for protein binding, immune response and extracellular space. For the remaining genes the software tools did not provide meaningful terms even though some important genes are worthy to mention. For instance, we observed significant regulation of alpha-N-acetyl-neuraminide alpha-2,8-sialyltransferase 6 (*St8sia6*), i.e. an enzyme that catalyzes the transfer of sialic acid to carbohydrates to influence cell–cell communication and adhesion. Its expression increased 1.8- and 3.3-fold in animals aged 6–26 and 52–130 weeks, respectively when compared to 1–5 week old animals. Another gene of interest is the membrane-spanning 4-domains, subfamily A, member 6B (Ms4a6b) which is expressed by regulatory T cells and functions as a co-stimulatory molecule to amplify antigen signals thereby modulating T-cell function [33, 34]. Indeed, this CD20 like precursor is induced (> 3-fold) when week 6–26 aged mice are compared to 1–5 week old animals. Its expression increased further when compared to aged animals. Another example refers to ecotropic viral integration site 2A, i.e. a leucine-zipper transmembrane protein [35] and protoncogene with multiple functions in tumor growth and inflammation [36, 37] as well as mitogenic MEK/ERK signaling in osteosarcoma [37]. Based on multi-omics network modeling Evi2a influenced ECM receptor interaction and focal adhesion [38], and we found *Evi2a* transcripts to continuously increase in expression. Additionally, the age-dependent regulation of the core component of nucleosome H4 clustered histone 17 (*Hist1h4m*) increased with age.

Apart from upregulated genes we searched for continuously down-regulated genes over time, and the results from the linear regression model are shown in Fig. 5D-E. Based on gene annotations the associated DEGs code for extracellular matrix and collagen fibril organization, respiratory system development, response to growth factors, blood vessel development and ossification (Fig. 5D). We classified 79% of continuously down-regulated DEGs with the David tool, and by querying the Metascape and the G:profiler software we assigned an additional 7% of DEGs to extracellular matrix structural components. Nonetheless, some DEGs were not allocated to specific ontology terms even though their regulation is of great significance. For instance, we found the gene coding for nicotinamide nucleotide transhydrogenase (Nnt), i.e. an enzyme that transfers protons to NAD^+^ and NADP^+^ repressed by 2-fold, respectively when animals aged week 1–5 were compared to aged mice. NADPH is a central cofactor for many biochemical processes and is required for the production of glutathione. Importantly, glutathione is one of the most important cellular defense lines in the detoxification of reactive oxygen species (ROS) [39] and also functions in inflammatory responses. Overexpression of Nnt in macrophages reduced intracellular ROS and the production of pro-inflammatory cytokines. Conversely, knockdown of *Nnt* increased intracellular ROS and inhibited cell proliferation [40]. Together, we consider the time dependent repression of *Nnt* as detrimental as it will dampen efficient ROS detoxification thereby contributing to cellular senescence.

A further example relates to Lys-Asp-Glu-Leu (KDEL) endoplasmic reticulum protein retention receptor 3 (Kdelr3), i.e. a seven-transmembrane-domain receptor localized within the Golgi complex. The binding of KDEL ligand to its receptor triggers phosphorylation of the Src-family of kinases, and the phosphorylation of p38 stimulates the activation of MAPKs [41]. Given that *Kdelr3* was 2-fold repressed in aged mice, we consider this change in gene expression as an adaptive response to age-related stress responses and inflammation.

Similar, we found 2-fold repressed hephaestin in aged mice. The gene codes for an exocytoplasmic ferroxidase and plays an essential role in cellular iron homeostasis. Hephaestin favours iron export from cells and the protein is expressed in epithelial cells of the alveoli, type II pneumocytes, the bronchiole and endothelial cells [42]. Due to its role in iron transport, we considered the repression of hephaestin to be detrimental as malfunction of the protein will lead to an iron-overload of pneumocytes and the propagation of reactive oxygen species (ROS). The importance of hephaestin in lung cancer iron homeostasis was the subject of a recent report, and reduced expression of hephaestin is associated with poor prognosis for lung adenocarcinoma and squamous cell carcinoma [43].

Regarding the regulation of genes coding for extracellular matrix a complex picture emerges with some being time dependently repressed, notably matrix metalloproteinase *(Mmp)-2*, *Mmp14*, the disintegrin and metalloproteinase with thrombospondin motifs *(Adamts)2*, while others (e.g., *Lrg1*, *Dcn*) were upregulated especially in animals aged 6–26 week when compared to old ones. The marker genes code for MMP and ADAMTS, and these function in ECM remodeling which we discuss in the following paragraph.

### Time resolved gene expression changes in the aged human lung

**Fig. 6:**
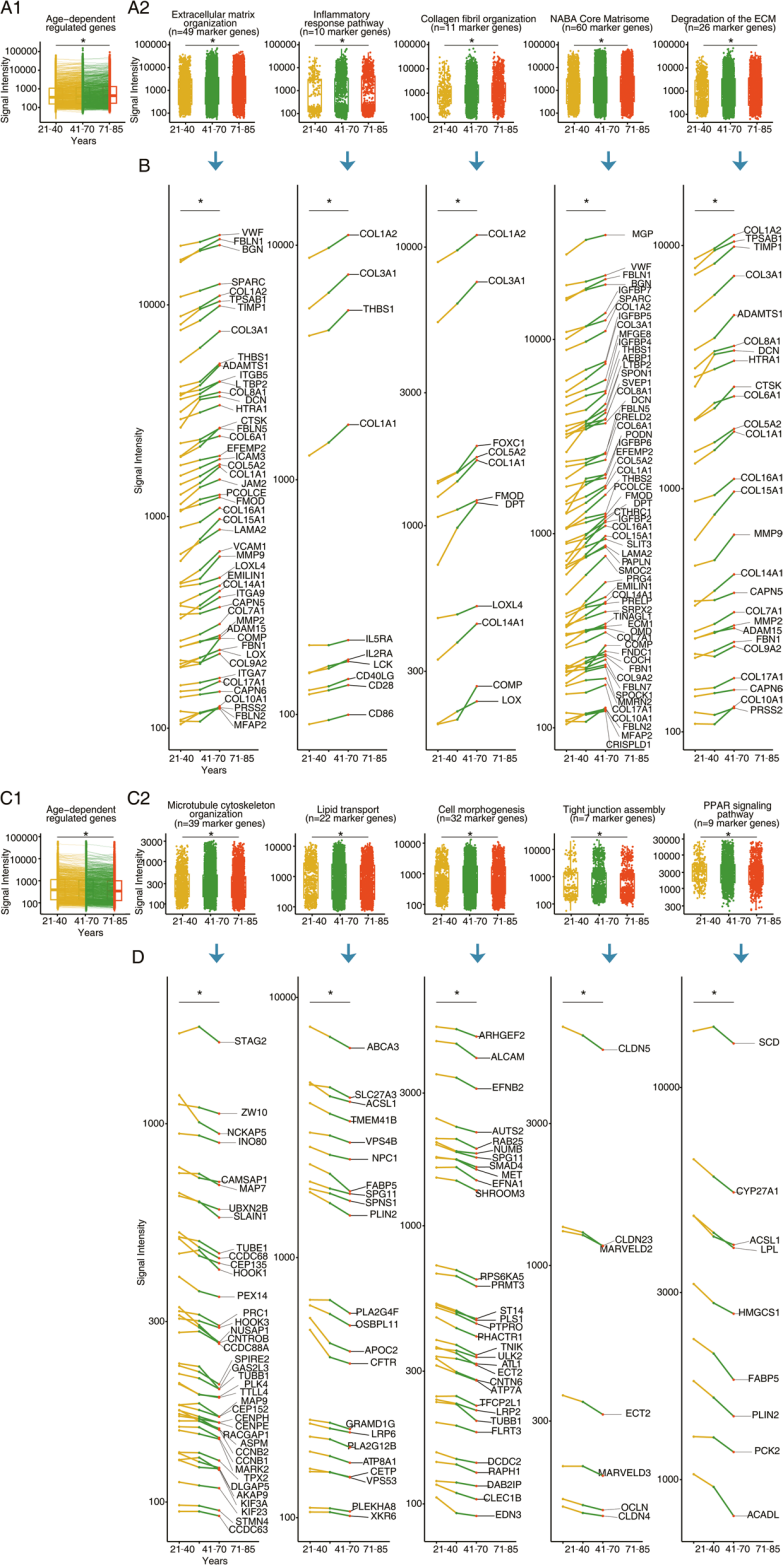
Gene expression changes of the aging human lung. Based on the linear regression model for *N* = 307 individual human genomic data sets (mice aged 21–85 years old), we identified 857 and 816 genes, respectively whose expression increased or declined with age (*p* < 0.05). Among the upregulated genes, 80 code for ECM remodeling, notably collagens (*N* = 13), proteoglycans (*N* = 7), matrix metalloproteinase (*N *= 4) and ECM-related glycoproteins (*N *= 33). We also include 10 genes coding for the inflammatory response pathway. Panel A1: Boxplot of 857 DEGs with increased expression over time. Panel A2: Boxplots of upregulated genes coding for ECM remodeling and immune response. **B** Regulation of individual genes with age. The genes refer to the data shown in the boxplots of A2 for the various ontology terms. Panel C1: Boxplot of 816 DEGs with decreased expression over time. Panel C2: Boxplots of down-regulated genes coding for cell morphogenesis, microtubule cytoskeleton organization, tight junction assembly, lipid transport and PPAR signaling. **D** Regulation of individual genes with age. The genes refer to the data shown in the boxplots of C2 for the various ontology terms

We searched for continuous gene expression changes in the pulmonary genome of the aging human lung and show in Fig. 6 the results for the validation set of 307 human lung samples. Given the primary aim of our study, we focused on the following GO terms for an age-related increase in gene expression changes: Naba core matrisome, extracellular matrix organization, degradation of the extracellular matrix, inflammatory response pathway and collagen fibril organization (Fig. 6A-B). The results for an age-related down-regulation of genes are shown in Fig. 6C-D. Here, we focused on the following GO terms: Cell morphogenesis, microtubule cytoskeleton organization, tight junction assembly, lipid transport and PPAR signaling.

### ECM remodeling in the aged lung of mice

**Fig. 7:**
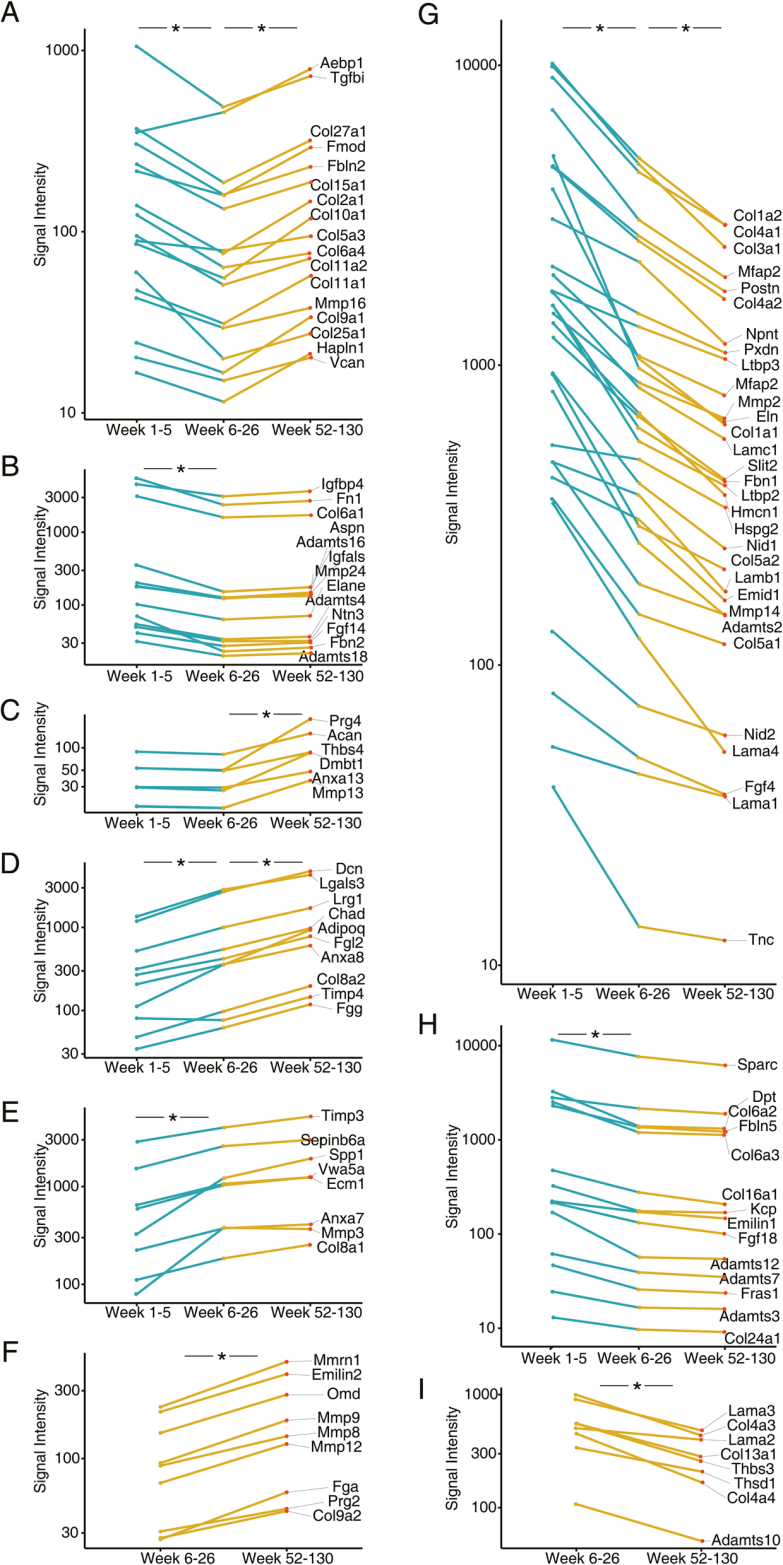
Age-dependent regulation of extracellular matrix coding genes. Shown is the regulation of 117 ECM coding gens in the lung of 89 individual mouse genomic data sets at different ages. These code for 28 collagens, 10 proteoglycans, 17 matrix metalloproteinase and 62 ECM-related glycoproteins. The data are expression values and the full genes names are given in supplementary Table S5. Note, significance testing is based on the LIMMA package. **A** V-shaped gene expression changes with age. The gene expression changes were significant among all age groups. **B** V-shaped gene expression changes with age. The gene expression changes were significant between mice aged 1–5 and 6–26 weeks. **C** V-shaped gene expression changes with age. The gene expression changes were significant between mice aged 6–26 weeks and old mice. **D** Continuously increased expression genes with age. The expression changes were significant between all age groups. **E** Continuously increased gene expression changes with age. The gene expression changes were significant between mice aged 1–5 and 6–26 weeks. **F** Continuously increased gene expression changes with age. The gene expression changes were significant between mice aged 6–26 weeks and old mice. **G** Continuously repressed gene expression changes with age. The expression changes were significant between all age groups. **H** Continuously repressed gene expression changes with age. The gene expression changes were significant between mice aged 1–5 and 6–26 weeks. **I** Continuously repressed gene expression changes with age. The gene expression changes were significant between week 6–26 and aged mice. *p < 0.05

To better understand the remodeling of extracellular matrix in the aging lung, we evaluated the expression of genes coding for collagens, proteoglycans, matrix metalloproteinase and their inhibitors in addition to glycoproteins over time. We considered the genomic data sets for N = 89 mice, and based on the linear regression model and the Limma statistical test we determined statistical significance for some ECM coding genes whose expression pattern followed a V-shape (Fig. 7A-C) in addition to continuously increased (Fig. 7D-F) and repressed transcript expression over time (Fig. 7G-I). Moreover, we identified a group of ECM coding genes which only increased in expression when 6–26 week old animals were compared to aged ones (Fig. 7F); in the same comparison some ECM coding genes declined in expression (Fig. 7I). Collectively, the genomic data informed on ECM remodeling in the aging lung (supplementary Table S5), and its increased deposition contributes to stiffness [44, 45].

#### V-shaped gene expression changes of ECM coding genes over time

Lung development and its organ function is highly dependent on the coordinated expression of ECM genes. For instance, independent research identified interstitial collagen (collagen I and III) to achieve maximal expression levels at day 7^th^ post partum, and this coincides with postnatal alveologenesis [45]. In the present study, several collagens followed a V-shape expression pattern, i.e. *Col2a1*, *Col6a4*, *Col9a1*, *Col10a1*, *Col11a1*, *Col11a2*, *Col15a1* and *Col25a1*, and the majority codes for proteins of the lung scaffold. However, we were surprised to see the regulation of Col2a1, Col9a1, Col10a1, Col11a1 and Col11a2 in the lung given its major role in cartilage matrix biology. Notwithstanding, independent research reported a 4-fold upregulation of *Col2a1* especially in aged mice [28] while in the present study a 2-fold upregulation was computed. Note, in the human lung upregulation of COL10A1 is part of the ECM remodeling, especially in lung cancer patients, and this collagen stimulates cell proliferation [46]. Alike, independent research revealed *Col10a1* as 1.9-fold increased in aged mice [5], which is similar to our data, i.e. 2.2-fold. Furthermore, collagen type V and VI are structural components of the connective tissue of the surrounding vascular and bronchial walls [47, 48] and are enriched in fibers of fibrotic lesion [47]. With age their augmented expression resulted in more dense and thick ECM fibrils. Therefore, the accumulation of these collagens contributes to lung stiffness [47]. Similarly, the lamina reticularis of the airway becomes thickened by the enhanced deposition of collagen I and V which limits airway gas exchange. It is part of an airway remodeling that eventually results in damage of the alveolar walls and the occurrence of emphysema [49]. The formation of heterotypic fibrils also included collagen I and collagen III, i.e. major collagens of the pulmonary ECM which declined with age (Fig. 7G). In fact, collagen VI forms a unique beaded-filament structure [50] and acts as a binding element between collagen I/III fibrils and basement membranes [48, 51]. Additionally, collagen VI promotes cell adhesive properties of the ECM [52] and contributes to epithelial cell homeostasis [50]. During lung fibrosis the expression of collagen VI is increased and typically forms fibrils with collagen III [48].

In the present study, the transcript expression of *Col6a4* increased whereas *Col6a1, Col6a2* and *Col6a3* decreased to a similar extent in aged mice (Fig. 7B and 7H), and independent research confirmed our findings at the protein level [5, 28]. Collagen VI is an essential basement membrane component, and Col15a1 acts as a biological ‘spring’ between the basement membrane and the interstitial border. It therefore contributes to the stabilization and protection of the pulmonary structure [53].

A further example relates to elastin which is highly expressed in the lung and required for alveogenesis. We compared neonatal mice to 6–26 week old ones and observed elastin mRNA expression declined by nearly 90% (Fig. 7G); and a similar finding was reported for the elastin protein in the lung of aged mice [28, 54]. Owing to its function in alveolar walls, a reduced elastin expression results in thinner and fragmented alveolar septa [55], and independent research showed a similar decline in elastin synthesis with age. As a result, a decreased tissue elastance was reported [28, 56], which leads to the regular airspace dilatation in the aging lung [57]. In fact, emphysema is characterized by reduced elasticity of the lung; however, the alveoli walls are destroyed mainly due to the degradation of elastin fibers [58]. Although lung compliance increases with age, the tissue elastance and tissue resistance decreases [56].

#### Continuously increased expression of ECM coding genes over time

Shown in Fig. 7D-F are upregulated ECM coding genes, and panel A illustrates the highly significant 3.5-fold upregulation of Leucine‐rich α‐2 glycoprotein (*Lrg1*). This glycoprotein plays an essential role in angiogenesis by modulating endothelial Tgfβ signaling [59]. In addition, recent research is suggestive for a link between Lrg1 expression and emphysema and inhibition of Lrg1 protected endothelial cells from vascular rarefaction (= malperfusion of vessels) and alveolar damage [60]. Additionally, Lrg1 promotes lung fibrosis following bleomycin treatment by influencing Tgfβ signaling in fibroblasts [61]. Strikingly, *Lrg1* ko mice are protected from lung fibrosis when given the same treatment. There is also evidence for Tnfα to induce expression of Lrg1, and therefore Lrg1 can be linked to tissue inflammation [62]. Over time, we observed a mild but significant increased *Tnfα* expression, and our study is suggestive for *Lrg1* to be a candidate gene for an age-related stiffness of the lung by instructing fibroblast proliferation. Importantly, we observed a 2.4- and 1.7-fold induced expression of the S100 calcium binding protein A4 in 6–26 week and aged mice, and it is of considerable importance that M2 polarized alveolar macrophages secreted S100a4 to stimulate lung fibroblast proliferation [63]. Correspondingly, inhibitors of S100a4 are explored as a therapeutic target for the treatment of lung fibrosis [64]. Furthermore, we observed asignificant upregulation of galectin 1 mRNA (*Lgals1*) in aged mice, and the coded protein induced fibroblast differentiation and increased ECM stiffness through an activation of the PI3-kinase and p38 MAPK pathway [65]. Indeed, independent research evidenced induction of galectin-1 by Tgfβ to be associated with fibroblast differentiation into myofibroblasts and nuclear retention of Smad2 [65]. However, in the present study *Smad2* was unchanged and *Smad3* was nearly 2-fold repressed. In addition, galectin 3 transcript expression increased continuously with age (> 3-fold, Fig. 7D). This protein functions in several biological processes including programmed cell death and innate immune response and promoted macrophage and fibroblast activity and ECM remodeling [66, 67]. Indeed, the Framingham Heart study revealed increased blood galectin-3 levels to be associated with restrictive lung disease and interstitial lung abnormalities [68].

Over time we calculated a nearly 4-fold increased decorin expression (Fig. 7D), and this proteoglycan is mainly produced by fibroblasts and functions as an inhibitor of Tgfβ to reduce fibrotic scares [69, 70]. In a landmark paper, the antifibrotic function of decorin was clearly established, i.e. transient transgene expression of decorin reduced the fibrotic response to bleomycin in the lung [71]. Similarly, in severe emphysema, decorin expression is reduced [72] to possibly aggravate the disease [73].

#### Continuous repression of ECM coding genes over time

Shown in Fig. 7G-I are ECM coding genes whose expression was repressed over time, and the majority code for the basement membrane. Specifically, the basal lamina of basement membranes contains laminin, and this heterotrimeric glycoprotein is composed of the α- (e.g., Lama1, Lama2, Lama4), β- (e.g., Lamb1) and γ-chain (e.g., Lamc1). Laminins are interfilament proteins and form polymers to support basement membrane function and are part of a mechanical scaffold [74, 75]. We found the laminins (Lama1, *Lama2*, *Lama4*, *Lamb1*, *Lamc1*), nidogens (*Nid1*, *Nid2*), collagen IV (*Col4a1*, *Col4a2*) and tenascin C (*Tnc*) to be repressed up to 6.5-fold, and all of these genes code for ECM of the basement membrane (Fig. 7G-I). Other investigators also reported repressed expression of components of the basement membrane. For instance, Godin and colleagues [28] cultured human bronchial epithelial cells and lung fibroblasts on ECM derived from young and aged lungs. The cells cultured on ECM derived from the lung of aged mice resulted in reduced expression of laminins, especially laminin α3 and α4. In fact, most laminins in the lung of aged mice were repressed as evidenced by mass spectrometry, and these findings confirm the results of the present study. Together, the gene expression changes contributed to age-related ECM remodeling and provide a molecular rationale for age-related decline in lung function. A further example refers to the glycoprotein nidogen which forms a scaffold with laminins and collagen IV in the basal lamina and supports epithelial cell-fibronectin interactions [76]. Moreover, research confirmed reduced laminin, nidogen and collagen VI protein expression in the aging lung, and this signifies the remodeling of the basal lamina to possible influence tissue regeneration [5, 28].

Additionally, the glycoprotein tenascin C (Tnc) is part of the ECM of the lamina reticularis and the matrix influences cell adhesion and proliferation [77, 78]. Typically, Tnc is highly expressed during branching morphogenesis and alveogenesis, however is barely detectable in neonates as evidenced by immunoblotting on day 21 post partum [79]. Similarly, we observed a 4-fold decline in *Tnc* expression over time and consider its regulation as a physiological process.

A further example relates to *Mmp2*, *Mmp14* and *Adamts2* which were repressed to about 20–30% of controls in the lung of aged mice (Fig. 7G), and these metalloproteinases degrade matrixproteins including collagens (I-V, VII, X-XI), fibronectin, laminin, elastin [80]. The regulation of matrix metalloproteinases in the lung have been the subjected of several reviews [81], and Mmp2 and Mmp14 are mainly expressed during lung development but decline sharply after birth. In fact, Mmp14 activates Mmp2 and apart from their role in lung morphogenesis such as lung alveolar septae development [82] research demonstrated *Mmp2* deletion to reduce cellular infiltration and fibrosis in an allotransplant model [83]. Similarly, Mmp14 is highly expressed on lung endothelial cells and degrades collagen I [84], which supports the migration of cells through dense connective tissue while Mmp2 degrades elastin and collagen fibers and therefore contributes to ECM remodeling. Moreover, we observed repressed *Adamts2* expression, and this disintegrin and metalloproteinase with thrombospondin motifs cleaves the propeptide region of collagen I, II and III and therefore allows collagen fibril formation and maturation. Its decreased expression in aged mice was also observed by other investigators [85], and owing to its function, we speculate a defective ECM turnover activity and changed ECM composition that will affect airway biology in different ways, i.e. cell adhesion, cell signaling etc.

Unlike the human lung (see below), we observed significantly repressed transcript expression of *Col1a1* and *Col3a1* in the aged lung of mice, and our findings agree with the data reported by other investigators [29, 85–88].

### ECM remodeling in the aging human lung

**Fig. 8:**
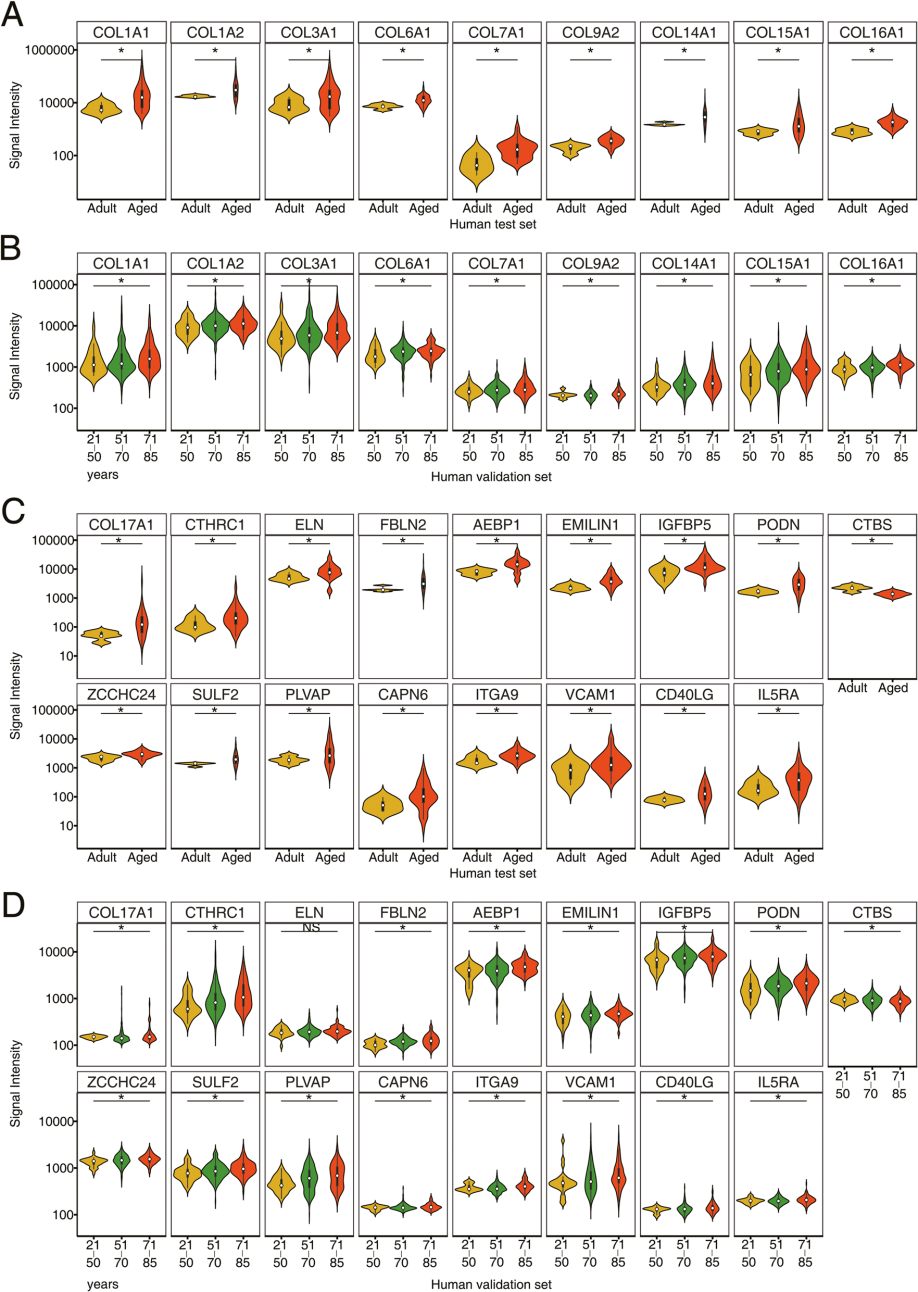
Violin plots of commonly regulated DEGs between the human lung test and validation set. Shown is the regulation of 26 commonly regulated genes among adult and aged individuals. Because we determined their regulation by two different platforms, i.e. RNAseq and microarrays, we show the data separately and give the full name of the genes in supplementary Table S6. Statistical testing is based on the DEseq for the RNAseq-data and the linear regression model for the human validation set. **A** Regulation of collagen coding genes among adult and aged individuals as determined by RNAseq in the human test set genomic data. **B** Regulation of collagen coding genes among adult and aged individuals as determined with linear regression model in the human validation set genomic data. **C** Gene expression changes of 17 individual genes among adult and aged individuals as determined by RNAseq for the human test set genomic data. **D** Gene expression changes of 17 individual genes among adult and aged individuals as determined for the human validation set genomic data. *p < 0.05. NS: not significant

As described above, we identified 26 genes commonly regulated between the human test and validation set (*N* = 307) (supplementary Table S6). Fourteen code for ECM components, notably several collagens, i.e. *COL1A1, COL1A2, COL3A1, COL6A1, COL7A1, COL9A2, COL14A1, COL15A1, COL16A1, COL17A1* and the collagen triple helix repeat containing 1 (*CTHRC1*), all of which were significantly upregulated in aged individuals. The results for the human test and validation set are shown in Fig. 8A-8D, and the data indicates increased extracellular matrix deposition with age. Given the significant upregulation of *COl1A1* and *COL1A2* in lung tissue of aged individuals, we obtained evidence for an increased expression of type I collagens during aging. Note, collagen type I forms a triple helix of two COL1A1 and one alpha 2 chain (COL1A2) and is abundantly expressed in the lung [89] where it contributes to the rigidity and elasticity of the pulmonary framework. Among the different cells of the lung, activated fibroblasts or myofibroblasts produce high levels of collagen, especially collagen type I and III, and typically the fibroblasts are located in the interstitial space around the alveolar septae and become activated in the process of wound repair. Additionally, mesenchymal cells, smooth muscle cells [90], pericytes [91] and macrophages [92] are able to synthesize collagens; however, they do not play a major role in wound repair. Initially, collagen type III is deposited in the early course of wound repair, followed by collagen type I to prepare a more rigid fiber network [93]. In the current study we found collagen 3A1 upregulated in aged individuals and therefore confirm the notion of an increased expression of collagens with age. Notwithstanding, the remodeling of the ECM appears to be selective in regards to the collagens involved. Comparable results were reported for mice [29, 86].

A further interesting result relates to the induced expression of *CTHRC1* (Fig. 8C, test set and 8D, validation set). This protein limits collagen matrix deposition and promotes cell migration as shown in studies with balloon-injured arteries of rats [94]. The protein is secreted in response to injury and blocks excessive collagen matrix deposition. As of today, the role of this protein in normal lung biology is not well understood; however its upregulation in the lung following exposure to bleomycin has been reported, and single cell transcriptome analysis evidenced induced *Cthrc1*-expression to be confined to fibroblasts while other cells of the lung do not express the protein [95]. Moreover, *Cthrc1* ko mice were protected from lung fibrosis following bleomycin treatment [96]. Furthermore, Caporarello and colleagues reported young and aged mice to differ in the expression of collagens following bleomycin treatment with aged mice expressing Col1a1 protein more abundantly [97]. This implies age-related differences in the wound repair. Collectively, its upregulation in aged individuals indicates a protective mechanism to a life time exposure to airborne pollutants and other materials causing inflammation and the associated wound repair. Notwithstanding, Cthrc1 also supports migration and adhesion activities of certain cancer cells [98].

In aged individuals, we found *COL6A1* nearly 2-fold increased and independent research confirmed its upregulation in a combined transcriptome and proteome study of 9 human lung tissue samples [99]. Therefore, our data agrees with findings reported by others, and this collagen is a structural component of the basement membrane. Strikingly, soluble collagen VI protected fibroblasts from apoptosis in serum-starved cultures by inhibiting the activity of the pro-apoptotic protein bax [100, 101]. It is tempting to speculate that the upregulation of this collagen in the lungs of aged individuals protected fibroblasts from cell death. We also observed an age-dependent increased expression of *COL7A1, i.e.* an anchoring fibril protein and structural component of the basement membrane (Fig. 8A). A further example relates to the about 2-fold induced expression of *COL15A1* and *COL16A1*. These collagens connect the basement membrane of the respiratory epithelium with the surrounding connective tissue.

Recently, studies with human airway smooth muscle cells from COPD (n = 7) and non-COPD (n = 7) susceptible smokers demonstrated TGFβ treatment of cell cultures to significantly stimulate *COL15A1* transcript expression, and the induced gene expression is modulated by histone H4 acetylation. Correspondingly, there are epigenetic control mechanisms in the ECM remodeling of the aging lung [102]. Furthermore, COL16A1 stabilizes collagen fibrils and anchoring microfibrils of the basement membrane, and its upregulation highlights the ECM remodeling related to the basement membrane. A proteomic study revealed Col16a1 19-fold induced in the lung of aged mice [5], and in the present study we observed a 2-fold induced expression for the aged human lung. Interestingly, in a very recent study, age-associated differences in the human lung extracellular matrix were reported [103], and the results agree with our findings for *COL1A1*, *COL6A1*, *COL14A1* and *FBLN2*.

Additional examples of significantly regulated DEGs include adipocyte enhancer-binding protein 1 (*AEBP1*), fibulin 2 (*FBLN2*) and coiled-coil domain containing 80 (*CCDC80*)*.* However, *CCDC80* was only significant in the human test set data. Specifically, AEBP1 is a protein secreted by fibroblast and myofibroblasts [104] and functions in wound healing and myofibroblast differentiation. It is of considerable importance that *Aebp1* deficient mice are protected from lung injury following bleomycin treatment [104], and in the present study, we observed a nearly 2-fold upregulation of *AEBP1* in aged individuals. For its role in augmenting myofibroblast activation, i.e. smooth muscle actin (SMA) expression and collagen deposition [104], we view its upregulation as a sign of defective wound repair in aged individuals that have been exposed for a life time to airborne pollutants and other harmful agents and materials. AEBP1 may also qualify as a marker of lung stiffness.

Another example relates to fibulin 2, and this secreted glycoprotein functions in elastic fibers of alveolar interstitium, airway and vessel walls, and has been reported to stabilize the basement membrane of mammary epithelium [105]. Interestingly, studies in ko mice revealed fibulin 2 not to be required for elastic fiber formation [106], and therefore a functional redundancy between fibulin members exists. We observed a significant upregulation of fibulin 2 in aged individuals and likewise elastin was significantly increased which implies a coordinate expression of both fibers with age. Note, the results are opposite to that obtained for mice (see above).

Lastly, we wish to highlight the regulation of coiled-coil domain containing 80, and this protein functions in matrix assembly and promotes cell adhesion while loss of *Ccdc80* negatively modulates glucose homeostasis in diet-induced obese mice [107]. CCDC80 is a component of the basement membrane and regulates matrix assembly and cell adhesion through binding with ECM molecules as shown in the 293T cell line [108]. We observed increased pulmonary expression of *CCDC80,* and a similar increase in plasma CCDC80 protein expression has been reported for aged individuals [109]. In fact, ongoing studies evaluate CCDC80 as a prognostic biomarker of multimorbid individuals [110].

Because of its structural domains, CCDC80 gained considerable interest as a thioredoxin-like antioxidant which may function as a tumor suppressor in some organs [108, 111]. Gene knockdown of *ccdc80* in zebrafish caused decreased expression of *Col1a1* while immunohistochemistry studies in a rat model of pulmonary hypertension demonstrated Ccdc80 protein to be induced, thus implying different roles of this protein in the pathogenesis of pulmonary hypertension and vascular remodeling [112]. Furthermore, a study with *Ccdc80* ko mice revealed this protein to function in lipid metabolism, and cytokine-cytokine receptor interactions which implies a role in inflammation [113].

### Comparison of ECM regulated genes and coded proteins in the aging mouse lung

Apart from considering ECM gene expression changes, we also determined whether ECM regulated genes are regulated at the protein level in lung tissue of mice. In this regard the data of Schiller and colleagues were highly informative [58, 114]. In fact most of the ECM coding genes reported in the presented study are also regulated at the protein level as evidenced by mass spectrometry. Apart from their identification, we wished to rank the relative abundance of the ECM coding genes by normalizing the signal intensities to a set of housekeeping genes, which were constantly expressed with age. We considered the expression of about 798 housekeeping genes (supplementary Table S7) which remained constant over time and inferred changes in the composition of the ECM based on the changes in transcript abundance relative to the housekeeping genes. Specifically, we considered the data of Booth and colleagues [115] who identified 96 proteins in the matrisome of the normal mouse lung, and based on other published works [58, 80, 115] compiled 256 proteins that code for extracellular matrix of which 156 are significantly changed at the transcript level over time (see supplementary Table S8). Among the age-related gene expression changes, we identified 63 as upregulated (Fig. 7A-F) of which decorin is an interesting example. Its relative abundance increased consistently over time and as detailed above, decorin functions as an inhibitor of Tgfβ to reduce fibrotic scares [69, 70]. Another example relates to the microfibril associated proteins *Mfap-2* and *Mfap4﻿,* and these glycoproteins colocalizes with elastin and decreased continuously during the aging process (Fig. 7G). The relative changes in transcript abundance for *Mfap2* were 1.5%, 1.2% and 0.9% when animals aged 1–5 were compared to 6–26 week old mice and old mice, respectively. Our data is in agreement with the original works of Mecham and Gibson [116] who reported *Mfap2* expression to be highest in neonatal mice. It is interesting that *Mfap2* knockouts are almost indistinguishable from wild type mice, and the knockouts form microfibrils with elastic fibers [116, 117]. Alike, *Mfap4* changed with age from 5.1% to 3.5% and 2.4%, and although Mfap4 is one of the most abundant ECM components it declined over time. Our findings agree with the results reported by Burgstaller and colleagues [58]. The fact that Mfap4 declines over time is puzzling. Essentially, studies with *Mfap4* knockouts revealed a significant decrease in alveolar surface area by 25%, and mice developed emphysema-like changes at the age of 6 months [118]. Interestingly, research dating back more than 30 years already demonstrated a decline in alveolar surface area during life [119]. Furthermore, the elastin content differed significantly between 3 month old wt and *Mfap4* ko mice even though electron microscopy did not evidence altered elastic fiber organization. In the present study we calculated an elastin content in the lung of about 3.6% for 1–5 week old mice which is similar to the protein expression data reported by Mecham [120]. However, over time we observed a drastic decline in elastin gene expression by nearly 10-fold (Fig. 7G) and therefore investigated the regulation of Tgfβ and Smad proteins, which are known to influence elastin transcription [121]. Essentially, transcript expression *Eln* and *Smad3* were positively but loosely correlated (R^2^ = 0.4) while *Eln* and *Smad6* were negatively correlated (R^2^ = 0.2). Smad3 is a mediator of Tgfβ signaling [122, 123] and deficiency of Smad3 represses tropoelastin expression. Conversely, Smad6 inhibits Tgfβ signaling [122, 123], and we observed a positive correlation between *Smad6* and *Tgfβ* transcript expression (*R* = 0.7).

### Senescence and senescence-associated secretory phenotype (SASP)

Cellular senescence is characterized by a stable cell cycle arrest [124], and the link between cellular stress responses and senescence has been the subject of several reviews [125–128]. For instance, replicative senescence may result from progressive telomere shortening, and at a critical telomere length the resultant genomic instability causes an activation of DNA damage programs. Additionally, premature senescence can arise from oxidative genotoxic stress and lack of homeostasis [129]. Typically, senescent cells fail in the proper functioning of metabolism, and with age the senescence-associated secretory phenotype (SASP) becomes activated [130]. The SASP comprises a range of inflammatory molecules including interleukins, chemokines, growth factors, proteases and in their review, Coppe et al. emphasized the multiple stimuli that provoke senescence, i.e. chromatin instability, telomere dysfunction, overexpressed cell cycle inhibitors, non-telomeric DNA damage and other stress signals [128]. Similarly, the works of Kumar et al. focused on the possible role of SASP in COPD [131], and based on these reviews, we assembled a list of SASP coding genes. We found 53 and 22 genes as significantly regulated, of which 31 and 12, respectively were upregulated in the lungs of mice and humans (supplementary Table S9).

In the aging lung of mice most SASP molecules code for inflammation, and examples of significantly upregulated genes include the interleukins Il1b, Il7, chemokines *Ccl8, Ccl11, Ccl13, Ccl20*, C-X-C motif chemokine ligand 2, 13 (*Cxcl2, Cxcl13*), the matrix metalloproteinase *Mmp12, Mmp13,* interleukin 6 cytokine family signal transducer, angiogenin, insulin like growth factor binding protein 6 and cathepsin B. Conversely, *Cxcl15*, hepatocyte growth factor, the insulin growth factor binding protein 4, the metalloproteinase *Mmp14* and *Fn1* were > 2-fold repressed. Collectively, we considered 71 SASP molecules (supplementary Table S9) of which 19 were regulated by ≥ 2-fold. Their regulation signifies inflammation and inhibition of growth. Specifically, Ccl8, Ccl11, Ccl13, Ccl20 function as chemoattractantants and recruit monocytes/histiocytes to sites of injury. These chemokines bind to the chemokine receptor 2, 3, 5 and 6 and display partially overlapping functions. Nonetheless, there are also important differences with Ccl8 and Cc13 functioning as a chemoattractant for monocytes, eosinophils and T cells, whereas Ccl20 stimulates the homing of lymphocytes and dendritic cells to senescent airway epithelial cells.

A further example relates to the C–C Motif Chemokine Receptor 2 (Ccr2), which is predominantly expressed in macrophages, fibroblasts and endothelial cells. We observed nearly 4-fold increased *Ccr2* expression in aged mice when compared to 1–5 week old ones. Additionally, we observed an induced expression in *Ccl2* and this cytokine binds to the Ccr2 receptor and attracts myeloid and lymphoid cells to sites of inflammation [132]. Indeed, treatment of radiation-injured mice with an Ccl2 inhibitor as well as studies in *Ccr2* knockout mice evidenced inhibition of Ccl2 signaling to result in reduced lung inflammation, normalization of endothelial cell morphology and vascular function [133]. Another study reported the senescence of umbilical cord blood-derived MSCs to be dependent on the activity of Ccl2, and this cytokine is epigenetically regulated by the polycomb protein BMI1 [134]. Furthermore, *Ccr2* ko mice exposed to 85% O_2_ for up to 6 days were protected against hyperoxia-induced tissue injury though inhibition of iNOS and subsequent ROS production [135].

Another example relates to the significantly induced expression of C-X-C Motif Chemokine Ligand *(Cxcl)-1)*. This cytokine attracts neutrophils to sites of inflammation [136]. In aged mice, we observed a slight increase in the expression of its receptor, i.e. *Cxcr2*, and clinical research evidenced its ligand Cxcl1 to be induced in the progression of interstitial pneumonia with autoimmune features [137]. Moreover, ectopic expression of Cxcr2 caused premature senescence via a p53-dependent mechanism whereas *Cxcr2* knockdown reinstalled cell proliferation [138].

As part of the cellular senescence program, the insulin-like growth factor binding proteins (IGFBP) are regulated, and in the present study, we observed induced expression of *Igfbp-2, Igfbp3, Igfbp4, Igfbp6* and *Igfbp7* in aged mice and human lung tissue [139–141]. Unfortunately, the exact mechanism by which Igfbps influences senescence are unknown and genetic loss-of-function-studies are inconclusive [142]. For instance, *Igfbp3*, *4*, and *5* triple knockouts resulted in a 25% reduction in body growth, decreased fat accumulation, but enhanced glucose homeostasis [142]. Likewise, through binding with insulin like growth factor (IGF)-1, IGFBP2 inhibits the binding between IGF1 and insulin like growth factor 1 receptor (IGF1R), and this reduces cell survival and mitogenesis [143]. One study reported induction of cellular senescence by Igfbp5 through a p53-dependent mechanism [144]. On the other hand, overexpression of Igfbp6 delayed replicative senescence of human fibroblasts and might therefore be a negative regulator of senescence [145].

Although we already addressed the importance of extracellular remodeling in the aging lung (see above), we wish to highlight the marked induction of the matrix metalloproteinase *Mmp3*, *Mmp10*, *Mmp12, Mmp13* and its inhibitor *Timp1* in the lung of aged mice and humans. The up to 5-fold induced expression of these metalloproteinases implies a senescence related change in ECM composition with obvious implications for its mechanical properties. Moreover, ECM remodeling facilitated extravasation and migration of inflammatory cells [81, 146]. Thus, senescence is hallmarked by various inflammatory responses, and in the present study we identified *Ccl11* (*Eotaxin*) and *Cxcl13* (*Blc*) as highly regulated in the aging lung of mice (supplementary Table S9). Their expression increased by 2.3- and 10-fold, respectively and are synthesized by various pulmonary cells including macrophages [147]. Ccl11 expression increased in mesenchymal stromal cells of aged lung [148] and attracted eosinophils to sites of inflammation [149, 150]. This cytokine mediates the migration of NK and monocytes to senescent cells [148].

Unlike Ccl11, the proinflammatory cytokine Cxcl13 supports maturation and B-cell homing to inflammatory foci [151] and augments IgM and IgA responses following cytokine signaling [152]. It is of considerable importance that the senescence related gene regulations described above were also observed by other investigators with some genes being > 20-fold induced in the aged lung of mice [5].

### Transcription factor networks and chromatin/telomerase modifiers in SASP

**Fig. 9:**
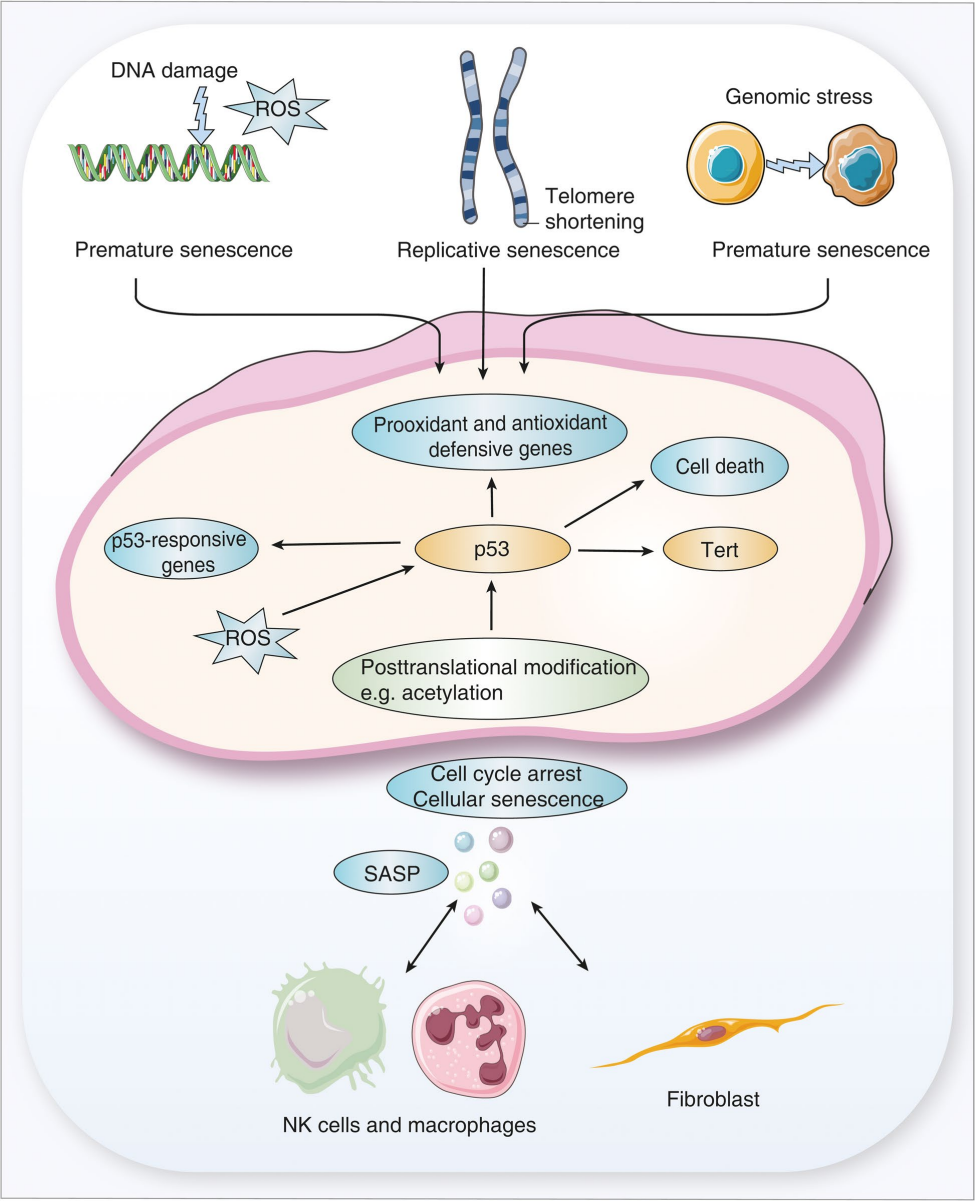
A simplified scheme of p53 activity and chromatin modifiers in the aging lung. p53 plays a central role in cellular senescence and reactive oxygen species (ROS) induced cellular stress. This stimulates p53 dependent activation of antioxidant defense genes. Telomere shortening leads to replicative senescence, DNA damage response (DDR) and cellular aging. Telomerase reverse transcriptase (Tert) restores telomere length following end capping. Senescent cells secrete a range of pro-inflammatory molecules subsumed under the so-called senescence-associated secretory phenotype (SASP)

Figure 9 depicts a simplified scheme of key transcription factors and chromatin modifiers deregulated during senescence. Undoubtedly, p53 plays a central role in cellular senescence [153], and it is well established that p53 functions as a guardian of genomic stability. We observed induced *p53* expression in the aging lung of mice. Note, ROS induced cellular stress stimulates p53 activity and the expression of antioxidant defense genes (Fig. 9). However, if cells are significantly damaged, p53 will support programmed cell death. Therefore, p53 takes on a dual role in senescence, and its activity depends on posttranslational modifications, i.e. acetylation [154] whereas sirtuin 1 (Sirt1) deacetylases p53 thereby inhibiting its activity. *Sirt1* is significantly induced in the lung of aged mice and activation of SIRT1 attenuates inflammaging in chronic inflammatory diseases [155]. In fact, SIRT1 is down-regulated by autophagy in senescence and ageing [156].

Among the antioxidant defensive genes, we observed significantly repressed expression of superoxide dismutase and of NADPH oxidase 4. The p53 inducible nuclear protein (*Trp53inp1*) was nearly 2-fold repressed and this protein acts as a positive regulator of autophagy [157, 158]. Furthermore, autophagy-related ubiquitin-like modifier LC3B is significantly repressed (supplementary Table S10), and this suggests an inadequate autophagy in aged lungs. The role of defective autophagy in senescence and disease has been the subject of a recent review [159]. In senescent cells, the expression of the p53 and cyclin dependent kinase inhibitor 1A (p21) protein is transient [160] while other cyclin dependent kinase (CDK) inhibitors become activated. However, in the present study the cyclin dependent kinase inhibitor 2A (*p16*) was basically unchanged (supplementary Table S10), and the cell cycle regulator *Cdk1* and p*21* were 2-fold repressed in the lung of aged mice to possibly delay senescence.

Moreover, we observed repressed sestrin 1 and 2 gene expression in the lung of aged mice, and the coded proteins bind directly to aminoacids and inhibit the target of rapamycin complex 1 with major implications for autophagy, i.e. lack of its activation. Therefore, we obtained further evidence for an impaired autophagy during senescence [161].

Unlike mice, most of the antioxidant defensive genes were unchanged in the human lung, eventhough we observed mildly repressed superoxide dismutase 1 (*SOD1*) and induced *P16* in the aged human lung.

We already emphasized the interplay of chronic inflammation and cellular senescence and next to various histones and p53, the Sirt1 deacteylase also influenced the activity of forkhead box O3 (FOXO3), NFκB and other proteins involved in DNA damage and repair responses [162]. Specifically, FOXO3 functions in the detoxification of oxidative stress, and research demonstrated Foxo3-deletion to result in ROS damage and ROS-induced reduction of the lifespan of erythrocytes [163]. Similar results were obtained for the lung and restoration of FOXO3 activity blocked bleomycin-induced fibrosis and reverted the idiopathic pulmonary fibrosis myofibroblast phenotype [164]. Therefore, FOXO3 is explored as a therapeutic target, and in the lung of aged mice we found *Foxo3* significantly increased.

Another target of Sirt1 is the peroxisome proliferator-activated receptor γ coactivator-1α *(Pgc1)* [165], and its expression is signficiantly induced in the lung of aged mice. Pgc1 functions as a transcriptional coactivator to stimulate mitochondrial biogenesis, lipid metabolism and to improve ATP production [166], and the SIRT1/PGC-1α/PPAR-γ signaling pathway is essential in senescence. Importantly, Sirt1 deacetylates Pgc1 thereby stimulating its activity and Sirt1 and Pgc1 counteract senescence [167]. Therefore, key players in overting senescence are induced in the lung of aged mice [168, 169].

A further point of considerable importance is the relationship between telomere size and cellular senescence, and owing to its function, the telomere size of chromosomes is considered to be of critical importance in the control of genomic stability. Its shortening will lead to replicative senescence, although more recent studies challenge this paradigm [170]. Telomere dysfunction leads to DNA damage response (DDR), and cellular aging [171].

One study investigated the telomere length of chromosomes in lung tissue of normal aged individuals and patients undergoing lung transplantation, and evidence for its progressive shortening was obtained. However, the relative telomere length did not correlate with regional disease severity [172]. Furthermore, expression of the telomerase reverse transcriptase (*Tert*), i.e. an enzyme complex that re-elongates shortened telomeres was significantly increased in aged mice. Additionally, grainyhead like transcription factor 2 (*Grhl2*) and paired box 8 (*Pax8*) are transcription factors which activate the Tert promoter. Given their increased expression in the lung of aged mice, we obtained evidence for a coordinate regulation of Tert with induced expression of its transcription factors.

Another factor that functions in telomere maintenance and protection against end-to-end fusion of chromosomes is telomeric repeat binding factor (Terf). Both isoforms, i.e. *Terf1* and *Terf2* were significantly but oppositely regulated in aged mice, and this is suggestive for an impaired telomere maintenance in aged animals and augmented senescence.

Unlike mice, most of the senescence associated genes are not regulated in the human lung. Nonetheless, we identified three upregulated genes in the test and validation sets, and all are p53 responsive genes. Specifically, sulfatase SULF2 is an enzyme which removes 6-O-sulfates from heparan sulfate (HS). This sulfatase is a p53 target gene [173] and its repression causes an impaired senescence response to genotoxic stress [174]. In fact, *SULF2* transcript expression increased mildly (1,5-fold) but significantly in the lung of aged individuals, and studies in mice ascribe SULF2 a protective role in epithelial cells of the lung following bleomycin induced injury [175].

The second gene codes for the Odd-Skipped related transcription factor 1 (OSR1, only significant in the human validation set). OSR is mildly but statistically significant upregulated, and its exact function in lung biology has not been established as yet. Nonetheless, it is highly expressed in the human lung [176] and was reported to inhibit lung cancer proliferation and invasion by reducing Wnt signaling through the suppression of SOX9 and β-catenin signaling pathways [177].

The third p53 target gene codes for NYN domain and retroviral integrase containing protein. There is only very limited information on the role of this retroviral integrase catalytic domain-containing protein other than it possesses nucleic acid binding ability and we found its expression 1, 7-fold increased in the human test set data.

### Age-related changes in the expression of immune cell marker genes

We interrogated four different databases to retrieve gene markers of different immune cells in the lung [5, 178–180] and searched for the consensus among them (supplementary Table S11, S12). Subsequently, we applied single sample gene set enrichment analysis (ssGSEA) and evaluated expression changes of the gene markers over time. The data allowed us to infer changes in the immune cell composition/phenotypes in the aging lung of mice. Given that about 36% of the gene markers are mutually expressed between various immune cells, we also performed the same computations by removing commonly expressed marker genes among the different immune cells and found the results between the two approaches to be comparable.

We evaluated age-related changes of marker genes on the basis of the enrichment score for individual immune cells (supplementary Table S13) and confirmed independently the results by interrogating single cell RNAseq data for pulmonary resident cells. We tested statistical significance with the Kolmogorov–Smirnov test, which assess the distribution of gene markers by comparing their position and distribution in individual lung samples from young and aged animals. Furthermore, we considered the distribution of enrichment scores, and if not normally distributed, adopted a non-parametric “Wilcoxon rank-sum” test to discover significant changes. We therefore infer an age-related change in an enrichment score with alteration in immune cell responses of the lung.

**Fig. 10:**
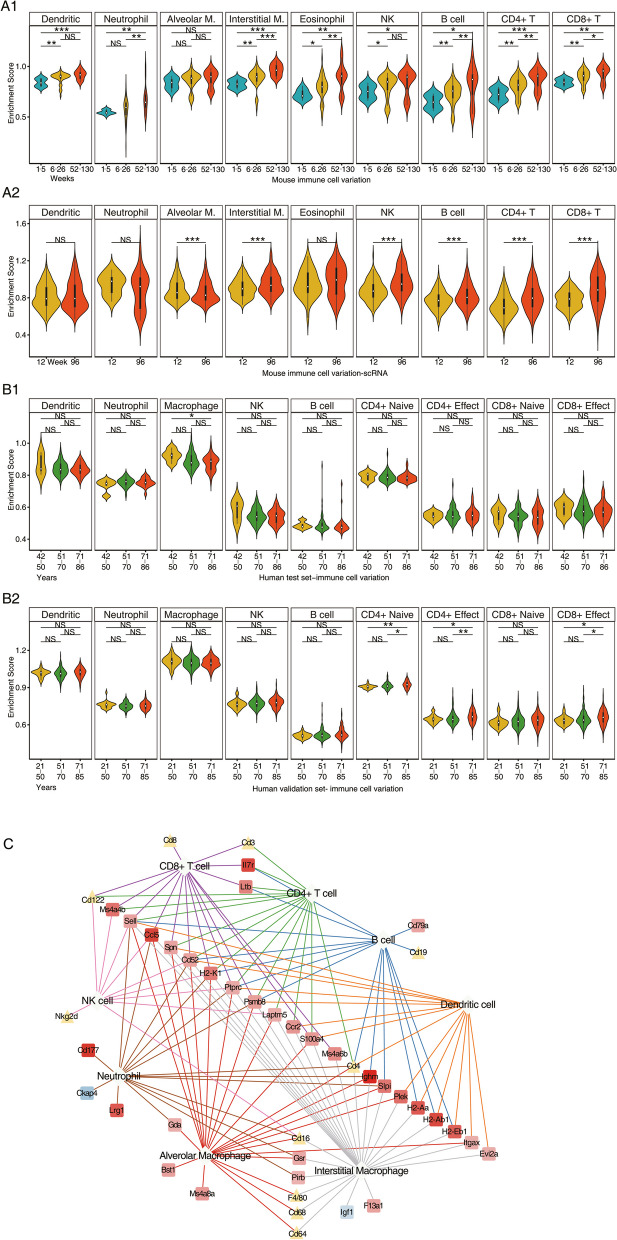

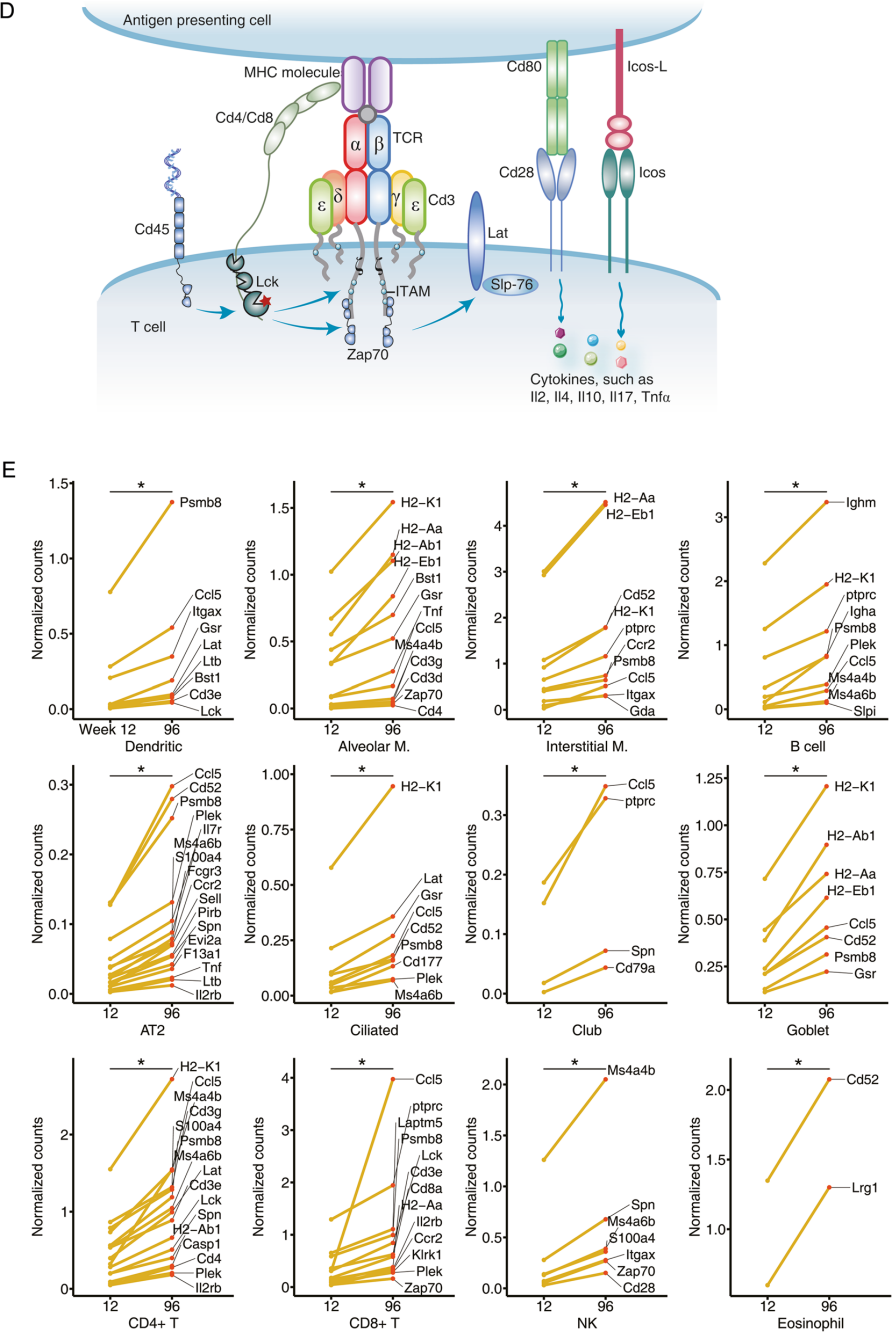
Age-dependent regulation of immune cell marker genes. For each immune cell, we retrieved marker genes from various databases (range 61–194 genes for the mouse and 133–1574 genes for human cells) and compiled information for individual marker genes in supplementary Table S12. The data represent enrichment scores based on signal intensities for individual genes of 89 individual mouse and 414 individual human genomic data sets. The enrichment score of marker genes infers a change in immune cell activity with age. To obtain independent validation, we analyzed single cell RNAseq data of pulmonary immune cells. **A** Panel A1: Depicted are violin plots of an age-related increase of enrichment scores for 9 different immune cells of the mouse. Panel A2: Depicted are violin plots of age-related variation of enrichment scores among 9 individual immune cells based on single cell RNAseq data. **B** Panel B1: Depicted are violin plots of an age-related variation of enrichment scores for 9 different immune cells of the human test set. Panel B2: Depicted are violin plots of an age-related variation of enrichment scores for 9 different immune cells of the human validation set. **C** Regulation of immune cell marker genes. Shown are commonly expressed gene markers between various immune cells in the lung of aged mice. The full genes names are given in supplementary Table S10. **D** The mouse CD3-T-cell receptor complex. Depicted are 20 upregulated genes coding for the CD3-TCR complex in the aging lung of mice. Activation of the CD3-TCR requires phosphorylation of the immunoreceptor tyrosine-based activation motif (ITAMs), and the lymphocyte-specific protein tyrosine kinase (Lck) catalyzes this reaction. Initially, the protein tyrosine phosphatase receptor type C (Cd45) activates Lck, and this phosphatase dephosphorylates the inhibitory C-terminal tail of Lck. Upon its activation, Lck phosphorylates the Zeta chain of T cell receptor associated protein (Zap70) and the Zap70 kinase phosphorylates linker for activation of T cells (Lat) and the lymphocyte cytosolic protein 2 (Slp76), which functions as a scaffold for signaling molecules. Moreover, Cd4 and Cd8 act as co-receptors to amplify TCR signaling. The co-stimulatory molecules Cd28, Cd80, Icos and Icos-L in addition to cytokines stimulate T cells activation and differentiation. **E** Independent validation of 61 immune response genes of the mouse genome by single cell RNAseq. Based on single cell RNAseq, we confirmed the regulation of immune response genes among individual immune and other resident cells of the lung. We compared the genomes of animals aged 12 weeks to 96 week old ones and assessed the expression of 61 immune response genes among dendritic, alveolar and interstitial macrophages, CD4^+^ and CD8^+^ T-cells, B-cells, NK, eosinophilic granulocytes, endothelial, AT1 and AT2 cells. Statistical significance testing: “Wilcoxon rank-sum” test. ns: not significant, *p < 0.05, **p < 0.01, ***p < 0.001. M.: Macrophage

Figure 10A, B depicts enrichment scores as violin plots for different immune cells. The violin plots shown in panel A refer to age-dependent changes in the enrichment score for gene marker sets of individual cell types. The individual genes are given in supplementary Table S12, and in the case of dendric cells and alveolar macrophages consisted of 194 and 158 ones, respectively. We independently confirmed their regulation by interrogating single-cell RNAseq data (Fig. 10A2) and the enrichment scores are significantly increased in animals aged 52–130 weeks. The data implies altered immune cell activity with age, and the change in enrichment scores in the lung of aged animals is suggestive for an age-related induced expression of gene markers of immune cells. Independent research found similar age-related changes in the expression of immune cell gene markers, and various investigators coined the phrase of “inflammaging” which implies an age-related sterile and chronic upregulation of pro-inflammatory cytokines [181]. Depicted in Fig. 10B1 and B2 are the results for the human test and validation set of 107 and 307 lung samples, respectively. Here, the data are less obvious and except for the reduction of enrichment score for the macrophage gene marker set of the test set (Fig. 10B1) could be ascertained. On the other hand, for the human validation set the marker genes for CD4^+^ naive, CD4^+^ effect and CD8^+^ effect cells increased significantly (Fig. 10B2).

One of the reasons for the differences between humans and mice is the large variation in the expression of marker genes seen with human lung samples (see the violin plots). Another point relates to the different methods employed, i.e. RNA-seq (Fig. 10B1 for the test set) and the microarray platform (Fig. 10B2 for the validation set). Therefore, we could not combine the data. Nonetheless, the results for the macrophage gene markers are in line with research reported by others. With age, the number of alveolar macrophages declined [182]; the various aspects of immunosenescence especially of macrophages was the subject of a recent review [183].

Figure 10C visualizes commonly expressed gene markers for various immune cells of the mouse lung. In total we observed 31 marker genes which increased continuously with age among 89 individual samples, and this included some highly regulated ones such as interleukin 7 receptor alpha chain (*Il7r*) which increased by 4-fold. Il7r is a key player in the maturation of B and T cells, and it was demonstrated that its expression is positively correlated with immune cell infiltration in the tumor microenvironment of lung adenocarcinoma [184]. Moreover, in the lung of aged mice, we observed > 3-fold induced expression of Ccl5, and this potent inducer of neutrophile migration stimulates alveolar macrophage activation [185]. Furthermore, we observed approximately 7- and 4-fold increases in the expression of the neutrophils marker genes *Cd177* and *Lrg1,* and observed upregulation of the major histocompatibility class II molecules, i.e. *HLA-DRB1*, *HLA-DQA1* and *HLA-DQB2*. Note we converted the mouse MHC class II to human ones based on the NCBI database and annotations reported in [186]. These MHC molecules are of critical importance in presenting antigens to the immune system. Although the significance of an induced expression of MHC molecules is far from clear, Jurewicz & Stern suggested that an upregulation of MHC-II-peptide complexes serves to focus the peptide repertoire and its processing by lymphocytes [187]. In general, the upregulation of MHC II molecules allows for the effective presentation of antigens. Strikingly, the genes coding for the immunoglobulin heavy constant mu (*Ighm*), immunoglobulin heavy constant alpha (*Igha*) and immunoglobulin heavy constant gamma (*Ighg*) were > 20-fold induced in the lung of aged mice. Typically, these are expressed on preB and B cells and contribute to immunoglobulin receptor binding activity. Their significant upregulation emphasizes inflammaging. We also observed a 2.3-fold upregulation of *Cd68* in the lung of aged mice, and the coded protein function as a scavenger receptor. CD68 is a specific macrophage marker and apart from its roles in immune response, the CD68 scavenger receptor phagocytoses cell debri and lipids. Furthermore, studies in *Cd68* ko mice are suggestive for CD68 to negatively regulate antigen uptake and loading onto MHC molecules [188].

We observed significant upregulation of four subunits of *Cd3* (*Cd3g*, *Cd3d*, *Cd3e*, *Cd3z*, corresponding to Cd3-gamma γ, -delta δ, -epsilon ε, -zeta ζ chains) in the lung of aged mice (Fig. 10D). Importantly, Cd3 and the εγ, εδ, ζζ subunits interact with the TCRαβ dimer to form the TCR-CD3 complex. Activation of the TCR-CD3 complex requires phosphorylation of the immunoreceptor tyrosine-based activation motif (ITAMs) [189], and this reaction is catalyzed by the lymphocyte-specific protein tyrosine kinase (Lck) which we found 2-fold increased in the lung of aged mice. The activation of the Lck kinase is mediated by protein tyrosine phosphatase receptor type C (Cd45). This phosphatase dephosphorylates the inhibitory C-terminal tail of Lck [190], and in the present study, we observed its induced expression to be strictly age-related. Lck phosphorylates the Zeta chain of T cell receptor associated protein (Zap70), and the gene coding for this kinase is significantly induced in the lung of aged mice (supplementary Table S10). Upon its activation, Zap70 phosphorylates linker for activation of T cells (Lat) and the lymphocyte cytosolic protein 2 (Slp76). Both genes were about 2-fold induced in the lung of aged mice. The coded proteins function as a scaffold for signaling molecules [191]. By now, it is well established that different antigens elicit distinct phosphorylations of the various ITAMS of the CD3 receptor [192], and although not fully understood, ITAM tyrosine phosphorylation diversity is required for optimal TCR signal transduction and subsequent T cell maturation [193].

Moreover, Cd4 and Cd8 act as coreceptors to amplify TCR signaling and their expression increased mildly but significantly (Fig. 10C). Likewise, the co-stimulatory molecules *Cd28*, *Cd80* and *Cd5* were increased in expression by about 2-fold in aged mice (Fig. 10D).

Specifically, the inducible T cell co-stimulator (Icos) is expressed on activated CD4^+^ and CD8^+^ T cells [194], but not on resting T cells, and Icos is rapidly induced after TCR and Cd28 activation [195]. Interestingly, TNFα treatment induced Icosl expression in the human lung A549 cell line [196], and we observed induced *Icos*, its ligand (*Icosl*) and *Tnfα* expression in the lung of aged mice (supplementary Table S10).

Through complex signaling events and co-stimulatory activity of Cd28 and Icos, activated T cells release several cytokines including Il2, Il4, Il10, Il17 and Tnfα [197]. While genes coding for these cytokines were mildy but significantly induced in the lung of aged mice, interferon γ remained unchanged. The mild upregulation of cytokine coding genes imply an age-related increase in inflammatory molecules but the contributions of specific immune cells to inflammaging remains uncertain. Based on single cell RNAseq data, we show an age-related induced expression of marker gene sets for interstitial macrophages, NK cell, B-cell, CD4 and CD8-T cells, and although the gene markers for neutrophils did not change (Fig. 10A2) the data derived from lung tissue studies were suggestive for an age-related increase in their expression (Fig. 10A1). Indeed, an age-related change in neutrophil trafficking and its pulmonary infiltration has been reported [198, 199]. Together, inflammaging can be regarded as a misdirected immune response and cellular senescence is a likely cause of it [200]. Aging cells are characterized by an accumulation of oxidative stress, metabolic deregulations, DNA damage and telomere shortening and all of these events trigger “alarm” signals which lead to the recruitment of immune cells [198]. Furthermore, we noted a mild (< 2-fold) but statistically significant upregulation of NLRP3/ASC/Caspase-1 [201] and therefore observed upregulation of certain components of the NLRP3 inflammasome machinery.

To independently validate the results, we interrogated single cell RNAseq data and the results are summarized in Fig. 10E. Here we compared animals aged 12 weeks to animals aged 96 weeks and assessed the expression pattern of 61 immune response genes (Fig. 10E) in dendritic, alveolar and interstitial macrophages, CD4^+^ and CD8^+^ T-cells, B-cells, NK, eosinophilic granulocytes as well as AT2 cells.

We confirmed an age-related induced expression of 44 genes and therefore validated 72% of immune response genes in single cell RNAseq data among various cells resident in lung tissue of mice. The genomic data signify upregulation of gene marker sets linked to dendritic, alveolar and interstitial macrophages, B-, CD4^+^ and CD8^+^ T and NK cells.

### Age-related changes in the expression of pulmonary cell marker genes

As detailed above, we interrogated different public databases to retrieve gene markers of pulmonary cells and searched for the consensus among them (supplementary Table S11, S12). We inferred age-related changes in the cell functions by considering differences in the expression of gene marker sets. The number of genes for a given cell type differed, and in the case of alveolar type 1 (AT1) and type 2 (AT2) cells consisted of 78 and 83 genes, respectively. For each cell type, we provide a list of marker genes in supplementary Table S12 and next to AT cells, we evaluated the enrichment score of gene marker sets for basal, ciliated, club, goblet, capillary, endothelial, fibroblast and myofibroblasts. Furthermore, we validated the expression of gene marker sets in single cell RNAseq data of AT1, AT2, ciliated, club, goblet endothelial and fibroblasts (supplementary Figure S7).

**Fig. 11:**
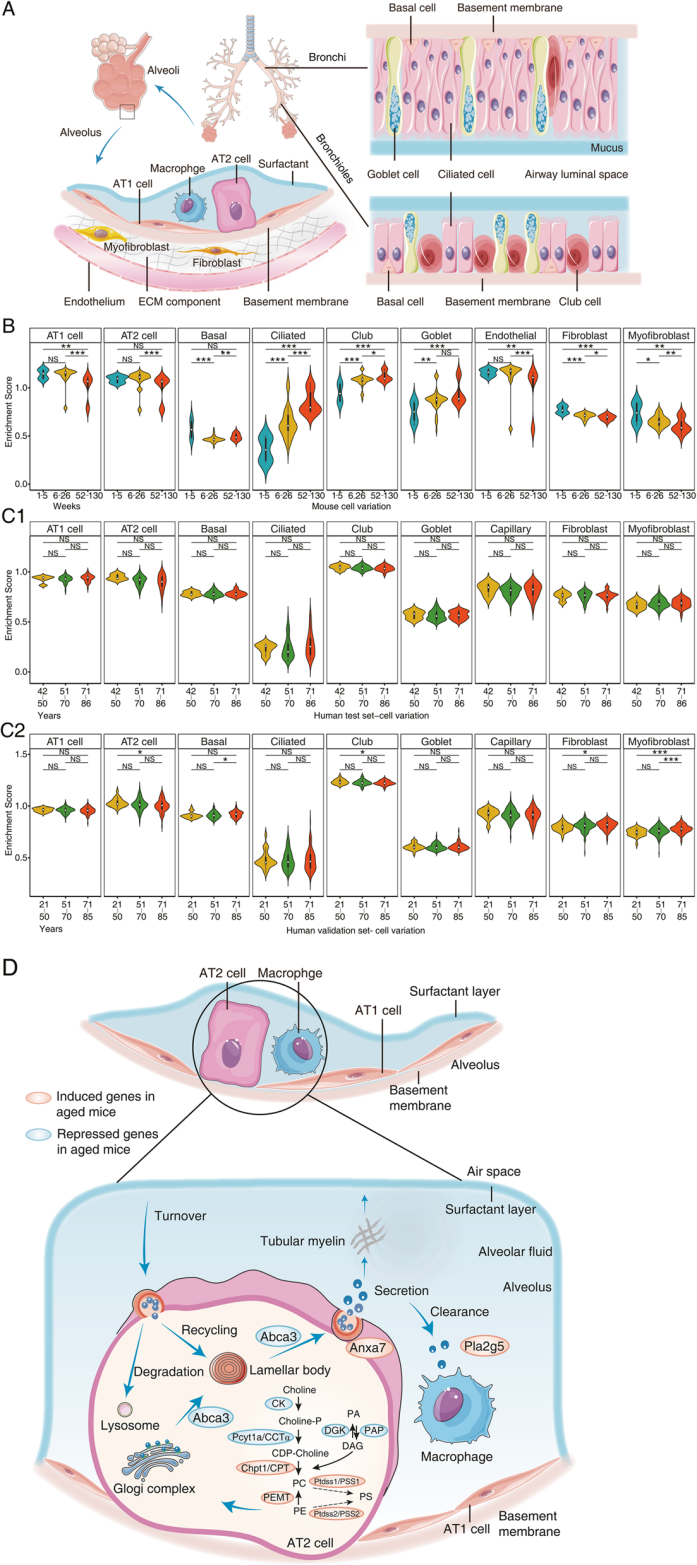
Age-dependent regulation of marker genes of pulmonary resident cells. We retrieved marker genes from various databases and list individual genes in supplementary Table S12. The data are enrichment scores based on signal intensities of individual genes and infer a change in cellular activity with age. For each cell type, we compute the enrichment score on a large set of genes (range 44–434 genes for the mouse and 147–922 genes for human pulmonary cells; supplementary Table S12). **A** Anatomical location of pulmonary cells in lung. **B** Depicted are violin plots of age regulated enrichment scores for 9 different pulmonary cells of the mouse. Panel C1: Depicted are violin plots of age regulated enrichment scores for 9 different pulmonary cells of the human test set. Panel C2: Depicted are violin plots of age regulated enrichment scores for 9 different pulmonary cells of the human validation set. Statistical significance testing: “Kruskal–Wallis” test. ns: not significant, *p < 0.05, **p < 0.01, ***p < 0.001. **D** Scheme of alveoli and the interplay of AT2 surfactant producing cells with alveolar macrophages. Alveolar type 2 cells produce surfactant. We show gene regulations in the aged lung of mice, which code for the main biosynthetic pathway of phosphatidylcholine, i.e., α-isoform of choline kinase (CK), choline-phosphate cytidylyltransferase (PCYT1A/CCTα) and choline phosphotransferase (CHPT1/CPT1). These genes code for main biosynthetic pathway of phosphatidylcholine, and the results imply an age-related change in the production and composition of surfactant phospholipids. Additionally, phosphatide phosphohydrolase (PAP) catalyzes the conversion of phosphatidic acid (PA) to diacylglycerol (DAG), and we observed its age-related down-regulation. Furthermore, diacylglycerol kinase (DGK) catalyzes the phosphorylation of diacylglycerol to PA and DGK expression is repressed. Through replacement of choline from phosphatidylcholine (PC) or the replacement of an ethanolamine group from phosphoethanolamine (PE) the enzymes phosphatidylserine synthase PSS1 and PSS2 catalyze the synthesis of phosphatidylserine (PS). Furthermore, phosphatidylethanolamine N-methyltransferase (PEMT) produces PC via methylation of PE, and in the lung of aged mice Pss1/2 and Pemt are upregulated. Finally, ATP binding cassette subfamily A member 3 (Abca3) is essential for the transport of lamellar body to the plasma membrane, and annexin A7 (Anxa7) supports the fusion with the plasma membrane and its secretion into alveolar fluid while phospholipase A2 isoform 5 (Pla2g5) hydrolyses surfactant phospholipids and stimulates the production of inflammatory lipids. In the aged lung, we found Abca3 repressed and Anxa7 and Pla2g5 upregulated

Shown in Fig. 11A are the various epithelial cells of the lung. Specifically, the airway luminal space of the bronchi is lined by pseudostratified columnar ciliated epithelium, which gradually changes from columnar to simple cuboidal and accounts for more than half of all epithelial cells in the conducting airway [202]. The luminal surfaces of the bronchus are covered by mucus of the secreting goblet cells and together with ciliated cells function as vital components of the mucociliary clearance. Although the physiological functions of the mucus gel of the bronchus and surfactant layer of alveoli differ, both are essential barriers in the defense against pathogens and airborne toxins. Furthermore, the surfactant in the alveolar space lowers surface tension, thereby preventing atelectasis as summarized in the seminal review of Han and Mallampalli [203].

Based on an assessment of marker gene sets, we observed for 89 mouse lung samples an age-related decrease in the enrichment scores for AT1 and AT2, endothelial cells, fibroblast and myofibroblast (Fig. 11B) whereas for ciliated, Club and Goblet cells the enrichment scores increased.

A major difference between AT1 and AT2 cells relates to the surfactant production, i.e. AT2 cells produce surfactant and transdifferentiate into AT1 cells, and this is associated with a change in epithelial cell morphology. Specifically, AT1 cells are flat and highly abundant and cover nearly 95% of the gas exchange surface area, whereas AT2 cells are cuboidal and to some extent possess the ability for self-renewal [204]. By interrogating single-cell RNAseq data of mice, we confirmed the regulation of AT marker genes (supplementary Figure S7). Furthermore, and unlike the human test set, we observed an age-related reduction in the AT2 and club enrichment score but an increase in enrichment scores for fibroblast, myofibroblast and basal cell when considering the human validation set of 307 individual samples (Fig. 11C2).

In regards to the regulation of AT2 cell marker genes in the mouse lung, we wish to highlight the 2- and 1.5-fold repressed expression of the ERBB receptor feedback inhibitor 1 (*Errfi1*) and the suppressor of cytokine signaling 2 (*Socs2*). Errfi1 is the feedback inhibitor of the EGFR signaling, and its repression supports EGFR dependent cell proliferation [205]. Conversely, Socs2 function as an antiinflammatory mediator and its repression supports inflammaging [206]. Furthermore, a recent study demonstrated the importance of cellular senescence of AT2 cells in pulmonary fibrosis [207]. We observed a significant but mild down-regulation of activated leukocyte cell-adhesion molecule (*Alcam*) in the lung of age mice, and its expression is repressed in experimental models of lung fibrosis and in clinical samples of idiopathic fibrosis (IPF). Moreover, gene silencing of Alcam is associated with increased cell death [208].

A further example relates to the transcriptional repressor SIN3 transcription regulator family member A (Sin3a) which was 1.5-fold repressed in the lung of aged mice. In the human lung, its repression did not reach statistical significance. Note, through genetic studies and single cell RNA sequencing, Yao and colleagues defined a key role for Sin3a in cellular senescence, and the loss of *Sin3a* leads to a drastic increase in cell size, a marked reduction in colony-forming efficiency of lung cells and an almost complete loss of cell proliferation capacity [207].

Additionally, the enrichment score for marker genes of basal cells declined, and these cells are attached to the basement membrane. The basal cell function as progenitor cells in the respiratory epithelium and are able to replace damaged cells following injury. In the lung of aged mice, we noticed predominantly repression of basal cell marker genes of which the sestrins are a remarkable example. These stress-inducible proteins are protective against oxidative stress and airway remodeling, and we observed sestrin 1–3 to be repressed less than 2-fold in the lung of aged mice. The functions and roles of sestrins in regulating human diseases have been the subject of a recent review [161] which included a description of their role in the support of stem cell homeostasis [209]. Furthermore, we observed significant upregulation of the stem cell marker leucine rich repeat containing G protein-coupled receptor 5 (*Lgr5*). Specifically, Lgr5 expression level increased during injury [210] and its expression is restricted to a subpopulation of basal cells with progenitor/stem cell properties [211].

Conversely, the marker gene set for ciliated cells increased when 6–26 week old mice were compared to 1–5 week old ones, and based on ciliated single cell RNA-seq data, we confirmed this change in the enrichment score between 12 and 96 week old mice (supplementary Figure S7). Our finding are in agreement with the results of Ilias and colleagues [5]. However, in the aged human lung, the enrichment score for ciliated cells did not change.

For club cells, we determined an age-related increase in the enrichment score and confirmed this change in single club cell RNA-seq data. Here, we compared 12 week old mice with aged ones (supplementary Figure S7). Note, club cells are thought to be progenitor/stem cell like cells which under condition of stress transdifferentiate into goblet and ciliated cells. In fact, there are several stem cell niches in the lung such as the basal and secretory stem cells in the large airways and the AT2 cells of the alveoli.

Furthermore the enrichment scores for marker gene sets of goblet cells increased in a strict age-related manner (Fig. 11B), and among the goblet marker genes, we found mucin 16 and 20 to be significantly upregulated which represent a major airway mucin [212]. In the human lung, however, the enrichment score for gene markers of the goblet cells did not differ. Furthermore, for the human validation set (Fig. 11C2) but not the test set (Fig. 11C1) we computed a significant decline of the enrichment score for club cells. It is tempting to speculate that such a decline in the enrichment score is linked to an age-related decline in the ability to clear mucus from the lung [212].

Likewise we observed a strict age-related decline in the enrichment scores for fibroblasts and myofibroblasts (Fig. 11B). Specifically, the gene marker set of fibroblasts consisted of 434 genes of which 33 declined in expression with age. Among them are basement membrane coding ones (e.g., *Lama4*, *Lamb1*, *Nid1*) and the ones coding for ECM degradation (*Mmp2* and *Adamts2*, see Fig. 7G). Strikingly, the transcript expression of tenascin C was highly repressed (> 3-fold) in the lung of aged mice, and this gene codes for a glycoprotein of the ECM (Fig. 7G) and is of critical importance in wound healing. A further example relates to slit guidance ligand 2 whose expression declined with age. This protein inhibits fibroblast differentiation and fibrosis [213], and its expression was drastically reduced to 15% when compared to young animals. For its protective role in bleomycin induced fibrosis, we considered the age-dependent repression of slit guidance ligand 2 as detrimental. Conversely, the age-dependent repression of follistatin-like 1 (1.5-fold) and cadherin 11 (1.5-fold) can be regarded as beneficial, given their role in the promotion of fibrosis [214, 215]. Notwithstanding, we could not confirm a decline in the enrichment score for fibroblasts based on single cell RNAseq data of 12 week and 96 week old mice (supplementary Figure S7).

### Lung surfactant coding genes in the aging lung

The surfactant of the alveolar space defines the pulmonary air–liquid interface, and is composed of phosphatidylcholine (PC ~ 80%), phosphatidylglycerol (~ 10%), minor phospholipids, i.e. phosphatidylserine (PS), cholesterol (~ 10%) and the surfactant proteins (SP-A to SP-D) [216, 217]. Its main function is to reduce surface tension thereby preventing atelectasis [203]. For the human and mouse lung, an alveolar surface of approximately 40–80 m^2^ and 80 cm^2^ has been determined [218]. Alveolar type 2 cells produce surfactant and lung surfactant represents an important barrier for airborne pathogens and toxins. Depicted in Fig. 11D are the various aspects of surfactant biology, i.e. the transport of lamellar body by the ATP binding cassette subfamily A member 3 (ABCA3) transporter to the plasma membrane, the fusion of the lamellar body with the plasma membrane and its secretion into alveolar fluid, its incorporation into the surfactant layer, the recycling of used surfactants, and finally, its degradation by lysosomes and alveolar macrophages.

We already emphasized (see above) the role of senescence of alveolar epithelium in the aging lung and now highlight some key steps in the production of surfactant. First, we considered age-related changes in the regulation of genes coding for the synthesis of phospholipids. Specifically, in the lung of aged mice, we observed about 2-fold repression of the α-isoform of choline kinase (CK) and choline-phosphate cytidylyltransferase (PCYT1A/CCTα) whereas choline phosphotransferase (CHPT1) and choline/ethanolamine phosphotransferase 1 (CEPT1) increased by 2.4 and 2-fold, respectively. These genes code for main biosynthetic pathway of phosphatidylcholine, and the results imply an age-related change in the production and composition of surfactant phospholipids. Other investigators also reported compositional changes of the surfactant with age [56, 219]. Unlike mice, the aforementioned genes were not regulated in the human lungs of our study cohort.

Additionally, in the lung of aged mice, we noticed repressed transcript expression of the phospholipid phosphatases 1 (*Plpp1*), whereas *Plpp3*, *Plpp2*, lipin 1 (*Lpin1*) and *Lpin2* were about 2-fold upregulated. The proteins function as phosphatide phosphohydrolase (PAP) and catalyze the reaction phosphatidic acid (PA) to diacylglycerol (DAG) [220]. Conversely, in the aged human lung, transcript expression of phospholipid phosphatase 2 increased by 1.7-fold and the enzyme converts phosphatidic acid (PA) to diacylglycerol.

As shown in Fig. 11D diacylglycerol kinase (DGK) catalyzes the phosphorylation of diacyl glycerol to phosphatidic acid (PA), and there are 10 isoforms of DGK which are grouped into 5 types. Both DGK and PA are important signaling molecules and function in various physiological pathways including immune response [221]. In the lung of aged mice, we observed 2-fold repressed expression of the type 2 kinases DGKδ and DGKη. Conversely, in the aged human lung, expression of Dgkd and Dgkq increased by about 2-fold, and these kinases play a role in T cell function and TCR response (see above) [222].

Phosphatidylserine (PS) is another component of the surfactant, and we observed small but significant increases in transcript expression of phosphatidylserine synthase (*Pss*)*-1* and *Pss2* in the lung of aged mice (Fig. 11D). The coded proteins catalyze the synthesis of PS from phosphatidylcholine or phosphatidyl-ethanolamine, and we observed an approx. 1.5-fold upregulation of phosphatidylethanolamine N-methyltransferase (*Pemt*). This enzyme catalyzes the formation of PC via methylation of phosphatidyl-ethanolamine. Although independent research confirmed the expression of this gene in the lung [223], it is uncertain whether the coded protein is functionally active in airway epithelial cells. Notwithstanding, the enzyme is abundantly expressed and highly active in the liver [224]. Furthermore, the CDP-choline pathway yields PC, and we observed a small but significant down-regulation of *Pcyt1a*. The coded protein functions as a CDP-choline:1,2-diacylglycerol cholinephosphotransferase in the production of PC (Fig. 11D). Noteworthy, ethanolamine kinase (ETNK1) catalyzes the first step in the production of phosphoethanolamine (PE) from ethanolamine, and PE can be methylated by PEMT to support PC production [224]. Unlike in humans, *Etnk1* transcript expression was significantly repressed by 2.2-fold, and this implies repressed PE production in the lung of old mice. However, other key enzymes of this pathway, i.e. *Pcyt2* and the choline/ethanolamine phosphotransferase 1 (*Cept1*) increased by 2-fold. Interestingly, some reports suggest phosphatidylethanolamine to positively regulate autophagy and longevity [225, 226].

The significant down-regulation of the *Abca3* gene in the lung of aged mice is another important finding. This lipid transporter of alveolar type 2 cells is of critical importance for the intracellular transport of lamellar bodies to the plasma membrane and the subsequent exocytosis of surfactant (Fig. 11D). Owing to its structural complexity, the Abca3 transporter is recycled or degraded in lysosomes, and there are multiple reports on human disease causing mutations of the *Abca3* gene [227, 228]. These are linked to various lung diseases, especially respiratory failure in term neonates, childhood interstitial lung disease (chILD), idiopathic pulmonary fibrosis (IPF) and diffuse parenchymal lung disease (DPLD) of adults [227].

Noteworthy, annexin A7 promotes membrane fusion between lamellar bodies and plasma membranes [229, 230], and this supports surfactant exocytosis of AT2 cells. We found 2-fold induced *Anxa7* expression in the lung of aged mice, and it has been reported that surfactant phosphatidylcholine secretion can be augmented in Anxa7-deficient AT2 cells through exogenous application of Anxa7 [230]. Together, the data implies impaired surfactant exocytosis in the lung of aged mice.

*ANXA13* is induced in the lungs of aged humans; however, the specific functions of ANXA13 in the lung have not been elucidated as yet.

In the lung of aged mice, we observed a significant 2.4-fold induced expression of phospholipase A2 isoform 5 (*Pla2g5)*. This enzyme plays a critical role in surfactant degradation and eicosanoid generation. Its induced expression aggravates lung injury in aged mice due to increased surfactant hydrolysis. However, eicosanoids are also key players in the inflammatory response and in the regulation of vascular tone [231]. Indeed, Pla2g5 participates in the antigen processing in macrophages and dendritic cells, and in *Pla2g5* knockout mice, macrophages are defective for phagocytosis [232] and antigen processing [232, 233]. Moreover, Pla2g5 expression in interstitial macrophages is augmented by IL4 [232] and in the present study, *Il4* expression was significantly induced in the lung of aged mice.

Finally, independent studies on the molecular composition of the alveolar lining fluid evidenced its enrichment with pro-inflammatory cytokines, modification of surfactant proteins and lipids and complement components in the lungs of aged mice and humans [234].

### The effects of tobacco smoke on age-related gene expression changes in the lung

We performed whole genome analysis of 52 control cases (*N* = 45 smokers and *N* = 7 never smokers) with definite information on tobacco use to address the question, whether smoking affected the age-related gene expression changes as disovered in the present investigation. Importantly, none of the age-related gene regulations in the human lung are influenced by tobacco use, and the data are given in supplementary Figure S8. We were astonished by the result as we expected tobacco product consumption to influence the aging process. However, we identified highly regulated genes in tobacco product users, and this included the xenobiotic defense gene cytochrome P450 family 1 subfamily A member 1 (*CYP1A1*) and the aryl hydrocarbon receptor repressor which were 42- and 5-fold induced, respectively in smokers. Likewise, we observed 10-fold induced expression of selectin E which allows adhesion of leucocytes at sites of inflammation [235]. Moreover, we observed a 20-fold induced expression of LIM homeobox domain 9, i.e. a putative tumor suppressor which was highly responsive to tobacco smoke exposure and determined 3-fold induced expression of epidermal growth factor to stimulate EGFR signaling. Further examples included the 6-fold induced expression of *MT1G* and this metallothionin inhibits ferroptosis [236]. Additionally, we noted 6-fold induced expression of *SLC4A1* and this solute carrier is abundantly expressed in erythrocytes and function in the transport of carbon dioxide from lung tissue [237]. Importantly, the concentration of CO2 in mainstream cigarette smoke is about 200-times higher when compared to the atmosphere [238]. Furthermore, tobacco use caused a > 7-fold increased expression of 5'-aminolevulinate synthase 2 and this erythrocyte-specific mitochondrial enzyme catalyzes the first step in the heme biosynthetic pathway, however decreased > 20-fold in the aged human lung. Given that tobacco smoke contains CO which harms erythrocytes and COHb concentrations may increase from 1 to 10% COHb or above among smokers, we regard the upregulation of 5'-aminolevulinate synthase 2 as an adaptive response to CO-harmed erythrocytes. Together, all these highly induced gene expression changes are logical and are caused by tobacco product use. We also noted a 8-fold induced expression of the G protein-coupled receptor 15. This GPCR may counteract inflammation following exposure to tobacco smoke [239, 240]. Finally, we wish to highlight the opposite regulation of collagens *COL10A1*, *COL11A1* and matrix metalloproteinase *MMP11* in control cases with a history of tobacco use. Note, for the aged human lung, we found *COL10A1* and *MMP11* mildly but significantly upregulated; however, tobacco smoke exposure nearly silenced their expression to about 10% of the non-smoking controls.

### Common gene regulations in the aged human and mouse lung

Initially, we performed comparative genomics to search for commonalities between the human test and validation set. Although the test and validation set identified 237 and 1673 DEGs, only 26 genes were regulated in common. Among the common DEGs, 14 code for ECM and the remaining 6 genes code for immune response. We already emphasized the importance of ECM remodeling in the aged lung; however, were surprised to see only a small number of common gene regulations between the two data sets. Our finding underscores the significant variability of genomic data in a cohort of 107 (test set) and 307 (validation set) individuals. Obviously, the genomic data is greatly affected by life style, nutrition, co-morbidities, and tobacco product use. As described above, we compared the genomic data of normal lung tissue of never-smokers with smokers, and although histopathology confirmed the lung tissues to be normal, we observed 20 highly regulated genes following tobacco smoke exposure. However, tobacco use did not influence age-dependent gene regulations as reported herein.

Subsequently, we analyzed the mouse and human pulmonary genomic data and compared DEGs from the lung of aged mice to the aged human lung. Strikingly, only two genes are commonly regulated, i.e. *Aebp1* and *Col9a2*. However, when DEGs of the human test and validation sets were combined, we obtained 86 DEGs (59 up and 27 down) commonly regulated between humans and mice. Moreover, 11% and 25% of up- and down-regulated DEGs differed with a FC > 2. Supplementary Table 14 compiles the commonly regulated genes, and the majority code for ECM remodeling, senescence, immune response and regulation of airway epithelium.

Examples of commonly upregulated genes between the mouse and human lung include collagens (*COL10A1*, *COL15A1*), MMP (*MMP16*), glycoproteins (thrombospondin 2) and proteoglycan (aggrecan, proteoglycan 4). For instance, expression of Thy-1 cell surface antigen (*THY1*) increased with age and THY1 stimulates fibroblast apoptosis and lung injury resolution [241] while protein tyrosine phosphatase receptor type T (PTPRT) contributes to lung stiffness.

Lastly, we observed opposite regulations of *Alas2*, *Slc4a1* and orosomucoid 1 between mice and humans. The aforementioned genes were upregulated in the lung of aged mice but down-regulated in the aged human lung. Conversely, calsyntenin 2, a protein so far only described for its function in the morphology of synaptic complexes in mice was repressed in expression in the lung of aged mice but upregulated in the human lung and may possibly related to synaptic changes in sensory nerves of the aged lung.

## Discussion

This study aimed to investigate age-related changes in the pulmonary genomes of mice and humans, and the gene set enrichment analysis of DEGs informed on various biological pathways which changed over time. We focused on four major biological processes, namely extracellular matrix remodeling, cellular senescence, immune response and surfactant biology and considered a wide range of data including single cell RNA sequencing of pulmonary cells. Furthermore, we probed for the concordance between the gene and coded protein data and searched for continuous gene expression changes over time.

### ECM remodeling in the aged lung

An important finding of our study is the age-dependent change in the expression of ECM coding genes, and for the mouse lung, a complex picture emerged with the regulation of 28 collagens, 10 proteoglycans, 17 matrix metalloproteinase and 62 ECM-related glycoproteins. The majority of continuously upregulated genes coded for metalloproteinases, especially *Mmp8*, *9* and *12* (Fig. 7F). These function in the degradation of macrophage inflammatory protein 1α to reduce lung inflammation (Mmp8) [242], cytokines and matrix-bound growth factors (Mmp9) [243] and are of critical importance in the onset and progression of lung emphysema (Mmp12) [244]. A recent study discovered the critical role of the endolysosomal cation channel mucolipin 3 (Trpml3) in controling Mmp12 reuptake [245]. Note Trpml3 is predominantly expressed in alveolar macrophages and Trpml3 is a critical regulator of Mmp12 clearance. We observed 2-fold induced expression of *Trpml3* and *Mmp12* in the aged lung of mice; however, *Timp1*, an inhibitor of Mmp12 was only marginally upregulated. Furthermore, in the human lung *MMP12* was nearly 2.2-fold repressed while its inhibitor *TIMP1* was induced. Given that *Mmp12* ko mice do not develop emphysema [246] we consider the marked repression of *Mmp12* as an age-related adaptive response to a lifetime exposure of pathogens and pollutants. Moreover, our study is suggestive for species dependent clearance of MMP12 via TRMPL3 or TIMP1. In fact, TRPML3 is not regulated in the human lung but in mice. Another example relates to the upregulation of *Timp3*, and the repressed expression of its targets *Mmp2* and *Mmp14* (Fig. 7G) underscores imbalances in the ECM homeostasis of the aging lung.

Additionally, we noticed upregulation of collagen type I and III in the aged human lung, and these are produced by activated fibroblasts which results in a more rigid fiber network and are part of a wound repair process. Interestingly, Mays et al. reported an age-related increase in the concentration and relative proportions of types I and III collagen in the lung of rats [86], and therefore similar changes occur in the human and rodent lung. Furthermore, we noticed a V-shaped expression pattern of collagen type II expression in neonatal and aged lungs. Importantly, in the aged human lung expression of collagen triple helix repeat containing 1 (*CTHRC1*) increased significantly, and a recently published atlas of collagen-producing cells of the human lung identified *Cthrc1*-expressing fibroblasts as a unique subpopulation in fibrotic lungs [95]. Based on histopathology, the lung specimens studied by us were classified as healthy. Nonetheless, our findings imply an age-related increase of a subset of fibroblasts which contribute to stiffness and possibly age-related mild fibrotic changes of the lung. Obviously, the continuous exposure to pollutants is a major reason for “inflammaging” and leads to an activation of fibroblasts. The question of how collagen becomes stiff has been the subject of a recent editorial [247]; HIF pathway activation leads to dysregulated collagen structure–function in the human lung [248].

We observed repressed expression of basement membrane coding genes, notably laminins (*Lama4*, *Lamb1*, *Lamc1*), nidogens (*Nid1*, *Nid2*), tenascin C (*Tnc*) and type IV & VI collagens in the lung of aged mice. These non-collagenous proteins function as linker in the ECM network while laminins form the basal lamina of basement membranes which are part of the mechanical scaffold. Our findings agree with the data reported by others [5, 28].

Strikingly, transcript expression of elastin (Eln), i.e. a fiber essential for pulmonary compliance declined by nearly 90%, and independent research confirmed a similar reduced elastin protein content with age [28, 54]. Owing to its function, a reduced elastin content results in thinner and fragile alveolar septa [55] to possibly effect the development of emphysema [58]. Additionally, there is evidence for Tgfβ to influence elastin transcription via phosphatidylinositol 3-kinase/Akt activity [121]. In the present study, we observed 2-fold repressed expression of the PI3K subunit α in adult and aged mice. Conceivably, this provides a rationale for the sharp decline in elastin expression between neonatal and adult mice (Fig. 7H).

We also identified an age-related up to 4.5-fold increased expression of the fibrinogen alpha (*Fga*) and gamma chain (*Fgg*). Note, enhanced fibrinogen deposition changes the extracellular matrix to support cell migration during matrix remodeling and tissue repair [249].

Lastly, we highlight the regulation of proteoglycans. These are composed of polysaccharide chains attached to core proteins [250] and when combined with collagens and elastin, the collagen-elastin network gets stabilized [251]. Proteoglycans participate in inflammatory and angiogenesis processes, and we identified 10 genes as significantly regulated in the lungs of aged mice [252–254]. Examples are fibromodulin and versican, and their induced expression negatively influences the elastic recoil [255]. Moreover, versican binds CC-chemokines [253] and promotes leukocyte migration [256]. Conversely, an age-related induced expression of decorin protects against fribrotic scares by inhibiting TGFβ cytokine signaling [69, 70].

### Cellular- and immunosenescence

Cellular senescence is characterized by a stable cell cycle arrest [124] and an activated senescence-associated secretory phenotype (SASP) [130]. The SASP comprises a range of inflammatory molecules and causes an aged related decline in the anatomical and physiological functions of the lung [257]. Moreover, immunosenescence is a key mediator for susceptibility to infection [258]. Altogether, we considered 71 SASP coding genes of which 53 were significantly regulated. For instance, Ccl8, Ccl13, Ccl20 function as chemoattractants and recruit monocytes to sites of injury. Similarly, regulation of matrix metalloproteinases supports an extravasation and migration of inflammatory cells [81, 146]. It is of considerable importance that all of the senescence associated gene regulations were independently confirmed by other studies with some genes such as *Cxcl13* were > 9-fold induced in the lungs of aged mice [5].

Senescence results in pathophysiological changes of the microenvironment, and there is growing evidence for a central role of p53 in directing cellular senescence programs [259]. Indeed, a lifetime exposure to airborne particulate matter and pollutants represents a source of chronic stress, and ROS induced cellular stress stimulates p53 activity and the expression of antioxidant defense genes. Through a complex interplay of factors, p53 either supports repair or triggers programmed cell death but also stimulates cellular senescence. We observed an age-related upregulation of antioxidant defensive genes as exemplified by the 2-fold induced expression of nuclear factor erythroid 2 related factor 2, i.e. a regulator of redox homeostasis; however, repressed NADPH oxidase 4. Similarly, expression of *p53* and Sirt1 which deacetylates p53, was significantly induced, and all this suggests age-related responses to oxidative stress. Intriguingly, the p53 inducible nuclear protein (*Trp53inp1*) was significantly repressed, and this protein acts as a positive regulator of autophagy [157, 158]. Furthermore, we observed autophagy-related ubiquitin-like modifier LC3B significantly repressed in the lungs of aged mice and therefore infer an inadequate autophagy in aged lungs which has also been the subject of a recent review [159].

Importantly, the telomere size is a critical factor in cellular senescence and genomic stability. Its shortening will lead to replicative senescence, although this paradigm has been challenged [170]. Telomere dysfunction leads to DNA damage response (DDR), and cellular aging [171]. We observed upregulation of telomerase reverse transcriptase, i.e. a rate-limiting subunit of telomerase in the aged lung of mice, and TERT stabilizes telomeric DNA. Therefore, we regard its upregulation as an adaptive response to counteract replicative senescence.

Lastly, immunosenescence is defined by an age-related change in the immune response, and based on immune cell gene marker sets, we detected significant changes of the genes coding for an innate and adaptive immune system. We observed an age-related increase in the expression of gene marker in the lung of aged mice which hallmark alveolar and interstitial macrophages, B-cell and dendritic cells, neutrophils and cytotoxic CD4^+^ and CD8^+^ T- cells (Fig. 10A1). With the exception of alveolar macrophages we confirmed the results by single cell RNAseq (Fig. 10A2). In stark contrast, and with the exception of the gene markers of alveolar macrophages of the test set (Fig. 10B1), none of the cell marker gene sets significantly changed in the aged human lung. However, with the larger human lung validation set of 307 individuals, gene markers for CD4^+^ and CD8^+^ cells were significanlty upregulated, and the results are comparable to the aging lung of mice.

Together, we observed species specific differences in immunosenescence, and Fig. 10C highlights the regulation of individual genes among different immune cells of the mouse. Based on single cell RNAseq similar age-related changes in the expression of immune cell marker genes have been reported [5]. Overall, we observed 31 immune genes whose expression increased continuously with age, and these code for immune cells recruitment, their activation, migration, adhesion and proinflammatory cytokine secretion. For instance, we observed about 4-fold induced expression of *Il7r* and *Ccl5*, and the coded proteins function in B and T cell maturation [184], neutrophil migration and alveolar macrophage activation [185]. Strikingly, the genes coding for the immunoglobulin heavy constant mu, *Igha* and *Ighg* were > 20-fold induced in the lung of aged mice, and we observed upregulation of 20 genes coding for the TCR-CD3 complex. Clearly, this emphasizes its role in inflammaging.

### Age-related changes in surfactant coding genes

The surfactant defines the pulmonary air–liquid interface and is composed of phospholipids, cholesterol and surfactant proteins. AT2 cells are of critical importance for the production of surfactant, and its main function is to reduce surface tension [203]. Moreover, it is an important barrier for airborne pathogens and toxins.

Among age-related changes, we noted repressed expression of the α-isoform of choline kinase (CK) and choline-phosphate cytidylyltransferase (PCYT1A/CCTα) whereas choline phosphotransferase (CHPT1) was upregulated (Fig. 11D). These code for the main biosynthetic pathway of phosphatidylcholine, and the data imply changes in the production and composition of surfactant phospholipids. Likewise, independent studies reported compositional changes of the surfactant with age [56, 219].

We considered the various aspects of surfactant biology and observed repressed expression of *Abca3* but upregulation of annexin VII. These function in the transport of lamellar body to the plasma membrane, support the fusion with the plasma membrane and surfactant secretion into alveolar fluid. Next to alveolar macrophages, AT1 cells are capable to recycle surfactant and degradate lipids in lysosomes. We observed induced expression of phospholipase A2 isoform 5 which directly hydrolyzes surfactant phospholipids and stimulates the production of inflammatory lipids. Additionally, the molecular composition of the alveolar lining fluid was the subject of independent reports, and there is evidence for its enrichment with pro-inflammatory cytokines, modification of surfactant proteins and lipids and complement components in the lung of aged mice and humans to possibly modify immune responses [234].

Note the luminal surfaces of the bronchus is covered by mucus which is synthesized by goblet cells and together with ciliated cells function as vital components of the mucociliary clearance. Although the physiological functions of the mucus gel of the bronchus differs from the surfactant layer of alveoli, both are essential barriers in the defense against pathogens and airborne toxins. In the present study, we observed an age-related increased expression of mucin 16 and 20 which are major airway mucin [212].

### Variations in the expression of cellular marker genes with age

As shown in Fig. 11B, the enrichment score for gene marker sets of various lung cells changed with age. Specifically, we observed age-related increases in the enrichment score for several lung cells. Considering the nature of the enrichment score, its overrepresentation in the lung of aged mice imply increased expression of gene marker sets with age. Importantly, each cell type is characterized by a large set of genes, and in the case of AT1 and AT2 cells, we considered 78 and 80 unique genes, respectively (supplementary Table S12). We observed a decreased enrichment score of AT1 and AT2 cells, and the findings underscore impaired regenerative capacity with age even though some key regulators of lung homeostasis were unchanged, particularly the transcriptional co-activators YAP or TAZ. Although the gene expression of the Tead1 transcription factor was significantly repressed (2-fold), the expression of other TEAD coding genes was unchanged. Given the establish role of TEAD in the control of AT1 specific gene expression [260], we performed in silico genomic footprinting, and found AT1 and AT2 marker genes to be nearly 3-fold enriched for TEAD binding sites (*p* < 0.05). Importantly, independent research confirmed AT2 cells not to decline in number with age, yet its differentiation into AT1 cells and the overall density might be reduced [261].

On the other hand, the enrichment scores for ciliated airway epithelium and club cells increased, and we confirmed the results by single cell RNA-seq. Here, we considered 143 and 31 unique genes, respectively to determine the enrichment score (supplementary Table S12). Importantly, club cells, especially of the secretoglobin family 1A member 1 (*Scgb1a1*)^+^ lineage, are thought to be progenitor/stem cell like cells, which under conditions of stress, transdifferentiate into goblet, ciliated and alveolar epithelial cells as well as basal cells [22]. The Scgb1a1 protein (formerly named club secretory protein 16) is secreted by club cells and confer protection against oxidative stress and inflammation [262, 263]. We observed an induced *SCGB1A1* expression in the aged human lung and consider this upregulation as an adaptive response to redox stress and inflammaging. Redox stress is common to tobacco product use. Therefore, we and others compared the expression of *SCGB1A1* in smokers and non-smokers. There is clear evidence for tobacco use to cause its repression [264], and in a cross-sectional study, decreased SCGB1A1 serum levels were associated with tobacco smoke induced chronic obstructive pulmonary disease [265] while smoking cessation for 3, 6 and 9 months restored SCGB1A1 level in BAL fluid [266].

Given that the GATA binding protein 6 declined with age, we assumed an Wnt dependent airway modulation in the lung of aged mice. Specifically, Zhang and colleagues demonstrated the fundamental role of Gata6-regulated Wnt signaling molecules in epithelial stem cell development and lung regeneration [267], and its repression likely contributed to impaired lung epithelial cell regeneration with age.

An important feature of basal cells is their ability to replace damaged cells. However, in the lung of aged mice, several basal cell marker genes were down-regulated, and this agrees with reports by others as summarized in a recent review [200]. For instance, we found sestrin 1–3 to be repressed in the lung of aged mice, and sestrins are stress-inducible proteins and protect against oxidative stress, airway remodeling and function in stem cell homeostasis [209]. Notwithstanding, basal cell self-renewal also depends on the ROS-Nrf2-Notch1 axis [268, 269], and in the lung of aged mice *Nrf2* is upregulated; however, *Notch1* is repressed. Our findings of an opposite regulation of cell marker genes imply an age-related deregulation of basal cell homeostasis, and previous investigations demonstrated a decline in basal cell number an function in the lungs of aged mice [270, 271].

### Study limitations

There are important caveats to our study we would like to highlight.

First, we report findings with and without animals of the age of one week, and although significant changes in the gene expression pattern are obvious, these animals are sexually immature and such young mice may not be appropriate in an aging study. On the other hand the lack of consensus on an aging biology paradigm among leaders of the field, and the marked disagreement on whether or not we know what biological aging means, show that many open questions remain [272]. In fact, some recent studies argue that aging starts very early in life [273].

Second, for the human lung only 26 genes were regulated in common between the test and validation set, and the variability of individual data prevented an identification of a larger set of significantly regulated genes eventhough we considered 414 individual human lung genomic data sets. Correspondingly, we combined significantly regulated genes of both human data sets to compare the results to mice. Third, the healthy lung tissue stems from patients undergoing surgery primarily for cancer indications, and although pathology confirmed the tissue to be normal (with the exception of slight to moderate emphysema) the tumor adjacent biopsies are potentially confounded by the underlying disease. Fourth, the platforms used differed between the human test and validation set (microarray versus RNAseq). Therefore, we compared the DEGs based on fold changes rather than signal intensities. Fifth, changes in the expression of marker genes are likely measures of endogenous activity and therefore may not reflect cellular abundance.

#### Concluding remarks

**Fig. 12:**
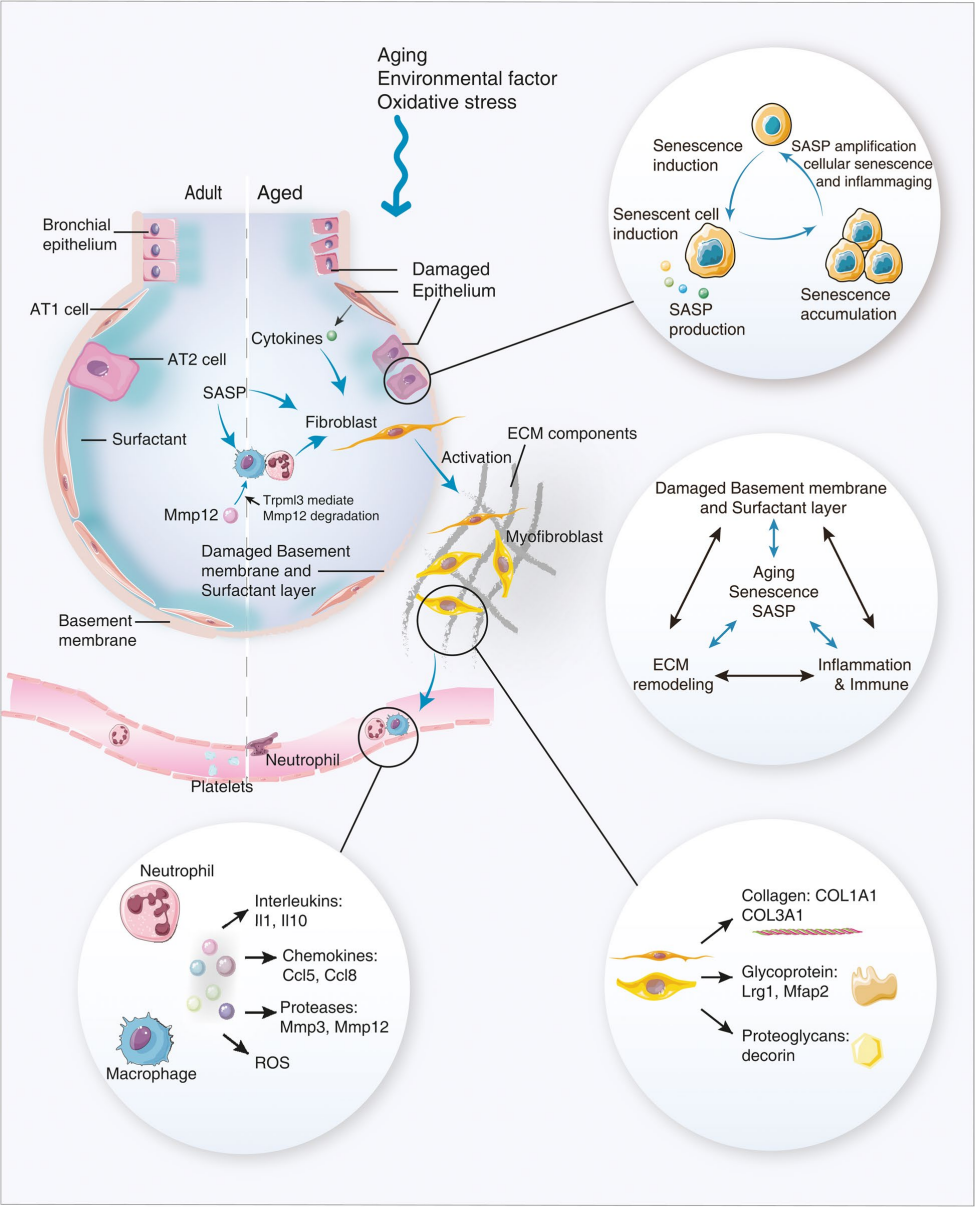
The aging lung with focus on ECM remodeling, immune response, senescence and pulmonary surfactant. The scheme highlights the complex interplay of ECM remodeling in healthy but aged lung and its link to immune response, senescence and surfactant lipids. The genomic landscape of the aging lung revealed extensive ECM remodeling which provided a rationale for an increased stiffness of the lung and damage of the basal membrane therefore contributing to an age-related emphysema. Additionally, we noticed an increased expression of genes coding for the senescence-associated secretory phenotype (SASP), thus augmenting inflammaging as well as immunosenescence. Finally, the genomic data of the aging lung indicated major changes in the production of pulmonary surfactant

Depicted in Fig. 12 is the complex interplay of ECM remodeling in healthy but aged individuals and its links to inflammaging, senescence and surfactant lipids. Obviously lifetime exposures to airborne pollutants, fine particles and pathogens has its toll on the mechanical properties of the lung. Yet, the cells themselves do no change mechanical properties [274]. Rather an age-related increase in the interstitial ECM matrix and its remodeling can be regarded as a primary cause for stiffness of the lung. In support of this notion we observed an increase in the enrichment score of fibroblasts and myofibroblasts in the human lung (Fig. 11C) but not in the aged mouse lung and these cells produce the ECM matrix. Our findings of an age-related increase in ECM deposition agrees with independent research reports [28–30], and the reviews listed herein are a valuable resource of ongoing and published research [32, 275–277].

Together there are 117 and 68 ECM mouse and human regulated genes of which two-thirds have been confirmed by independent research (supplementary Table S10). Likewise, we identified 73 and 31 significantly regulated genes coding for senescence in mice and humans, of which 47 and 17 have been confirmed by others (supplementary Table S10). Furthermore, we identified 41 immune cell marker genes in mice whose age-dependent regulations have been confirmed by others [5, 26, 27].

Finally, our study revealed a number of, as yet, unknown age-related gene regulations. For instance, we observed α‐2 glycoprotein and galectin 3 as significantly upregulated in mouse lung tissue and the coded proteins instruct ECM remodeling. A further example relates to the regulation of adipocyte enhancer-binding protein 1, and we highlighted its role in wound healing, ECM remodeling and myofibroblast differentiation. We observed regulation of collagen triple helix repeat containing 1, i.e. a gene marker specific of a fibroblast subpopulation. Noteworthy is also an age-dependent regulation of grainyhead like transcription factor 2 and paired box 8. These transcription factors activate the telomerase reverse transcriptase promoter and therefore revert the shortening of telomeres.

Additionally, we observed significant regulations of 20 genes coding for the TCR-CD3 complex. However, this complex was unchanged in the aged human lung. Notwithstanding, we identified 12 genes coding for immune response in the aged human lung and confirmed 7 in an independent RNAseq data set which was recently published [5]. For example, we observed an age-related increase in the expression of complement factor H. Owing to its function, we view its regulation as an adaptive response to inflammaging. A further example relates to an upregulation of endothelial cell‑specific protein plasmalemma vesicle‑associated protein (*PLVAP*), which allows for basal cell permeability, leukocyte migration and angiogenesis. This finding is specific to the aged human lung. Other inflammaging gene regulation included phosphodiesterase type 2A and thrombospondin 2, and their age-related upregulation has not been reported sofar.

Noteworthy is also the down-regulation of *Abca3*, which is a major surfactant transporter in AT2 cells. Furthermore, the predominant repression of genes coding for surfactant provides a clue for age-dependent changes in surfactant biology.

We also identified 77 and 13 genes, respectively in the mouse lung which were either continuously increased or repressed in expression with age, and these code for various biological processes such as ECM, inflammation and cell adhesion while 42 genes displayed a V-shaped expression pattern, i.e. high expression in neonates, down-regulated in young adult mice and upregulated in aged mice, and most are coding for ECM (Fig. 7).

## Methods

Depicted in Fig. 2 is the workflow and data analysis for the mouse and human pulmonary genomes and single cell RNAseq.

### Mouse lung genomic data

We retrieved data from the Gene Expression Omnibus (GEO) database (https://www.ncbi.nlm.nih.gov/geo/). A total of 89 individual data sets from 15 studies (see supplementary Table S1& supplementary Figure S1) were selected based on the following criteria: Healthy mice of the C57BL/6 strain, comparable age and sex and identical experimental platform, i.e. Affymetrix Mouse Genome 430 2.0. We excluded treatment related genomic data or results from genetic animal models and data sets where the correct age and sex could not be determined. Shown in supplementary Figure S1 is the gender distribution over age. All 89 genomic data sets were used to compute the linear regression model and other statistical testing (see below).

### Mouse single cell genomic data

We considered single cell RNA-sequencing data to assign age-related gene expression changes to specific cell types of the lung. For this purpose we queried single cell transcriptome RNAseq counts data from GSE124872 [5] in addition to data deposited in four different databases, i.e. CellMarker (http://biocc.hrbmu.edu.cn/CellMarker/) [178], Mouse Cell Atlas (http://bis.zju.edu.cn/MCA/index.html) [179], LungGENS (https://research.cchmc.org/pbge/lunggens/mainportal.html) [180] and Lung Aging Atlas (http://146.107.176.18:3838/MLAA_backup/) [5].

### Human lung genomic data

We retrieved genomic data from the Cancer Genome Atlas (TCGA) (https://portal.gdc.cancer.gov/) and GEO public repository. Furthermore, we collected single cell genomic data from the CellMarker and the LungGENS database in addition to data from a research article [278]. Next, we divided the data into test and validation sets and provide information on the patient characteristics in supplementary Table S2. Note, the study cohorts are balanced for gender.

### Human lung genomic test set data

We retrieved RNA-Seq data of normal human lung from TCGA. We selected 107 individual data sets by the following criteria: “Primary Site” as “bronchus and lung”, “Sample type” as “solid tissue normal” defined by histology, “age at diagnosis”, i.e. 42–50 years (N = 5), 51–70 years (*N* = 62) and 71–86 years (*N* = 40).

#### Human lung genomic validation data

First, we retrieved 40 data sets derived of histologically normal lung tissue from 23 human donors (GSE1643). After removal of duplicates (*N* = 4) and specific location, i.e. upper lobe (*N* = 13) a total of 23 lower lobe lung samples were considered for further analysis.

Second, we considered the genomic data of 284 individuals who underwent lobectomy for lung adenocarcinoma and considered the genomic data of non-involved (apparently normal) lung parenchyma of this study (GSE71181). We selected three age groups, i.e. 21–50 years (*N* = 27), 51–70 years (*N* = 191) and 71–85 years (*N* = 89). Together, the human test and validation set consisted of 414 human lung tissue samples.

### Human single cell genomic data

We retrieved single cell RNA-seq data to assess age-related gene expression changes of specific cell types of the human lung. For this purpose, three databases were queried, i.e. CellMarker, LungGENS and data given in [278]. Subsequently the data were processed as detailed below.

### Data normalization

We performed computations in the R language (version 3.6.3) and applied the Robust Multi-array Average (RMA) algorithm (“affy” package, version 1.66.0) for quantile normalization and background correction of the mouse genomic data. Likewise, we used the “Seurat” package (version 4.3.0) to normalize single cell RNAseq data as CPM. In the case of the human test set data, we selected the “DESeq2” package (version 1.26.0) to normalize RNA-seq counts. The human validation set data consisted of microarray transcriptomic data which have been processed by the “GC Robust Multi-array Average (GCRMA)” method (GSE1643) and the “robust spline normalization” and “ComBat batch adjustment” algorithm (GSE71181). We only considered genes with a signal intensities of > 75.

### Differentially Expressed Genes (DEGs)

In order to define age-dependent gene expression changes and to compare mouse and human lung genomes, we analyzed the RMA normalized mouse and human validation transcriptomic data by a liener regression model and by computing the Linear Models for Microarray data (LIMMA, version 3.42.2). In the case of LIMMA, we selected DEGs based on the following criteria: Signal intensity > 75, Benjamini-Hochberg (BH) -adjusted *p*-value < 0.05 [279] and a fold change (FC) ≥ 1.5-fold. For the human test set data, we selected the package “DESeq2”, and DEGs were considered statistically significant based on BH-adjusted *p*-value < 0.05 and FC ≥ 1.5-fold. Statistical testing of single cell transcriptome data involved the “Wilcoxon rank-sum” test (within the “Seurat” package), and we considered significant DEGs based on BH-adjusted *p*-value < 0.05 and a FC ≥ 1.5-fold.

Apart from identifying significantly regulated genes by the linear regression model and LIMMA, we also applied the hypergeometric test to measure statistical significance. We observed good agreement between the methods. For instance, for down-regulated genes the range is 90%-98% when the results from LIMMA and the hypergeometric test were compared (supplementary Figure S9).

Furthermore, we constructed heatmaps by utilizing the function “pheatmap” (version 1.0.12) in R and applied the complete clustering method. We used the Z-score to plot the heatmap.

### Gene enrichment analysis

#### Gene ontology enrichment analysis

We employed the software: Metascape [280], DAVID [281] and the g:Profiler resource [282] to identify enriched Gene Ontology (GO) terms [283]. Only terms with a *p*-value < 0.05 and a BH-adjusted *p*-value < 0.05 were considered. Subsequently, we determined the consensus of enriched terms among the different software to define commonalities between them.

##### Gene set enrichment analysis (GSEA)

We computed the gene set enrichment analysis (GSEA) [31] with the “clusterProfiler” tool in R (version 3.14.3) [284]. For the mouse and human genomic data, we retrieved information deposited in the “Gene Set Knowledgebase (GSKB)” (version 1.18.0) [285] and the Molecular Signature Database (http://www.gsea-msigdb.org/gsea/msigdb/index.jsp) [31]. We normalized the enrichment scores (ES) based on random sampling of ES of the same gene set size and applied an absolute “normalized enrichment score” (NES) > 1.5 and a *p*-value < 0.05 for further analysis.

##### Single-sample gene set enrichment analysis (ssGSEA)

This procedure is an extension of the GSEA method and is computed in R with the tool “Gene Set Variation Analysis (GSVA)” (version 1.34.0) [286]. The results are presented as violin plots separated by the different age groups.

### Venn diagram analysis

Venn diagrams [287] were drawn to show the relationship between different DEGs and enrichment terms among the different ages.

### Statistical analysis

We used R to compute a linear regression model for all mouse and human data sets, i.e. *N* = 89 mouse and *N* = 414 human lung samples. Genes with a *p*-value < 0.05 and R2 > 0.4 were considered statistically significant. Furthermore, we used Bioconducter to perform statistical computations and applied the “Shapiro–Wilk” test for normality to evaluate the data distribution, and the “Bartlett” test to assess the homogeneity of variance for time dependent gene expression changes. If the data were not normally distributed, we used the “Kruskal–Wallis” and “Wilcoxon rank-sum” test (Mann–Whitney-U-Test) for significance testing. Specifically, the “Kruskal–Wallis” test provided an indiscriminate estimate of time dependent changes in the expression of gene markers of different cell types of the lung where as the “Wilcoxon rank-sum” test discriminated among the different age groups. Furthermore, we assessed the distribution of gene markers for pulmonary resident cells with the Kolmogorov–Smirnov test (see ssGSEA) which assesses the distribution of gene markers by comparing their position and distribution in individual lung samples from young and aged mouse and human pulmonary tissue samples.

The data are visualized as violin- and boxplots with interquartile range (IQR) and 1.5*IQR whiskers with outliers defined as data points outside the whiskers. A *p*-value of < 0.05 is considered significant.

### Supplementary Information

**Additional file 1: Supplementary Figure S1. **The age distribution of 89 individual mouse data sets retrieved from 15 studies.** Supplementary Figure S2. **Computation a linear regression model considering all the data sets (supplementary Table S1) but separated them by sex. We only considered DEGs that fulfilled the criteria: FDR adjusted *p*-value of 0.05 and a coefficient of determination R2>0.5.** Supplementary Figure S3. **Linear Regression Model for mouse data (89 samples).** Supplementary Figure S4. **Overlapped genes between the linear regression model with significantly regulated genes as defined by the DESeq2 method in human test set. We retrieved RNA-Seq data of 107 histologically proven normal lung tissue samples from the TCGA repository, i.e. resection material from lung cancer patients (supplementary Table S2).The cohort consisted of individuals aged 42-86 years, and based on normalized counts the linear regression model fitted 237 significantly regulated genes (27 up- and 210 down-). We also analyzed the RNAseq data with the DESeq2 package and compared individuals aged 42-50 (*N*=5) to 71-86 (*N*=40) old ones. This defined 1430 genes (716 up-, 714 down-) with a FC≥1,5 and an FDR adjusted *p*-value <0.05. Note, 56% of genes coming from the linear regression model overlap with significantly regulated genes as defined by the DESeq2 method.** Supplementary Figure S5. **Consensus among different gene ontology tools. **Supplementary Figure S6.** Continuous gene expression changes of the aging mouse lung by computing a linear regression model with 89 mouse samples.** Supplementary Figure S7. **Validation of marker genes by single cell RNA sequencing of pulmonary cells. We retrieved marker genes from various databases and considered their age-dependent regulation in pulmonary cells by single cell RNA sequencing. We computed enrichment scores based on a large set of genes (range 44-434 genes for the mouse, supplementary Table S16). By interrogating single cell RNAseq data of 7 different pulmonary cells, we confirmed the results in Figure 10B&C by an independent method. We computed statistical significance with the “Wilcoxon rank-sum” test. ns: not significant, *p<0.05, **p<0.01, ***p<0.001.** Supplementary Figure S8. **Comparative human pulmonary genomics of smokers and non-smokers. In order to examine the influence of tobacco product use on age-related gene expression changes, we compared the genomic data of morphologically unaltered lung tissue of 45 smokers to 7 non-smokers. We show that tobacco product use per se did not influence the expression of age-regulated genes reported in the present study; however, tobacco smoke exposure caused marked induction of xenobiotic defense genes. ns: not significant, Wilcoxon rank sum test, *p<0.05.** Supplementary Figure S9. **Computation of DEGs for the mouse lung by two methods, i.e. Linear Models for Microarray data (LIMMA) and the hypergeometric test.

**Additional file 2: Supplementary Table S1.** Detailed information of 89 individual mouse genome data sets. **Supplementary Table S2.** Sample characteristics of human genomic data sets. **Supplementary Table S3.** Gene set enrichment analysis of the mouse genomic data. **Supplementary Table S4.** Linear regression model defined 77 up- and 13 down-regulated genes whose expression changed with age. **Supplementary Table S5.** Regulation of ECM coding genes in mice. **Supplementary Table S6.** Commonly regulated DEGs between the human test and validation set. **Supplementary Table S7.** 798 housekeeping genes in mice which do not change their expression with age. **Supplementary Table S8. **Inferred compositional changes of ECM in the lung of mice. **Supplementary Table S9. **Genes of the senescence-associated secretory phenotype. **Supplementary Table S10.** We compared the age-dependent gene expression changes of the present study to published findings. **Supplementary Table S11. **Data retrieval to define marker gene sets for different cells of the lung. **Supplementary Table S12. **List of individual marker gene sets.**Supplementary Table S13. **ssGSEA enrichment score for different mouse and human pulmonary cells. **Supplementary Table S14. **Commonly regulated DEGs between human and mouse pulmonary genomes.

## Data Availability

The data supporting the findings of this study are available from GEO database (GSE38594, GSE66721, GSE55162, GSE34378, GSE38754, GSE23106, GSE25640, GSE18341, GSE15999, GSE14525, GSE11662, GSE10246, GSE9954, GSE6591, GSE3100, GSE124872, GSE1643, GSE71181) and TCGA database (supplementary Table S1 and S2).

## Abbreviations

Abca3: ATP binding cassette subfamily A member 3
Adamts: ADAM metallopeptidase with thrombospondin type 1 motif 1
AEBP1: Adipocyte enhancer-binding protein 1
ALAS2: 5'-Aminolevulinate synthase 2)
Alcam: Activated leukocyte cell-adhesion molecule
Anxa: Annexin
AT1: Type 1 pneumocytes
AT2: Type 2 pneumocytes
BH: Benjamini-Hochberg
CCDC80: Coiled-coil domain containing 80
Ccl: C-C motif chemokine ligand
Ccl11: Eotaxin (C–C motif chemokine ligand 11
Ccn2: Cellular communication network factor 2
Ccr2: C-C motif chemokine receptor 2
Cd177: CD177 molecule
Cd3d: CD3 delta subunit of T-cell receptor complex
Cd3e: CD3 epsilon subunit of T-cell receptor complex
Cd3g: CD3 gamma subunit of T-cell receptor complex
Cd3z: CD3 zeta subunit of T-cell receptor complex
Cd45: Protein tyrosine phosphatase receptor type C
CDK: Cyclin dependent kinase
Cept1: Choline/ethanolamine phosphotransferase 1
chILD: Childhood interstitial lung disease
CHKA: Choline kinase alpha
CHPT1: Choline phosphotransferase
CLSTN2: Calsyntenin 2
COPD: Chronic obstructive pulmonary disease
CSF: The colony stimulating factor
CTHRC1: Collagen triple helix repeat containing 1
Cxcl: C-X-C motif chemokine ligand
Cxcl13; Blc: C-X-C motif chemokine ligand 13
CYP1A1: Cytochrome P450 family 1 subfamily A member 1
DDR: DNA damage response
DEG: Differentially expressed genes
DGK: Diacylglycerol kinase
DPLD: Diffuse parenchymal lung disease
ECM: Extracellular matrix
Eln: Elastin
Errfi1: ERBB receptor feedback inhibitor 1
ES: Enrichment scores
ETNK1: Ethanolamine kinase
Evi2a: Ecotropic viral integration site 2A
FBLN2: Fibulin 2
FC: Fold change
Fga: Fibrinogen alpha chain
Fgb: Fibrinogen beta chain
Fgg: Fibrinogen gamma chain
FOXO3: Forkhead box O3
Gata6: GATA binding protein 6
GCRMA: GC robust multi-array average
GEO: Gene Expression Omnibus
GO: Gene Ontology
GPCR: G protein-coupled receptor
Grhl2: Grainyhead like transcription factor 2
GSEA: Gene set enrichment analysis
GSKB: Gene Set Knowledgebase
GSVA: Gene set variation analysis
Hist1h4m: Nucleosome H4 clustered histone 17
HS: Heparan sulfate
Htra1: HtrA serine peptidase 1
Icos: Inducible T cell co-stimulator
Icosl: Inducible T cell co-stimulator ligand
IGF: Insulin like growth factor
IGF1R: Insulin like growth factor 1 receptor
IGFBP: Insulin-like growth factor binding proteins
Igha: Immunoglobulin heavy constant alpha
Ighg: Immunoglobulin heavy constant gamma
Ighm: Immunoglobulin heavy constant mu
Il7r: Interleukin 7 receptor alpha chain
ILD: Interstitial lung disease
IPF: Idiopathic pulmonary fibrosis
IQR: Interquartile range
ITAMs: Immunoreceptor tyrosine-based activation motif
Kdelr3: (Lys-Asp-Glu-Leu (KDEL) endoplasmic reticulum protein retention receptor 3
KLF: KLF transcription factor
Lama2: Laminin subunit alpha 2
Lama4: Laminin subunit alpha 4
Lamb1: Laminin subunit beta 1
Lamc1: Laminin subunit gamma 1
Lat: Linker for activation of T cells
Lck: Lymphocyte-specific protein tyrosine kinase
Lgals1: Galectin 1
Lgr5: Leucine rich repeat containing G protein-coupled receptor 5
LIMMA: Linear models for microarray data
Lpin: Lipin
Lrg1: Leucine‐rich α‐2 glycoprotein
M: Macrophage
Mfap: Microfibril associated proteins
MMP: Matrix metalloproteinase
Ms4a6b: Membrane-spanning 4-domains, subfamily A, member 6B
MT1G: Metallothionein 1G
NAMPT: Nicotinamide phosphoribosyltransferase
NES: Normalized enrichment score
NFIL3: Nuclear factor interleukin 3 regulated
Nid: Nidogen
NLRP3: NLR family pyrin domain containing 3
Nnt: Nicotinamide nucleotide transhydrogenase
Nrf2: Nuclear factor erythroid 2 related factor 2
ORM1: Orosomucoid 1
OSR1: Odd-skipped related transcription factor 1
p16; Cdkn2a: Cyclin dependent kinase inhibitor 2A
p21; Cdkn1a: Cyclin dependent kinase inhibitor 1A
PA: Phosphatidic acid
Pax8: Paired box 8
PC: Phosphatidylcholine
PCA: Principal component analysis
PCYT1A: Choline-phosphate cytidylyltransferase
Pdgfrl: Platelet-derived growth factor receptor-like gene
PE: Phosphoethanolamine
PEMT: Phosphatidylethanolamine N-methyltransferase
Pgc1: Peroxisome proliferator-activated receptor γ coactivator-1α
Pla2g5: Phospholipase A2 isoform 5
Plpp: Phospholipid phosphatases
PLVAP: Plasmalemma vesicle‑associated protein
PS: Phosphatidylserine
Pss; Ptdss: Phosphatidylserine synthase
PTPRT: Protein tyrosine phosphatase receptor type T
RMA: Robust multi-array average
ROS: Reactive oxygen species
S100a4: S100 calcium binding protein A4
SASP: Senescence-associated secretory phenotyp
Scgb1a1: Secretoglobin family 1A member 1
Sin3a: SIN3 transcription regulator family member A
SLC4A1: Solute carrier family 4 member 1
Slp76: Lymphocyte cytosolic protein 2
SMA: Smooth muscle actin
Socs2: Suppressor of cytokine signaling 2
SOD2: Superoxide dismutase 2
ssGSEA: Single-sample gene set enrichment analysis
St8sia6: ST8 alpha-N-acetyl-neuraminide alpha-2,8-sialyltransferase 6
SULF2: Sulfatase 2
TCGA: The Cancer Genome Atlas
Terf: Telomeric repeat binding factor
Tert: Telomerase reverse transcriptase
THY1: Thy-1 cell surface antigen
TIMP: TIMP metallopeptidase inhibitor
Tnc: Tenascin C
Trp53inp1: The p53 inducible nuclear protein 1
Trpml3: Endolysosomal cation channel mucolipin 3
WNT7B: Wnt family member 7B
Zap70: Zeta chain of T cell receptor associated protein
