# Correction: A genomic perspective of the aging human and mouse lung with a focus on immune response and cellular senescence

**DOI:** 10.1186/s12979-023-00407-y

**Published:** 2024-01-16

**Authors:** Meng He, Jürgen Borlak

**Affiliations:** Centre for Pharmacology and Toxicology, Hannover Medical School, Carl‑Neuberg‑Str. 1, 30625 Hannover, Germany

**Correction: Immun Ageing 20, 58 (2023)**

10.1186/s12979-023-00373-5

Following publication of the original article [1], the authors reported an error in the HTML version of this article. The graphical abstract displayed is not the correct image but, a copy of Fig. 10 and in addition Fig. 11 is not fully displayed.

The publishers apologise for this error.

The original article [1] has been updated.
