# Supplementary material for: A genomic perspective of the aging human and mouse lung with a focus on immune response and cellular senescence
Source: Immun Ageing. 2023 Nov 6;20:58. doi: 10.1186/s12979-023-00373-5 (PMC10626779; doi:10.1186/s12979-023-00373-5)

Supplementary Figure S1

The age distribution of 89 individual mouse data sets retrieved from 15 studies.
See supplementary Table S1 for further details.

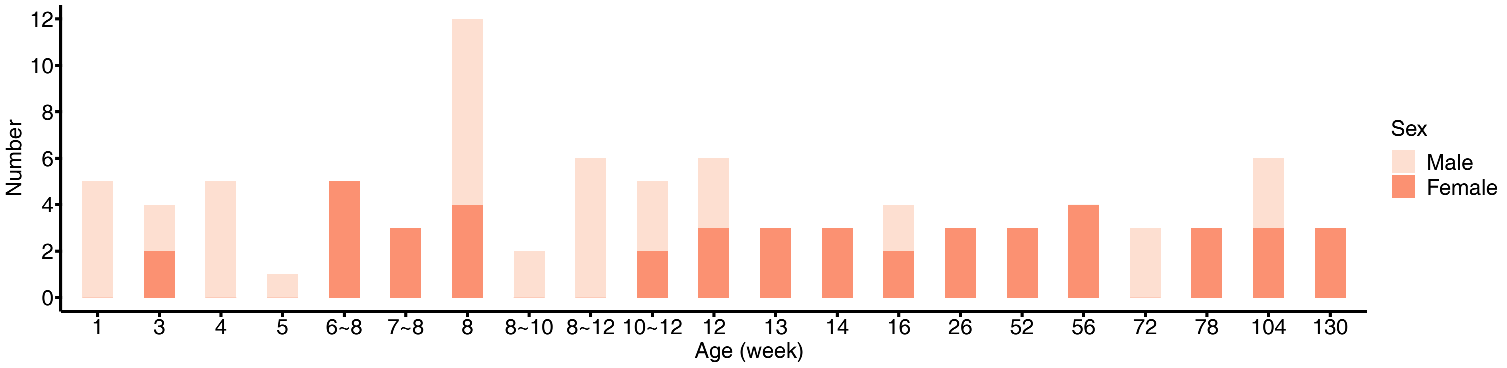

Supplementary Figure S2

Computation a linear regression model considering all the data sets (supplementary Table S1) but separated them by sex.
We only considered DEGs that fulfilled the criteria: FDR adjusted p-value of 0.05 and a coefficient of determination R2>0.5.

A Venn diagram of the upregulated genes comparison male vs female, with all the male and female data, separately. This revealed 305 up in male and 145 up in female DEGs fitting the regression model.

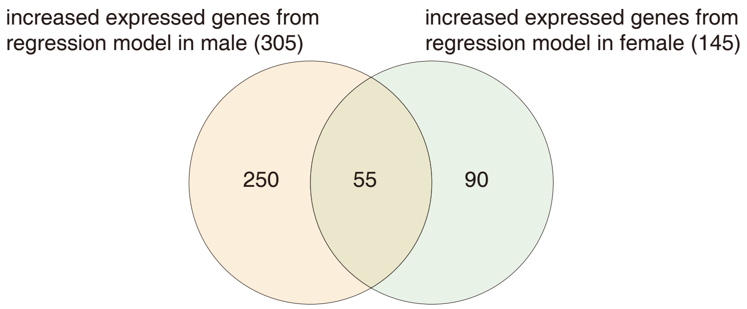

Male mice specific 250 upregulated genes enrichment analysis:

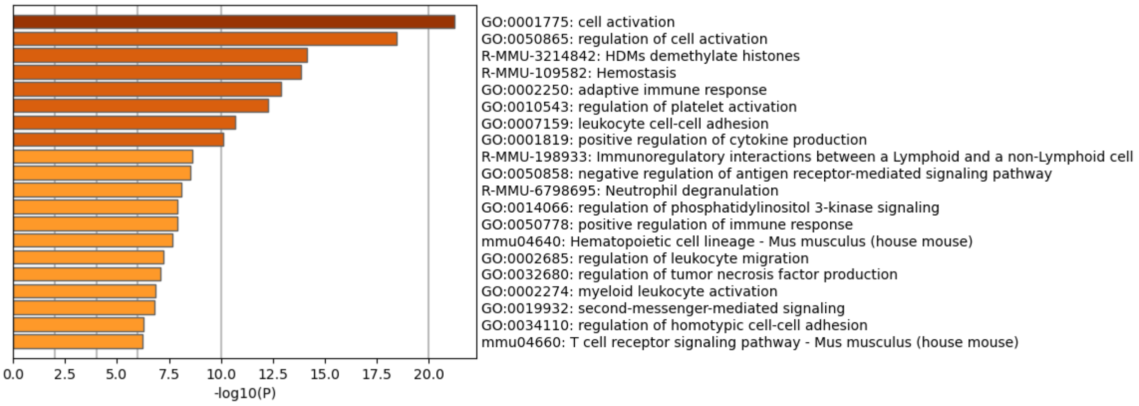

Female mice specific 90 upregulated genes enrichment analysis:

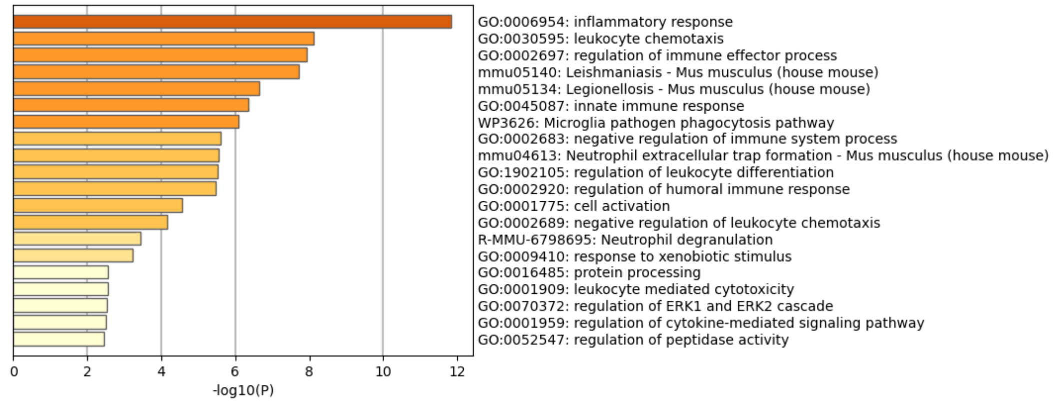

B Venn diagram of the downregulated genes comparison male vs female, with all the male and female data, separately. This revealed 386 down in male and 126 down in female DEGs fitting the regression model.

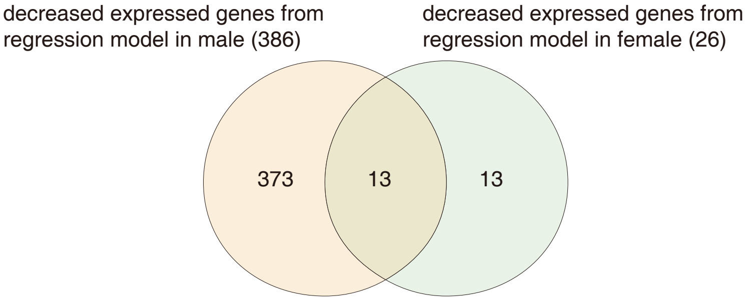

Male mice specific 373 downregulated genes enrichment analysis:

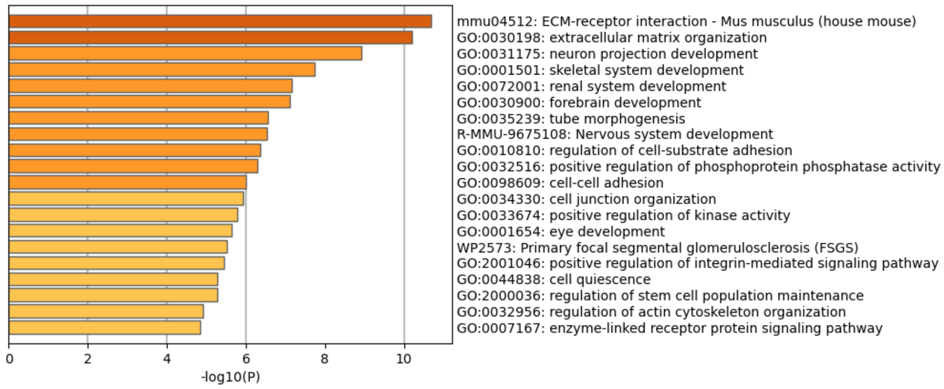

Female mice specific 13 downregulated genes enrichment analysis:

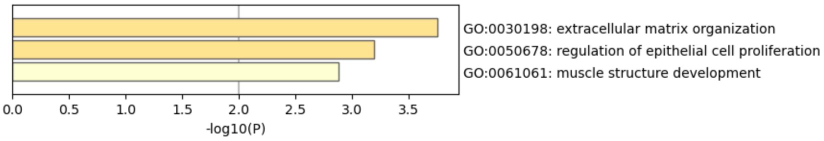

Supplementary Figure S3

Linear Regression Model for mouse data (89 samples)

This showed the genes which R2>0.5

Genes：Igkv4-72 Clec4e Igkv6-14 Cst7 Cxcl2

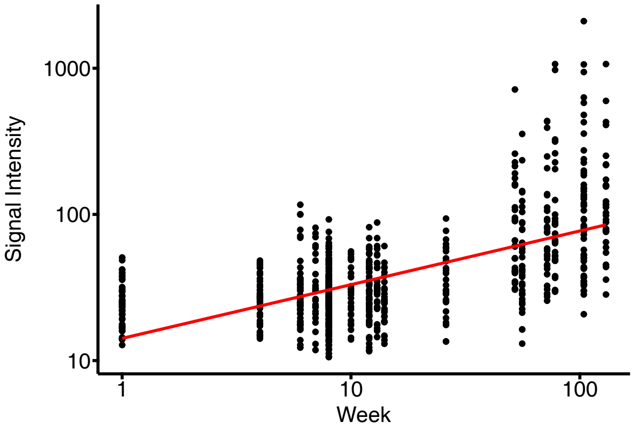

Genes：Gzmk Ighg Ighg3 Il7 Slc6a20a Trgv2

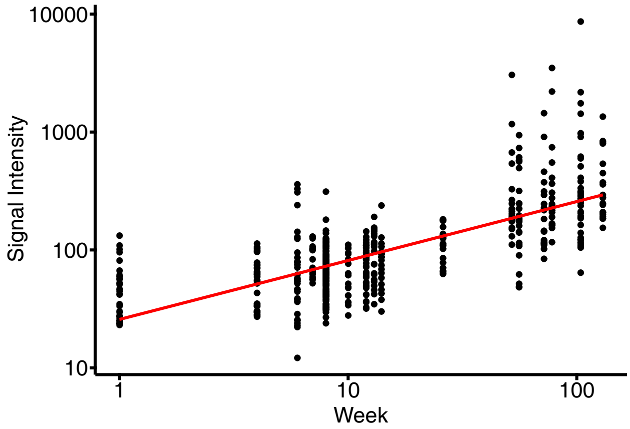

Genes：Ccl8 Cxcl13 Fyb Igk Il1b Jchain Mzb1 Pla2g7 Slc2a3 Wfdc17

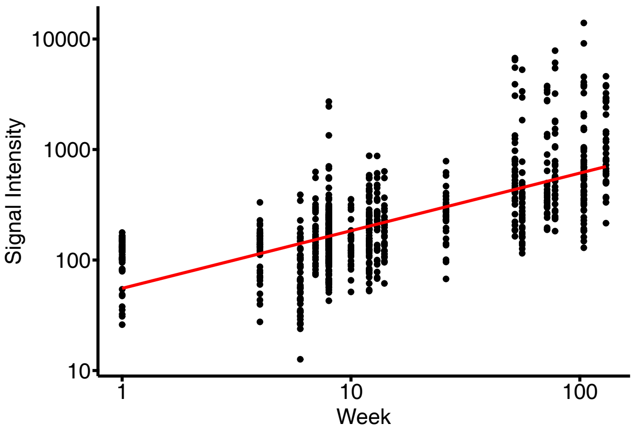

Genes：Ccl9 Ighm Aplnr

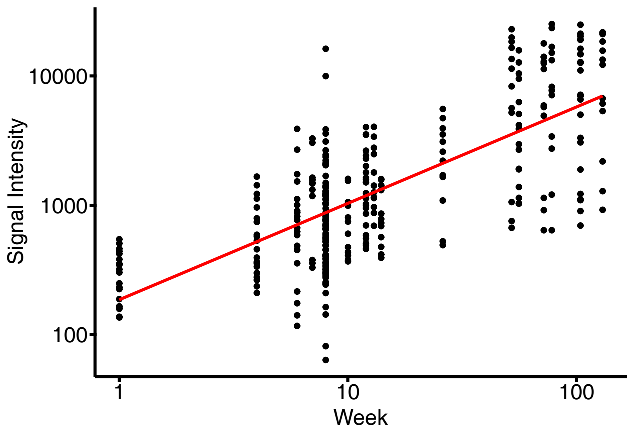

Genes：Col3a1 Nrep Thbs3

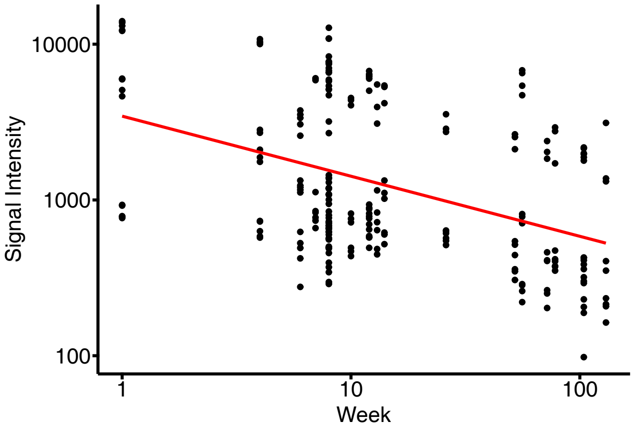

Supplementary Figure S4

Overlapped genes between the linear regression model with significantly regulated genes as defined by the DESeq2 method in human test set. We retrieved RNA-Seq data of 107 histologically proven normal lung tissue samples from the TCGA repository, i.e. resection material from lung cancer patients (supplementary Table S2). The cohort consisted of individuals aged 42-86 years and based on normalized counts, the linear regression model fitted 237 significantly regulated genes (27 up and 210 down). We also analysed the RNAseq data with the DESeq2 package and compared individuals aged 42-50 (N=5) to 71-86 (N=40) old ones. This defined 1430 genes (716 up, 714 down) with a FC $\geq$ 1,5 and an FDR adjusted p-value <0.05. Note, 56% of genes coming from the linear regression model overlap with significantly regulated genes as defined by the DESeq2 method.

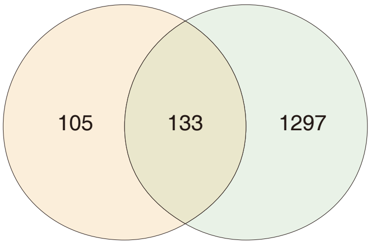

Supplementary Figure S5

Consensus among different gene ontology tools

A Shown are Venn diagrams for enriched terms for upregulated genes in mice aged 6-26 week compared to 52-130 week ones.

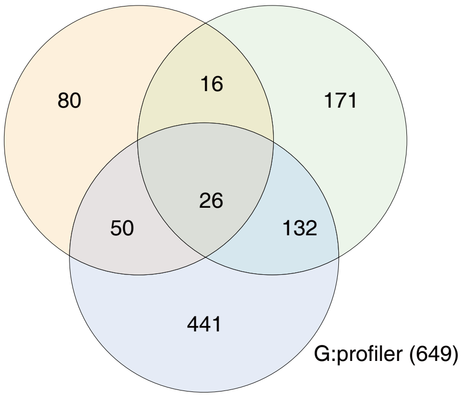

B Shown are Venn diagrams enriched terms for downregulated genes in mice aged 6-26 week compared to 52-130 week ones.

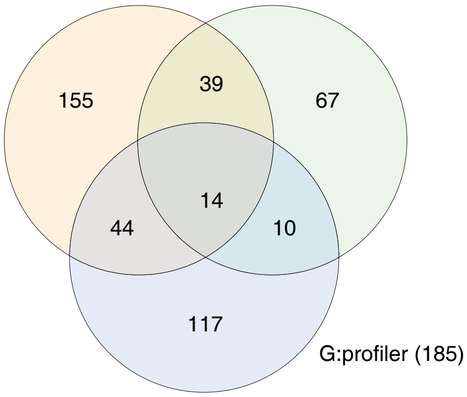

Supplementary Figure S6

Continuous gene expression changes of the aging mouse lung by computing a linear regression model with 89 mouse samples.
This defined 91 up- and 14 down-regulated genes whose expression changed with age.
Panel A1: Box plot of 91 DEGs with increased expression over time.
Panel A2: Box plots of upregulated genes coding for immune response.
Panel B: Regulation of individual immune genes with age. The genes refer to the data shown in the box plots of A2 for the various ontology terms.
Panel C1: Box plot for 14 significant DEGs with repressed expression over time.
Panel C2: Box plot for significantly downregulated DEGs based on enriched gene ontology terms.
Panel D: Regulation of individual genes with age. The genes refer to the data shown in the box plots of C2 for the various ontology terms.
In Figure S6, we used the “Kruskal-Wallis” test. *p<0.05. M.: Macrophage

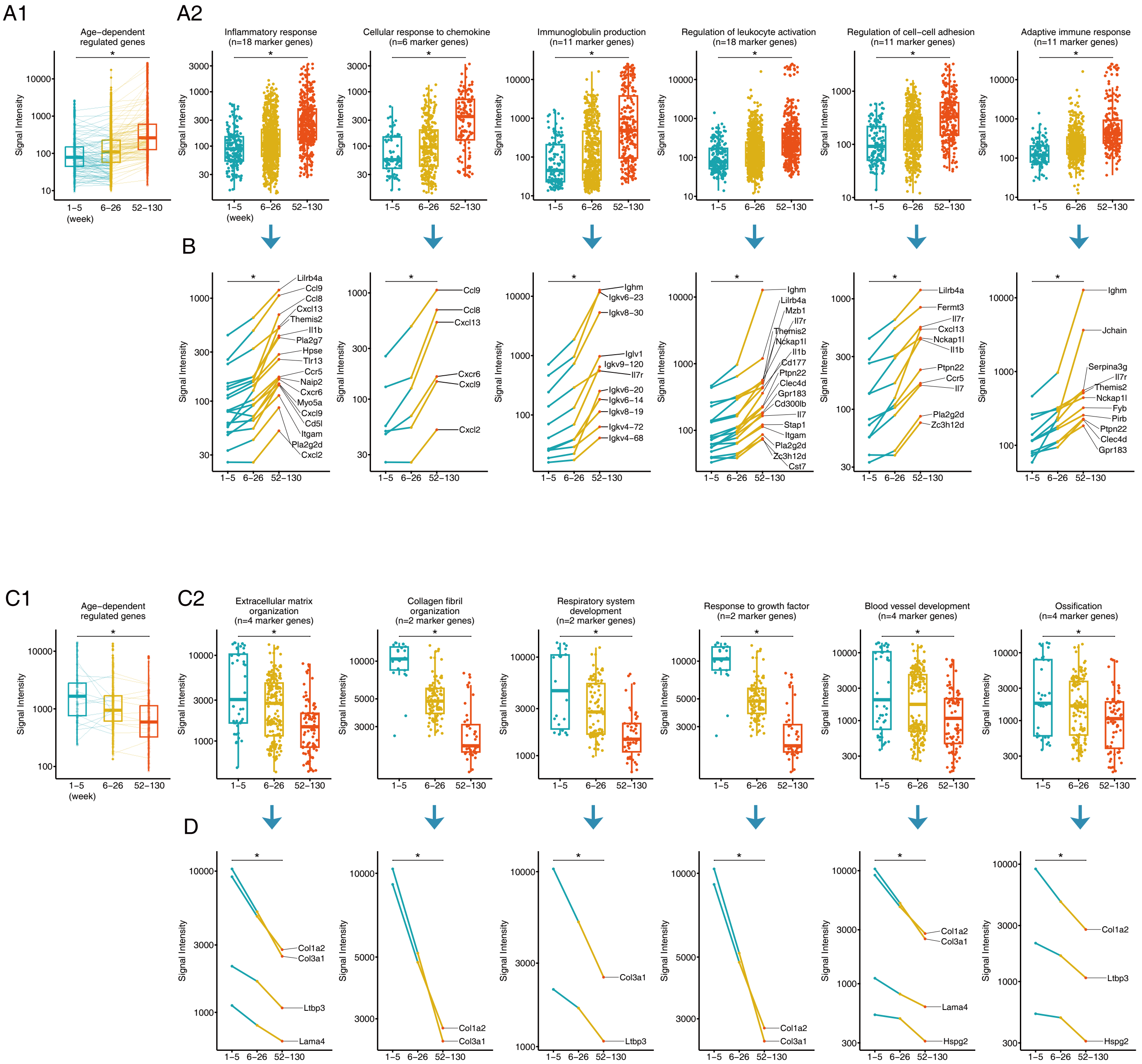

Supplementary Figure S7

Validation of marker genes by single cell RNA sequencing of pulmonary cells.

We retrieved marker genes from various databases and considered their age-dependent regulation in pulmonary cells by single cell RNA sequencing. We computed enrichment scores based on a large set of genes (range 44-434 genes for the mouse, supplementary Table S16). By interrogating single cell RNAseq data of 7 different pulmonary cells, we confirmed the results in Figure 10B&C by an independent method. We computed statistical significance with the "Wilcoxon rank-sum" test. ns: not significant, *p<0.05, **p<0.01, ***p<0.001.

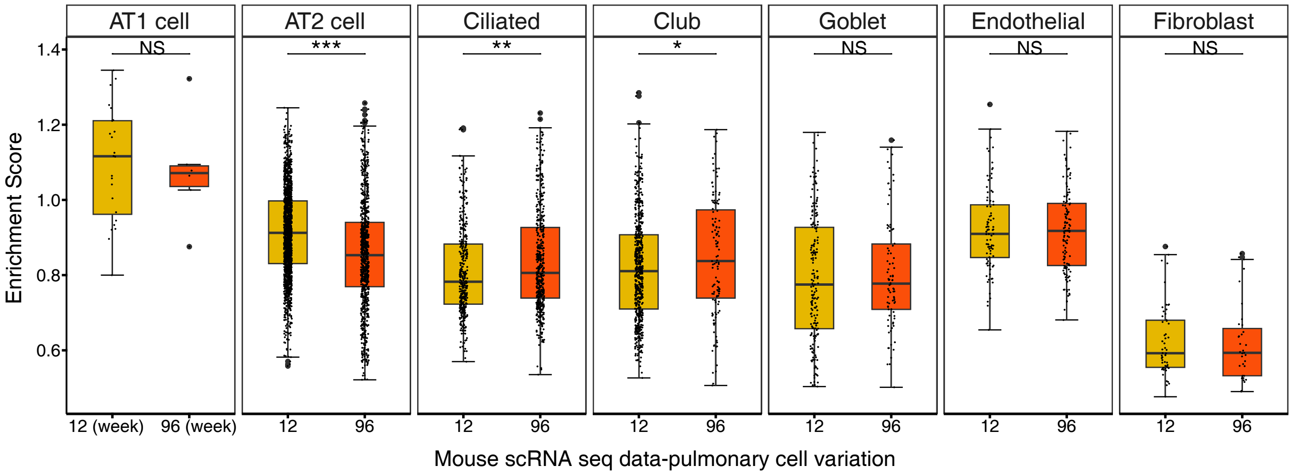

Supplementary Figure S8

Comparative human pulmonary genomics of smokers and non-smokers.
In order to examine the influence of tobacco product use on age-related gene expression changes, we compared the genomic data of morphologically unaltered lung tissue of 45 smokers to 7 non-smokers. We show that tobacco product use per se did not influence the expression of age-regulated genes reported in the present study; however, tobacco smoke exposure caused marked induction of xenobiotic defense genes.
ns: not significant, Wilcoxon rank sum test, *p<0.05.

A Box plots of 20 genes highly regulated following tobacco smoke exposure.

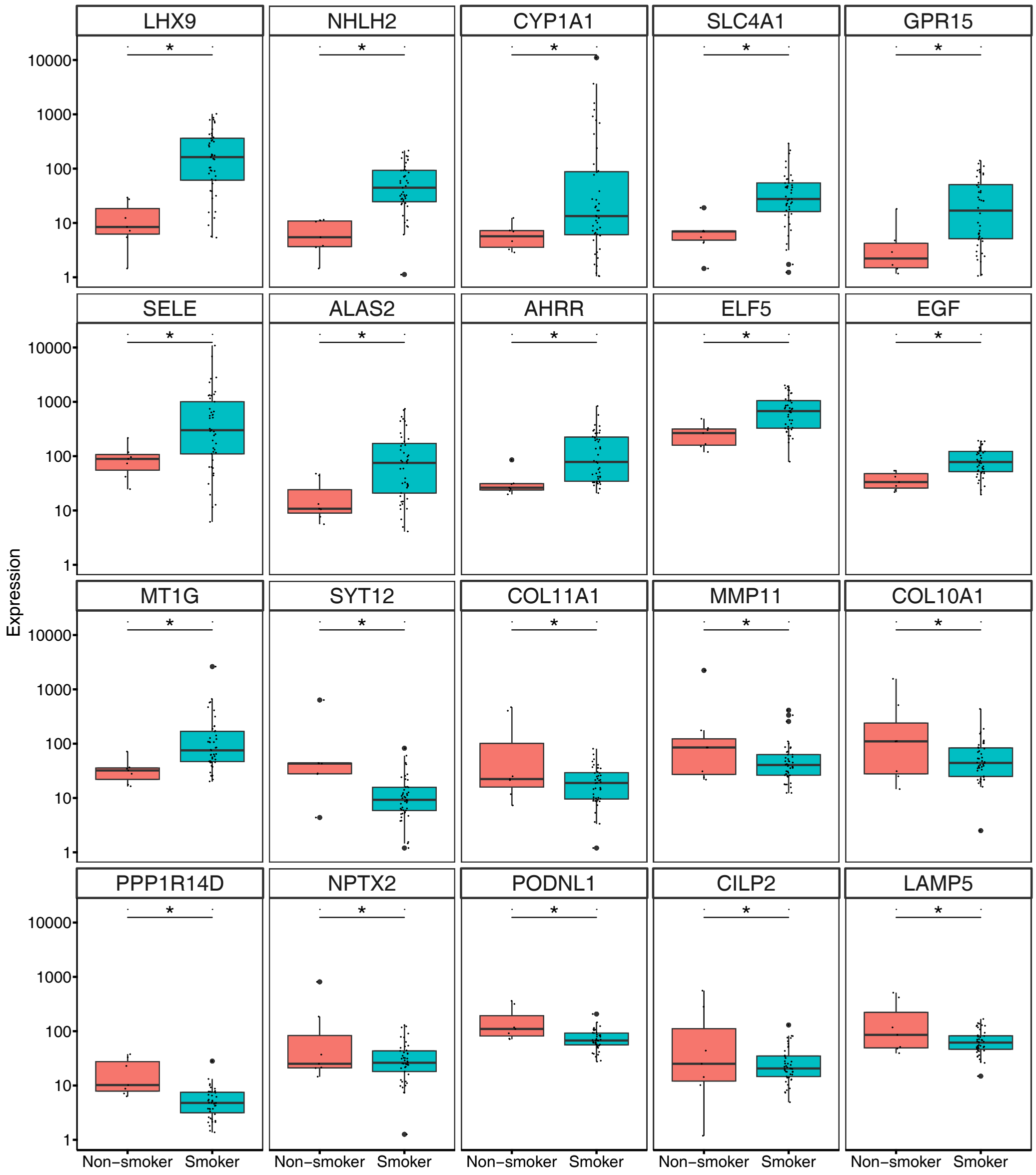

B

None of the age-related gene expression changes in the human lung were influenced by tobacco use.

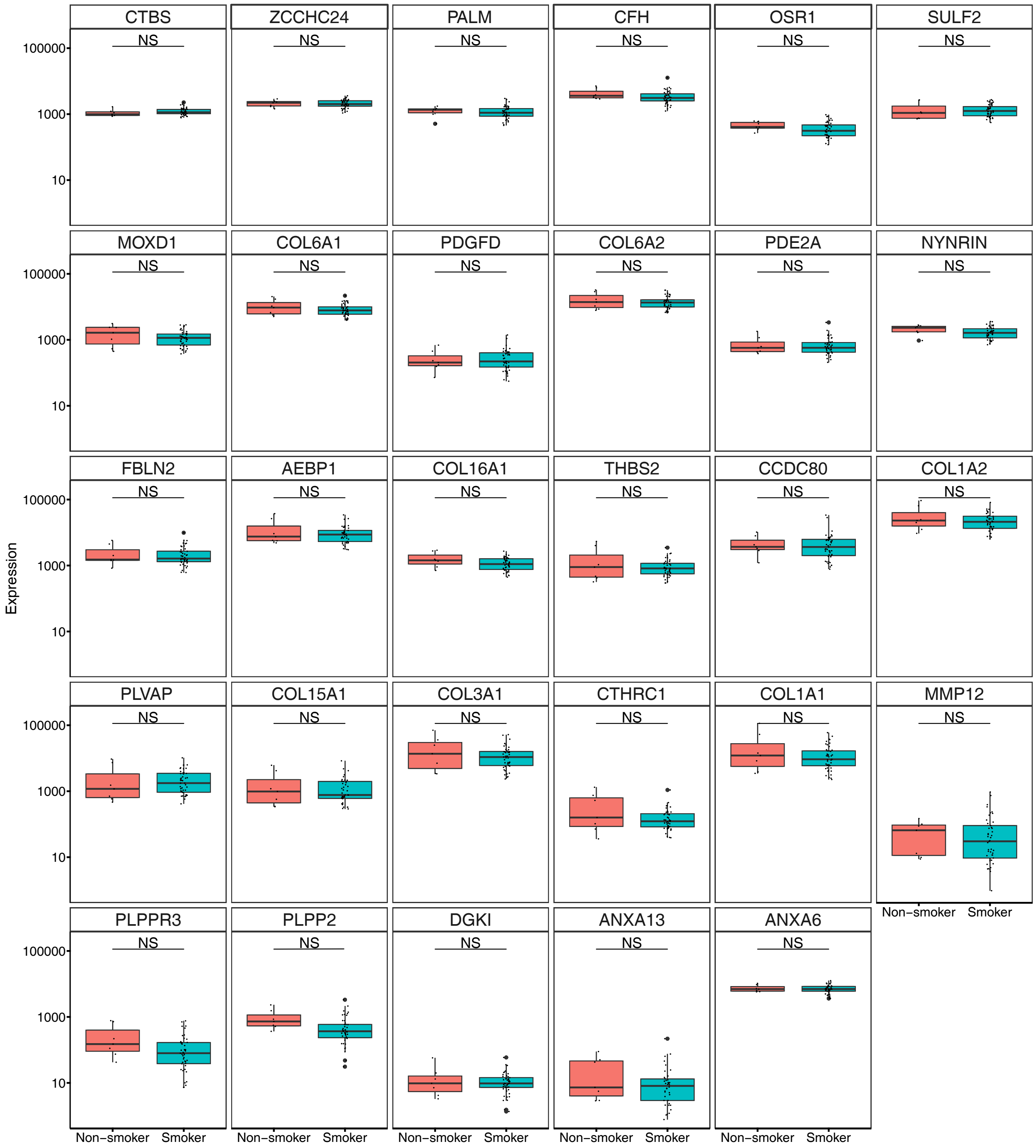

Supplementary Figure S9

Computation of DEGs for the mouse lung by two methods, i.e. Linear Models for Microarray data (LIMMA) and the hypergeometric test. We compared DEGs from the two statistical tests using the following criteria: BH-adjusted p-value <0.05 and FC $\geq$ 3-fold.

A Venn diagram of the comparison Week 1-5 vs Week 6-26, for upregulated DEGs

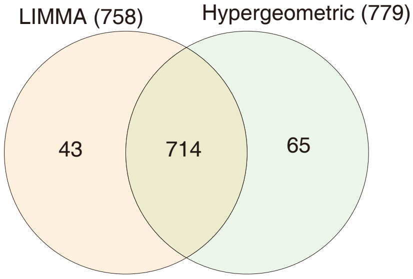

B Venn diagram of the comparison Week 1-5 vs Week 6-26, for downregulated DEGs

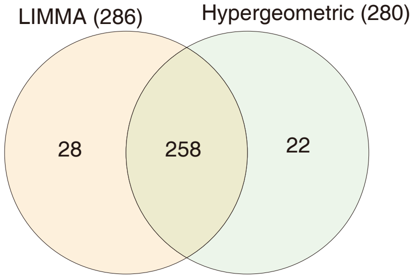

Supplement: Supplementary file 1 — Additional file 1: Supplementary Figure S1. The age distribution of 89 individual mouse data sets retrieved from 15 studies. Supplementary Figure S2. Computation a linear regression model considering all the data sets (supplementary Table S1) but separated them by sex. We only considered DEGs that fulfilled the criteria: FDR adjusted p-value of 0.05 and a coefficient of determination R2>0.5. Supplementary Figure S3. Linear Regression Model for mouse data (89 samples). Supplementary Figure S4. Overlapped genes between the linear regression model with significantly regulated genes as defined by the DESeq2 method in human test set. We retrieved RNA-Seq data of 107 histologically proven normal lung tissue samples from the TCGA repository, i.e. resection material from lung cancer patients (supplementary Table S2).The cohort consisted of individuals aged 42-86 years, and based on normalized counts the linear regression model fitted 237 significantly regulated genes (27 up- and 210 down-). We also analyzed the RNAseq data with the DESeq2 package and compared individuals aged 42-50 (N=5) to 71-86 (N=40) old ones. This defined 1430 genes (716 up-, 714 down-) with a FC≥1,5 and an FDR adjusted p-value <0.05. Note, 56% of genes coming from the linear regression model overlap with significantly regulated genes as defined by the DESeq2 method. Supplementary Figure S5. Consensus among different gene ontology tools. Supplementary Figure S6. Continuous gene expression changes of the aging mouse lung by computing a linear regression model with 89 mouse samples. Supplementary Figure S7. Validation of marker genes by single cell RNA sequencing of pulmonary cells. We retrieved marker genes from various databases and considered their age-dependent regulation in pulmonary cells by single cell RNA sequencing. We computed enrichment scores based on a large set of genes (range 44-434 genes for the mouse, supplementary Table S16). By interrogating single cell RNAseq data of 7 different pulmon [file 12979_2023_373_MOESM1_ESM.pdf]
