# Supplementary material for: A genomic perspective of the aging human and mouse lung with a focus on immune response and cellular senescence
Source: Immun Ageing. 2023 Nov 6;20:58. doi: 10.1186/s12979-023-00373-5 (PMC10626779; doi:10.1186/s12979-023-00373-5)
Supplement: Supplementary file 2 — Additional file 2: Supplementary Table S1. Detailed information of 89 individual mouse genome data sets. Supplementary Table S2. Sample characteristics of human genomic data sets. Supplementary Table S3. Gene set enrichment analysis of the mouse genomic data. Supplementary Table S4. Linear regression model defined 77 up- and 13 down-regulated genes whose expression changed with age. Supplementary Table S5. Regulation of ECM coding genes in mice. Supplementary Table S6. Commonly regulated DEGs between the human test and validation set. Supplementary Table S7. 798 housekeeping genes in mice which do not change their expression with age. Supplementary Table S8. Inferred compositional changes of ECM in the lung of mice. Supplementary Table S9. Genes of the senescence-associated secretory phenotype. Supplementary Table S10. We compared the age-dependent gene expression changes of the present study to published findings. Supplementary Table S11. Data retrieval to define marker gene sets for different cells of the lung. Supplementary Table S12. List of individual marker gene sets.Supplementary Table S13. ssGSEA enrichment score for different mouse and human pulmonary cells. Supplementary Table S14. Commonly regulated DEGs between human and mouse pulmonary genomes. [file 12979_2023_373_MOESM2_ESM.pdf]

Supplementary Table S1
Detailed information of 89 individual mouse genome data sets

Mice-89 samples	GEO series
Week 5 male-1 samples	GSE83594
Week 6-8 female-5 samples	GSE66721
Week 8 female-4 samples<br>Week 56 female-4 samples	GSE55162
Week 13 female-3 samples<br>Week 26 female-3 samples<br>Week 52 female-3 samples<br>Week 78 female-3 samples<br>Week 104 female-3 samples<br>Week 130 female-3 samples	GSE34378
Week 1 male-5 samples<br>Week 4 male-5 samples<br>Week 8 male-5 samples	GSE38754
Week 14 female -3 samples	GSE23016
Week 7-8 female -3 samples	GSE25640
Week 3 male-2 samples<br>Week 3 female-2 samples<br>Week 16 male-2 samples<br>Week 16 female-2 samples	GSE18341
Week 10-12 female-2 samples	GSE15999
Week 8-12 male -3 samples	GSE14525
Week 8-12 male-3 samples	GSE11662
Week 8-10 male -2 samples	GSE10246
Week 10-12 male -3 samples	GSE9954
Week 8 male -3 samples<br>Week 72 male -3 samples<br>Week 104 male -3 samples	GSE6591
Week 12 male-3 samples<br>Week 12 female-3 samples	GSE3100

Supplementary Table S2
Sample characteristics of human genomic data sets

Human test set

Sample	Age	Sex	Disease	Histology of lung tissue	Tobacco smoke history
TCGA-55-6979-11a	59	female	Lung Adenocarcinoma	Normal lung tissue	Smoker
TCGA-91-6831-11a	66	male	Lung Adenocarcinoma	Normal lung tissue	Smoker
TCGA-44-6777-11a	85	female	Lung Adenocarcinoma	Normal lung tissue	Smoker
TCGA-44-3396-11a	74	female	Lung Adenocarcinoma	Normal lung tissue	Smoker
TCGA-49-6742-11a	70	male	Lung Adenocarcinoma	Normal lung tissue	Smoker
TCGA-50-5931-11a	75	female	Lung Adenocarcinoma	Normal lung tissue	Smoker
TCGA-44-6147-11a	67	female	Lung Adenocarcinoma	Normal lung tissue	Smoker
TCGA-55-6969-11a	52	male	Lung Adenocarcinoma	Normal lung tissue	Smoker
TCGA-49-6761-11a	68	female	Lung Adenocarcinoma	Normal lung tissue	Smoker
TCGA-44-5645-11a	61	female	Lung Adenocarcinoma	Normal lung tissue	Smoker
TCGA-91-6836-11a	52	female	Lung Adenocarcinoma	Normal lung tissue	Smoker
TCGA-55-6984-11A	71	female	Lung Adenocarcinoma	Normal lung tissue	Smoker
TCGA-49-4490-11a	45	female	Lung Adenocarcinoma	Normal lung tissue	Smoker
TCGA-49-6745-11a	82	male	Lung Adenocarcinoma	Normal lung tissue	Smoker
TCGA-55-6971-11a	59	female	Lung Adenocarcinoma	Normal lung tissue	Smoker
TCGA-44-2655-11a	65	female	Lung Adenocarcinoma	Normal lung tissue	Smoker
TCGA-44-2665-11a	55	female	Lung Adenocarcinoma	Normal lung tissue	Non-Smoker
TCGA-73-4676-11a	45	male	Lung Adenocarcinoma	Normal lung tissue	Smoker
TCGA-38-4625-11a	66	female	Lung Adenocarcinoma	Normal lung tissue	Smoker
TCGA-55-6986-11a	74	female	Lung Adenocarcinoma	Normal lung tissue	Non-Smoker
TCGA-55-6983-11a	81	male	Lung Adenocarcinoma	Normal lung tissue	Smoker
TCGA-49-4512-11a	69	female	Lung Adenocarcinoma	Normal lung tissue	Non-Smoker
TCGA-44-2661-11a	69	female	Lung Adenocarcinoma	Normal lung tissue	Non-Smoker
TCGA-38-4627-11a	64	female	Lung Adenocarcinoma	Normal lung tissue	Smoker
TCGA-49-6743-11a	81	female	Lung Adenocarcinoma	Normal lung tissue	Smoker
TCGA-44-6776-11a	60	female	Lung Adenocarcinoma	Normal lung tissue	Smoker
TCGA-50-5936-11A	58	male	Lung Adenocarcinoma	Normal lung tissue	Smoker

TCGA-91-6828-11a	70	male	Lung Adenocarcinoma	Normal lung tissue	Smoker
TCGA-44-6146-11a	64	male	Lung Adenocarcinoma	Normal lung tissue	Smoker
TCGA-44-6144-11a	58	male	Lung Adenocarcinoma	Normal lung tissue	Smoker
TCGA-50-5930-11A	47	male	Lung Adenocarcinoma	Normal lung tissue	Smoker
TCGA-50-5932-11A	75	male	Lung Adenocarcinoma	Normal lung tissue	Smoker
TCGA-38-4626-11a	57	female	Lung Adenocarcinoma	Normal lung tissue	Smoker
TCGA-50-5935-11A	86	female	Lung Adenocarcinoma	Normal lung tissue	Smoker
TCGA-38-4632-11a	42	male	Lung Adenocarcinoma	Normal lung tissue	Smoker
TCGA-91-6847-11a	62	female	Lung Adenocarcinoma	Normal lung tissue	Smoker
TCGA-55-6975-11a	61	male	Lung Adenocarcinoma	Normal lung tissue	Smoker
TCGA-50-5933-11A	72	male	Lung Adenocarcinoma	Normal lung tissue	Smoker
TCGA-44-6778-11a	59	male	Lung Adenocarcinoma	Normal lung tissue	Smoker
TCGA-91-6835-11a	81	female	Lung Adenocarcinoma	Normal lung tissue	Smoker
TCGA-44-2657-11a	74	female	Lung Adenocarcinoma	Normal lung tissue	Smoker
TCGA-50-5939-11a	85	male	Lung Adenocarcinoma	Normal lung tissue	Smoker
TCGA-49-6744-11a	64	female	Lung Adenocarcinoma	Normal lung tissue	Smoker
TCGA-55-6968-11a	61	male	Lung Adenocarcinoma	Normal lung tissue	Smoker
TCGA-55-6985-11a	58	female	Lung Adenocarcinoma	Normal lung tissue	Smoker
TCGA-50-6595-11a	74	female	Lung Adenocarcinoma	Normal lung tissue	Smoker
TCGA-55-6982-11a	79	female	Lung Adenocarcinoma	Normal lung tissue	Non-Smoker
TCGA-44-6148-11a	60	male	Lung Adenocarcinoma	Normal lung tissue	Smoker
TCGA-44-2662-11a	65	male	Lung Adenocarcinoma	Normal lung tissue	Smoker
TCGA-91-6829-11a	78	male	Lung Adenocarcinoma	Normal lung tissue	Smoker
TCGA-55-6981-11a	53	female	Lung Adenocarcinoma	Normal lung tissue	Smoker
TCGA-55-6978-11a	81	male	Lung Adenocarcinoma	Normal lung tissue	Non-Smoker
TCGA-44-2668-11a	51	male	Lung Adenocarcinoma	Normal lung tissue	Smoker
TCGA-55-6972-11a	72	male	Lung Adenocarcinoma	Normal lung tissue	Smoker
TCGA-55-6980-11a	56	male	Lung Adenocarcinoma	Normal lung tissue	Non-Smoker
TCGA-55-6970-11a	67	female	Lung Adenocarcinoma	Normal lung tissue	Smoker
TCGA-44-6145-11a	62	female	Lung Adenocarcinoma	Normal lung tissue	Smoker
TCGA-91-6849-11a	75	female	Lung Adenocarcinoma	Normal lung tissue	Smoker
TCGA-77-7142-11A	59	female	Lung Squamous Cell Carcinoma	Normal lung tissue	Smoker
TCGA-22-5472-11A	67	male	Lung Squamous Cell Carcinoma	Normal lung tissue	Smoker

TCGA-34-7107-11A	70	male	Lung Squamous Cell Carcinoma	Normal lung tissue	Smoker
TCGA-34-8454-11A	62	female	Lung Squamous Cell Carcinoma	Normal lung tissue	Smoker
TCGA-43-6143-11A	70	male	Lung Squamous Cell Carcinoma	Normal lung tissue	Smoker
TCGA-77-7338-11A	64	male	Lung Squamous Cell Carcinoma	Normal lung tissue	Smoker
TCGA-77-7138-11A	67	male	Lung Squamous Cell Carcinoma	Normal lung tissue	Smoker
TCGA-22-5471-11A	75	male	Lung Squamous Cell Carcinoma	Normal lung tissue	Smoker
TCGA-56-7823-11A	58	female	Lung Squamous Cell Carcinoma	Normal lung tissue	Smoker
TCGA-77-7337-11A	65	male	Lung Squamous Cell Carcinoma	Normal lung tissue	Smoker
TCGA-60-2709-11A	69	male	Lung Squamous Cell Carcinoma	Normal lung tissue	Smoker
TCGA-58-8386-11A	75	male	Lung Squamous Cell Carcinoma	Normal lung tissue	Smoker
TCGA-22-4609-11A	81	male	Lung Squamous Cell Carcinoma	Normal lung tissue	Smoker
TCGA-22-5489-11A	64	male	Lung Squamous Cell Carcinoma	Normal lung tissue	Smoker
TCGA-51-4081-11A	55	male	Lung Squamous Cell Carcinoma	Normal lung tissue	Smoker
TCGA-22-4593-11A	77	male	Lung Squamous Cell Carcinoma	Normal lung tissue	Smoker
TCGA-22-5481-11A	72	female	Lung Squamous Cell Carcinoma	Normal lung tissue	Smoker
TCGA-33-4587-11A	63	female	Lung Squamous Cell Carcinoma	Normal lung tissue	Smoker
TCGA-43-7658-11A	75	female	Lung Squamous Cell Carcinoma	Normal lung tissue	Smoker
TCGA-77-8008-11A	68	male	Lung Squamous Cell Carcinoma	Normal lung tissue	Smoker
TCGA-56-8201-11A	74	male	Lung Squamous Cell Carcinoma	Normal lung tissue	Smoker
TCGA-56-8309-11A	66	male	Lung Squamous Cell Carcinoma	Normal lung tissue	Smoker
TCGA-43-6647-11A	69	female	Lung Squamous Cell Carcinoma	Normal lung tissue	Smoker
TCGA-56-7579-11A	61	male	Lung Squamous Cell Carcinoma	Normal lung tissue	Smoker
TCGA-77-7335-11A	62	female	Lung Squamous Cell Carcinoma	Normal lung tissue	Smoker
TCGA-85-7710-11A	59	female	Lung Squamous Cell Carcinoma	Normal lung tissue	Smoker
TCGA-56-8623-11A	71	male	Lung Squamous Cell Carcinoma	Normal lung tissue	Smoker
TCGA-22-5482-11A	81	male	Lung Squamous Cell Carcinoma	Normal lung tissue	Smoker
TCGA-43-5670-11A	70	male	Lung Squamous Cell Carcinoma	Normal lung tissue	Smoker
TCGA-33-6737-11A	71	male	Lung Squamous Cell Carcinoma	Normal lung tissue	Smoker
TCGA-39-5040-11A	59	male	Lung Squamous Cell Carcinoma	Normal lung tissue	Smoker
TCGA-56-7582-11A	83	male	Lung Squamous Cell Carcinoma	Normal lung tissue	Smoker
TCGA-90-6837-11A	64	male	Lung Squamous Cell Carcinoma	Normal lung tissue	Smoker
TCGA-22-5491-11A	74	male	Lung Squamous Cell Carcinoma	Normal lung tissue	Smoker
TCGA-43-6773-11A	76	male	Lung Squamous Cell Carcinoma	Normal lung tissue	Smoker

TCGA-92-7340-11A	45	female	Lung Squamous Cell Carcinoma	Normal lung tissue	Smoker
TCGA-56-8082-11A	80	female	Lung Squamous Cell Carcinoma	Normal lung tissue	Smoker
TCGA-43-7657-11A	68	female	Lung Squamous Cell Carcinoma	Normal lung tissue	Smoker
TCGA-43-6771-11A	85	male	Lung Squamous Cell Carcinoma	Normal lung tissue	Smoker
TCGA-56-8083-11A	56	male	Lung Squamous Cell Carcinoma	Normal lung tissue	Smoker
TCGA-90-7767-11A	56	male	Lung Squamous Cell Carcinoma	Normal lung tissue	Smoker
TCGA-22-5478-11A	79	male	Lung Squamous Cell Carcinoma	Normal lung tissue	Smoker
TCGA-22-5483-11A	74	male	Lung Squamous Cell Carcinoma	Normal lung tissue	Smoker
TCGA-56-7580-11A	84	male	Lung Squamous Cell Carcinoma	Normal lung tissue	Smoker
TCGA-56-7222-11A	60	male	Lung Squamous Cell Carcinoma	Normal lung tissue	Smoker
TCGA-56-7731-11A	66	female	Lung Squamous Cell Carcinoma	Normal lung tissue	Smoker
TCGA-77-8007-11A	68	male	Lung Squamous Cell Carcinoma	Normal lung tissue	Smoker
TCGA-51-4079-11A	73	female	Lung Squamous Cell Carcinoma	Normal lung tissue	Smoker
TCGA-56-7730-11A	73	male	Lung Squamous Cell Carcinoma	Normal lung tissue	Smoker

Human validation set

Sample	Age	Sex	Disease	Histology of lung tissue	Tobacco smoke history
GSM1828889	59	male	Lung adenocarcinoma	Normal lung tissue	Smoker
GSM1828890	53	female	Lung adenocarcinoma	Normal lung tissue	Smoker
GSM1828891	73	female	Lung adenocarcinoma	Normal lung tissue	Smoker
GSM1828892	69	male	Lung adenocarcinoma	Normal lung tissue	Smoker
GSM1828893	65	female	Lung adenocarcinoma	Normal lung tissue	Smoker
GSM1828894	51	female	Lung adenocarcinoma	Normal lung tissue	Smoker
GSM1828895	40	female	Lung adenocarcinoma	Normal lung tissue	Smoker
GSM1828896	56	female	Lung adenocarcinoma	Normal lung tissue	Smoker
GSM1828897	53	male	Lung adenocarcinoma	Normal lung tissue	Smoker
GSM1828898	66	male	Lung adenocarcinoma	Normal lung tissue	Smoker
GSM1828899	67	female	Lung adenocarcinoma	Normal lung tissue	Smoker
GSM1828900	68	male	Lung adenocarcinoma	Normal lung tissue	Smoker
GSM1828901	62	male	Lung adenocarcinoma	Normal lung tissue	Smoker
GSM1828902	55	female	Lung adenocarcinoma	Normal lung tissue	Smoker
GSM1828903	66	male	Lung adenocarcinoma	Normal lung tissue	Smoker

GSM1828904	83	female	Lung adenocarcinoma	Normal lung tissue	Smoker
GSM1828905	79	male	Lung adenocarcinoma	Normal lung tissue	Smoker
GSM1828906	67	male	Lung adenocarcinoma	Normal lung tissue	Smoker
GSM1828907	64	male	Lung adenocarcinoma	Normal lung tissue	Smoker
GSM1828908	48	female	Lung adenocarcinoma	Normal lung tissue	Smoker
GSM1828909	68	male	Lung adenocarcinoma	Normal lung tissue	Smoker
GSM1828910	53	male	Lung adenocarcinoma	Normal lung tissue	Smoker
GSM1828911	60	male	Lung adenocarcinoma	Normal lung tissue	Smoker
GSM1828912	72	male	Lung adenocarcinoma	Normal lung tissue	Smoker
GSM1828913	68	female	Lung adenocarcinoma	Normal lung tissue	Smoker
GSM1828914	66	male	Lung adenocarcinoma	Normal lung tissue	Smoker
GSM1828915	42	male	Lung adenocarcinoma	Normal lung tissue	Smoker
GSM1828916	55	female	Lung adenocarcinoma	Normal lung tissue	Smoker
GSM1828917	79	male	Lung adenocarcinoma	Normal lung tissue	Smoker
GSM1828918	77	female	Lung adenocarcinoma	Normal lung tissue	Smoker
GSM1828919	54	female	Lung adenocarcinoma	Normal lung tissue	Smoker
GSM1828920	76	female	Lung adenocarcinoma	Normal lung tissue	Smoker
GSM1828921	59	male	Lung adenocarcinoma	Normal lung tissue	Smoker
GSM1828922	59	male	Lung adenocarcinoma	Normal lung tissue	Smoker
GSM1828923	59	male	Lung adenocarcinoma	Normal lung tissue	Smoker
GSM1828924	60	male	Lung adenocarcinoma	Normal lung tissue	Smoker
GSM1828925	72	male	Lung adenocarcinoma	Normal lung tissue	Smoker
GSM1828926	67	male	Lung adenocarcinoma	Normal lung tissue	Smoker
GSM1828927	71	male	Lung adenocarcinoma	Normal lung tissue	Smoker
GSM1828928	63	male	Lung adenocarcinoma	Normal lung tissue	Smoker
GSM1828929	69	male	Lung adenocarcinoma	Normal lung tissue	Smoker
GSM1828930	56	male	Lung adenocarcinoma	Normal lung tissue	Smoker
GSM1828931	51	female	Lung adenocarcinoma	Normal lung tissue	Smoker
GSM1828932	66	male	Lung adenocarcinoma	Normal lung tissue	Smoker
GSM1828933	57	male	Lung adenocarcinoma	Normal lung tissue	Smoker
GSM1828934	47	male	Lung adenocarcinoma	Normal lung tissue	Smoker
GSM1828935	77	male	Lung adenocarcinoma	Normal lung tissue	Smoker
GSM1828936	52	male	Lung adenocarcinoma	Normal lung tissue	Smoker

GSM1828937	72	male	Lung adenocarcinoma	Normal lung tissue	Smoker
GSM1828938	52	female	Lung adenocarcinoma	Normal lung tissue	Smoker
GSM1828939	73	female	Lung adenocarcinoma	Normal lung tissue	Smoker
GSM1828940	65	male	Lung adenocarcinoma	Normal lung tissue	Smoker
GSM1828941	57	female	Lung adenocarcinoma	Normal lung tissue	Smoker
GSM1828942	56	male	Lung adenocarcinoma	Normal lung tissue	Smoker
GSM1828943	80	female	Lung adenocarcinoma	Normal lung tissue	Smoker
GSM1828944	66	male	Lung adenocarcinoma	Normal lung tissue	Smoker
GSM1828945	78	male	Lung adenocarcinoma	Normal lung tissue	Smoker
GSM1828946	83	male	Lung adenocarcinoma	Normal lung tissue	Smoker
GSM1828947	71	male	Lung adenocarcinoma	Normal lung tissue	Smoker
GSM1828948	61	male	Lung adenocarcinoma	Normal lung tissue	Smoker
GSM1828949	67	male	Lung adenocarcinoma	Normal lung tissue	Smoker
GSM1828950	65	male	Lung adenocarcinoma	Normal lung tissue	Smoker
GSM1828951	74	female	Lung adenocarcinoma	Normal lung tissue	Smoker
GSM1828952	69	male	Lung adenocarcinoma	Normal lung tissue	Smoker
GSM1828953	74	male	Lung adenocarcinoma	Normal lung tissue	Smoker
GSM1828954	53	male	Lung adenocarcinoma	Normal lung tissue	Smoker
GSM1828955	63	male	Lung adenocarcinoma	Normal lung tissue	Smoker
GSM1828956	75	male	Lung adenocarcinoma	Normal lung tissue	Smoker
GSM1828957	49	male	Lung adenocarcinoma	Normal lung tissue	Smoker
GSM1828958	67	male	Lung adenocarcinoma	Normal lung tissue	Smoker
GSM1828959	67	male	Lung adenocarcinoma	Normal lung tissue	Smoker
GSM1828960	74	female	Lung adenocarcinoma	Normal lung tissue	Smoker
GSM1828961	74	male	Lung adenocarcinoma	Normal lung tissue	Smoker
GSM1828962	68	male	Lung adenocarcinoma	Normal lung tissue	Smoker
GSM1828963	55	female	Lung adenocarcinoma	Normal lung tissue	Smoker
GSM1828964	55	male	Lung adenocarcinoma	Normal lung tissue	Smoker
GSM1828965	60	male	Lung adenocarcinoma	Normal lung tissue	Smoker
GSM1828966	36	male	Lung adenocarcinoma	Normal lung tissue	Smoker
GSM1828967	64	female	Lung adenocarcinoma	Normal lung tissue	Smoker
GSM1828968	77	female	Lung adenocarcinoma	Normal lung tissue	Smoker
GSM1828969	66	male	Lung adenocarcinoma	Normal lung tissue	Smoker

GSM1828970	80	male	Lung adenocarcinoma	Normal lung tissue	Smoker
GSM1828971	74	male	Lung adenocarcinoma	Normal lung tissue	Smoker
GSM1828972	52	female	Lung adenocarcinoma	Normal lung tissue	Smoker
GSM1828973	80	male	Lung adenocarcinoma	Normal lung tissue	Smoker
GSM1828974	66	female	Lung adenocarcinoma	Normal lung tissue	Smoker
GSM1828975	70	female	Lung adenocarcinoma	Normal lung tissue	Smoker
GSM1828976	68	male	Lung adenocarcinoma	Normal lung tissue	Smoker
GSM1828977	63	male	Lung adenocarcinoma	Normal lung tissue	Smoker
GSM1828978	59	male	Lung adenocarcinoma	Normal lung tissue	Smoker
GSM1828979	80	male	Lung adenocarcinoma	Normal lung tissue	Smoker
GSM1828980	77	female	Lung adenocarcinoma	Normal lung tissue	Smoker
GSM1828981	68	female	Lung adenocarcinoma	Normal lung tissue	Smoker
GSM1828982	58	male	Lung adenocarcinoma	Normal lung tissue	Smoker
GSM1828983	60	male	Lung adenocarcinoma	Normal lung tissue	Smoker
GSM1828984	60	male	Lung adenocarcinoma	Normal lung tissue	Smoker
GSM1828985	75	male	Lung adenocarcinoma	Normal lung tissue	Smoker
GSM1828986	76	male	Lung adenocarcinoma	Normal lung tissue	Smoker
GSM1828987	55	male	Lung adenocarcinoma	Normal lung tissue	Smoker
GSM1828988	82	male	Lung adenocarcinoma	Normal lung tissue	Smoker
GSM1828989	61	male	Lung adenocarcinoma	Normal lung tissue	Smoker
GSM1828990	59	male	Lung adenocarcinoma	Normal lung tissue	Smoker
GSM1828991	77	male	Lung adenocarcinoma	Normal lung tissue	Smoker
GSM1828992	71	male	Lung adenocarcinoma	Normal lung tissue	Smoker
GSM1828993	71	female	Lung adenocarcinoma	Normal lung tissue	Smoker
GSM1828994	79	female	Lung adenocarcinoma	Normal lung tissue	Smoker
GSM1828995	52	female	Lung adenocarcinoma	Normal lung tissue	Smoker
GSM1828996	69	male	Lung adenocarcinoma	Normal lung tissue	Smoker
GSM1828997	51	female	Lung adenocarcinoma	Normal lung tissue	Smoker
GSM1828998	62	male	Lung adenocarcinoma	Normal lung tissue	Smoker
GSM1828999	83	female	Lung adenocarcinoma	Normal lung tissue	Smoker
GSM1829000	61	male	Lung adenocarcinoma	Normal lung tissue	Smoker
GSM1829001	63	male	Lung adenocarcinoma	Normal lung tissue	Smoker
GSM1829002	53	male	Lung adenocarcinoma	Normal lung tissue	Smoker

GSM1829003	46	male	Lung adenocarcinoma	Normal lung tissue	Smoker
GSM1829004	69	male	Lung adenocarcinoma	Normal lung tissue	Smoker
GSM1829005	69	male	Lung adenocarcinoma	Normal lung tissue	Smoker
GSM1829006	73	male	Lung adenocarcinoma	Normal lung tissue	Smoker
GSM1829007	52	male	Lung adenocarcinoma	Normal lung tissue	Smoker
GSM1829008	77	male	Lung adenocarcinoma	Normal lung tissue	Smoker
GSM1829009	55	female	Lung adenocarcinoma	Normal lung tissue	Smoker
GSM1829010	62	female	Lung adenocarcinoma	Normal lung tissue	Smoker
GSM1829011	58	male	Lung adenocarcinoma	Normal lung tissue	Smoker
GSM1829012	76	male	Lung adenocarcinoma	Normal lung tissue	Smoker
GSM1829013	74	male	Lung adenocarcinoma	Normal lung tissue	Smoker
GSM1829014	50	male	Lung adenocarcinoma	Normal lung tissue	Smoker
GSM1829015	74	male	Lung adenocarcinoma	Normal lung tissue	Smoker
GSM1829016	60	female	Lung adenocarcinoma	Normal lung tissue	Smoker
GSM1829017	70	male	Lung adenocarcinoma	Normal lung tissue	Smoker
GSM1829018	75	male	Lung adenocarcinoma	Normal lung tissue	Smoker
GSM1829019	59	male	Lung adenocarcinoma	Normal lung tissue	Smoker
GSM1829020	53	male	Lung adenocarcinoma	Normal lung tissue	Smoker
GSM1829021	56	male	Lung adenocarcinoma	Normal lung tissue	Smoker
GSM1829022	47	female	Lung adenocarcinoma	Normal lung tissue	Smoker
GSM1829023	63	male	Lung adenocarcinoma	Normal lung tissue	Smoker
GSM1829024	71	female	Lung adenocarcinoma	Normal lung tissue	Smoker
GSM1829025	52	female	Lung adenocarcinoma	Normal lung tissue	Smoker
GSM1829026	49	female	Lung adenocarcinoma	Normal lung tissue	Smoker
GSM1829027	57	female	Lung adenocarcinoma	Normal lung tissue	Smoker
GSM1829028	70	male	Lung adenocarcinoma	Normal lung tissue	Smoker
GSM1829029	59	female	Lung adenocarcinoma	Normal lung tissue	Smoker
GSM1829030	69	female	Lung adenocarcinoma	Normal lung tissue	Smoker
GSM1829031	73	male	Lung adenocarcinoma	Normal lung tissue	Smoker
GSM1829032	73	male	Lung adenocarcinoma	Normal lung tissue	Smoker
GSM1829033	66	female	Lung adenocarcinoma	Normal lung tissue	Smoker
GSM1829034	57	female	Lung adenocarcinoma	Normal lung tissue	Smoker
GSM1829035	81	female	Lung adenocarcinoma	Normal lung tissue	Smoker

GSM1829036	58	male	Lung adenocarcinoma	Normal lung tissue	Smoker
GSM1829037	62	female	Lung adenocarcinoma	Normal lung tissue	Smoker
GSM1829038	73	male	Lung adenocarcinoma	Normal lung tissue	Smoker
GSM1829039	66	male	Lung adenocarcinoma	Normal lung tissue	Smoker
GSM1829040	64	male	Lung adenocarcinoma	Normal lung tissue	Smoker
GSM1829041	58	male	Lung adenocarcinoma	Normal lung tissue	Smoker
GSM1829042	68	female	Lung adenocarcinoma	Normal lung tissue	Smoker
GSM1829043	62	female	Lung adenocarcinoma	Normal lung tissue	Smoker
GSM1829044	69	female	Lung adenocarcinoma	Normal lung tissue	Smoker
GSM1829045	67	male	Lung adenocarcinoma	Normal lung tissue	Smoker
GSM1829046	77	male	Lung adenocarcinoma	Normal lung tissue	Smoker
GSM1829047	60	male	Lung adenocarcinoma	Normal lung tissue	Smoker
GSM1829048	72	male	Lung adenocarcinoma	Normal lung tissue	Smoker
GSM1829049	55	female	Lung adenocarcinoma	Normal lung tissue	Smoker
GSM1829050	64	female	Lung adenocarcinoma	Normal lung tissue	Smoker
GSM1829051	79	male	Lung adenocarcinoma	Normal lung tissue	Smoker
GSM1829052	76	male	Lung adenocarcinoma	Normal lung tissue	Smoker
GSM1829053	78	male	Lung adenocarcinoma	Normal lung tissue	Smoker
GSM1829054	75	male	Lung adenocarcinoma	Normal lung tissue	Smoker
GSM1829055	44	female	Lung adenocarcinoma	Normal lung tissue	Smoker
GSM1829056	62	male	Lung adenocarcinoma	Normal lung tissue	Smoker
GSM1829057	72	male	Lung adenocarcinoma	Normal lung tissue	Smoker
GSM1829058	54	male	Lung adenocarcinoma	Normal lung tissue	Smoker
GSM1829059	75	female	Lung adenocarcinoma	Normal lung tissue	Smoker
GSM1829060	62	male	Lung adenocarcinoma	Normal lung tissue	Smoker
GSM1829061	56	male	Lung adenocarcinoma	Normal lung tissue	Smoker
GSM1829062	70	male	Lung adenocarcinoma	Normal lung tissue	Smoker
GSM1829063	48	female	Lung adenocarcinoma	Normal lung tissue	Smoker
GSM1829064	64	male	Lung adenocarcinoma	Normal lung tissue	Smoker
GSM1829065	69	male	Lung adenocarcinoma	Normal lung tissue	Smoker
GSM1829066	67	male	Lung adenocarcinoma	Normal lung tissue	Smoker
GSM1829067	69	male	Lung adenocarcinoma	Normal lung tissue	Smoker
GSM1829068	69	female	Lung adenocarcinoma	Normal lung tissue	Smoker

GSM1829069	56	female	Lung adenocarcinoma	Normal lung tissue	Smoker
GSM1829070	67	male	Lung adenocarcinoma	Normal lung tissue	Smoker
GSM1829071	80	female	Lung adenocarcinoma	Normal lung tissue	Smoker
GSM1829072	72	male	Lung adenocarcinoma	Normal lung tissue	Smoker
GSM1829073	49	female	Lung adenocarcinoma	Normal lung tissue	Smoker
GSM1829074	65	male	Lung adenocarcinoma	Normal lung tissue	Smoker
GSM1829075	60	male	Lung adenocarcinoma	Normal lung tissue	Smoker
GSM1829076	54	male	Lung adenocarcinoma	Normal lung tissue	Smoker
GSM1829077	74	male	Lung adenocarcinoma	Normal lung tissue	Smoker
GSM1829078	67	male	Lung adenocarcinoma	Normal lung tissue	Smoker
GSM1829079	62	male	Lung adenocarcinoma	Normal lung tissue	Smoker
GSM1829080	68	female	Lung adenocarcinoma	Normal lung tissue	Smoker
GSM1829081	54	male	Lung adenocarcinoma	Normal lung tissue	Smoker
GSM1829082	74	male	Lung adenocarcinoma	Normal lung tissue	Smoker
GSM1829083	77	male	Lung adenocarcinoma	Normal lung tissue	Smoker
GSM1829084	60	male	Lung adenocarcinoma	Normal lung tissue	Smoker
GSM1829085	65	female	Lung adenocarcinoma	Normal lung tissue	Smoker
GSM1829086	56	female	Lung adenocarcinoma	Normal lung tissue	Smoker
GSM1829087	56	male	Lung adenocarcinoma	Normal lung tissue	Smoker
GSM1829088	71	male	Lung adenocarcinoma	Normal lung tissue	Smoker
GSM1829089	70	male	Lung adenocarcinoma	Normal lung tissue	Smoker
GSM1829090	47	male	Lung adenocarcinoma	Normal lung tissue	Non-Smoker
GSM1829091	71	male	Lung adenocarcinoma	Normal lung tissue	Smoker
GSM1829092	68	male	Lung adenocarcinoma	Normal lung tissue	Smoker
GSM1829093	48	female	Lung adenocarcinoma	Normal lung tissue	Smoker
GSM1829094	62	female	Lung adenocarcinoma	Normal lung tissue	Smoker
GSM1829095	51	female	Lung adenocarcinoma	Normal lung tissue	Smoker
GSM1829096	53	female	Lung adenocarcinoma	Normal lung tissue	Smoker
GSM1829097	70	male	Lung adenocarcinoma	Normal lung tissue	Smoker
GSM1829098	62	male	Lung adenocarcinoma	Normal lung tissue	Smoker
GSM1829099	58	male	Lung adenocarcinoma	Normal lung tissue	Smoker
GSM1829100	49	male	Lung adenocarcinoma	Normal lung tissue	Smoker
GSM1829101	72	male	Lung adenocarcinoma	Normal lung tissue	Smoker

GSM1829102	76	male	Lung adenocarcinoma	Normal lung tissue	Smoker
GSM1829103	74	male	Lung adenocarcinoma	Normal lung tissue	Smoker
GSM1829104	60	male	Lung adenocarcinoma	Normal lung tissue	Smoker
GSM1829105	58	female	Lung adenocarcinoma	Normal lung tissue	Smoker
GSM1829106	54	male	Lung adenocarcinoma	Normal lung tissue	Smoker
GSM1829107	60	male	Lung adenocarcinoma	Normal lung tissue	Smoker
GSM1829108	63	male	Lung adenocarcinoma	Normal lung tissue	Smoker
GSM1829109	79	male	Lung adenocarcinoma	Normal lung tissue	Smoker
GSM1829110	83	male	Lung adenocarcinoma	Normal lung tissue	Smoker
GSM1829111	85	male	Lung adenocarcinoma	Normal lung tissue	Smoker
GSM1829112	75	female	Lung adenocarcinoma	Normal lung tissue	Smoker
GSM1829113	67	female	Lung adenocarcinoma	Normal lung tissue	Smoker
GSM1829114	59	female	Lung adenocarcinoma	Normal lung tissue	Smoker
GSM1829115	61	male	Lung adenocarcinoma	Normal lung tissue	Smoker
GSM1829116	69	male	Lung adenocarcinoma	Normal lung tissue	Smoker
GSM1829117	60	male	Lung adenocarcinoma	Normal lung tissue	Smoker
GSM1829118	71	male	Lung adenocarcinoma	Normal lung tissue	Smoker
GSM1829119	69	male	Lung adenocarcinoma	Normal lung tissue	Smoker
GSM1829120	60	male	Lung adenocarcinoma	Normal lung tissue	Smoker
GSM1829121	67	male	Lung adenocarcinoma	Normal lung tissue	Smoker
GSM1829122	58	female	Lung adenocarcinoma	Normal lung tissue	Smoker
GSM1829123	57	male	Lung adenocarcinoma	Normal lung tissue	Smoker
GSM1829124	55	female	Lung adenocarcinoma	Normal lung tissue	Smoker
GSM1829125	72	male	Lung adenocarcinoma	Normal lung tissue	Smoker
GSM1829126	70	male	Lung adenocarcinoma	Normal lung tissue	Smoker
GSM1829127	73	male	Lung adenocarcinoma	Normal lung tissue	Smoker
GSM1829128	59	male	Lung adenocarcinoma	Normal lung tissue	Smoker
GSM1829129	73	male	Lung adenocarcinoma	Normal lung tissue	Smoker
GSM1829130	68	female	Lung adenocarcinoma	Normal lung tissue	Smoker
GSM1829131	67	male	Lung adenocarcinoma	Normal lung tissue	Smoker
GSM1829132	69	male	Lung adenocarcinoma	Normal lung tissue	Smoker
GSM1829133	59	male	Lung adenocarcinoma	Normal lung tissue	Smoker
GSM1829134	73	male	Lung adenocarcinoma	Normal lung tissue	Smoker

GSM1829135	74	male	Lung adenocarcinoma	Normal lung tissue	Smoker
GSM1829136	57	female	Lung adenocarcinoma	Normal lung tissue	Smoker
GSM1829137	75	male	Lung adenocarcinoma	Normal lung tissue	Smoker
GSM1829138	60	male	Lung adenocarcinoma	Normal lung tissue	Smoker
GSM1829139	63	male	Lung adenocarcinoma	Normal lung tissue	Smoker
GSM1829140	53	male	Lung adenocarcinoma	Normal lung tissue	Smoker
GSM1829141	65	male	Lung adenocarcinoma	Normal lung tissue	Smoker
GSM1829142	75	male	Lung adenocarcinoma	Normal lung tissue	Smoker
GSM1829143	60	male	Lung adenocarcinoma	Normal lung tissue	Smoker
GSM1829144	71	male	Lung adenocarcinoma	Normal lung tissue	Smoker
GSM1829145	67	female	Lung adenocarcinoma	Normal lung tissue	Smoker
GSM1829146	65	female	Lung adenocarcinoma	Normal lung tissue	Smoker
GSM1829147	68	male	Lung adenocarcinoma	Normal lung tissue	Smoker
GSM1829148	63	female	Lung adenocarcinoma	Normal lung tissue	Smoker
GSM1829149	77	male	Lung adenocarcinoma	Normal lung tissue	Smoker
GSM1829150	61	female	Lung adenocarcinoma	Normal lung tissue	Smoker
GSM1829151	66	male	Lung adenocarcinoma	Normal lung tissue	Smoker
GSM1829152	71	male	Lung adenocarcinoma	Normal lung tissue	Smoker
GSM1829153	56	male	Lung adenocarcinoma	Normal lung tissue	Smoker
GSM1829154	73	male	Lung adenocarcinoma	Normal lung tissue	Smoker
GSM1829155	71	male	Lung adenocarcinoma	Normal lung tissue	Smoker
GSM1829156	68	male	Lung adenocarcinoma	Normal lung tissue	Smoker
GSM1829157	60	male	Lung adenocarcinoma	Normal lung tissue	Smoker
GSM1829158	73	male	Lung adenocarcinoma	Normal lung tissue	Smoker
GSM1829159	61	male	Lung adenocarcinoma	Normal lung tissue	Smoker
GSM1829160	55	female	Lung adenocarcinoma	Normal lung tissue	Smoker
GSM1829161	73	male	Lung adenocarcinoma	Normal lung tissue	Smoker
GSM1829162	54	male	Lung adenocarcinoma	Normal lung tissue	Smoker
GSM1829163	54	female	Lung adenocarcinoma	Normal lung tissue	Smoker
GSM1829164	68	male	Lung adenocarcinoma	Normal lung tissue	Smoker
GSM1829165	67	male	Lung adenocarcinoma	Normal lung tissue	Smoker
GSM1829166	68	male	Lung adenocarcinoma	Normal lung tissue	Smoker
GSM1829167	72	male	Lung adenocarcinoma	Normal lung tissue	Smoker

GSM1829168	69	male	Lung adenocarcinoma	Normal lung tissue	Smoker
GSM1829169	58	male	Lung adenocarcinoma	Normal lung tissue	Smoker
GSM1829170	65	male	Lung adenocarcinoma	Normal lung tissue	Smoker
GSM1829171	72	male	Lung adenocarcinoma	Normal lung tissue	Smoker
GSM1829172	71	male	Lung adenocarcinoma	Normal lung tissue	Smoker
GSM27778	58	Male	cerebrovascular accident	Normal lung tissue	Non-Smoker
GSM27779	63	Female	cerebrovascular accident	Normal lung tissue	Smoker
GSM27781	31	Male	Head trauma	Normal lung tissue	Non-Smoker
GSM27783	48	Female	Hydrocephalus	Normal lung tissue	Non-Smoker
GSM27785	64	Male	intracranial hemorrhage	Normal lung tissue	Smoker
GSM27786	24	Male	Head trauma	Normal lung tissue	Non-Smoker
GSM27788	63	Female	cerebrovascular accident	Normal lung tissue	Smoker
GSM27789	71	Male	cerebrovascular accident	Normal lung tissue	Smoker
GSM27791	23	Male	Head trauma	Normal lung tissue	Smoker
GSM27793	21	Male	Head trauma	Normal lung tissue	Smoker
GSM27794	47	Male	Head trauma	Normal lung tissue	Smoker
GSM27795	74	Female	cerebrovascular accident	Normal lung tissue	Non-Smoker
GSM27796	64	Male	cerebrovascular accident	Normal lung tissue	Non-Smoker
GSM27797	71	Female	cerebrovascular accident	Normal lung tissue	Smoker
GSM27798	26	Female	Head trauma	Normal lung tissue	Non-Smoker
GSM27799	53	Female	cerebrovascular accident	Normal lung tissue	Smoker
GSM27800	54	Female	Head trauma	Normal lung tissue	Non-Smoker
GSM27825	50	Female	cerebrovascular accident	Normal lung tissue	Smoker
GSM27826	38	Male	CNS tumor	Normal lung tissue	Smoker
GSM27827	38	Female	cerebrovascular accident	Normal lung tissue	Smoker
GSM27828	46	Female	cerebrovascular accident	Normal lung tissue	Smoker
GSM27829	69	Male	Anoxia	Normal lung tissue	Smoker
GSM27831	59	Female	intracranial hemorrhage	Normal lung tissue	Smoker

Supplementary Table S3
Gene set enrichment analysis of the mouse genomic data

Week 1-5~6-26

Description	setSize	enrichmentScore	NES	pvalue	p.adjust
Cell Adhesion	39	0.27	1.09	0.33	0.48
Cell Division	36	0.34	1.37	0.08	0.24
Collagen Fibril Organization	32	0.48	1.90	0.00	0.06
Epithelial To Mesenchymal Transition	26	0.42	1.57	0.02	0.14
Branching Morphogenesis Of A Tube	33	0.35	1.35	0.07	0.22
Keratinization	23	0.32	1.12	0.31	0.46
Morphogenesis Of An Epithelium	22	0.51	1.75	0.01	0.08
Extracellular Matrix Structural Constituent	26	0.70	2.57	0.00	0.06

Week 6-26~52-130

Description	setSize	enrichmentScore	NES	pvalue	p.adjust
Blood Vessel Development	40	0.61	2.21	0.00	0.06
Basal Lamina	18	0.68	2.01	0.00	0.06
Cellular Senescence	13	0.50	1.37	0.15	0.35
Positive Regulation Of Autophagy	11	0.67	1.72	0.01	0.08
Transcriptional Repressor Complex	37	0.55	1.95	0.00	0.06
Integrin Binding	40	0.39	1.40	0.06	0.20
Cellular Response To Vascular Endothelial Growth Factor Stimulus	13	0.67	1.82	0.01	0.07
Positive Regulation Of B Cell Proliferation	33	0.39	1.36	0.09	0.26
Scavenger Receptor Activity	35	-0.47	-1.61	0.02	0.14
Acute-Phase Response	29	-0.40	-1.31	0.13	0.33
B Cell Activation	25	-0.50	-1.57	0.03	0.16
Extracellular Space	27	-0.48	-1.52	0.05	0.19
Fatty Acid Transport	12	-0.42	-1.08	0.38	0.53
Antigen Processing And Presentation Of Exogenous Peptide Antigen Via Mhc Class Ii	14	-0.43	-1.16	0.29	0.47
Platelet Activation	28	-0.40	-1.27	0.16	0.36

Supplementary Table S4

Linear regression model defined 77 up- and 13 down-regulated genes whose expression changed with age

Upregulated genes from linear regression model

Probes	Genes	Full names	Estimate	Std. Error	p-value	R2
1424631_a_at	Ighg	Immunoglobulin heavy chain (gamma polypeptide)	14.36	1.23	0.00	0.67
1424305_at	Jchain	immunoglobulin joining chain	13.09	1.34	0.00	0.59
1425385_a_at	Ighm	immunoglobulin heavy constant mu	13.63	1.37	0.00	0.60
1425738_at	Igk	immunoglobulin kappa chain complex	13.73	1.27	0.00	0.64
1451962_at	Igkv6-20	immunoglobulin kappa variable 6-20	15.58	1.38	0.00	0.66
1460423_x_at	Igkv9-120	immunoglobulin kappa chain variable 9-120	12.82	1.81	0.00	0.43
1424931_s_at	Iglc1	immunoglobulin lambda constant 1	13.27	1.79	0.00	0.45
1435792_at	LOC100503923	proteinase-activated receptor 1-like	18.92	1.69	0.00	0.66
1430523_s_at	Iglv1	immunoglobulin lambda variable 1	13.89	1.90	0.00	0.45
1452463_x_at	Igkv8-30	immunoglobulin kappa chain variable 8-30	17.89	1.76	0.00	0.61
1427455_x_at	Igkv6-23	immunoglobulin kappa variable 6-23	18.09	1.95	0.00	0.56
1427860_at	Igkv6-14	immunoglobulin kappa variable 6-14	19.11	2.33	0.00	0.51
1419684_at	Ccl8	chemokine (C-C motif) ligand 8	21.12	2.42	0.00	0.54
1417851_at	Cxcl13	chemokine (C-X-C motif) ligand 13	21.14	2.31	0.00	0.56
1424509_at	Cd177	CD177 antigen	19.30	2.78	0.00	0.42
1435660_at	LOC664787	Sp110 nuclear body protein pseudogene	31.20	2.39	0.00	0.72
1428947_at	Mzb1	marginal zone B and B1 cell-specific protein 1	21.52	2.65	0.00	0.50
1425078_x_at	LOC102638047	sp110 nuclear body protein-like	28.48	2.35	0.00	0.69
1427221_at	Slc6a20a	solute carrier family 6 (neurotransmitter transporter), member 20A	24.81	2.96	0.00	0.52
1427851_x_at	Ighv1-72	immunoglobulin heavy variable 1-72	22.12	3.20	0.00	0.42
1420330_at	Clec4e	C-type lectin domain family 4, member e	21.53	2.49	0.00	0.53
1422140_at	LOC100503923	proteinase-activated receptor 1-like	35.69	3.01	0.00	0.68
1427577_x_at	Igkv6-14	immunoglobulin kappa variable 6-14	23.71	3.12	0.00	0.47
1436530_at	Wfdc17	WAP four-disulfide core domain 17	25.61	2.80	0.00	0.56
1422188_s_at	Tcrg-V4	T cell receptor gamma, variable 4	26.83	3.47	0.00	0.48
1437052_s_at	Slc2a3	solute carrier family 2 (facilitated glucose transporter), member 3	23.75	3.30	0.00	0.44
1424923_at	Serpina3g	serine (or cysteine) peptidase inhibitor, clade A, member 3G	21.07	3.07	0.00	0.42

1449399_a_at	Il1b	interleukin 1 beta	24.49	3.08	0.00	0.49
1430700_a_at	Pla2g7	phospholipase A2, group VII (platelet-activating factor acetylhydrolase, plasma)	28.14	3.71	0.00	0.47
1417601_at	Rgs1	regulator of G-protein signaling 1	25.35	3.70	0.00	0.42
1449856_at	Rgs18	regulator of G-protein signaling 18	23.53	3.38	0.00	0.42
1420804_s_at	Clec4d	C-type lectin domain family 4, member d	20.39	3.03	0.00	0.41
1427503_at	Ighg3	Immunoglobulin heavy constant gamma 3	36.52	4.17	0.00	0.54
1427857_x_at	Igkv4-68	immunoglobulin kappa variable 4-68	39.04	4.16	0.00	0.57
1425832_a_at	Cxcr6	chemokine (C-X-C motif) receptor 6	25.88	3.87	0.00	0.40
1445882_at	Cd300lb	CD300 molecule like family member B	25.30	3.45	0.00	0.45
1418531_at	Oosp1	oocyte secreted protein 1	30.39	3.88	0.00	0.48
1422280_at	Gzmk	granzyme K	41.65	3.58	0.00	0.67
1442339_at	Stfa2l1	stefin A2 like 1	23.81	3.17	0.00	0.46
1417936_at	Ccl9	chemokine (C-C motif) ligand 9	34.62	4.33	0.00	0.49
1455882_x_at	Vwc2	von Willebrand factor C domain containing 2	29.09	3.98	0.00	0.45
1426201_at	Igkv4-72	immunoglobulin kappa chain variable 4-72	36.30	4.07	0.00	0.55
1454157_a_at	Pla2g2d	phospholipase A2, group IID	31.92	4.43	0.00	0.44
1460416_s_at	LOC101055758	component of Sp100-rs-like	34.04	4.73	0.00	0.44
1449984_at	Cxcl2	chemokine (C-X-C motif) ligand 2	33.16	3.99	0.00	0.51
1435710_at	AI661384	expressed sequence AI661384	30.04	4.20	0.00	0.44
1449193_at	Cd5l	CD5 antigen-like	32.23	4.33	0.00	0.46
1436779_at	Cybb	cytochrome b-245, beta polypeptide	29.42	4.21	0.00	0.43
1458504_at	Zc3h12d	zinc finger CCCH type containing 12D	30.65	4.35	0.00	0.43
1448291_at	Mmp9	matrix metallopeptidase 9	28.78	4.15	0.00	0.42
1420361_at	Slc11a1	solute carrier family 11 (proton-coupled divalent metal ion transporters), member 1	30.50	4.57	0.00	0.40
1421098_at	Stap1	signal transducing adaptor family member 1	34.86	5.25	0.00	0.40
1448575_at	Il7r	interleukin 7 receptor	32.91	4.84	0.00	0.41
1454171_x_at	9530053H05Rik	RIKEN cDNA 9530053H05 gene	33.54	4.55	0.00	0.45
1450241_a_at	Evi2a	ecotropic viral integration site 2a	35.11	5.03	0.00	0.42
1418243_at	Fcna	ficolin A	37.06	5.21	0.00	0.43
1436861_at	Il7	interleukin 7	42.20	5.55	0.00	0.47
1460273_a_at	Naip2	NLR family, apoptosis inhibitory protein 2	37.95	5.00	0.00	0.47

1433930_at	Hpse	heparanase	40.03	5.59	0.00	0.44
1444962_at	Gm34940	predicted gene, 34940	37.86	5.31	0.00	0.44
1435353_a_at	Pisd-ps1	phosphatidylserine decarboxylase, pseudogene 1	40.28	5.46	0.00	0.45
1435144_at	Fyb	FYN binding protein	38.46	5.16	0.00	0.46
1457753_at	Tlr13	toll-like receptor 13	30.64	4.62	0.00	0.40
1422189_x_at	Tcrg-V4	T cell receptor gamma, variable 4	41.39	5.84	0.00	0.43
1418465_at	Ncf4	neutrophil cytosolic factor 4	31.76	4.40	0.00	0.44
1430373_at	5430427O19Rik	RIKEN cDNA 5430427O19 gene	40.52	5.44	0.00	0.46
1422046_at	Itgam	integrin alpha M	32.62	4.71	0.00	0.42
1424832_at	Cd300ld	CD300 molecule like family member d	41.57	5.35	0.00	0.48
1439069_a_at	Pisd-ps3	phosphatidylserine decarboxylase, pseudogene 3	50.65	6.58	0.00	0.47
1419202_at	Cst7	cystatin F (leukocystatin)	48.83	6.11	0.00	0.49
1456635_at	Gm32088	predicted gene, 32088	43.52	6.40	0.00	0.41
1455031_at	Cdk19	cyclin-dependent kinase 19	47.52	6.67	0.00	0.43
1460271_at	Trem3	triggering receptor expressed on myeloid cells 3	39.51	5.84	0.00	0.41
1418980_a_at	Cnp	2',3'-cyclic nucleotide 3' phosphodiesterase	59.86	8.27	0.00	0.44
1449925_at	Cxcr3	chemokine (C-X-C motif) receptor 3	54.71	7.76	0.00	0.43
1432459_a_at	Zbtb32	zinc finger and BTB domain containing 32	60.30	8.79	0.00	0.42
1435945_a_at	Kcnn4	potassium intermediate	77.62	11.14	0.00	0.42

Downregulated genes from linear regression model

probes	genes	Full names	Estimate	Std. Error	p-value	R2
1427986_a_at	Col16a1	collagen, type XVI, alpha 1	-71.18	10.00	0.00	0.43
1416498_at	Ppic	peptidylprolyl isomerase C	-81.37	9.32	0.00	0.54
1423854_a_at	Rasl11b	RAS-like, family 11, member B	-61.02	8.93	0.00	0.41
1438312_s_at	Ltbp3	latent transforming growth factor beta binding protein 3	-54.29	7.78	0.00	0.42
1418670_s_at	Hspg2	perlecan (heparan sulfate proteoglycan 2)	-43.82	6.18	0.00	0.43
1423885_at	Lamc1	laminin, gamma 1	-44.32	5.99	0.00	0.45
1458341_x_at	Pde5a	phosphodiesterase 5A, cGMP-specific	-44.06	6.15	0.00	0.44
1416623_at	Thbs3	thrombospondin 3	-45.47	5.73	0.00	0.49
1435990_at	Adamts2	rin-like and metallopeptidase (reprolysin type) with thrombospondin type	-34.38	4.50	0.00	0.47
1427883_a_at	Col3a1	collagen, type III, alpha 1	-36.10	4.16	0.00	0.53
1423594_a_at	Ednrb	endothelin receptor type B	-29.34	3.72	0.00	0.49

1450839_at	Nrep	neuronal regeneration related protein	-37.54	3.31	0.00	0.66
1438651_a_at	Aplnr	apelin receptor	-25.87	3.29	0.00	0.48

Supplementary Table S5
Regulation of ECM coding genes in mice

Genes	Full names	Week 1-5	Week 6-26	Week 52-130
**Lrg1**	leucine-rich alpha-2-glycoprotein 1	9.03	9.97	10.74
**Adipoq**	adiponectin, C1Q and collagen domain containing	6.81	8.48	9.87
**Fgg**	fibrinogen gamma chain	5.11	5.95	6.89
**Lgals3**	lectin, galactose binding, soluble 3	10.40	11.48	12.07
**Dcn**	decorin	10.22	11.39	12.21
**Col8a2**	collagen, type VIII, alpha 2	5.58	6.61	7.63
**Fgl2**	fibrinogen-like protein 2	8.07	8.72	9.61
**Timp4**	tissue inhibitor of metalloproteinase 4	6.34	6.26	7.19
**Chad**	chondroadherin	8.30	9.09	9.93
**Anxa8**	annexin A8	7.69	8.49	9.24
**Col9a2**	collagen, type IX, alpha 2	4.76	4.79	5.42
**Omd**	osteomodulin	6.72	7.24	8.14
**Fga**	fibrinogen alpha chain	4.39	4.76	5.86
**Prg2**	proteoglycan 2, bone marrow	4.71	4.94	5.47
**Mmp9**	matrix metallopeptidase 9	6.10	6.54	7.54
**Mmp12**	matrix metallopeptidase 12	5.88	6.07	6.99
**Mmp8**	matrix metallopeptidase 8	6.06	6.48	7.17
**Emilin2**	elastin microfibril interfacer 2	7.47	7.74	8.62
**Mmrn1**	multimerin 1	7.82	7.85	8.91
**Mmp3**	matrix metallopeptidase 3	6.31	8.57	8.52
**Col8a1**	collagen, type VIII, alpha 1	6.80	7.53	7.99
**Anxa7**	annexin A7	7.80	8.55	8.68
**Vwa5a**	von Willebrand factor A domain containing 5A	9.34	10.06	10.28
**Serpinb6a**	serine (or cysteine) peptidase inhibitor, clade B, member 6a	10.58	11.34	11.55
**Spp1**	secreted phosphoprotein 1	8.35	10.25	10.91
**Ecm1**	extracellular matrix protein 1	9.21	10.01	10.28
**Timp3**	tissue inhibitor of metalloproteinase 3	11.49	11.97	12.34

**Aebp1**	AE binding protein 1	8.46	8.83	9.63
**Col27a1**	collagen, type XXVII, alpha 1	8.53	7.54	8.32
**Vcan**	versican	4.34	3.91	4.33
**Col15a1**	collagen, type XV, alpha 1	7.88	7.06	7.55
**Col2a1**	collagen, type II, alpha 1	7.12	6.24	7.20
**Col6a4**	collagen, type VI, alpha 4	6.95	5.99	6.25
**Col9a1**	collagen, type IX, alpha 1	4.61	4.06	5.08
**Col5a3**	collagen, type V, alpha 3	6.47	6.30	6.56
**Col11a1**	collagen, type XI, alpha 1	5.57	4.96	5.84
**Mmp16**	matrix metallopeptidase 16	5.43	4.88	5.25
**Col10a1**	collagen, type X, alpha 1	6.42	5.79	6.89
**Col11a2**	collagen, type XI, alpha 2	6.57	5.67	6.15
**Fmod**	fibromodulin	7.75	7.31	8.19
**Col25a1**	collagen, type XXV, alpha 1	5.90	4.32	4.77
**Tgfbi**	transforming growth factor, beta induced	10.04	8.93	9.50
**Hapln1**	hyaluronan and proteoglycan link protein 1	4.06	3.53	4.41
**Fbln2**	fibulin 2	8.25	7.32	7.83
**Fgf14**	fibroblast growth factor 14	5.37	4.78	4.94
**Igfbp4**	insulin-like growth factor binding protein 4	12.16	11.60	11.84
**Fn1**	fibronectin 1	12.43	11.21	11.40
**Fbn2**	fibrillin 2	6.14	4.53	4.71
**Adamts18**	a disintegrin-like and metallopeptidase (reprolysin type) with thrombospondin type 1 motif, 18	4.99	4.33	4.45
**Mmp24**	matrix metallopeptidase 24	7.48	7.00	7.06
**Igfals**	insulin-like growth factor binding protein, acid labile subunit	7.51	6.94	7.16
**Adamts16**	a disintegrin-like and metallopeptidase (reprolysin type) with thrombospondin type 1 motif, 16	7.66	6.98	7.21
**Adamts4**	a disintegrin-like and metallopeptidase (reprolysin type) with thrombospondin type 1 motif, 4	5.77	5.08	5.20
**Elane**	elastase, neutrophil expressed	6.68	5.99	6.15
**Aspn**	asporin	8.47	7.25	7.46
**Col6a1**	collagen, type VI, alpha 1	11.61	10.64	10.75

**Ntn3**	netrin 3	5.65	5.00	5.00
**Anxa13**	annexin A13	4.89	4.87	5.58
**Mmp13**	matrix metallopeptidase 13	4.04	3.97	5.19
**Dmbt1**	deleted in malignant brain tumors 1	4.87	4.74	6.42
**Prg4**	proteoglycan 4 (megakaryocyte stimulating factor, articular superficial zone protein)	5.74	5.65	7.93
**Acan**	aggrecan	6.47	6.35	7.28
**Thbs4**	thrombospondin 4	5.74	5.62	6.44
**Eln**	elastin	12.28	9.93	9.34
**Lama1**	laminin, alpha 1	5.74	5.44	5.19
**Mfap4**	microfibrillar-associated protein 4	12.79	11.58	10.94
**Mfap2**	microfibrillar-associated protein 2	10.96	10.07	9.63
**Fbn1**	fibrillin 1	10.63	9.27	8.68
**Hspg2**	perlecan (heparan sulfate proteoglycan 2)	9.08	8.92	8.39
**Col1a1**	collagen, type I, alpha 1	11.91	10.04	9.31
**Fgf4**	fibroblast growth factor 4	6.33	5.62	5.21
**Col5a1**	collagen, type V, alpha 1	8.48	7.21	6.87
**Col4a1**	collagen, type IV, alpha 1	13.27	12.26	11.52
**Tnc**	tenascin C	5.29	3.75	3.60
**Lamc1**	laminin, gamma 1	10.77	9.71	9.15
**Lama4**	laminin, alpha 4	8.43	6.94	5.68
**Nid1**	nidogen 1	9.88	8.66	7.94
**Ltbp2**	latent transforming growth factor beta binding protein 2	10.63	9.12	8.63
**Col4a2**	collagen, type IV, alpha 2	12.16	11.34	10.70
**Nid2**	nidogen 2	7.01	6.19	5.86
**Lamb1**	laminin B1	8.89	8.53	7.46
**Col1a2**	collagen, type I, alpha 2	13.15	12.10	11.52
**Postn**	periostin, osteoblast specific factor	12.17	11.40	10.78
**Pxdn**	peroxidasin	11.06	10.54	10.10
**Emid1**	EMI domain containing 1	8.72	8.26	7.36
**Col5a2**	collagen, type V, alpha 2	9.86	8.18	7.70
**Hmcn1**	hemicentin 1	10.43	9.43	8.53

**Mmp14**	matrix metallopeptidase 14 (membrane-inserted)	8.89	7.54	7.20
**Mmp2**	matrix metallopeptidase 2	10.54	9.77	9.38
**Slit2**	slit homolog 2 (Drosophila)	10.27	9.39	8.70
**Adamts2**	a disintegrin-like and metallopeptidase (reprolysin type) with thrombospondin type 1 motif, 2	9.67	8.00	7.19
**Col3a1**	collagen, type III, alpha 1	13.31	12.19	11.27
**Npnt**	nephronectin	11.58	11.11	10.20
**Ltbp3**	latent transforming growth factor beta binding protein 3	10.79	10.39	10.03
**Col6a3**	collagen, type VI, alpha 3	11.30	10.23	10.14
**Kcp**	kielin/chordin-like protein	8.34	7.45	7.40
**Emilin1**	elastin microfibril interfacer 1	7.80	7.42	7.20
**Adamts3**	a disintegrin-like and metallopeptidase (reprolysin type) with thrombospondin type 1 motif, 3	4.61	4.05	4.00
**Fbln5**	fibulin 5	11.17	10.41	10.25
**Fgf18**	fibroblast growth factor 18	7.75	7.05	6.66
**Col6a2**	collagen, type VI, alpha 2	11.67	10.45	10.36
**Dpt**	dermatopontin	11.45	11.07	10.88
**Col24a1**	collagen, type XXIV, alpha 1	3.70	3.28	3.19
**Adamts12**	a disintegrin-like and metallopeptidase (reprolysin type) with thrombospondin type 1 motif, 12	7.41	5.82	5.76
**Sparc**	secreted acidic cysteine rich glycoprotein	13.49	12.90	12.59
**Fras1**	Fraser extracellular matrix complex subunit 1	5.54	4.69	4.56
**Col16a1**	collagen, type XVI, alpha 1	8.89	8.11	7.70
**Adamts7**	a disintegrin-like and metallopeptidase (reprolysin type) with thrombospondin type 1 motif, 7	5.94	5.30	5.12
**Fbln5**	fibulin 5	11.17	10.41	10.25
**Col4a3**	collagen, type IV, alpha 3	9.96	9.96	8.77
**Col4a4**	collagen, type IV, alpha 4	8.82	8.82	7.39
**Adamts10**	a disintegrin-like and metallopeptidase (reprolysin type) with thrombospondin type 1 motif, 10	7.25	6.75	5.66
**Col13a1**	collagen, type XIII, alpha 1	9.22	9.11	8.14
**Thbs3**	thrombospondin 3	9.40	9.11	8.01

**Lama2**	laminin, alpha 2	9.05	8.98	8.64
**Lama3**	laminin, alpha 3	9.94	9.82	8.91
**Thsd1**	thrombospondin, type I, domain 1	8.72	8.41	7.70

Supplementary Table S6
Commonly regulated DEGs between the human test and validation set

Upregulated overalapped genes

Genes	Full name
COL1A1	collagen type I alpha 1 chain
COL1A2	collagen type I alpha 2 chain
COL3A1	collagen type III alpha 1 chain
COL6A1	collagen type VI alpha 1 chain
COL7A1	collagen type VII alpha 1 chain
COL9A2	collagen type IX alpha 2 chain
COL14A1	collagen type XIV alpha 1 chain
COL15A1	collagen type XV alpha 1 chain
COL16A1	collagen type XVI alpha 1 chain
COL17A1	collagen type XVII alpha 1 chain
CTHRC1	collagen triple helix repeat containing 1
ELN	elastin
FBLN2	fibulin 2
AEBP1	AE binding protein 1
EMILIN1	elastin microfibril interfacer 1
IGFBP5	insulin like growth factor binding protein 5
PODN	podocan
ZCCHC24	zinc finger CCHC-type containing 24
SULF2	sulfatase 2
PLVAP	plasmalemma vesicle associated protein
CAPN6	calpain 6
ITGA9	integrin subunit alpha 9
VCAM1	vascular cell adhesion molecule 1
CD40LG	CD40 ligand
IL5RA	interleukin 5 receptor subunit alpha

Downregulated overalapped genes

Genes	Full name
CTBS	chitobiase

Supplementary Table S7

798 housekeeping genes in mice which do not change their expression with age

Genes

Cdk12	Pex19	Clk1	Ilf3	Matr3
Hnf1a	Kars	Vps28	Phf12	Elovl5
Yipf3	Wdr33	Rpl22	Pom121	Ndufa3
Zfp184	Ppp6c	Rab2a	Ttc17	Eif3i
Lman1	Sh3gl1	Zfand5	Uchl5	Ssr3
Ddx27	Sbds	Sh3bgrl	Psip1	Cox6b1
Uxt	Syngr2	Clic4	Fam193a	Vdac2
Pogz	Sirt2	Ppib	Pum2	Cox7a2
Atp2c1	Dars	Vim	Hspe1	Srsf5
Pspc1	Tcf7l1	Rpl37a	Rab35	Tpm3
Etf1	Dctn3	Ogfod1	Sh3tc1	Cirh1a
Bcl7b	Usmg5	Casc3	Nosip	Arcn1
Odc1	Leprot	Dctn4	Ypel5	Eif3a
Pfkp	Banf1	Rpl5	Msl3	Nap1l1
Dcps	Usp22	Copb2	Pfdn2	Eif3f
Stx18	Slc44a2	Numb	Srf	Eif3m
Urm1	Nedd8	Ndufb11	Rspry1	Psen1
Gnaq	Tpm2	Atpif1	Ccdc90b	Sh2b1
Mars	Ndufb9	Surf2	Vps53	Zfp606
Apex1	Hsp90ab1	Celf1	Sf3b2	Chchd5
Coro1c	Gnb2	Btf3l4	Ube2b	Ifnar1
Ppp1r11	Tmsb10	Fbxo25	Gga2	Tbc1d1
Syf2	Pcsk7	Atg13	Ewsr1	Xrcc5
Fxr1	Polr1d	Ensa	Csnk1e	Hddc2
Arpp19	Flywch2	Taf6	Rad23b	Arf3
Yeats4	Atg3	Iars	Zfand3	AU019823
Ubxn1	Clns1a	Thoc7	Ube2g1	Gosr1
Wbp2	Eif3d	Usf2	Srp72	Frg1
Serbp1	Ddx23	Ptpra	Cdv3	Adipor1
Arf5	Dvl3	Flot2	Eif1ax	Arl8a
Mgrn1	Rps17	Anapc13	Rab8b	Manbal
Azi2	Me2	Nrbp1	Gpbp1	Prpf31
Sdhaf2	Rufy1	Bccip	B4galt1	Pycard
C1qbp	Eif2s3x	Fbxl5	Rnf10	Necap2
Cops6	Sult1a1	Sssca1	Ech1	Uros
Tsg101	Eapp	Rtn4	Stx7	Ergic1
Rexo2	BC005624	Uqcr11	Rnf181	Dda1
Rhot1	Emd	Ndel1	Prnp	Hexb
Ube2q1	Slc48a1	Ube2i	Actr3	Egr1
Pten	Pa2g4	Capzb	Hsp90aa1	Fchsd2
Glud1	Sipa1	Gdi2	Nop10	Nrd1
Pdcd10	Cdc123	Cct3	Klhdc3	Zfp36
H2afz	Ptp4a2	Tceb2	Tbca	Ppp2r5c
Pomp	Sorbs3	Arpc2	Caprin1	Grb2
Mtch1	Tardbp	Rpl7	Rpl18	Ddx19a

Pcnp	Vps26a	Tmsb4x	Zyx	Herpud1
Cdc42	Phpt1	Optn	Morf4l2	Ubtf
Hsp90b1	Cope	Picalm	Rps4x	Atp5g1
Gpx4	Ywhaz	Foxp4	Rps7	Vps29
Arf4	Set	Spg21	Ssrp1	Ndufb6
Eif4a2	Hexa	Tcf3	Nup54	Ggnbp2
Atp5g3	Hnrnpu	Uba1	Thrap3	Ankrd40
Rpl19	Cct5	Gtf3c6	Adrm1	Wtap
B4galt5	Hspb1	Kif22	Jun	Stk25
Ino80b	Gstp1	Ints3	Mff	Slc35b1
Cbx5	B2m	Tnfrsf13c	Cdkn1b	Basp1
H3f3b	Dynlrb1	Atf7ip	Rpl14	Akt1
Ggt7	Rcor3	Zfp566	Nupl2	Ndufb8
Mtif3	Tmem189	Sfswap	Csnk2a1	Paip2
Ttc1	Meaf6	F8a	Hdac2	Mdh2
Utf1	Yme1l1	Sumf2	9130011E15Rik	Tmem176a
Arl8b	Ccdc47	Pcmt1	Pmf1	Fam120a
Glyr1	Ahsa1	Gps2	St13	Dnaja1
Actn4	Cd300lg	Ercc1	Mrpl3	Sh3glb1
Atp5k	Sec62	Elf2	Dhps	App
Stub1	Gstm4	Ak2	Amdhd2	P4hb
Ppm1g	Eps15	Mcm4	Ndrg1	Tcof1
Xrcc6	Dapk3	Nfe2l1	Pdcd6ip	Ranbp9
Serf2	Mapkapk3	Brd2	Cirbp	Ssu72
Vapa	BC017158	Stam2	Hspbp1	Zfp87
Rnf141	Mrpl40	Dnajb1	Ccnt1	Ppp1cb
Pcsk1n	Oxsr1	Actb	Mfn2	Dpp7
Rinl	Rnf168	Ube2j1	Ube2m	Gstm7
Hsd17b11	Psmg2	Sat1	Bre	Vamp2
Atp6v1g2	Leng8	Dpf2	Zc3h15	Thyn1
Max	Ncoa5	Snrpc	Ifngr1	D8Ertd738e
Sap30bp	Atp6v1c1	Ghitm	Snx1	Ggps1
Slbp	Dap3	Ssr2	Mea1	
Hipk1	Fam173a	Hspd1	Cfdp1	
Gtf2ird2	Trim33	Atp5l	Tpd52l2	
Golph3l	Eif4e2	Psmb7	Srrt	
Exosc8	Ndufs5	Snrpe	Arid1a	
Taf9b	Nutf2-ps1	Atp5c1	Ndufb5	
Cinp	Cript	Rhoc	Creb3	
Aff4	Srp14	Ywhaq	Txndc12	
Parp6	Sfpq	Ppp1ca	Gnb1	
Ppp1r7	Zfp207	Rps15	2310036O22Rik	
4833439L19Rik	Trmt112	Hmgn1	Degs1	
Prpf38a	Nono	Ndufa8	Tmem50b	
Atf6	Cd81	Cog1	Timm23	
Mrps30	Rps24	Kpnb1	Psmc2	
Eif4e	Myl12b	Ipo5	Fnta	
Fbxo7	Tpm4	Cct6a	Prpf8	
Myg1	Rpl38	Nucb1	Eif3l	
Ubl5	Rps29	Atp5a1	Chmp5	

Mapkap1	Ociad1	Cap1	Rps27l	
Clasrp	Pex10	Anxa7	Eif2s2	
Ctcf	Rnf220	Tesk1	Ncl	
Mtss1	Mapkapk2	Tsta3	Pdha1	
Txnl1	Vdac3	Rpl12	Arf1	
Snx9	Fip1l1	Sec14l1	Cstb	
Cd2bp2	Ppp2cb	0610011F06Rik	Txnip	
Mrps18c	Hspa1b	Ndufaf3	Ran	
Nup98	Atn1	Tmx2	Cox5a	
Ncstn	Ap3m1	Utp11l	Tmbim6	
Ubr4	Fubp1	Fam53c	Snx3	
Itfg1	Sod3	Cetn2	Hnrnpa2b1	
Dazap1	Mta1	Polr2f	Cox4i1	
Tmem123	Fam32a	Rps25	Laptm4a	
Samm50	Stard3	Hsd17b12	Rabep2	
Lrpap1	Tomm22	Tspyl1	1110004F10Rik	
Actr10	Eif3g	Fkbp3	Zmat2	
Prkcsh	Drap1	Fam192a	Aprt	
Kpna2	Prkar1a	Srp68	Rpl4	
Rpl37	Btg1	Sgta	Ccdc86	
Rcn2	Eif4g1	Cic	Sart1	
Hdlbp	Slc25a39	Atp6v1f	Ppt1	
Glod4	Rps19	Psmd8	Gyltl1b	
Lrrc59	Kdm3b	Glrx5	Atf6b	
Tmco1	Trap1	Atxn10	Lrrfip2	
Gorasp2	Polr2i	Hp1bp3	Stx8	
Prickle4	Clta	Ndufa13	Nudc	
Eif4b	Rpl35	Mapk3	Gpbp1l1	
Sumo1	Atad3a	Zrsr2	Exosc10	
Specc1l	Npm1	Rnf41	Rps6	
Gltscr2	Ddx39b	Psmd3	Krt80	
Arglu1	Brd3	Eif3h	Sh3glb2	
Arl6ip1	Rps9	Gmppa	Rad23a	
Rpn1	Sf1	Arpc5	Ergic3	
Ywhae	Ciz1	Otub1	Stau1	
Ldha	Agap3	Smap2	G3bp2	
Cyb5r3	Mapre2	Zfp12	Aida	
Rbbp7	Vamp3	Flot1	Srp9	
Rac1	Rnf13	Pfdn5	Ppp1r2	
Uqcrh	Pafah1b1	Mprip	Lsm3	
Slc25a3	Cltb	Exosc1	Tiprl	
Sdcbp	Parp1	Psmf1	E430025E21Rik	
Ddx5	Nme1	Nomo1	Ube2h	
Hspa5	Vta1	Commd6	Csnk1d	
Calr	Ldhb	Apold1	Snx2	
Rpl18a	Pea15a	AI597479	Ndufa6	
Gdi1	Nap1l4	Pde6d	Ufc1	
Ppa1	Park7	Ubqln1	Vti1b	
Anp32a	Bud31	Hsf1	Tmcc2	
Prdx5	Gatad2a	Rbm6	Tra2b	

Atp5h	Ap2m1	Hnrnpl	Vkorc1	
Tmem59	Csnk2b	Fdps	Rtn3	
Zfp160	Bcap31	Dctn1	Arf6	
Ppp1r14b	Anapc5	Parp2	Gtf2i	
Dido1	Skp1a	Fam107b	Tcp1	
Lyrm1	Ccni	Rnaseh2c	Tmem50a	
Tlk1	Eef1a1	Polr2e	Rab11b	
Farsb	Eif3e	Fbxo3	Txn1	
Stambp	Rps20	Zfand6	Eif4h	
Srrm1	Med6	Cacybp	Dynll1	
Aup1	Zfpl1	Slc3a2	Ubap2l	
Ube2d1	Rab5b	Pdap1	Srrm2	
Arhgap17	Ppp1r10	Tcf25	Scamp3	
Mcmbp	Rsl1d1	Cdc37	Ccdc130	
Add3	Faf1	Atp5f1	Anapc2	
Ube2g2	Dpy30	Cycs	Ndufa10	
Pmpcb	Camta2	Iscu	Cd79b	
Aftph	Srsf7	Cmpk1	Mbtps1	
Atic	Arl6ip5	Rhob	Acot13	
Egln1	Ctdnep1	Nptn	Ngrn	
Casc4	Qrich1	Atp5b	Eif2ak1	
Srm	Fkbp4	Eif2b2	Cd99l2	
Maf1	Ube2z	Chmp2a	Apbb1	
Bmi1	Hadha	Canx	Tra2a	
Chic2	Cdc42se1	Zfp605	Ccdc124	
Hnrnpa1	Gars	Suv420h1	Stk24	
Rnf130	Tmx1	Mrps15	Anapc7	
Smurf2	Ndufa12	Dyrk1a	Cdc26	
Psmb3	Atp6v1g1	Bcor	Strap	
Idh3g	Hnrnpul1	Son	Pgd	
Btf3	Rnf11	1700025G04Rik	Wbp11	
Snrnp27	Eif3k	Ccng1	Rab14	
Atp5g2	Arpc3	Stip1	Med30	
Capza2	Mrfap1	Fam65a	Srsf6	
Mbnl1	Slc25a5	Prpf6	Ndufv2	

Supplementary Table S8
Inferred compositional changes of ECM in the lung of mice.
The cumulative expression of each ECM coding gene was summed up and set to 1. To calculate the relative expression for a given time point, we divided the specific expression value by the cumulative expression value and this provided a measure for relative compositional changes.

	Relative percentage (%)		
**Genes**	**Week 1-5**	**Week 6-26**	**Week 52-130**
**Lrg1**	0.38	1.14	2.08
**Adipoq**	0.08	0.40	1.13
**Fgg**	0.02	0.07	0.14
**Lgals3**	0.98	3.23	5.22
**Dcn**	0.86	3.05	5.77
**Col8a2**	0.03	0.11	0.24
**Fgl2**	0.19	0.48	0.95
**Timp4**	0.06	0.09	0.18
**Chad**	0.23	0.62	1.19
**Anxa8**	0.15	0.41	0.73
**Col9a2**	0.02	0.03	0.05
**Omd**	0.08	0.17	0.34
**Fga**	0.02	0.03	0.07
**Prg2**	0.02	0.03	0.05
**Mmp9**	0.05	0.11	0.23
**Mmp12**	0.04	0.08	0.15
**Mmp8**	0.05	0.10	0.17
**Emilin2**	0.13	0.24	0.48
**Mmrn1**	0.16	0.26	0.58
**Mmp3**	0.06	0.43	0.45
**Col8a1**	0.08	0.21	0.31
**Anxa7**	0.16	0.42	0.50
**Vwa5a**	0.47	1.21	1.51
**Serpinb6a**	1.10	2.93	3.64
**Spp1**	0.24	1.38	2.34
**Ecm1**	0.43	1.17	1.51
**Timp3**	2.07	4.54	6.31
**Aebp1**	0.26	0.52	0.96
**Col27a1**	0.27	0.21	0.39
**Vcan**	0.01	0.02	0.02
**Col15a1**	0.17	0.15	0.23
**Col2a1**	0.10	0.09	0.18
**Col6a4**	0.09	0.07	0.09
**Col9a1**	0.02	0.02	0.04
**Col5a3**	0.06	0.09	0.11
**Col11a1**	0.03	0.04	0.07
**Mmp16**	0.03	0.03	0.05
**Col10a1**	0.06	0.06	0.14
**Col11a2**	0.07	0.06	0.09
**Fmod**	0.16	0.18	0.35

**Col25a1**	0.04	0.02	0.03
**Tgfbi**	0.76	0.55	0.88
**Hapln1**	0.01	0.01	0.03
**Fbln2**	0.22	0.18	0.28
**Fgf14**	0.03	0.03	0.04
**Igfbp4**	3.30	3.53	4.43
**Fn1**	3.99	2.69	3.27
**Fbn2**	0.05	0.03	0.03
**Adamts18**	0.02	0.02	0.03
**Mmp24**	0.13	0.14	0.16
**Igfals**	0.13	0.14	0.17
**Adamts16**	0.15	0.14	0.18
**Adamts4**	0.04	0.04	0.04
**Elane**	0.07	0.07	0.09
**Aspn**	0.26	0.17	0.21
**Col6a1**	2.26	1.81	2.09
**Ntn3**	0.04	0.04	0.04
**Anxa13**	0.02	0.03	0.06
**Mmp13**	0.01	0.02	0.04
**Dmbt1**	0.02	0.03	0.10
**Prg4**	0.04	0.06	0.30
**Acan**	0.06	0.09	0.19
**Thbs4**	0.04	0.06	0.11
**Eln**	3.61	1.11	0.79
**Lama1**	0.04	0.05	0.04
**Mfap4**	5.13	3.46	2.38
**Mfap2**	1.44	1.22	0.96
**Fbn1**	1.14	0.70	0.50
**Hspg2**	0.39	0.55	0.41
**Col1a1**	2.79	1.19	0.77
**Fgf4**	0.06	0.06	0.04
**Col5a1**	0.26	0.17	0.14
**Col4a1**	7.16	5.56	3.56
**Tnc**	0.03	0.02	0.01
**Lamc1**	1.26	0.95	0.69
**Lama4**	0.25	0.14	0.06
**Nid1**	0.68	0.46	0.30
**Ltbp2**	1.14	0.63	0.48
**Col4a2**	3.31	2.94	2.02
**Nid2**	0.09	0.08	0.07
**Lamb1**	0.34	0.42	0.21
**Col1a2**	6.58	4.99	3.57
**Postn**	3.34	3.06	2.14
**Pxdn**	1.54	1.68	1.34
**Emid1**	0.30	0.35	0.20
**Col5a2**	0.67	0.33	0.25
**Hmcn1**	1.00	0.78	0.45
**Mmp14**	0.34	0.21	0.18
**Mmp2**	1.08	0.99	0.81
**Slit2**	0.89	0.76	0.50

**Adamts2**	0.59	0.29	0.18
**Col3a1**	7.33	5.31	3.01
**Npnt**	2.22	2.50	1.43
**Ltbp3**	1.28	1.52	1.27
**Col6a3**	1.82	1.36	1.37
**Kcp**	0.23	0.20	0.20
**Emilin1**	0.16	0.19	0.18
**Adamts3**	0.02	0.02	0.02
**Fbln5**	1.67	1.55	1.48
**Fgf18**	0.16	0.15	0.12
**Col6a2**	2.36	1.58	1.60
**Dpt**	2.03	2.44	2.29
**Col24a1**	0.01	0.01	0.01
**Adamts12**	0.12	0.06	0.07
**Sparc**	8.34	8.68	7.50
**Fras1**	0.03	0.03	0.03
**Col16a1**	0.34	0.31	0.25
**Adamts7**	0.04	0.04	0.04
**Fbln5**	1.67	1.55	1.48
**Col4a3**	0.72	1.13	0.53
**Col4a4**	0.33	0.51	0.20
**Adamts10**	0.11	0.12	0.06
**Col13a1**	0.43	0.63	0.34
**Thbs3**	0.49	0.63	0.31
**Lama2**	0.38	0.57	0.48
**Lama3**	0.71	1.03	0.59
**Thsd1**	0.31	0.38	0.25

Supplementary Table S9

Genes of the senescence-associated secretory phenotype

Mouse data

		6-26~1-5		52-130~6-26		52-130~1-5	
**Interleukins**	**Full names**	**logFC**	**adj-P**	**logFC**	**adj-P**	**logFC**	**adj-P**
Il6	interleukin 6	0.18	0.31	0.06	0.70	0.62	0.01
Il7	interleukin 7	0.57	0.00	0.90	0.00	1.52	0.00
Il1a	interleukin 1 alpha	0.11	0.40	0.11	0.40	0.20	0.17
Il1b	interleukin 1 beta	0.41	0.09	1.00	0.00	1.73	0.00
Il13	interleukin 13	0.07	0.53	-0.03	0.76	-0.02	0.83
Il15	interleukin 15	0.05	0.84	0.41	0.04	0.49	0.00
**Chemokines**							
Cxcl15	chemokine (C-X-C motif) ligand 15	0.31	0.00	-1.67	0.00	-1.05	0.16
Cxcl1	chemokine (C-X-C motif) ligand 1	-0.79	0.00	0.59	0.00	-0.63	0.00
Cxcl2	chemokine (C-X-C motif) ligand 2	0.01	0.91	0.82	0.00	1.03	0.00
Gro-G	growth-related oncogene						
Ccl8	chemokine (C-C motif) ligand 8	0.29	0.30	1.94	0.00	2.33	0.00
Mcpt4	mast cell protease 4	1.25	0.00	1.76	0.00	3.26	0.00
Ccl2	chemokine (C-C motif) ligand 2	0.15	0.18	0.15	0.14	0.21	0.05
Ccl20	chemokine (C-C motif) ligand 20	-0.34	0.02	0.87	0.00	0.30	0.14
Ccl3	chemokine (C-C motif) ligand 3	-0.12	0.47	0.39	0.01	0.48	0.03
Ccl16	chemokine (C-C motif) ligand 16						
Ccl11	chemokine (C-C motif) ligand 11	0.40	0.00	0.82	0.00	1.16	0.00
Ccl26	chemokine (C-C motif) ligand 26						
Ccl25	chemokine (C-C motif) ligand 25	-0.54	0.00	-0.01	0.92	-0.60	0.00
Cxcl5	chemokine (C-X-C motif) ligand 5	0.12	0.73	0.04	0.89	0.38	0.15
Ccl1	chemokine (C-C motif) ligand 1	-0.14	0.20	-0.12	0.23	-0.39	0.01
Cxcl11	chemokine (C-X-C motif) ligand 11	-0.04	0.63	-0.11	0.20	-0.05	0.55
**Other Inflammatory Molecules**							
Tgfb1	transforming growth factor, beta 1	-0.51	0.01	0.17	0.51	-0.54	0.00
Csf2	colony stimulating factor 2 (granulocyte-macrophage)	-0.12	0.41	0.04	0.79	-0.11	0.48

Human data

	Human test Aged~Adult		Human validation Aged~Adult
**Interleukins**	**logFC**	**adj-P**	**adj-P**
IL6	0.29	0.25	0.06
IL7	-0.06	0.73	0.61
IL1A	-1.90		0.24
IL1B	-1.57		0.32
IL13	0.42	0.20	0.82
IL15	-0.15	0.37	0.29
**Chemokines**			
CXCL8	-0.45	0.12	
CXCL1	0.12	0.94	0.59
CXCL2	0.35	0.23	0.84
GRO-G			
CCL8	-0.47	0.10	0.02
CCL13	-1.31		0.24
CCL2	0.04	0.89	0.07
CCL20	-0.35	0.14	0.28
CCL3	-0.60	0.07	
CCL16	-0.42	0.27	0.73
CCL11	0.45	0.11	0.10
CCL26	0.08	0.98	0.85
CCL25	0.05	0.60	0.94
CXCL5	-0.26	0.28	0.14
CCL1	0.33	0.15	0.09
CXCL11	0.05	0.94	0.98
**Other Inflammatory Molecules**			
TGFB1	0.08	0.65	0.63
CSF2	-0.29	0.43	0.21

Csf3	colony stimulating factor 3 (granulocyte)	-0.42	0.01	0.28	0.09	-0.35	0.03
Ifng	interferon gamma	0.32	0.01	-0.07	0.52	0.41	0.00
Cxcl13	chemokine (C-X-C motif) ligand 13	1.25	0.00	1.76	0.00	3.26	0.00
Mif	macrophage migration inhibitory factor	-0.50	0.00	0.51	0.01	0.57	0.00
**Growth Factors; Regulators**							
Areg	amphiregulin	0.80	0.00	-0.61	0.00	0.16	0.34
Ereg	epiregulin	-0.00	0.98	0.65	0.00	0.58	0.03
Nrg1	neuregulin 1	0.14	0.11	0.10	0.27	0.21	0.02
Egf	epidermal growth factor	-0.15	0.28	0.26	0.05	-0.16	0.22
Fgf2	fibroblast growth factor 2	0.27	0.49	-0.61	0.07	-0.45	0.01
Hgf	hepatocyte growth factor	0.32	0.07	-0.75	0.00	-1.20	0.00
Fgf7	fibroblast growth factor 7	0.72	0.00	-0.29	0.02	0.94	0.00
Vegfa	vascular endothelial growth factor A	0.21	0.09	-0.51	0.01	-0.84	0.01
Ang	angiogenin, ribonuclease, RNase A family, 5	0.94	0.00	0.49	0.28	1.38	0.00
Scf	stem cell factor						
Cxcl12	chemokine (C-X-C motif) ligand 12	0.55	0.03	0.59	0.01	1.26	0.00
Pigf	phosphatidylinositol glycan anchor biosynthesis, class F	0.46	0.00	-0.02	0.89	0.57	0.00
Ngf	nerve growth factor	-0.11	0.55	-0.07	0.62	-0.27	0.12
Igfbp2	insulin-like growth factor binding protein 2	0.27	0.29	0.06	0.83	0.30	0.46
Igfbp3	insulin-like growth factor binding protein 3	2.27	0.00	-0.69	0.00	1.68	0.00
Igfbp4	insulin-like growth factor binding protein 4	-0.94	0.00	0.23	0.02	-1.11	0.00
Igfbp6	insulin-like growth factor binding protein 6	1.64	0.00	-0.19	0.25	1.44	0.00
Igfbp7	insulin-like growth factor binding protein 7	0.33	0.04	-0.50	0.00	-0.59	0.00
**Proteases and Regulators**							
Mmp1a	matrix metallopeptidase 1a (interstitial collagenase)	-0.07	0.31	0.09	0.15	-0.02	0.81
Mmp3	matrix metallopeptidase 3	2.26	0.00	-0.04	0.84	2.34	0.00
Mmp10	matrix metallopeptidase 10	-0.11	0.24	0.09	0.31	-0.03	0.77
Mmp12	matrix metallopeptidase 12	0.20	0.24	0.91	0.00	1.31	0.00

CSF3	0.37	0.27	0.68
IFNG	0.24	0.38	0.32
CXCL13	0.56	0.41	0.01
MIF	-0.54	0.02	0.06
**Growth Factors; Regulators**			
AREG	0.51	0.03	0.07
EREG	0.06	0.95	0.67
NRG1	0.22	0.40	0.62
EGF	-0.32	0.27	0.00
FGF2	-0.01	0.93	0.43
HGF	0.09	0.65	0.52
FGF7	-0.47	0.04	0.52
VEGFA	0.51	0.02	0.26
ANG	-0.14	0.41	0.26
SCF			
CXCL12	0.35	0.11	0.01
PIGF	-0.29	0.02	0.57
NGF	0.16	0.57	0.46
IGFBP2	0.47	0.06	0.00
IGFBP3	0.17	0.58	0.15
IGFBP4	-0.10	0.61	0.02
IGFBP6	0.10	0.74	0.01
IGFBP7	0.02	0.95	0.00
**Proteases and Regulators**			
MMP1	0.06	0.35	0.32
MMP3	0.17	0.28	0.29
MMP10	0.60	0.00	0.10
MMP12	-1.06	0.00	0.32

Mmp13	matrix metallopeptidase 13	-0.08	0.43	1.22	0.00	1.12	0.06
Mmp14	matrix metallopeptidase 14 (membrane-inserted)	-1.35	0.00	-0.34	0.07	-1.64	0.00
Timp1	tissue inhibitor of metalloproteinase 1	0.16	0.51	0.29	0.19	0.36	0.06
Timp2	tissue inhibitor of metalloproteinase 2	0.39	0.00	-0.21	0.04	-0.78	0.01
Serpine1	serine (or cysteine) peptidase inhibitor, clade E, member 1	0.47	0.05	0.09	0.66	0.46	0.01
Serpine2	serine (or cysteine) peptidase inhibitor, clade E, member 2	-0.47	0.03	0.07	0.55	-0.34	0.18
Plat	plasminogen activator, tissue	0.16	0.26	0.59	0.06	0.57	0.02
Plau	plasminogen activator, urokinase	0.32	0.42	-0.38	0.28	-0.38	0.00
Ctsb	cathepsin B	0.70	0.00	0.41	0.00	1.13	0.00
**Receptors; Ligands**							
Icam1	intercellular adhesion molecule 1	0.81	0.00	-0.50	0.00	0.32	0.28
Icam3	intercellular adhesion molecule 3	#NV	#NV	#NV	#NV	#NV	#NV
Tnfsf11	tumor necrosis factor (ligand) superfamily, member 11	0.24	0.03	0.10	0.37	0.28	0.00
Tnfrsf1a	tumor necrosis factor receptor superfamily, member 1a	-0.05	0.84	0.14	0.49	0.02	0.88
Tnfrsf1b	tumor necrosis factor receptor superfamily, member 1b	0.52	0.03	0.08	0.59	0.49	0.00
Tnfrsf10c	tumor necrosis factor receptor superfamily, member 10c	#NV	#NV	#NV	#NV	#NV	#NV
Fas	Fas (TNF receptor superfamily member 6)	0.89	0.00	-0.30	0.14	0.85	0.00
Plaur	plasminogen activator, urokinase receptor	0.18	0.25	-0.44	0.00	-0.07	0.69
Il6st	interleukin 6 signal transducer	0.96	0.00	-1.28	0.01	0.80	0.00
Egfr	epidermal growth factor receptor	-0.92	0.00	-0.47	0.01	-0.67	0.00
**Non-Protein Molecules**							
Ptger2	prostaglandin E receptor 2 (subtype EP2)	-0.18	0.51	0.08	0.74	-0.03	0.90
Nos2	nitric oxide synthase 2, inducible	-0.41	0.00	-0.05	0.64	-0.51	0.00
Ros1	Ros1 proto-oncogene	-0.57	0.00	0.26	0.02	-0.37	0.04
**Insoluble Factors**							
Fn1	fibronectin 1	-1.43	0.00	0.18	0.16	-1.38	0.00
collagens							

MMP13	0.34	0.18	0.51
MMP14	0.36	0.09	0.70
TIMP1	0.21	0.44	0.01
TIMP2	0.13	0.43	0.08
SERPINE1	0.22	0.32	0.27
SERPINE2	0.06	0.75	0.02
PLAT	0.09	0.66	0.01
PLAU	-0.62	0.01	0.41
CTSB	-0.99	0.00	0.67
**Receptors; Ligands**			
ICAM1	0.40	0.21	0.28
ICAM3	-0.04	0.87	0.00
TNFSF11	0.07	0.31	0.76
TNFRSF1A	-0.15	0.42	0.43
TNFRSF1B	0.14	0.51	0.10
TNFRSF10C	0.64	0.01	0.03
FAS	0.02	0.95	0.08
PLAUR	0.05	0.88	0.87
IL6ST	-0.29	0.07	0.61
EGFR	0.16	0.42	0.06
**Non-Protein Molecules**			
PTGER2	-0.41	0.08	0.23
NOS2	-0.09	0.77	0.80
ROS1	0.23	0.30	0.21
**Insoluble Factors**			
FN1	0.37	0.13	0.64
collagens			

laminin							

laminin			

Supplementary Table S10A

We compared the age dependent gene expression changes of the present study to published findings.

The data are log FC of aged divided by adult mice.

Genes	Full names	Our study		Our study		Our study		PMID:30814501 Aged/Adult				PMID:33397975		PMID: 2556897		PMID: 33982668		PMID:1776388
		6-26~1-5		52-130~6-26		52-130~1-5		In silico transcripto		Bulk transcriptome		PMID:26954258		PMID:29212667		PMID:1772692		
		LogFC	p-value		p-value		p-value	LogFC	Protein									
Ighg	Immunoglobulin heavy chain (gamma polypeptide)	0.99	0.00	3.67	0.00	4.75	0.00											
Jchain	immunoglobulin joining chain	2.07	0.00	3.58	0.00	5.89	0.00			3.83								
Ighm	immunoglobulin heavy constant mu	1.24	0.00	3.15	0.00	4.65	0.00	4.93	1.85	3.50				Upregula	1.73			
Igk	immunoglobulin kappa chain complex	0.22	0.14	3.34	0.00	3.58	0.00								-1.95			
Igkv6-20	immunoglobulin kappa variable 6-20	0.28	0.11	3.16	0.00	3.89	0.00	10.33		5.77								
Igkv9-120	immunoglobulin kappa chain variable 9-120	-0.07	0.51	0.05	0.59	-0.11	0.14	3.83		1.95								
Iglc1		1.19	6.17	2.46	0.00	3.93	0.00											
LOC100503923		0.72	5.63	2.86	0.00	3.62	0.00											
Iglv1	immunoglobulin lambda variable 1	1.05	7.00	2.52	0.00	3.65	0.00	5.14		3.58								
Igkv8-30	immunoglobulin kappa chain variable 8-30	1.75	9.35	2.45	0.00	4.47	0.00	5.28		1.64								
Igkv6-23		1.61	10.71	2.36	0.00	3.83	0.00	4.78		4.62								
Igkv6-14	immunoglobulin kappa variable 6-14	0.70	5.17	1.95	0.00	2.91	0.00	4.54		1.39								
Ccl8	chemokine (C-C motif) ligand 8	0.29	7.26	1.94	0.00	2.33	0.00	3.75		3.34				Upregulated				
Cxcl13	chemokine (C-X-C motif) ligand 13	1.25	6.85	1.76	0.00	3.26	0.00	1.94		2.00				Upregula	1.99			
Cd177	CD177 antigen	0.66	6.33	2.14	0.00	2.76	0.00	1.76	0.99	2.98					2.48			
LOC664787	Sp110 nuclear body protein pseudogene	-0.52	6.48	1.81	0.00	1.31	0.00											
Mzb1	marginal zone B and B1 cell-specific protein 1	0.33	7.18	1.51	0.00	1.99	0.00	2.78		2.31								
LOC102638047		0.02	4.05	1.51	0.00	1.97	0.00											
Slc6a20a	solute carrier family 6 (neurotransmitter transporter), member 20A	1.38	5.99	1.58	0.00	3.23	0.00	0.97		1.14								
Ighv1-72		-0.31	5.01	1.44	0.00	1.48	0.00	3.27		2.63								
Clec4e	C-type lectin domain family 4, member e	-0.08	5.79	1.26	0.00	1.44	0.00	1.74		1.35					1.75			

LOC100503923		-0.07	5.18	1.77	0.00	1.33	0.00										
Igkv6-14	immunoglobulin kappa variable 6-14	0.64	5.40	1.21	0.00	2.20	0.00	4.54		1.39							
Wfdc17	WAP four-disulfide core domain 17	0.80	7.93	1.42	0.00	2.24	0.00	2.51		1.99					1.98		
Tcrg-V4	T cell receptor gamma, variable 4	0.76	6.19	1.20	0.00	2.11	0.00	NA		0.13							
Slc2a3	solute carrier family 2 (facilitated glucose transporter), member 3	0.62	8.02	1.60	0.00	1.94	0.00	2.20		1.16							
Serpina3g	serine (or cysteine) peptidase inhibitor, clade A, member 3G	1.04	7.76	1.04	0.00	2.22	0.00	2.49		1.37							
Il1b	interleukin 1 beta	0.41	7.43	1.00	0.00	1.73	0.00	2.44		2.34	AgeDown						
Pla2g7	phospholipase A2, group VII (platelet-activating factor acetylhydrolase, plasma)	0.16	7.40	1.29	0.00	1.45	0.00	1.35		1.52					1.99		
Rgs1	regulator of G-protein signaling 1	0.85	5.92	0.98	0.00	2.13	0.00	1.30		1.33				Upregula	1.18		
Rgs18	regulator of G-protein signaling 18	0.93	6.54	1.26	0.00	2.08	0.00	1.73		1.40							
Clec4d	C-type lectin domain family 4, member d	0.39	6.54	0.97	0.00	1.49	0.00	2.08		2.38					1.63		
Ighg3	Immunoglobulin heavy constant gamma 3	-0.21	6.59	1.29	0.00	0.90	0.00	5.24	-0.55	3.93				Upregulated			
Igkv4-68	immunoglobulin kappa variable 4-68	0.16	4.08	0.99	0.00	1.55	0.00	2.59		3.40							
Cxcr6	chemokine (C-X-C motif) receptor 6	0.57	6.07	1.09	0.00	1.83	0.00	2.64		1.78				Upregulated			
Cd300lb	CD300 molecule like family member B	0.39	6.29	0.96	0.00	1.47	0.00	1.48		1.16							
Oosp1	oocyte secreted protein 1	-0.65	5.29	1.09	0.00	0.53	0.08	3.99		3.19							
Gzmk	granzyme K	-0.09	5.82	1.03	0.00	1.07	0.00	5.29		5.21	AgeUp			Upregulated			
Stfa2l1	stefin A2 like 1	-1.23	5.07	0.99	0.00	-0.28	0.59	3.89		2.85					1.93		
Ccl9	chemokine (C-C motif) ligand 9	0.82	8.68	1.07	0.00	2.06	0.00	0.37		1.89					2.52		
Vwc2	von Willebrand factor C domain containing 2	0.00	6.06	1.24	0.00	0.88	0.00	0.73		0.99							
Igkv4-72	immunoglobulin kappa chain variable 4-72	0.24	4.85	0.87	0.00	1.29	0.00	2.62		4.49							
Pla2g2d	phospholipase A2, group IID	0.36	5.60	0.82	0.00	1.41	0.00	2.15		0.97				Upregulated			
LOC101055758		-0.24	5.37	1.13	0.00	0.77	0.00								1.64		
Cxcl2	chemokine (C-X-C motif) ligand 2	0.01	4.70	0.82	0.00	1.03	0.00	1.16		0.82							
AI661384	expressed sequence AI661384	1.35	6.31	0.93	0.00	2.26	0.00										
Cd5l	CD5 antigen-like	-0.16	6.11	1.01	0.00	0.92	0.00	4.03		-1.63							
Cybb	cytochrome b-245, beta polypeptide	0.13	8.06	0.72	0.00	1.09	0.00	0.61	0.58	0.44					1.74		
Zc3h12d	zinc finger CCCH type containing 12D	0.06	5.37	0.96	0.00	1.02	0.00	2.07		1.11							

Mmp9	matrix metallopeptidase 9	0.44	6.45	1.00	0.00	1.35	0.00	0.94	0.61	1.36							downregulated
Slc11a1	solute carrier family 11 (proton-coupled divalent metal ion transporters), member 1	-0.20	7.51	0.94	0.00	0.76	0.00	1.90		1.26					1.51		
Stap1	signal transducing adaptor family member 1	0.38	5.87	0.76	0.00	1.28	0.00	1.39		0.78				Upregulated			
Il7r	interleukin 7 receptor	1.14	7.95	0.77	0.00	1.96	0.00	1.72		0.97					1.35		
9530053H05Rik	RIKEN cDNA 9530053H05 gene	0.63	7.10	0.71	0.00	1.62	0.00										
Evi2a	ecotropic viral integration site 2a	0.84	7.47	0.73	0.00	1.78	0.00	0.73		1.38							
Fcna	ficolin A	-0.10	6.76	0.99	0.00	0.82	0.00	1.10		-0.72							
Il7	interleukin 7	0.57	6.23	0.90	0.00	1.52	0.00	-0.27		0.62				Upregulated			
Naip2	NLR family, apoptosis inhibitory protein 2	0.64	6.73	0.80	0.00	1.48	0.00	0.20		0.45					1.26		
Hpse	heparanase	0.32	7.00	1.09	0.00	1.27	0.00	1.46		0.99					1.27		
Gm34940		-0.28	5.51	0.86	0.00	0.72	0.00										
Pisd-ps1		0.33	9.69	0.69	0.00	1.18	0.00										
Fyb	FYN binding protein	0.61	7.45	0.64	0.00	1.49	0.00	1.38	0.47	0.48				Upregulated			
Tlr13	toll-like receptor 13	0.47	7.34	0.57	0.00	1.20	0.00	1.19		0.65					2.45		
Tcrg-V4	T cell receptor gamma, variable 4	-0.16	5.53	0.76	0.00	0.74	0.00	NA		0.13							
Ncf4	neutrophil cytosolic factor 4	0.39	7.60	0.63	0.00	1.13	0.00	1.46	0.35	1.61							
5430427O19Rik	RIKEN cDNA 5430427O19 gene	0.13	5.53	0.76	0.00	0.93	0.00	1.00		1.32							
Itgam	integrin alpha M	0.09	6.09	0.66	0.00	0.81	0.00	1.44	0.53	0.32					2.19		
Cd300ld	CD300 molecule like family member d	0.04	7.14	0.70	0.00	0.68	0.00	1.75		0.76					1.22		
Pisd-ps3	phosphatidylserine decarboxylase, pseudogene 3	0.10	10.59	0.76	0.00	0.86	0.00										
Cst7	cystatin F (leukocystatin)	0.20	5.60	0.80	0.00	0.99	0.00	3.21		2.20				Upregulated			
Gm32088		-0.19	7.60	0.68	0.00	0.51	0.00										
Cdk19	cyclin-dependent kinase 19	0.44	9.43	0.64	0.00	1.17	0.00	0.48		0.77							
Trem3	triggering receptor expressed on myeloid cells 3	0.11	6.56	0.58	0.00	0.73	0.00	1.40		1.54							
Cnp	2',3'-cyclic nucleotide 3' phosphodiesterase	0.47	8.32	0.58	0.00	1.08	0.00	1.20	0.17	0.85							
Cxcr3	chemokine (C-X-C motif) receptor 3	-0.15	6.83	0.57	0.00	0.46	0.00	2.12		2.17				Upregulated			
Zbtb32	zinc finger and BTB domain containing 32	-0.27	7.27	0.42	0.00	0.22	0.21	1.52		0.57				Upregulated			

Kcnn4	potassium intermediate/small conductance calcium-activated channel, subfamily N, member 4	-0.09	7.95	0.35	0.00	0.35	0.01	1.32		1.77							
Hist1h4m								1.31		0.18							
St8sia6	ST8 alpha-N-acetyl-neuraminide alpha-2,8-sialyltransferase 6	0.79	0.01	1.72	0.00	2.35	0.00	1.58		-1.20							
Ms4a6b	membrane-spanning 4-domains, subfamily A, member 6B	1.01	0.00	0.61	0.00	1.68	0.00	1.86		1.82					1.14		
Evi2a	ecotropic viral integration site 2a	0.84	0.00	0.73	0.00	1.78	0.00	0.73		1.38							
Nnt	nicotinamide nucleotide transhydrogenase	-0.70	0.00	-0.33	0.23	-1.15	0.00	-1.31	-8.25	-0.27				Downregulated			
Kdelr3	KDEL (Lys-Asp-Glu-Leu) endoplasmic reticulum protein retention receptor 3	-0.71	0.00	-0.33	0.06	-1.00	0.00	-0.06		0.49							
Heph	hephaestin	-0.48	0.01	-0.80	0.00	-1.15	0.00	-2.43		-1.19				Upregulated			
Mmp2	matrix metallopeptidase 2	-1.06	0.00	-0.40	0.07	-1.20	0.00	-0.21	-0.26	0.27	AgeUp			Upregulated			downegulated
Mmp14	matrix metallopeptidase 14 (membrane-inserted)	-1.35	0.00	-0.34	0.07	-1.64	0.00	0.22		0.44				Upregula	1.25		
Adamts2	a disintegrin-like and metallopeptidase (reprolysin type) with thrombospondin type 1 motif, 2	-1.68	0.00	-0.80	0.00	-2.46	0.00	-0.51		-0.88				Upregulated			
Lrg1	leucine-rich alpha-2-glycoprotein 1	0.94	0.00	0.77	0.02	1.60	0.00	-0.02	2.60	1.80				Downreg	1.61		
Dcn	decorin	1.18	0.00	0.82	0.00	1.86	0.00	0.15	-0.64	-0.22		-1.94	up in old sample				
Lrg1	leucine-rich alpha-2-glycoprotein 1	0.94	0.00	0.77	0.02	1.60	0.00	-0.02	2.60	1.80				Downreg	1.61		
Adipoq	adiponectin, C1Q and collagen domain containing	1.67	0.00	1.39	0.00	3.02	0.00	-0.50	-0.57	-1.63							
Fgg	fibrinogen gamma chain	0.85	0.04	0.94	0.03	2.12	0.00	-0.95	0.38	1.83					1.34		
Lgals3	lectin, galactose binding, soluble 3	1.07	0.00	0.59	0.01	1.68	0.00	-0.10	0.39	1.92					1.40		
Dcn	decorin	1.18	0.00	0.82	0.00	1.86	0.00	0.15	-0.64	-0.22		-1.94	up in old sample				
Col8a2	collagen, type VIII, alpha 2	1.03	0.00	1.01	0.00	1.88	0.00	0.75		0.60							
Fgl2	fibrinogen-like protein 2	0.85	0.00	0.89	0.01	1.71	0.00	0.73		0.35				Upregulated			
Timp4	tissue inhibitor of metalloproteinase 4	1.12	0.00	0.93	0.00	2.61	0.00	0.23		1.33							
Chad	chondroadherin	0.79	0.01	0.84	0.02	1.55	0.00	-1.05	-1.72	0.28					-2.39		
Anxa8	annexin A8	0.80	0.00	0.75	0.00	1.95	0.00	0.33	-0.14	0.36							
genes																	
col9a2	collagen, type IX, alpha 2	0.03	0.76	0.63	0.00	0.56	0.09	0.27		3.30				Upregulated			
Omd	osteomodulin	0.52	0.06	0.89	0.03	1.43	0.03	-0.54		-0.83							

Fga	fibrinogen alpha chain	0.36	0.43	1.10	0.02	1.44	0.00	-0.23	0.33	1.50							
Prg2	proteoglycan 2, bone marrow	0.23	0.17	0.53	0.00	1.01	0.00	6.70	0.75	0.95							
Mmp9	matrix metallopeptidase 9	0.44	0.02	1.00	0.00	1.27	0.00	0.94	0.61	1.36							downregulated
Mmp12	matrix metallopeptidase 12	0.20	0.24	0.91	0.00	1.31	0.00	0.70		2.76	AgeDown				3.20		
Mmp8	matrix metallopeptidase 8	0.41	0.07	0.69	0.01	1.08	0.02	0.54	0.52	0.66					2.13		
Emilin2	elastin microfibril interfacer 2	0.27	0.23	0.88	0.00	1.24	0.00	0.95	-0.31	0.79							
Mmrn1	multimerin 1	0.07	0.33	1.06	0.00	1.12	0.00	-1.11	0.43	-0.34							
genes																	
mmp3	matrix metallopeptidase 3	2.26	0.00	-0.04	0.84	2.34	0.00	0.28		0.20					1.19		
Col8a1	collagen, type VIII, alpha 1	0.73	0.00	0.46	0.09	1.14	0.00	0.23	0.12	-0.36				Upregula	1.29		
Anxa7	annexin A7	0.74	0.00	0.13	0.15	0.92	0.00	-0.05	-0.20	-0.72							
Vwa5a	von Willebrand factor A domain containing 5A	0.72	0.00	0.23	0.09	1.03	0.00	0.24	0.24	-0.47				Upregulated			
Serpinb6a	serine (or cysteine) peptidase inhibitor, clade B, member 6a	0.76	0.00	0.22	0.16	1.08	0.00	-0.16		0.49							
Spp1	secreted phosphoprotein 1	1.90	0.00	0.66	0.07	2.74	0.00	0.19		1.12					3.40		
Ecm1	extracellular matrix protein 1	0.80	0.00	0.27	0.10	0.93	0.00	0.48	-0.63	0.41				Upregulated			
Timp3	tissue inhibitor of metalloproteinase 3	0.71	0.00	0.38	0.04	0.87	0.00	-0.38	1.12	-2.54							
genes																	
Aebp1	AE binding protein 1	-0.79	0.00	0.79	0.00	1.14	0.00	0.04	0.21	0.36	AgeUp			Upregulated			
Col27a1	collagen, type XXVII, alpha 1	-0.99	0.00	0.77	0.00	-0.67	0.02	-0.10		-0.48							
Vcan	versican	-1.42	0.00	0.42	0.00	-1.45	0.00	1.92	0.31	-0.25					1.55		
Col15a1	collagen, type XV, alpha 1	-0.82	0.00	0.49	0.01	-0.53	0.06	-0.71	0.77	-0.32							
Col2a1	collagen, type II, alpha 1	-0.88	0.00	0.95	0.00	-0.16	0.65	-0.45		0.15							
Col6a4	collagen, type VI, alpha 4	-0.96	0.00	0.26	0.09	-0.68	0.00	-0.50	-3.88	-1.89							
Col9a1	collagen, type IX, alpha 1	-0.55	0.00	1.02	0.00	-0.34	0.05	-0.86		0.97							
Col5a3	collagen, type V, alpha 3	-0.16	0.14	0.26	0.11	-0.24	0.01	-0.35		0.68							
Col11a1	collagen, type XI, alpha 1	-0.61	0.00	0.88	0.00	0.78	0.20	-0.42		-1.52							
Mmp16	matrix metallopeptidase 16	-0.55	0.00	0.37	0.01	-0.33	0.00	-1.17		-1.73							
Col10a1	collagen, type X, alpha 1	-0.62	0.00	1.09	0.00	0.19	0.71	-0.50	0.88	1.62	AgeUp			Upregulated			
Col11a2	collagen, type XI, alpha 2	-0.90	0.00	0.48	0.00	-0.61	0.00	0.90		0.91	AgeUp						
Fmod	fibromodulin	-0.45	0.00	0.88	0.00	1.18	0.04	0.74	-3.67	-0.69				Upregulated			
Col25a1	collagen, type XXV, alpha 1	-0.70	0.00	0.46	0.01	-0.76	0.00			-1.98							

Tgfbi	transforming growth factor, beta induced	-1.11	0.00	0.56	0.02	-0.52	0.17	0.19	0.19	0.47					1.96		
Hapln1	hyaluronan and proteoglycan link protein 1	-0.53	0.00	0.88	0.00	0.45	0.36	-1.53	0.27	-1.87				Downregulated			
Fbln2	fibulin 2	-0.93	0.00	0.51	0.01	-0.60	0.10	-0.98	0.22	-0.12				Upregulated			
genes																	
Fgf14	fibroblast growth factor 14	-0.59	0.00	0.16	0.14	-0.46	0.00	-1.26		-1.14	AgeUp			Upregulated			
Igfbp4	insulin-like growth factor binding protein 4	-0.94	0.00	0.23	0.02	-1.11	0.00	-0.10		0.24							
Fn1	fibronectin 1	-1.43	0.00	0.18	0.16	-1.38	0.00	0.27	0.22	-1.00		-0.79			1.56		
Fbn2	fibrillin 2	-1.78	0.00	0.18	0.13	-1.92	0.00	-0.08		-1.70							
Adamts18	a disintegrin-like and metallopeptidase (reprolysin type) with thrombospondin type 1 motif, 18	-0.66	0.00	0.12	0.28	-0.62	0.00	0.32		-1.25							
Mmp24	matrix metallopeptidase 24	-0.43	0.00	0.07	0.66	-0.55	0.00	0.68		-0.92							
Igfals	insulin-like growth factor binding protein, acid labile subunit	-0.57	0.00	0.22	0.11	-0.51	0.00	-1.25		0.35							
Adamts16	a disintegrin-like and metallopeptidase (reprolysin type) with thrombospondin type 1 motif, 16	-0.68	0.00	0.23	0.05	-0.56	0.00	NA									
Adamts4	a disintegrin-like and metallopeptidase (reprolysin type) with thrombospondin type 1 motif, 4	-0.60	0.00	0.12	0.26	-0.67	0.00	-0.45		0.38					1.80		
Elane	elastase, neutrophil expressed	-0.69	0.00	0.17	0.37	-0.54	0.02	1.87		0.47							
Aspn	asporin	-1.21	0.00	0.21	0.37	-0.86	0.06	1.52	-0.22	-0.37	AgeUp						
Col6a1	collagen, type VI, alpha 1	-0.97	0.00	0.11	0.68	-1.00	0.00	-1.36	0.12	-0.49			up in old	Upregulated			
Ntn3	netrin 3	-0.65	0.00	0.01	0.96	-0.78	0.00	-0.49									
genes																	
Anxa13	annexin A13	-0.03	0.77	0.72	0.00	0.52	0.08	1.08		0.16							
mmp13	matrix metallopeptidase 13	-0.08	0.43	1.22	0.00	1.12	0.06	1.47		0.52					2.00		
Dmbt1	deleted in malignant brain tumors 1	-0.14	0.56	1.69	0.00	0.98	0.12	1.83		2.53							
Prg4	proteoglycan 4 (megakaryocyte stimulating factor, articular superficial zone protein)	-0.08	0.76	2.28	0.00	1.61	0.01	0.79		-3.12				Upregula	1.13		
Acan	aggrecan	-0.12	0.20	0.93	0.00	0.57	0.28			-0.77							
Thbs4	thrombospondin 4	-0.12	0.33	0.82	0.00	0.60	0.04	NA		0.53							
genes																	
Eln	elastin	-2.35	0.00	-0.59	0.01	-2.99	0.00	-0.33	0.74	0.57	AgeUp	-1.59		Upregulated		downregulated	

Lama1	laminin, alpha 1	-0.30	0.05	-0.25	0.04	-0.58	0.00	0.27	-0.29	1.34					1.88		
Mfap4	microfibrillar-associated protein 4	-1.22	0.00	-0.64	0.00	-1.77	0.00	0.84	0.39	0.49	AgeUp	-1.40		Upregulated			
Mfap2	microfibrillar-associated protein 2	-0.90	0.00	-0.44	0.01	-1.33	0.00	0.53		0.54							
Fbn1	fibrillin 1	-1.36	0.00	-0.59	0.00	-1.99	0.00	-0.25	-0.26	-0.69							
Hspg2	perlecan (heparan sulfate proteoglycan 2)	-0.94	0.00	-0.53	0.00	-0.90	0.00	-0.79	0.42	-0.89		-0.82	up in old sample				
Col1a1	collagen, type I, alpha 1	-2.03	0.00	-0.73	0.00	-2.73	0.00	-1.36	-0.79	-0.52							
Fgf4	fibroblast growth factor 4	-0.61	0.00	-0.41	0.00	-1.06	0.00			-1.74							
Col5a1	collagen, type V, alpha 1	-1.28	0.00	-0.33	0.00	-1.76	0.00	-0.18	-0.49	-0.28							
Col4a1	collagen, type IV, alpha 1	-1.01	0.00	-0.74	0.00	-1.82	0.00	-0.96	0.72	-0.62				Downregulated			
Tnc	tenascin C	-2.03	0.00	-0.15	0.17	-1.69	0.00	1.03	-0.19	-0.30					3.23		
Lamc1	laminin, gamma 1	-1.06	0.00	-0.57	0.00	-2.03	0.00	-0.82	0.24	-1.13							
Lama4	laminin, alpha 4	-1.50	0.00	-1.26	0.00	-2.68	0.00	-0.49	-0.77	-1.11							
Nid1	nidogen 1	-0.96	0.00	-0.72	0.00	-2.16	0.00	0.86	0.28	-1.25							
Ltbp2	latent transforming growth factor beta binding protein 2	-1.51	0.00	-0.49	0.00	-1.96	0.00	-0.25	-1.61	-0.35				Upregulated			
Col4a2	collagen, type IV, alpha 2	-0.82	0.00	-0.64	0.00	-1.51	0.00	-0.87	0.68	-0.54			up in old sample				
Nid2	nidogen 2	-0.82	0.00	-0.33	0.09	-1.25	0.00	-0.73		-1.24							
Lamb1	laminin B1	-0.64	0.00	-1.07	0.00	-1.54	0.00	0.32	-1.65	-0.32							
Col1a2	collagen, type I, alpha 2	-2.04	0.00	-0.58	0.00	-2.82	0.00	-1.07	-0.69	-0.42							
Postn	periostin, osteoblast specific factor	-0.77	0.00	-0.62	0.00	-1.24	0.00	-1.26	0.75	-0.98							
Pxdn	peroxidasin	-0.52	0.00	-0.43	0.00	-0.91	0.00	0.47	0.17	-0.61							
Emid1	EMI domain containing 1	-0.46	0.01	-0.90	0.00	-1.27	0.00	-0.86	0.18	0.20				Upregulated			
Col5a2	collagen, type V, alpha 2	-1.68	0.00	-0.48	0.00	-2.05	0.00	-1.07	-0.37	-0.32					1.13		
Hmcn1	hemicentin 1	-1.00	0.00	-0.91	0.00	-1.65	0.00	0.75	-0.60	-2.12					-1.13		
Mmp14	matrix metallopeptidase 14 (membrane-inserted)	-1.35	0.00	-0.34	0.07	-1.64	0.00	0.22		0.44				Upregula	1.25		
Mmp2	matrix metallopeptidase 2	-1.06	0.00	-0.40	0.07	-1.20	0.00	-0.21	-0.26	0.27	AgeUp			Upregulated			downegulated
Slit2	slit homolog 2 (Drosophila)	-1.20	0.00	-0.70	0.00	-1.62	0.00	-0.38		-1.18				Upregulated			
Adamts2	a disintegrin-like and metallopeptidase (reprolysin type) with thrombospondin type 1 motif, 2	-1.68	0.00	-0.80	0.00	-2.46	0.00	-0.51		-0.88				Upregulated			
Col3a1	collagen, type III, alpha 1	-1.91	0.00	-0.92	0.00	-1.99	0.00	-1.24	-0.71	-0.19						downreg	upregulated
Npnt	nephronectin	-0.73	0.00	-0.91	0.00	-2.22	0.00	-1.04	-0.22	-1.86		0.19					

Ltbp3	latent transforming growth factor beta binding protein 3	-0.65	0.00	-0.71	0.00	-0.97	0.00	-0.74		-0.22	AgeUp			Upregulated			
genes																	
Col6a3	collagen, type VI, alpha 3	-1.07	0.00	-0.09	0.75	-1.18	0.00	-0.03	0.11	-0.72							
Kcp	kielin/chordin-like protein	-0.89	0.00	-0.05	0.69	-0.93	0.00	-0.10		0.32							
Emilin1	elastin microfibril interfacer 1	-0.38	0.04	-0.23	0.16	-0.64	0.00	-1.05	-0.85	0.56		-1.29		Upregulated			
Adamts3	a disintegrin-like and metallopeptidase (reprolysin type) with thrombospondin type 1 motif, 3	-0.56	0.00	-0.05	0.65	-0.66	0.00	1.06		-1.14							
Fbln5	fibulin 5	-0.61	0.01	-0.16	0.51	-0.59	0.00	-1.34	-0.23	-0.44	AgeUp	-0.35		Upregulated			
Fgf18	fibroblast growth factor 18	-0.71	0.00	-0.39	0.04	-1.10	0.00	0.57		-0.58							
Col6a2	collagen, type VI, alpha 2	-1.23	0.00	-0.08	0.71	-1.34	0.00	-1.19	0.16	-0.31				Upregulated			
Dpt	dermatopontin	-0.38	0.00	-0.19	0.13	-0.53	0.02	-0.51	-0.33	-0.12	AgeUp			Upregulated			
Col24a1	collagen, type XXIV, alpha 1	-0.43	0.00	-0.09	0.06	-0.53	0.00	-0.86		-1.44							
Adamts12	a disintegrin-like and metallopeptidase (reprolysin type) with thrombospondin type 1 motif, 12	-1.58	0.00	-0.06	0.62	-1.65	0.00	0.44		-2.40				Upregulated			
Sparc	secreted acidic cysteine rich glycoprotein	-0.59	0.00	-0.31	0.00	-1.03	0.00	-1.29	-0.21	-0.39							
Fras1	Fraser extracellular matrix complex subunit 1	-1.48	0.00	-0.13	0.35	-1.79	0.00	-0.62	-3.57	-1.40				Upregulated			
Col16a1	collagen, type XVI, alpha 1	-0.78	0.00	-0.42	0.00	-1.23	0.00	-0.77	4.24	0.63				Upregulated			
Adamts7	a disintegrin-like and metallopeptidase (reprolysin type) with thrombospondin type 1 motif, 7	-0.65	0.00	-0.17	0.16	-0.88	0.00	NA		-0.29							
fbln5	fibulin 5	-0.61	0.01	-0.16	0.51	-0.59	0.00	-1.34	-0.23	-0.44	AgeUp	-0.35		Upregulated			
genes																	
Col4a3	collagen, type IV, alpha 3	-0.06	0.71	-1.19	0.00	-1.08	0.01	-1.36	1.56	-1.64			up in old sample				
Col4a4	collagen, type IV, alpha 4	-0.21	0.05	-1.43	0.00	-1.31	0.00	-1.39	0.97	-1.44			up in old sample				
Adamts10	a disintegrin-like and metallopeptidase (reprolysin type) with thrombospondin type 1 motif, 10	-0.40	0.00	-1.08	0.00	-1.49	0.00	0.74		-0.24							
Col13a1	collagen, type XIII, alpha 1	-0.11	0.38	-0.97	0.00	-0.87	0.00	1.01		-0.19				Upregulated			
Thbs3	thrombospondin 3	-0.28	0.01	-1.10	0.00	-1.20	0.00	-0.99	-1.54	-0.46				Upregula	-1.26		
Lama2	laminin, alpha 2	-0.07	0.60	-0.34	0.02	-0.35	0.02	-0.06	-1.15	-0.94		-1.00					
lama3	laminin, alpha 3	-0.11	0.34	-0.91	0.00	-0.31	0.00	-1.69	0.38	-1.67				Downregulated			
Thsd1	thrombospondin, type I, domain 1	-0.31	0.02	-0.70	0.00	-0.72	0.00	0.22		-0.34							

S100a4	S100 calcium binding protein A4	1.26	0.00	0.69	0.00	2.09	0.00	1.58	0.38	1.84							
Lgals1	lectin, galactose binding, soluble 1	-0.43	0.03	0.50	0.01	-0.10	0.61	0.36	0.24	0.69				Upregula	1.45		
Smad3	SMAD family member 3	-0.40	0.10	-0.45	0.04	-0.76	0.00	-0.19	-0.59	-0.22				Upregulated			
Lgals3	lectin, galactose binding, soluble 3	1.07	0.00	0.59	0.01	1.68	0.00	-0.10	0.39	1.92					1.40		
SASP																	
Interleukins																	
Il6	interleukin 6	0.18	0.31	0.06	0.70	0.62	0.01	2.42		0.82					1.75		
Il7	interleukin 7	0.57	0.00	0.90	0.00	1.52	0.00	-0.27		0.62				Upregulated			
Il1a	interleukin 1 alpha	0.11	0.40	0.11	0.40	0.20	0.17	1.02		-0.37				Downregulated			
Il1b	interleukin 1 beta	0.41	0.09	1.00	0.00	1.73	0.00	2.44		2.34	AgeDown						
Il13	interleukin 13	0.07	0.53	-0.03	0.76	-0.02	0.83	-0.04		2.72	AgeUp						
Il15	interleukin 15	0.05	0.84	0.41	0.04	0.49	0.00	0.50		0.26				Upregulated			
Chemokines																	
Cxcl15	chemokine (C-X-C motif) ligand 15	0.31	0.00	-1.67	0.00	-1.05	0.16	-0.99	-0.48	0.25							
Cxcl1	chemokine (C-X-C motif) ligand 1	-0.79	0.00	0.59	0.00	-0.63	0.00	-0.60		1.69	AgeDown			Downregulated			
Cxcl2	chemokine (C-X-C motif) ligand 2	0.01	0.91	0.82	0.00	1.03	0.00	1.16		0.82							
Gro-g																	
Ccl8	chemokine (C-C motif) ligand 8	0.29	0.30	1.94	0.00	2.33	0.00	3.75		3.34				Upregulated			
Mcpt4	mast cell protease 4	-0.53	0.00	0.85	0.00	0.17	0.53	-1.15	-1.14	1.53							
Ccl2	chemokine (C-C motif) ligand 2	0.15	0.18	0.15	0.14	0.21	0.05	1.10		1.42					2.35		
Ccl20	chemokine (C-C motif) ligand 20	-0.34	0.02	0.87	0.00	0.30	0.14	4.40		2.37	AgeDown			Downregulated			
Ccl3	chemokine (C-C motif) ligand 3	-0.12	0.47	0.39	0.01	0.48	0.03	1.75		2.58					2.13		
Ccl16																	
Ccl11	chemokine (C-C motif) ligand 11	0.40	0.00	0.82	0.00	1.16	0.00	-0.79		0.59							
Ccl26										-0.77							
Ccl25	chemokine (C-C motif) ligand 25	-0.54	0.00	-0.01	0.92	-0.60	0.00	-0.08		0.13							
Cxcl5	chemokine (C-X-C motif) ligand 5	0.12	0.73	0.04	0.89	0.38	0.15	1.77		1.94							
Ccl1	chemokine (C-C motif) ligand 1	-0.14	0.20	-0.12	0.23	-0.39	0.01	-1.46		2.75							
Cxcl11	chemokine (C-X-C motif) ligand 11	-0.04	0.63	-0.11	0.20	-0.05	0.55							Upregulated			
Other inflammatory molecules																	
Tgfb1	transforming growth factor, beta 1	-0.51	0.01	0.17	0.51	-0.54	0.00	-0.36		0.44				Upregulated			
Csf2	colony stimulating factor 2 (granulocyte-macrophage)	-0.12	0.41	0.04	0.79	-0.11	0.48	-0.26		0.44	AgeDown						

Csf3	colony stimulating factor 3 (granulocyte)	-0.42	0.01	0.28	0.09	-0.35	0.03	-1.20		1.29				Downregulated			
Ifng	interferon gamma	0.32	0.01	-0.07	0.52	0.41	0.00	2.12		1.59				Upregulated			
Cxcl13	chemokine (C-X-C motif) ligand 13	1.25	0.00	1.76	0.00	3.26	0.00	1.94		2.00				Upregula	1.99		
Mif	macrophage migration inhibitory factor	-0.50	0.00	0.51	0.01	0.57	0.00	-0.20	0.19	1.39							
Growth factors; regulators																	
Areg	amphiregulin	0.80	0.00	-0.61	0.00	0.16	0.34	-1.48		0.71					1.52		
Ereg	epiregulin	-0.00	0.98	0.65	0.00	0.58	0.03	1.04		-1.14					1.17		
Nrg1	neuregulin 1	0.14	0.11	0.10	0.27	0.21	0.02	0.03		-0.65							
Egf	epidermal growth factor	-0.15	0.28	0.26	0.05	-0.16	0.22	-0.45		-0.31				Downregulated			
Fgf2	fibroblast growth factor 2	0.27	0.49	-0.61	0.07	-0.45	0.01	0.81	-0.82	-3.39				Upregulated			
Hgf	hepatocyte growth factor	0.32	0.07	-0.75	0.00	-1.20	0.00	-0.05		-1.88				Upregulated			
Fgf7	fibroblast growth factor 7	0.72	0.00	-0.29	0.02	0.94	0.00	0.40		-0.50							
Vegfa	vascular endothelial growth factor A	0.21	0.09	-0.51	0.01	-0.84	0.01	-0.26		-0.52				Downregulated			
Ang	angiogenin, ribonuclease, RNase A family, 5	0.94	0.00	0.49	0.28	1.38	0.00	-1.03		0.23							
Scf																	
Cxcl12	chemokine (C-X-C motif) ligand 12	0.55	0.03	0.59	0.01	1.26	0.00	1.13	2.86	-0.55				Upregulated			
Pigf	phosphatidylinositol glycan anchor biosynthesis, class F	0.46	0.00	-0.02	0.89	0.57	0.00	-0.06		0.23							
Ngf	nerve growth factor	-0.11	0.55	-0.07	0.62	-0.27	0.12	-0.50		-0.13							
Igfbp2	insulin-like growth factor binding protein 2	0.27	0.29	0.06	0.83	0.30	0.46	-0.08	1.87	1.14				Upregulated			
Igfbp3	insulin-like growth factor binding protein 3	2.27	0.00	-0.69	0.00	1.68	0.00	0.51		-0.28	AgeUp			Upregula	-1.62		
Igfbp4	insulin-like growth factor binding protein 4	-0.94	0.00	0.23	0.02	-1.11	0.00	-0.10		0.24							
Igfbp6	insulin-like growth factor binding protein 6	1.64	0.00	-0.19	0.25	1.44	0.00	-0.74	-0.67	0.42				Upregulated			
Igfbp7	insulin-like growth factor binding protein 7	0.33	0.04	-0.50	0.00	-0.59	0.00	-0.85	-0.97	-0.49	AgeUp			Upregulated			
Proteases and regulators																	
Mmp1a	matrix metallopeptidase 1a (interstitial collagenase)	-0.07	0.31	0.09	0.15	-0.02	0.81										
Mmp3	matrix metallopeptidase 3	2.26	0.00	-0.04	0.84	2.34	0.00	0.28		0.20					1.19		
Mmp10	matrix metallopeptidase 10	-0.11	0.24	0.09	0.31	-0.03	0.77	NA		1.67							
Mmp12	matrix metallopeptidase 12	0.20	0.24	0.91	0.00	1.31	0.00	0.70		2.76	AgeDown				3.20		

Mmp13	matrix metallopeptidase 13	-0.08	0.43	1.22	0.00	1.12	0.06	1.47		0.52					2.00		
Mmp14	matrix metallopeptidase 14 (membrane-inserted)	-1.35	0.00	-0.34	0.07	-1.64	0.00	0.22		0.44				Upregula	1.25		
Timp1	tissue inhibitor of metalloproteinase 1	0.16	0.51	0.29	0.19	0.36	0.06	-0.84		1.96				Upregula	2.63		upregulated
Timp2	tissue inhibitor of metalloproteinase 2	0.39	0.00	-0.21	0.04	-0.78	0.01	0.41		-0.53				Upregulated			upregulated
Serpine1	serine (or cysteine) peptidase inhibitor, clade E, member 1	0.47	0.05	0.09	0.66	0.46	0.01	-0.33		-0.53					1.77		
Serpine2	serine (or cysteine) peptidase inhibitor, clade E, member 2	-0.47	0.03	0.07	0.55	-0.34	0.18	-0.35	0.82	0.15							
Plat	plasminogen activator, tissue	0.16	0.26	0.59	0.06	0.57	0.02	-0.10	1.16	-0.88				Upregulated			
Plau	plasminogen activator, urokinase	0.32	0.42	-0.38	0.28	-0.38	0.00	0.86	-0.19	0.52					1.45		
Ctsb	cathepsin B	0.70	0.00	0.41	0.00	1.13	0.00	0.34	-0.61	0.31					1.36		
Receptors; ligands																	
Icam1	intercellular adhesion molecule 1	0.81	0.00	-0.50	0.00	0.32	0.28	-0.47	-0.23	-0.62							
Icam3														Upregulated			
Tnfsf11	tumor necrosis factor (ligand) superfamily, member 11	0.24	0.03	0.10	0.37	0.28	0.00	1.02		0.82							
Tnfrsf1a	tumor necrosis factor receptor superfamily, member 1a	-0.05	0.84	0.14	0.49	0.02	0.88	0.18		0.25							
Tnfrsf1b	tumor necrosis factor receptor superfamily, member 1b	0.52	0.03	0.08	0.59	0.49	0.00	1.09		0.63				Upregula	1.30		
Tnfrsf10c																	
Fas	Fas (TNF receptor superfamily member 6)	0.89	0.00	-0.30	0.14	0.85	0.00	0.24	-0.28	0.32				Upregulated			
Plaur	plasminogen activator, urokinase receptor	0.18	0.25	-0.44	0.00	-0.07	0.69	0.14		-0.15							
Il6st	interleukin 6 signal transducer	0.96	0.00	-1.28	0.01	0.80	0.00	0.20		-0.64							
Egfr	epidermal growth factor receptor	-0.92	0.00	-0.47	0.01	-0.67	0.00	-0.04		-1.23							
Non-protein molecules																	
Ptger2	prostaglandin E receptor 2 (subtype EP2)	-0.18	0.51	0.08	0.74	-0.03	0.90	-0.40		0.49							
Nos2	nitric oxide synthase 2, inducible	-0.41	0.00	-0.05	0.64	-0.51	0.00	0.44		0.27	AgeUp						
Ros1	Ros1 proto-oncogene	-0.57	0.00	0.26	0.02	-0.37	0.04	-0.66		-0.33							
Insoluble factors																	
Fn1	fibronectin 1	-1.43	0.00	0.18	0.16	-1.38	0.00	0.27	0.22	-1.00		-0.79			1.56		
collagens																	

laminin																	
Ccr2	chemokine (C-C motif) receptor 2	0.70	0.00	0.66	0.00	1.80	0.00	1.54		1.27					1.38		
Ccl2	chemokine (C-C motif) ligand 2	0.15	0.18	0.15	0.14	0.21	0.05	1.10		1.42					2.35		
Cxcr2	chemokine (C-X-C motif) receptor 2	-0.16	0.43	0.39	0.07	0.18	0.49	2.29		1.64							
Senescence regulation genes																	
Trp53	transformation related protein 53	-0.26	0.00	0.55	0.00	-0.31	0.00	0.08		0.28							
Sirt1	sirtuin 1	0.67	0.00	0.03	0.87	0.71	0.00	0.19		-0.61							
Nfe2l2	nuclear factor, erythroid derived 2, like 2	0.46	0.14	0.15	0.36	0.80	0.00	-0.47		-0.32							
Sod1	superoxide dismutase 1, soluble	0.49	0.00	-0.45	0.00	0.37	0.00	-0.19	-0.55	0.62							
Nox4	NADPH oxidase 4	0.23	0.14	-0.54	0.00	0.27	0.12	1.46		-0.87	AgeUp			Upregulated			
Trp53inp1	transformation related protein 53 inducible nuclear protein 1	0.69	0.00	-0.76	0.01	0.72	0.00	0.79		-0.59							
Atg10	autophagy related 10	0.54	0.00	-0.01	0.95	0.67	0.00	-0.61		-0.15							
MAP1LC3B	microtubule-associated protein 1 light chain 3 beta	0.42	0.00	-0.54	0.01	0.45	0.00	-0.45		0.29							
Cdkn1a	cyclin-dependent kinase inhibitor 1A (P21)	1.18	0.00	-0.77	0.09	0.91	0.00	0.23		0.67				Upregula	1.45		
CDKN2A	cyclin-dependent kinase inhibitor 2A	-0.16	0.14	-0.01	0.96	-0.09	0.27	2.54		1.47	AgeUp			Upregulated			
cdk1	cyclin-dependent kinase 1	-1.25	0.00	0.18	0.16	-0.95	0.00	-0.84		0.52	AgeDown				1.37		
sesn1	sestrin 1	0.64	0.00	-0.68	0.00	0.47	0.01	0.27		-0.29							
sesn2	sestrin 2	0.29	0.05	-0.37	0.00	-0.38	0.03	-0.79		-0.84							
foxo3	forkhead box O3	0.74	0.00	-0.28	0.02	0.49	0.00	-0.68		-0.83	AgeUp			Upregulated			
ppargc1a	peroxisome proliferative activated receptor, gamma, coactivator 1 alpha	0.90	0.00	0.33	0.06	0.70	0.00	-0.18		-1.15							
terf1	telomeric repeat binding factor 1	0.66	0.00	0.43	0.07	0.65	0.00	-0.13		0.11							
grhl2	grainyhead-like 2 (Drosophila)	0.68	0.00	0.34	0.07	1.10	0.00	-0.37		-0.28							
pax8	paired box 8	-0.79	0.00	0.22	0.02	-0.86	0.00	-0.09		1.23							
terf2	telomeric repeat binding factor 2	-0.34	0.00	-0.41	0.03	-0.50	0.00	-0.40	0.44	0.16							
tert	telomerase reverse transcriptase	-1.08	0.00	0.33	0.04	-0.98	0.00	1.20		-0.98							
Immune genes																	
Fcgr1	Fc receptor, IgG, high affinity I	-0.66	0.00	0.29	0.05	-0.42	0.00	0.51		1.16					2.35		
Adgre1	adhesion G protein-coupled receptor E1	0.17	0.60	0.70	0.01	1.01	0.00			0.17					1.74		
Cd68	CD68 antigen	0.57	0.01	0.63	0.00	1.15	0.00	0.08		0.88					2.73		
Il2rb	interleukin 2 receptor, beta chain	0.42	0.01	0.41	0.01	0.73	0.00	1.78		0.76				Upregulated			

Cd19	CD19 antigen	0.12	0.54	0.43	0.07	0.61	0.05	1.95		1.77							
Cd3d	CD3 antigen, delta polypeptide	0.57	0.01	0.47	0.03	1.06	0.00	2.03		2.69							
Cd4	CD4 antigen	-0.43	0.01	0.21	0.17	-0.23	0.05	1.88		0.92				Upregulated			
Cd8a	CD8 antigen, alpha chain	-0.65	0.02	0.54	0.01	-0.37	0.06	3.08		2.47							
Fcgr3	Fc receptor, IgG, low affinity III	0.72	0.00	0.38	0.02	1.18	0.00	0.25		0.40					1.66		
Itgax	integrin alpha X	0.72	0.00	0.04	0.83	0.78	0.01	-0.03		0.22					1.18		
Bst1	bone marrow stromal cell antigen 1	0.91	0.00	-0.21	0.40	1.06	0.01	0.07	0.42	0.46					1.53		
Gda	guanine deaminase	0.70	0.01	1.16	0.00	1.50	0.00	0.59	0.68	0.18					1.54		
Laptm5	lysosomal-associated protein transmembrane 5	0.91	0.00	0.26	0.01	1.06	0.00	0.20		0.87							
Ms4a8a	membrane-spanning 4-domains, subfamily A, member 8A	0.64	0.01	0.64	0.00	1.17	0.00	-0.85		0.95					1.72		
Slpi	secretory leukocyte peptidase inhibitor	0.82	0.00	0.64	0.02	1.48	0.00	0.24		0.75							
ptprc	protein tyrosine phosphatase, receptor type, C	0.96	0.00	0.36	0.03	1.44	0.00	1.11	0.11	0.72				Upregulated			
Ccr2	chemokine (C-C motif) receptor 2	0.70	0.00	0.66	0.00	1.80	0.00	1.54		1.27					1.38		
Evi2a	ecotropic viral integration site 2a	0.84	0.00	0.73	0.00	1.78	0.00	0.73		1.38							
F13a1	coagulation factor XIII, A1 subunit	0.98	0.00	1.50	0.00	2.40	0.00	1.19	0.82	1.14				Upregula	1.38		
H2-Aa	histocompatibility 2, class II antigen A, alpha	1.63	0.00	0.24	0.02	1.86	0.00	0.54	0.30	1.15							
H2-Ab1	histocompatibility 2, class II antigen A, beta 1	1.44	0.00	0.25	0.08	1.66	0.00	0.48		1.44							
H2-Eb1	histocompatibility 2, class II antigen E beta	1.43	0.00	0.58	0.00	1.95	0.00	0.81		1.37							
Ms4a6b	membrane-spanning 4-domains, subfamily A, member 6B	1.01	0.00	0.61	0.00	1.68	0.00	1.86		1.82					1.14		
Pirb	paired Ig-like receptor B	0.61	0.00	0.37	0.02	1.14	0.00	0.82		1.69					1.16		
Ccl5	chemokine (C-C motif) ligand 5	0.94	0.00	0.72	0.00	1.65	0.00	2.50		2.41				Upregulated			
Cd52	CD52 antigen	1.00	0.01	0.79	0.03	2.01	0.00	1.33		1.97					1.26		
H2-K1	histocompatibility 2, K1, K region	0.95	0.00	0.43	0.00	1.22	0.00	0.99	0.12	1.20							
Ms4a4b	membrane-spanning 4-domains, subfamily A, member 4B	1.17	0.00	-0.43	0.00	1.38	0.01	2.16		1.58							
Sell	selectin, lymphocyte	0.52	0.01	0.18	0.51	0.80	0.01	1.38		0.96							
Cd79a	CD79A antigen (immunoglobulin-associated alpha)	0.65	0.01	0.45	0.16	1.32	0.00	1.90		2.36				Upregulated			
Ighm	immunoglobulin heavy constant mu	1.24	0.00	3.15	0.00	4.65	0.00	4.93	1.85	3.50				Upregula	1.73		
Ltb	lymphotoxin B	0.98	0.00	0.58	0.05	1.66	0.00	1.71		2.35				Upregulated			

Il7r	interleukin 7 receptor	1.14	0.00	0.77	0.00	1.96	0.00	1.72		0.97					1.35		
S100a4	S100 calcium binding protein A4	1.26	0.00	0.69	0.00	2.09	0.00	1.58	0.38	1.84							
Spn	sialophorin	1.03	0.00	0.38	0.01	1.38	0.00	1.74		1.84							
Psmb8	proteasome (prosome, macropain) subunit, beta type 8 (large multifunctional peptidase 7)	1.31	0.00	-0.10	0.41	1.45	0.00	1.25	-0.30	1.53				Upregulated			
Cd177	CD177 antigen	0.66	0.00	2.14	0.00	2.76	0.00	1.76	0.99	2.98					2.48		
Gsr	glutathione reductase	0.72	0.00	0.63	0.00	1.23	0.00	0.81	0.19	0.65							
Lrg1	leucine-rich alpha-2-glycoprotein 1	0.94	0.00	0.77	0.02	1.60	0.00	-0.02	2.60	1.80				Downreg	1.61		
Plek	pleckstrin	1.05	0.00	0.70	0.02	1.70	0.00	0.54	0.67	0.76				Upregula	1.41		
Scnn1a	sodium channel, nonvoltage-gated 1 alpha	0.08	0.79	0.50	0.06	0.38	0.15	-0.87		-0.37							
Cd3g	CD3 antigen, gamma polypeptide	0.73	0.00	0.62	0.01	1.34	0.00	2.04		2.84				Upregulated			
Cd3e	CD3 antigen, epsilon polypeptide	-0.16	0.31	0.48	0.00	0.07	0.56	2.36		1.44				Upregulated			
Cd247	CD247 antigen	0.27	0.09	0.23	0.11	0.54	0.00	2.48		1.12				Upregulated			
Lck	lymphocyte protein tyrosine kinase	0.51	0.09	-0.55	0.02	1.08	0.00	2.06		2.11				Upregulated			
Zap70	zeta-chain (TCR) associated protein kinase	-0.70	0.00	0.21	0.07	-0.68	0.00	1.66		1.57				Upregulated			
Lat	linker for activation of T cells	0.35	0.03	0.38	0.02	0.63	0.00	1.79		2.40				Upregulated			
Lcp2	lymphocyte cytosolic protein 2	0.61	0.00	0.25	0.03	1.07	0.00	1.21		0.94				Upregulated			
Cd28	CD28 antigen	0.66	0.00	0.83	0.00	1.21	0.00	1.97		0.83				Upregulated			
Cd80	CD80 antigen	0.14	0.22	0.32	0.01	0.40	0.00	0.95		0.29					1.26		
Cd5	CD5 antigen	-0.05	0.75	0.35	0.02	0.15	0.32	2.64		2.27				Upregulated			
Icos	inducible T cell co-stimulator	-0.50	0.00	0.51	0.00	0.16	0.23	2.13		0.84				Upregulated			
Icosl	icos ligand	-0.39	0.00	0.23	0.09	-0.47	0.00	0.47		-0.32							
Il2	interleukin 2	-0.34	0.00	0.15	0.19	-0.37	0.00	0.48		1.71							
Il4	interleukin 4	-0.28	0.02	0.21	0.07	-0.16	0.22	1.21		0.67							
Il10	interleukin 10	-0.38	0.00	0.49	0.00	-0.01	0.92	2.99		3.41							
Il17a	interleukin 17A	-0.45	0.03	0.36	0.04	-0.13	0.33	0.68		1.82							
Tnf	tumor necrosis factor	0.13	0.25	0.28	0.01	0.28	0.01	1.21		2.34				Upregulated			
Nlrp3	NLR family, pyrin domain containing 3	-0.35	0.04	0.40	0.01	-0.08	0.59	0.66		0.76				Upregulated			
Pycard	PYD and CARD domain containing	-0.55	0.00	0.62	0.00	1.19	0.00	0.69	0.49	1.28							
Casp1	caspase 1	1.01	0.00	0.94	0.00	2.16	0.00	0.40	1.65	1.17							
Surfactant																	

Chka	choline kinase alpha	0.79	0.00	-0.80	0.00	0.47	0.00	-0.33		-0.32				Downregulated			
Chkb	choline kinase beta	0.22	0.06	-0.12	0.22	0.16	0.14	0.15		0.63							
Pcyt1a	phosphate cytidylyltransferase 1, choline, alpha isoform	0.56	0.00	-0.38	0.03	0.55	0.00	-0.38	-0.85	-0.48							
Chpt1	choline phosphotransferase 1	1.02	0.00	0.39	0.00	1.26	0.00	-0.06		-0.79							
Plpp1	phospholipid phosphatase 1	0.23	0.14	-0.21	0.13	-0.21	0.01			-0.37							
Plpp2	phospholipid phosphatase 2	0.26	0.02	0.33	0.00	0.63	0.00			0.44							
Ptdss1	phosphatidylserine synthase 1	0.29	0.00	-0.33	0.32	0.22	0.02	-0.13		-0.15							
Ptdss2	phosphatidylserine synthase 2	-0.80	0.00	0.77	0.01	-0.45	0.00	-0.46		0.33							
Pemt	phosphatidylethanolamine N-methyltransferase	-0.32	0.02	0.32	0.02	-0.16	0.23	0.29		1.58							
Plpp1	phospholipid phosphatase 1	0.23	0.14	-0.21	0.13	-0.21	0.01			-0.37							
Plpp3	phospholipid phosphatase 3	0.61	0.00	-0.13	0.61	0.56	0.00			-0.56							
Plpp2	phospholipid phosphatase 2	0.26	0.02	0.33	0.00	0.63	0.00			0.44							
Plpp4	phospholipid phosphatase 4	-0.25	0.04	0.02	0.83	-0.33	0.01			1.12							
Plpp5	phospholipid phosphatase 5	-0.05	0.66	0.27	0.01	0.30	0.01			0.18							
Lpin1	lipin 1	1.08	0.00	0.12	0.56	1.03	0.00	-0.06		-1.28							
Lpin2	lipin 2	1.25	0.00	-1.17	0.00	0.84	0.00	-0.73	-0.88	-0.86							
Lpin3	lipin 3	-0.03	0.84	-0.09	0.46	-0.19	0.15	-0.17		-0.27							
Dgka	diacylglycerol kinase, alpha	0.49	0.00	-0.05	0.65	0.49	0.00	0.30		-0.13				Upregulated			
Dgkb	diacylglycerol kinase, beta	-0.49	0.00	0.34	0.03	-0.69	0.00	-0.04		-1.23							
Dgkg	diacylglycerol kinase, gamma	0.95	0.00	0.49	0.02	1.51	0.00	-0.63		-1.12							
Dgkd	diacylglycerol kinase, delta	-0.67	0.00	-0.95	0.00	-0.87	0.00	-0.07		-0.33							
Dgkh	diacylglycerol kinase, eta	0.45	0.03	-0.73	0.00	-0.44	0.02	0.85	-0.87	-2.88				Downregulated			
Dgkk	diacylglycerol kinase kappa	-0.41	0.00	0.06	0.52	-0.32	0.00	0.32		-3.44							
Dgke	diacylglycerol kinase, epsilon	0.23	0.22	-0.18	0.04	0.35	0.00	-0.44		-0.24							
Dgkz	diacylglycerol kinase zeta	-0.26	0.10	-0.50	0.00	-0.69	0.00	0.38	-1.27	-0.73							
Dgki	diacylglycerol kinase, iota	-0.16	0.13	0.02	0.84	-0.03	0.79	-0.86		-2.18							
Dgkq	diacylglycerol kinase, theta	-0.38	0.01	-0.07	0.59	-0.56	0.00	-0.10		-0.93							
etnk1	ethanolamine kinase 1	0.69	0.00	-1.07	0.01	0.87	0.00	-0.01		-0.55				Downregulated			
pcyt2	phosphate cytidylyltransferase 2, ethanolamine	0.37	0.00	-0.14	0.08	0.17	0.04	-0.15	-0.17	0.61							
cept1	choline/ethanolaminephosphotransferase 1	0.56	0.02	0.26	0.14	0.94	0.00	-0.10		0.15							

Anxa7	annexin A7	0.74	0.00	0.13	0.15	0.92	0.00	-0.05	-0.20	-0.72							
Abca3	ATP-binding cassette, sub-family A (ABC1), member 3	0.37	0.00	-0.58	0.00	-0.08	0.56	-0.80	0.46	-0.95				Downregulated			
Pla2g5	phospholipase A2, group V	-0.09	0.34	1.24	0.00	0.93	0.08	1.20		-0.33				Upregulated			
Csf2	colony stimulating factor 2 (granulocyte-macrophage)	-0.12	0.41	0.04	0.79	-0.11	0.48	-0.26		0.44	AgeDown						
Lpin1	lipin 1	1.08	0.00	0.12	0.56	1.03	0.00	-0.06		-1.28							
Lpin2	lipin 2	1.25	0.00	-1.17	0.00	0.84	0.00	-0.73	-0.88	-0.86							
Dgkh	diacylglycerol kinase, eta	0.45	0.03	-0.73	0.00	-0.44	0.02	0.85	-0.87	-2.88				Downregulated			
Dgkd	diacylglycerol kinase, delta	-0.67	0.00	-0.95	0.00	-0.87	0.00	-0.07		-0.33							
Cell marker																	
Errfi1	ERBB receptor feedback inhibitor 1	1.70	0.00	-1.00	0.00	0.88	0.01	-1.49		-0.90				Downregulated			
Socs2	suppressor of cytokine signaling 2	0.74	0.00	-0.43	0.00	0.24	0.02	-0.98		-0.23							
Alcam	activated leukocyte cell adhesion molecule	0.64	0.00	-0.54	0.00	0.54	0.00	-0.70	-0.20	-0.84							
Sin3a	transcriptional regulator, SIN3A (yeast)	0.34	0.04	-0.50	0.00	0.26	0.00	-0.13	0.49	-0.42							
Lgr5	leucine rich repeat containing G protein coupled receptor 5	-0.51	0.01	0.57	0.00	-0.15	0.39	0.32		0.15							
Muc16	mucin 16	0.04	0.87	1.34	0.00	1.05	0.05	0.85									
Muc20	mucin 20	-0.17	0.08	0.48	0.01	0.28	0.07	0.10		-0.25							
pik3cd	phosphatidylinositol 3-kinase catalytic delta polypeptide	-0.98	0.00	1.29	0.02	-0.82	0.00	1.89		0.94				Upregulated			
pik3ca	phosphatidylinositol 3-kinase, catalytic, alpha polypeptide	0.34	0.01	-0.97	0.00	0.41	0.00	-0.24		-0.65							
pik3cg	phosphoinositide-3-kinase, catalytic, gamma polypeptide	0.40	0.06	0.55	0.00	1.21	0.00	0.84		0.11							
pik3cb	phosphatidylinositol 3-kinase, catalytic, beta polypeptide	0.63	0.00	-0.23	0.05	0.41	0.01	0.14		-0.77							
fga	fibrinogen alpha chain	0.36	0.43	1.10	0.02	1.44	0.00	-0.23	0.33	1.50							
fgb	fibrinogen beta chain	0.63	0.17	-0.21	0.60	0.33	0.16		0.36	-3.26		-0.35					
fgg	fibrinogen gamma chain	0.85	0.04	0.94	0.03	2.12	0.00	-0.95	0.38	1.83					1.34		
fmod	fibromodulin	-0.45	0.00	0.88	0.00	1.18	0.04	0.74	-3.67	-0.69				Upregulated			
vcan	versican	-1.42	0.00	0.42	0.00	-1.45	0.00	1.92	0.31	-0.25					1.55		
tead1	TEA domain family member 1	0.16	0.18	-0.44	0.00	-0.68	0.01	-0.74	-0.82	-1.36							
Scgb1a1	secretoglobin, family 1A, member 1 (uteroglobin)	0.17	0.07	-0.06	0.45	0.16	0.04	-1.59	0.37	0.90							
gata6	GATA binding protein 6	-0.29	0.08	-0.28	0.01	-0.36	0.01	-0.67		-0.34							

notch1	notch 1	0.42	0.00	-0.67	0.00	-0.85	0.00	0.05		-1.82							
slit2	slit homolog 2 (Drosophila)	-1.20	0.00	-0.70	0.00	-1.62	0.00	-0.38		-1.18				Upregulated			
fstl1	follistatin-like 1	-1.16	0.00	-0.31	0.02	-1.40	0.00	0.17	-0.21	-0.61							
cdh11	cadherin 11	-0.81	0.00	-0.44	0.00	-1.08	0.00	0.83	-0.55	-0.58							
trpml3																	
mcoln3	mucolipin 3	0.80	0.00	0.06	0.79	0.91	0.02	0.08		0.13							
mmp8	matrix metallopeptidase 8	0.41	0.07	0.69	0.01	1.08	0.02	0.54	0.52	0.66					2.13		
mmp9	matrix metallopeptidase 9	0.44	0.02	1.00	0.00	1.27	0.00	0.94	0.61	1.36							downregulated
mmp12	matrix metallopeptidase 12	0.20	0.24	0.91	0.00	1.31	0.00	0.70		2.76	AgeDown				3.20		
timp1	tissue inhibitor of metalloproteinase 1	0.16	0.51	0.29	0.19	0.36	0.06	-0.84		1.96				Upregula	2.63		upregulated
timp2	tissue inhibitor of metalloproteinase 2	0.39	0.00	-0.21	0.04	-0.78	0.01	0.41		-0.53				Upregulated			upregulated
timp3	tissue inhibitor of metalloproteinase 3	0.71	0.00	0.38	0.04	0.87	0.00	-0.38	1.12	-2.54							
Col10a1	collagen, type X, alpha 1	-0.62	0.00	1.09	0.00	0.19	0.71	-0.50	0.88	1.62	AgeUp			Upregulated			
Col15a1	collagen, type XV, alpha 1	-0.82	0.00	0.49	0.01	-0.53	0.06	-0.71	0.77	-0.32							
Thy1	thymus cell antigen 1, theta	0.89	0.00	1.04	0.00	1.85	0.00	1.93	0.15	1.66							
Ptprt	protein tyrosine phosphatase, receptor type, T	-0.66	0.00	0.24	0.06	-0.56	0.00	-0.81		-1.86							
ORM																	
CLSTN2	calsyntenin 2	-0.63	0.00	-1.52	0.00	-1.43	0.00	-2.37		-0.95				Upregulated			
Scgb1a1	secretoglobin, family 1A, member 1 (uteroglobin)	0.17	0.07	-0.06	0.45	0.16	0.04	-1.59	0.37	0.90							

Supplementary Table S10B

We compared the age dependent gene expression changes of the present study to published findings.

The data are log FC of aged divided by adult humans.

Genes	Human test set		Human validation s	PMID:30814501			PMID: 33982668	PMID:33397975	PMID: 29212667
	Aged/Adult		Aged/Adult	In silico transcriptome		Bulk transcriptome			
	LogFC	p-value	p-value		Protein				
COL1A1	0.37	0.00	0.03	-1.36	-0.08	-0.05			
COL1A2	0.56	0.02	0.01	-1.07	-0.07	-0.42			
COL3A1	0.42	0.02	0.01	-1.24	-0.71	-0.19			
COL6A1	0.59	0.01	0.04	-1.36	0.01	-0.49			Upregulated
COL7A1	0.88	0.00	0.01	-0.84	0.41	0.07		AgeUp	
COL9A2	0.70	0.00	0.01	0.27		3.30			Upregulated
COL14A1	0.63	0.01	0.00	-0.53	-1.45	-0.88			Upregulated
COL15A1	0.58	0.01	0.00	-0.71	0.08	-0.30			
COL16A1	0.84	0.00	0.00	-0.77	4.24	0.06			Upregulated
COL17A1	0.96	0.01	0.04	-0.86		-0.23			
CTHRC1	0.38	0.05	0.00	-0.43		0.39			Upregulated
ELN	0.49	0.06	0.12	-0.33	0.07	0.57		AgeUp	Upregulated
FBLN2	0.53	0.04	0.00	-0.98	0.22	-0.12			Upregulated
AEBP1	0.65	0.01	0.00	0.04	0.21	0.04		AgeUp	Upregulated
EMILIN1	0.60	0.02	0.01	-1.05	-0.09	0.56			Upregulated
IGFBP5	0.65	0.01	0.01	-0.21	-	-0.73			Upregulated
PODN	0.58	0.02	0.00	-0.79	-0.05	-0.58			Upregulated
ZCCHC24	0.39	0.03	0.01	0.33		-0.54			Upregulated
SULF2	0.52	0.04	0.00	-0.49		0.05			Upregulated
PLVAP	0.55	0.04	0.00	-0.23	-0.33	0.47			
CAPN6	0.57	0.02	0.05	-1.54		-2.29			
ITGA9	0.52	0.02	0.00	-0.70		-1.14			Upregulated
VCAM1	0.59	0.03	0.00	-0.28	-0.01	-0.21			Upregulated
CD40LG	0.54	0.04	0.01	1.61		-0.01			Upregulated
IL5RA	0.67	0.02	0.04	2.73		-0.85			Upregulated

CTBS	-0.66	0.00	0.04	-0.38		-0.70			Downregulated
Cdcc80									
OSR1	0.39	0.15	0.05	0.16		-0.27			Upregulated
NYNRIN	0.73	0.00	0.13	-0.57		-2.36			
Dgka	0.55	0.01	0.98	0.30		-0.13			Upregulated
Dgkd	0.70	0.00	0.10	-0.07		-0.33			
Dgkz	0.38	0.01	0.10	0.38	-1.03	-0.07			
Dgki	0.52	0.04	0.71	-0.86		-2.18			
Dgkq	0.73	0.00	0.44	-0.10		-0.93			
ACAN	0.31	0.07	0.26			-0.77			
ADAMTS4	0.43	0.09	0.16	-0.45		0.31	1.08		
ANXA13	0.64	0.02	0.48	1.08		0.02			
BGN	0.34	0.19	0.04	0.01	0.53	-0.30			
COL10A1	0.20	0.17	0.00	-0.50	0.88	1.62		AgeUp	Upregulated
COL18A1	0.65	0.01	0.70	-0.63	1.10	-0.16			Upregulated
COL19A1	0.61	0.01	0.90	-0.50		-2.76			
COL5A1	0.57	0.01	0.10	-0.18	-0.49	-0.28			
COL5A2	0.47	0.06	0.02	-1.07	-0.37	-0.31	1.10		
COL6A2	0.57	0.02	0.41	-1.19	0.16	-0.31			Upregulated
DPT	0.46	0.09	0.00	-0.51	-0.33	-0.12		AgeUp	Upregulated
FBLN1	0.37	0.13	0.00	-0.62	-0.20	-0.03			Upregulated
FBN1	0.36	0.15	0.01	-0.25	-0.26	-0.69			
FBN2	0.82	0.00	0.89	-0.08		-1.69			
FGF9	0.76	0.01	0.57	0.15		-0.32			
HMCN2	0.67	0.00		1.13		-0.15			
HSPG2	0.66	0.01	0.09	-0.79	0.04	-0.89			
IGFBP7	0.02	0.95	0.00	-0.85	-0.97	-0.05		AgeUp	Upregulated
LAMA2	0.26	0.28	0.00	-0.06	-1.15	-0.94			
LAMC1	0.12	0.56	0.10	-0.82	0.24	-1.13			
LTBP2	0.39	0.12	0.00	-0.25	-1.61	-0.30			Upregulated
MGP	-0.16	0.51	0.00	-1.05	3.17	0.60			Upregulated
MMP10	0.60	0.00	0.10	NA		1.67			
MMP11	0.32	0.07	0.05	-0.17		0.18			

MMP12	-1.06	0.00	0.32	0.70		2.76	3.20	AgeDown	
MMP16	0.47	0.03	0.65	-1.17		-1.73			
MMP17	0.66	0.01	0.06	-0.09		-0.30			
NID2	0.63	0.01	0.45	-0.73		-1.24			
NTN1	0.64	0.02	0.63	0.49	0.01	-0.13			
PCOLCE	0.10	0.67	0.04	-0.65	-0.38	0.29			Upregulated
PRG4	0.64	0.15	0.00	0.79		-3.12	1.01		Upregulated
SLIT3	0.23	0.32	0.02	-0.63	-0.18	-1.26			Upregulated
SRGN	-0.79	0.00	0.09	1.15		0.45			
TIMP1	0.21	0.44	0.01	-0.84		1.10	2.63		Upregulated
TNC	0.58	0.02	0.09	1.03	-0.19	-0.02	3.23		
VWA3A	0.83	0.00	0.87	0.16		0.11			Upregulated
PALM	0.55	0.01	0.05	1.40	0.04	0.65			
CFH	0.31	0.24	0.00	0.43	0.41	-0.74			Upregulated
MOXD1	0.50	0.05	0.00	-0.09		-0.03			Upregulated
PDGFD	0.36	0.22	0.00	0.53	-	-0.60		AgeUp	Upregulated
PDE2A	0.45	0.10	0.00	1.54	0.46			AgeUp	Upregulated
THBS2	0.34	0.08	0.00	1.52		-0.92			Upregulated
CCDC80	0.51	0.06	0.14	-0.80		-0.69			
IL6	0.29	0.25	0.06	2.42		0.81	1.07		
CXCL8	-0.45	0.12							
CCL2	0.04	0.89	0.07	1.10		1.41	2.35		
CCL3	-0.60	0.07		1.75		2.58	2.13		
IGFBP2	0.47	0.06	0.00	-0.08	1.09	1.14			Upregulated
IGFBP4	-0.10	0.61	0.02	-0.10		0.23			
Thy1	0.36	0.11	0.02	1.93	0.15	1.66			
Ptprt	0.64	0.02	0.86	-0.81		-1.86			
Alas2	-0.94	0.01	0.91	-0.33		-1.22			
Slc4a1	-0.90	0.01	0.51	3.75	0.58	-1.56			
ORM									
CLSTN2	0.87	0.00	0.18	-2.37		-0.95			Upregulated

Supplementary Table S11
Data retrieval to define marker gene sets for different cells of the lung

A: Cell Marker database (http://biocc.hrbmu.edu.cn/CellMarker/) (http://xteam.xbio.top/CellMarker/)

B: the Mouse Cell Atlas repository (http://bis.zju.edu.cn/MCA/index.html) ---single cell transcriptome

C: the Lung Aging Atlas (http://146.107.176.18:3838/MLAA_backup/) . ---single cell transcriptome
D: the LungGENS database (https://research.cchmc.org/pbge/lunggens/mainportal.html) ---single cell transcriptome- at embryonic day, E16.5-for most data
E: PMID: 33208946

**Mouse-cell-ssGSEA-marker**
AT1： overlapped the two databases (B+C) , then add the data of Cellmarker database (A).
AT2： overlapped the two databases (B+C) , then add the data of Cellmarker database(A).
Basal cells: the data of Cellmarker database (A).
Ciliated cells: overlapped three databases (A+B+C).
Cuboid cells: overlapped the two databases (B+C) , then add the data of Cellmarker database (A).
Goblet cells: combine three databases (A+C+D).
Endothelial cells: overlapped three databases (A+B+C).
Eosinophil ： overlapped the two databases (B+C).
Myofibroblast: the data of Cellmarker database (A).
Fibroblast: combine two databases (A+C).
Alveolar macrophage ： overlapped the two databases (B+C).
Interstitial macrophage: overlapped the two databases (B+C).
Neutrophil: overlapped the three databases (A+B+C).
NK cells: overlapped the two databases (B+C).
B cells : overlapped the two databases (B+C).
CD4+ T cells: overlapped the two databases (B+C).
CD8+ T cells: overlapped the two databases (B+C).
Dendritic cell: overlapped the two databases (B+C) , then add the data of Cellmarker database (A).

When select the single cell transcriptome database, genes log FC would be condsidered> 0.7.

Human-cell-ssGSEA-marker

Club cell: overlap “Nature paper” two cluster (E), add Cellmarker database (A).
Ciliated cells: mainly Cellmarker database (A), consider the “Nature paper” two cluster.
Basal cells: combine “Nature paper” two cluster (E), add the markers of Cellmarker database (A).
Goblet cells: combine “Nature paper” two cluster (E), the other two database do not have this kind of cell (D+E).
AT1: combine “Nature paper” two cluster (E), consider the other two databases (A+D).
AT2: combine “Nature paper” two cluster (E), consider the other two databases (A+D).
Myofibroblast: combine “Nature paper” two cluster (E), consider the other two databases (A+D).
Fibroblast: combine “Nature paper” two cluster (E), consider the other two databases (A+D).
B cells: combine “Nature paper” two cluster (E), consider the other two databases (A+D).
Cd8+ effect: combine “Nature paper” two cluster (E), consider the other two databases (A+D).
Cd8+ navie: combine “Nature paper” two cluster (E), consider the other two databases (A+D).
Cd4+ effect: combine “Nature paper” two cluster (E), consider the other two databases (A+D).

Cd4+ navie: combine “Nature paper” two cluster (E), consider the other two databases (A+D).
NK: combine “Nature paper” two cluster (E), consider the other two databases (A+D).
Neutrophil: “Nature paper” one cluster (E), add the markers of Cellmarker database (A).
Macrophage: combine “Nature paper” two cluster (E), add the markers of Cellmarker database (A).
Capillary: combine “Nature paper” two cluster.

Supplementary Table S12A
List of individual marker gene sets

Mouse immune cell marker

Dendritic	Neutrophil	Alveolar M.	Interstitial M.	Eosinophil	NK	B cell	CD4+ T	CD8+ T
194	78	158	120	67	74	61	62	140
Cst3	Camp	Ccl6	C1qc	S100a9	Gzma	Ms4a1	Cxcr6	Ms4a4b
Naaa	Ngp	Ear2	C1qa	S100a8	Ccl5	Ly6d	Icos	Cd8b1
Plbd1	Ltf	Atp6v0d2	C1qb	RETNLB	Klra8	Cd79b	Thy1	Ccl5
Irf8	Cd177	Mrc1	Pf4	Il1b	Nkg7	Iglc2	S100a4	Il7r
Cd74	Fcnb	Fabp1	Apoe	Csf3r	Klrb1c	Cd79a	Cd3g	Ly6c2
H2-DMa	Itgb2l	Ear1	Ccl8	Clec4d	Klre1	Cd37	Trbc2	Ms4a6b
Ppt1	Cebpe	Ctsd	Mgl2	Il1f9	Klra4	H2-DMb2	Trac	Cd3d
H2-Eb1	Ly6g	Abcg1	Mmp12	H2-Q10	Prf1	Ebf1	Rora	Trbc2
Ckb	Pglyrp1	Cd9	Csf1r	Slfn4	Gzmb	Cd19	Maf	Nkg7
H2-Ab1	Ifitm6	Ctss	Cd74	Il1r2	Klra9	Ighm	Lat	Dapl1
H2-Aa	Adpgk	F7	Fcrls	Ifitm1	Klrg1	Igkc	Skap1	Trbc1
Ifi205	Mrgpra2b	Ltc4s	H2-Eb1	Ccr1	Ncr1	Siglecg	Itgb7	Cd8a
Gm2a	Trem3	Lpl	Ccl12	Mxd1	Klra7	Fcrla	Trbc1	Thy1
H2-DMb1	S100a9	Il18	Fcgr3	Dusp1	Klrd1	Mzb1	Ifngr1	Tmsb10
Psap	S100a8	Lgals3	Folr2	Wfdc17	Klrc2	H2-Ob	Hcst	Ctsw
Tnni2	Mmp9	Krt79	Lgmn	Srgn	Klri2	H2-Eb1	Il2rg	Tcf7
Qpct	Slfn4	Cd44	Ms4a7	Slpi	Klrk1	H2-Ab1	Ltb	Lck
Crip1	Retnlg	Sgk1	Fcgr2b	Tyrobp	Cma1	H2-Aa	Cd3d	Rpl13a
Wdfy4	AA467197	Cebpb	Cd14	Fos	Klra3	Ptprcap	Junb	Rps15a
Bcl2a1d	Serpinb1a	Marco	Maf	Pla2g7	AW112010	Ralgps2	Il7r	Cd3g
Xcr1	Lcn2	Mpeg1	Mafb	Clec4e	Ms4a4b	Mndal	Selplg	Hcst
Ccl17	Mmp8	Plin2	Aif1	Grina	Ctsw	Cd52	Gimap4	Ccr7
Hepacam2	Hmgn2	Hebp1	Stab1	Pglyrp1	Lgals1	Mef2c	Ets1	Rps19
Ccr2	Ly6c2	Tnfaip2	F13a1	Gsr	Id2	Rpl18a	Gimap1	Rps18
Rgs10	Itgam	Cd302	Hexb	Msrb1	Ptprc	H2-Oa	Ms4a4b	Gm10275
Id2	Clec4a2	Cybb	Mrc1	Slc2a3	Il2rb	Ltb	Ptprcap	Trac
Cldn1	Hmgb2	Clec4n	H2-Aa	2310001H17Rik	Dok2	Gimap6	H2-Q7	Rps5
Cxx1b	Lta4h	Pld3	Dab2	Alox5ap	Tyrobp	Syk	Ccr2	Rpl32
Aif1	Ncf4	Ms4a8a	H2-Ab1	Cxcl2	44440	Rpl32	Lck	H2-Q7
Cd52	Anxa1	Klhdc4	Wfdc17	S100a6	Fcer1g	Rps18	S100a10	Rpl18a
Alox5ap	Igsf6	Sirpa	Cxcl16	Junb	Sell	Rps15a	Fkbp3	Rplp0
Havcr2	Rdh12	Axl	C3ar1	S100a11	Ptprcap	Rac2	Cd82	Rps24
Mpeg1	Il1r2	Msrb1	Cfp	Cd300ld	Lck	Rpl35	Lgals1	Rps14
Cd83	Arhgdib	Itgax	Ccl9	Lst1	Tmsb10	Rps7	1-Sep	Cd69
Psmb9	Rac2	Fth1	Pltp	Cd300lf	Ly6c2	Ets1	Samsn1	Rps3
Naga	Fpr2	Tyrobp	Rgs10	Ccl6	Gimap1	Ly6e	Rgs1	Cd247
Cbfa2t3	G0s2	Cidec	Ctsb	Fxyd5	Selplg	Apobec3	Ptpn18	Gimap4
Fgd2	Ffar2	Laptm5	Ctss	Sell	Rac2	Rpl4	Tes	Ets1
Tmsb4x	Stx11	Vim	Igf1	Ifitm2	Hcst	Cnp	Rac2	Lat
Cd24a	Ncf1	Fcer1g	Trem2	Gmfg	Gimap4	Rps20	Tbc1d10c	Skap1
Bcl2a1a	Cks2	Nceh1	Cx3cr1	Mcl1	Serpinb9	Sh3bp5	Coro1a	Rplp2
Pak1	Plaur	Actb	Unc93b1	mt-Rnr2	Cd52	Arhgdib	Shisa5	Rpl36
Klrb1b	Prdx5	Tcf7l2	Nfkbia	Cd52	Il2rg	Sell	Tmsb10	Satb1

Evi2a	Pnkp	Acaa1b	Trf	Zyx	Hsd11b1	Ptpn6	AW112010	Rplp1
Fgl2	Pilrb2	Ctsk	Ninj1	Cyp4f18	B2m	Snx2	Cd52	Wdr89
Irf5	Alox5ap	Alox5ap	Tgfbi	Stk17b	Pfn1	Unc93b1	Cd53	Rpl14
Cxcl16	Ckap4	Snx10	Tmem176b	C5ar1	Irf8	Ddx5	Stk17b	Rps9
Lsp1	Lrg1	Fabp4	Slamf9	Hdc	H2-Q7	Cd69	S100a6	Cd7
Plekho1	Cklf	Cd68	Ms4a6d	Hp	H2-D1	Rpl13	B2m	Rps11
Atox1	Cybb	Fpr1	Pid1	Ncf2	Spn	Hvcn1	Pfn1	Rpl4
Syngr2	Hp	Olr1	Pla2g7	Taldo1	Jak1	Coro1a	H2-K1	Rpl41
Hfe	Mgst2	Psap	Grn	Rac2	Cd48	Foxp1	Rps11	Gm10073
Rab32	Dgat1	Aprt	Arrb2	Gadd45a	Bin2	Dnajc7	Laptm5	Gm7808
Man2b1	Cotl1	Car4	Fos	Pilra	Arhgdib	Il2rg	Rps18	1-Sep
Sub1	Cd52	Dab2	Cd68	Tpd52	Ifngr1	Cd2	H2-D1	Rpsa
Olfm1	Gsr	Cd300lf	Clec4a2	Adipor1	Ppp1r18	Shisa5	Eef1a1	Rps2
Psmb8	Gpsm3	Gal	Apobec1	Fcer1g	S100a10	H3f3a	Rplp1	Ptprc
Sept6	Lst1	Slpi	Tmem176a	Ier3	Cd2	Btg1	Rps15a	Rpl18
Gdi2	Ethe1	Anxa2	Psap	Plek	Ptpn18	4930523C07Rik	Rps24	Eef1a1
Sh3bgrl3	Tyrobp	Bst1	Ccr2	Litaf	Serpinb6b	Tmem123	Rps3	Rpl37
Rnase6	Msra	Bcl2a1a	Clec4a3	Cd53	H2-K1	H2-DMa	Rpl4	Pabpc1
Arpc2	Marcksl1	Lipa	C5ar1	Map1lc3b	H2afz		Rpl32	Rps4x
Fnbp1	Taldo1	Lrp1	H2-DMb1	Cd44	Gimap6			Rps20
Csf2ra	Gmfg	Mertk	Hexa	Lsp1	Sh3bgrl3			Rpl39
Slamf7	Glrx	Spp1	Bcl2a1b	Btg1	Trbc1			Rps26
Clec12a	Pfn1	Cyba	Ctsa	Cdk2ap2	Emp3			Rac2
Phf11b	Hcst	Cd164	Cybb	Spi1	H2-Q6			Rps3a1
Pmaip1	Cyba	Abhd12	Ly86		Laptm5			Rps28
BC028528	Pkm	Gpnmb	Ms4a6c		Ccnd2			Rps7
Actr3	Rbm38	Cstb	H2-DMa		Pycard			Sell
Ptms	Fcer1g	Adipor2	Evi2a		Coro1a			Rpl13
Ucp2	S100a11	Pygl	Marcks		Itgb2			Crlf3
Kctd12	Msrb1	Naaa	Fcer1g		Capzb			Rpl34
Rgs1	G6pdx	Fxyd5	Itm2b		Arl6ip5			Rps21
Vim	Gda	Nabp1	Pld4					Gm9843
Itgb7	Gadd45a	Pparg	Ctsc					Rpl35a
Cyth4	Gpi1	Ptpn12	Atp2b1					B2m
Actb	Pilra	Bcl2a1d	Dusp1					Rpl22
Sdf2l1		Lmo4	Fcgrt					Rps10
Skap2		Blvra	Snx5					Rps15
Camk1d		Gda	Cd81					Cd28
Bcl2a1b		Sh2d1b1	Blvrb					Rpl8
Bhlhe40		Itgb2	Plekho1					Gimap6
Rgs2		Mt1	Pea15a					Gramd3
Cd86		Pla2g15	Serinc3					Rps25
Commd8		Bcl2a1b	Cst3					Il2rg
Fcrla		Serpinb1a	Ftl1					Rpl35
Eef1b2		Csf2rb	Cyth4					Rpl21
Txndc15		Hexa	Clec12a					Sp100
Vrk1		Fcgr3	Kctd12					Gm10076
Ifi30		Txn1	Clec4a1					Ptpn18
Snx3		Plaur	Tmem37					Rps16
Gusb		Taldo1	Pirb					Gm7536

Nfkbia		Trf	Mpeg1					Ptprcap
Tmsb10		Baz1a	Spi1					Eef1b2
Ivns1abp		Ctsz	Il10rb					Rpl6
Dock10		9-Sep	Lst1					Uba52
Hcls1		F10	Asah1					Rpl29
St3gal5		Lcp1	Ms4a6b					Rpl26
Plp2		Cox5a	Ifngr1					Cd2
Tnfaip8		Ctsa	Fth1					Arhgdib
Ptpn18		Reep5	Ehd4					Rpl3
Unc93b1		Slc7a2	Clta					Selplg
Sla		Tlr2	Man2b1					Cd52
Rps11		Tmem14c	Lcp1					AW112010
Ccdc12		C5ar1	Gpx1					Npm1
Pomp		Clec4a3	Tyrobp					Tbc1d10c
Cotl1		Lmna	Alox5ap					Rpl30
Anxa1		Capg	Fyb					Rpl23
Sh3bgrl		Flna	Mef2c					H2-K1
Clic1		Colgalt1	Rgs2					Shisa5
Klrd1		Dhrs7b	Laptm5					Rpl22l1
Atpif1		Cd2	Ctsz					Gm2000
Ppp1r11		Krt19	Cd48					Smc4
Tap1		Tgfbi	Emp3					Atp1b3
Cd48		Sdcbp	Sh3bgrl3					Fyb
Spi1		Csf2ra	Lat2					Eef1g
Fam105a		Sirpb1c	Plbd1					Rpl9
Ifngr1		Pilra	Cyba					Evl
Psme1		Spi1	B2m					Rps23
Efhd2		Sulf2						Dgka
Pabpc1		Ftl1						Gas5
Rps5		Apbb1ip						Ltb
Lcp1		Iqgap1						Bin2
Lat2		Gadd45g						Rpl19
Actg1		Tmed5						Cd53
Vasp		Snx1						Psmb8
Gpx1		Grn						Rpl38
Erp29		Hvcn1						Rps17
Arpc1b		Lpin1						Gmfg
Apobec3		Camk1						Limd2
Cfl1		Gusb						Rpl7
Laptm5		Atp6v0e						Rps8
Pld4		Ncf2						Dnajc15
Anxa2		Pitpna						Tpt1
Mndal		Evl						Rps13
Pitpna		Plgrkt						Coro1a
Brk1		Rassf4						Stk17b
Gm9843		Mgll						Gm8186
Calm1		Comt						Rpl11
Cpne3		Mpp1						
Zyx		Gngt2						
Rpl22		Acp5						

Cytip		Ramp1						
Cdc42		Slc6a6						
H2afz		Rexo2						
Ywhah		Fam96a						
Rps9		Cotl1						
Tes		Ier5						
Rps16		Hmgn2						
Coro1a		Rnh1						
Klf6		Atp6v1b2						
Zfp36		Mcl1						
Mkrn1		Coro1b						
Rps25		Cndp2						
Fcgr2b		Atp6v1c1						
Rplp0		Itgal						
Rpsa		Fcgrt						
Dnajc8								
Rpl39								
Rps4x								
Rps7								
Rpl35a								
Rps3a1								
Rps26								
Rplp1								
Gnai2								
Selplg								
Rpl37								
Rpl14								
Rps18								
Fxyd5								
Arpc3								
S100a11								
Rpl18a								
Rpl32								
Eef1a1								
Rps3								
Rps28								
Rps13								
Rpl3								
Rps23								
Rps15a								
Rpl4								
Rps2								
Rpl13								
Rps21								
Rpl26								
H2-DMb2								
Pfn1								
Itgae								
Itgax								
cd8								

Tlr9								

Mouse pulmonary cell marker

AT1	AT2	Basal	Ciliated	Club	Goblet	Endothelial	Fibroblast	Myofibroblast
78	83	351	144	89	71	57	434	44
Ager	Etv5	Abi3bp	Ccdc153	Chad	Muc5ac	Gpihbp1	Dcn	SMN1
Agrn	Lamp3	Aqp3	Dynlrb2	Scgb1a1	Agr2	Tspan7	Col1a2	Acta2
Ahnak	Sftpc	Dapl1	Sec14l3	Scgb3a2	Dmbt1	Ptprb	Col3a1	Mustn1
Akap2	Slc34a2	Gstm2	Tmem212	Cyp2f2	Gp2	Calcrl	Serping1	Myh11
Akap5	Bex2	Hpgd	Fam183b	Hp	Lman1l	Cd93	Clec3b	Tagln
Aqp5	Cd36	Icam1	Tppp3	Lypd2	Wfdc18	Clec14a	Dpt	AC124170.2
Cav1	Chi3l1	Phlda3	Rsph1	Pon1	Cgref1	Egfl7	Mfap5	AP1
Clic5	Chsy1	Sdc1	Ccdc39	1810010H24Rik	Tff1	Ramp2	Mmp3	Actg2
Col4a3	Cxcl15	2700094K13Rik	Riiad1	Akr1c18	Fkbp11	BC028528	Col1a1	Aspn
Dpysl2	Dlk1	2700099C18Rik	1110017D15Rik	Aldh1a7	Fgl2	Edn1	Serpinf1	Cd248
Emp2	Egfl6	2810417H13Rik	Pcp4l1	Alox15	Tff2	Cdh5	Cygb	Ckb
Hopx	Fabp12	2900026A02Rik	Cyp2s1	Aox3	Lmcd1	Cldn5	Fbln1	Col1a1
Lgals3	Fabp5	Acaa1b	Sntn	Bpifa1	Serpinb11	Tek	Col14a1	Ednrb
Limch1	Fasn	Adh7	1700016K19Rik	Cbr2	Sbpl	Clec1a	Pi16	Enc1
Lmo7	Gabrp	Aldh6a1	Tuba1a	Ccnd1	Ltf	Cav1	Serpina3n	Fstl1
Malat1	Glrx	Alg14	Elof1	Ccnd2	Creb3l4	Gata2	Timp1	Ghr
Msn	Hc	Alyref	1700007K13Rik	Cd200	Qsox1	Slc43a3	Ccl11	Gypc
PDPN	Il33	Amotl1	AU040972	Ces1f	Msln	Plvap	Scara5	Hhip
P2rx7	Lcn2	Anapc5	Hsp90aa1	Cldn10	Bace2	Epas1	Penk	Igf1
Pmp22	Lgi3	Anln	Mlf1	Col23a1	Mfsd4a	Scn7a	Apod	Iigp1
Ptrf	Lyz1	Aqp4	BC051019	Cyp4a12b	Cldn10	Acvrl1	Srpx	Itga9
Qk	Lyz2	Arhgap11a	Ak7	Dcxr	P2rx4	Pecam1	Lsamp	Junb
Rtkn2	Mid1ip1	Arhgap19	Ccdc113	Ffar4	Scgb3a1	Clic5	Gdf10	Mfap2
S100a14	Mlc1	Arhgef39	Traf3ip1	Fmo3	Reg3g	Ldb2	Osr1	Mgp
S100a6	Rab27a	Arl6ip1	Foxj1	Gabrp	Bpifb1	Myzap	Cpxm1	Mllt3
Samhd1	Retnla	Asf1b	Ccdc146	Gsta3	Lypd2	Ctla2a	Lum	Myl9
Sdpr	S100g	Aspm	Hspa4l	Gsto1	Sult1d1	Ace	Loxl1	Mylk
Sema3a	Scd1	Atad2	Aldh3b1	Itm2a	Muc5b	Icam2	Entpd2	Myocd
Timp3	Sftpa1	Atp1b3	Drc1	Iyd	Chad	Cd36	Dpep1	Nnat
Tinagl1	Sftpb	Aurka	Fhad1	Kdr	Pigr	Slc9a3r2	Abcc9	Nrep
Vegfa	Soat1	Aurkb	Spa17	Krt15	Bpifa1	Myct1	Abca8a	Nt5e
Scnn1g	Sftpd	Banf1	Rsph4a	Mgat3	Scgb3a2	Tie1	Igfbp4	Pdgfra
Igfbp2	Napsa	Birc5	Enkur	Mgst1	Fmo3	Tm4sf1	Pcolce2	Pdlim3
Flrt3	Sfta2	Bora	Ccdc181	Mia1	Cp	Cav2	Lama2	Rgs2
Spock2	Dram1	Bub1b	Calml4	Mtus1	Adh7	Flt1	Adamts2	Robo1
Lama3	Lpcat1	C330027C09Rik	Cdhr3	Nrarp	Aldh3a1	Esam	Adh1	Scara5
Pxdc1	Rnase4	Casc5	Tmem107	Nupr1	Cyp2f2	S100a16	Sod3	Scx
Krt7	Bex4	Cbx3	1700024G13Rik	Osgin1	Fxyd3	Cxx1a	Ogn	Smpx
Myo1b	Elovl1	Ccdc3	Stk33	Pir	Gsto1	Ecscr	Clec11a	Sms
Col4a4	Ptprf	Ccna2	Iqcg	Ppap2b	Gabrp	Rhoj	Inmt	Synpo2
Fbln5	Atp1b1	Ccnb1	Efcab10	Rassf9	Scgb1a1	Tspan13	C1s	Tbx5
Ndnf	Wfdc2	Ccnb2	Fam213a	SFTPB	Gsta4	Cd200	Mmp2	Tgfbi
Cryab	Prnp	Ccng2	Nme5	Selenbp1	Cxcl17	Crip2	Rbp1	Tpm2
Sparc	Muc1	Cdc20	Chchd10	Sftpa1	Gsta3	Ly6e	Mgp	Itgb1
Sema3e	Car8	Cdc25c	1110004E09Rik	Sftpd	Slc16a11	Ptrf	Prrx1	
Hs2st1	Cox6a2	Cdca2	Tubb4b	Tacstd2	Wfdc2	4931406P16Rik	Islr	
Fam189a2	Ppp1r14c	Cdca3	Lrrc51	Tst	Aldh1a1	Pde4b	Fxyd1	

Gprc5a	Abca3	Cdca4	Spag17	Upk3a	Cyp2a5	Grap	Fbn1	
Cldn18	Npc2	Cdca8	Vpreb3	Wfdc1	8430408G22Rik	Col4a1	Bgn	
Tmod1	Cbr2	Cdh3	Spef2	Aldh1a1	Retnla	Fkbp1a	Ltbp4	
Fam174b	Cldn3	Cdk1	Ift74	Retnla	Gpx2	Jup	Gpc3	
Slc39a8	Sdc1	Cdkn2c	Meig1	Cp	Tspan1	Cxx1b	C1ra	
Phactr1	Irx1	Cdkn2d	Odf3b	B430010I23Rik	Mgat3	Arhgap31	Nbl1	
Col4a2	Zdhhc3	Cdkn3	Stmnd1	Pigr	Mgst1	Kdr	Pcolce	
Hbegf	Pi4k2b	Cenpa	Dnah5	Gstm2	Cbr2	Cd34	Meox2	
Cadm1	Socs2	Cenpe	Tctex1d4	Gsta4	Ifitm1	Snrk	Cd248	
Hspg2	Lrg1	Cenpf	Pifo	Ldhb	Pglyrp1	Gimap6	Fbln2	
Npnt	Acot7	Cenpl	Aldh1a1	Slc16a11	Gdpd2		Gfpt2	
Scnn1b	Pla2g1b	Cenpm	1700001C02Rik	5330417C22Rik	Hp		Art4	
Scnn1a	Gclc	Cenpn	Iqca	Wfdc2	Cyp4b1		Ecm2	
Mal2	Cpm	Cenpw	Mycbp	Hes1	Por		Gsn	
Clic3	Apoc1	Cep192	Tekt1	8430408G22Rik	Selenbp1		Htra3	
Prdx6	Fgfr2	Cep55	Mns1	Por	Krt18		Fstl1	
Nbl1	Cldn18	Cit	Osbpl6	Gstm1	F3		Rcn3	
Rgcc	Atp11a	Ckap2	Ift43	Pgrmc1	Tmem176a		Medag	
Cyp4b1	Nkx2-1	Ckap2l	Lrrc23	Prdx6	Malat1		Akap12	
Mthfd1	Tmem243	Ckap5	Dnali1	Ces1d	Pon1		Gpc6	
Dag1	Acoxl	Cks1b	1700088E04Rik	Scgb1c1			Cfh	
Igfbp6	Ank3	Cks2	Tcea3	Tspan1			Sparcl1	
Neat1	Tgoln1	Clic1	Ccdc78	Cxcl17			Igsf10	
Tmem37	Abcd3	Cotl1	Bphl	Cckar			Aebp1	
Tns1	Errfi1	Crlf1	4833427G06Rik	Alas1			C4b	
Crip2	Ctsh	Ctdspl	Akap14	Trf			Ms4a4d	
Icam1	Acsl4	Dbf4	Capsl	Aldh2			Col5a2	
Scd2	Itih4	Dck	Syt5	Sec14l3			Olfml2b	
Col4a1	Alcam	Dcn	Gipc2	Cyp4b1			Svep1	
Cyp2b10	Scd2	Defb1	Dnah6	Ephx1			Rarres2	
Pdgfa	Rbpjl	Dek	Ppp1r36	Tmem205			Cped1	
	Atp8a1	Depdc1a	Trp53bp2	Scnn1b			Ism1	
	H2afj	Diap3	Ccdc17	Rbp4			Pdgfra	
	Sdc4	Dlgap5	Rsph9	Gpx2			Igfbp7	
	Atp6v1c2	Dlk2	Wdr78	mt-Nd5			Fgf7	
	Neat1	Dnph1	Tm4sf1	Atp1b1			Serpinh1	
		Dusp1	Ppil6	Pls3			Col5a1	
		Dut	Cdhr4	Gstk1			C7	
		Ect2	Erich2	mt-Nd2			Mfap4	
		Emp1	Lrrc6	Fmo2			Cd34	
		Epas1	Hspa2	Cd55			Timp3	
		Esco2	Cetn4	Krt19			Olfml3	
		Espl1	Aoc1				C3	
		F2r	Agr3				Dkk3	
		F3	Dmkn				Igfbp6	
		Fam107b	1700007G11Rik				Cxcl12	
		Fam111a	6820408C15Rik				Igfbp3	
		Fam64a	Fam47e				Sparc	
		Fam83d	Smim5				Col6a3	
		Fat2	Fbxo36				Lpar1	

		Fbxo5	Clic6				P3H3	
		Fen1	B9d1				Podn	
		Fmr1	Rabl2				Timp2	
		Fn1	Pih1d2				Mmp23	
		Foxm1	D430036J16Rik				Slit3	
		Frat2	Rsph10b				Plxdc2	
		Fxyd3	Csrp2				Ctsl	
		Fzd8	Chchd6				Syn3	
		G2e3	2410004P03Rik				Gas1	
		Gas2l3	Dnaic2				Tcf21	
		Gas6	Ctxn1				Selm	
		Gdpd2	Dcdc2b				Crispld2	
		Gfra1	Efcab1				Colec12	
		Gltscr2	Fam92b				Ddr2	
		Glul	Eno4				Gabra3	
		Gm10073	Nudc				Ndrg2	
		Gm10184	Dynll1				Abi3bp	
		Gm11438	Calm1				Peg3	
		Gm12355	1700001L19Rik				Nox4	
		Gm12504	Dynlt1f				Lamb1	
		Gm12857	2610028H24Rik				Efemp1	
		Gm13160	Dusp14				Rbp4	
		Gm13237	Kcnmb2				Galnt15	
		Gm13827	Zmynd10				Cdh11	
		Gm14633	Ubxn10				C1qtnf7	
		Gm15427	Mcee				Cpq	
		Gm15500	Cmbl				Ndn	
		Gm17541	Cdc14a				Prkcdbp	
		Gm2000	Bok				Vcan	
		Gm20302	Ttc39a				Egfr	
		Gm21596	Ldlrad1				Sfrp1	
		Gm4997	Oscp1				Adamts5	
		Gm5641	Morn5				Lox	
		Gm5805	Ccdc151				Gpx3	
		Gm6085	Ttc12				C2	
		Gm7536	Acsl3				Lhfp	
		Gm8186	Usp18				Atf5	
		Gnb2l1	Fam216a				Fkbp7	
		Gpsm2	Cetn2				Lama4	
		Gsta4	1110032A03Rik				Nid1	
		Gtse1	Cxcl17				Mt2	
		H1f0	Hdc				Fmo2	
		H1fx	Dync2li1				Sdc2	
		H2afv	Vwa3a				Ecm1	
		H2afx	4931406C07Rik				Lgals1	
		H2afz	Morn3				Ifi205	
		Hdgf	Crip2				Klhl13	
		Hirip3					Gpm6b	
		Hist1h2ao					Pam	
		Hist1h2ap					Nr2f2	

		Hjurp					S100a6	
		Hlf					Hsd11b1	
		Hmgb1					Hspg2	
		Hmgb2					Htra1	
		Hmgn2					Ptgis	
		Hmmr					Col6a2	
		Hn1					Fhl1	
		Hn1l					Bicc1	
		Hnrnpul2					Dclk1	
		Hp1bp3					Fgfr1	
		Hspa1a					Ifitm3	
		Hspb1					Il11ra1	
		Id1					Igf1	
		Id3					Slc43a3	
		Igfbp7					Meg3	
		Incenp					Pmp22	
		Iqgap3					Rcn1	
		Junb					Copz2	
		Kif11					Cd302	
		Kif15					Gaa	
		Kif18b					Cst3	
		Kif20a					Mmp14	
		Kif20b					Ntn1	
		Kif22					Ly6a	
		Kif23					G0s2	
		Kif2c					Ifitm2	
		Kif4					Vcam1	
		Kifc1					Ctgf	
		Knstrn					Col4a1	
		Kpna2					Pmepa1	
		Kpnb1					Csf1	
		Krt15					Pdpn	
		Krt17					Plat	
		Krt5					Efemp2	
		Lbr					F3	
		Ldlrap1					Col6a1	
		Lig1					Nfib	
		Lin54					Pid1	
		Lmna					Zcchc24	
		Lmnb1					Adamts1	
		Lsm2					Id3	
		Lsm3					Fn1	
		Lsm5					Cd81	
		Lsm6					Cd63	
		Lsm8					Flt3l	
		Mad2l1					Hmgcs2	
		Mcm3					Vkorc1	
		Mcm7					Mfge8	
		Miip					Apoe	
		Mis18a					Mt1	

		Mis18bp1					Spock2	
		Mki67					1110018G07Rik	
		Mxd3					1810010H24Rik	
		Mylip					1810037I17Rik	
		Ncapd2					2810417H13Rik	
		Ncapd3					3110001D03Rik	
		Ncapg					6030408B16Rik	
		Ncaph					AC107641.2	
		Ndc1					AC119810.1	
		Ndc80					Adamts17	
		Nde1					Abca9	
		Ndrg1					Acap2	
		Ndrg2					Adamts19	
		Ngfr					Adm	
		Notch1					Adpgk	
		Nrm					Agpat2	
		Nsmce2					Airn	
		Ntf3					Angptl4	
		Nucks1					Slf1	
		Nudt1					Anp32e	
		Nuf2					Arl6ip6	
		Nup62					Armcx3	
		Nusap1					Atad2	
		Nxn					Atxn2	
		Nxt1					B3galnt1	
		Oat					B4galt1	
		Olfml2a					BC017158	
		Pbk					Birc5	
		Pcna					Bmp5	
		Pcolce					Bmper	
		Pdzd2					Bub1	
		Perp					Bud31	
		Pif1					Capn6	
		Pik3r1					Cass4	
		Plac9a					Ccbe1	
		Plac9b					Ccdc34	
		Plk1					Ccna2	
		Pmf1					Ccnb1	
		Ppia					Ccnb2	
		Prc1					Cd40	
		Procr					Cdc7	
		Psat1					Cdca3	
		Ptma					Cdo1	
		Ptms					Cenpa	
		Ptn					Cenpe	
		RP23					Cenpf	
		RP23					Cep57	
		RP24					Ces1d	
		Racgap1					Chst15	
		Rad21					Cks2	

		Rad51					Cmklr1	
		Rad54l					Cnot6	
		Ran					Col13a1	
		Rangap1					Col16a1	
		Rasl2-9					Col25a1	
		Rbms3					Cp	
		Rcc1					Csrp1	
		Reep4					Csrp2	
		Rfwd3					Ccar2	
		Rnaseh2c					Dctpp1	
		Rnd3					Ddah2	
		Rnf26					Dennd4a	
		Rpl13					Dhx9	
		Rpl18					Dock4	
		Rpl18a					Dtna	
		Rpl18a-ps1					Egflam	
		Rpl23					Eif4ebp1	
		Rpl26					Emilin1	
		Rpl28					Eml1	
		Rpl30					Enc1	
		Rpl37					Enpep	
		Rpl4					Epha3	
		Rpl7					Evi5	
		Rpl8					Exosc8	
		Rpl9					Tcaf1	
		Rplp0					Fam173a	
		Rps10					Fam64a	
		Rps12					Fancc	
		Rps14					Fblim1	
		Rps15					Fgf10	
		Rps16					Vegfd	
		Rps18					Gfm2	
		Rps19					Gm12924	
		Rps2					Gm14420	
		Rps21					Gm15844	
		Rps23					Gm16559	
		Rps25					Gm22353	
		Rps27					Gnpat	
		Rps27a					Gnpda2	
		Rps27rt					Gpr153	
		Rps3					Gps1	
		Rps4x					Gria3	
		Rps5					Gstm2	
		Rps8					Gstm5	
		Rrm1					Gstm7	
		Rrm2					Gulp1	
		S100a14					Gyg	
		S100a16					H2afv	
		Sae1					H2afx	
		Sat1					H2afz	

		Serpinb8					H6pd		
		Sesn1					Hibadh		
		Sfn					Hist1h2ab		
		Sgk1					Hivep3		
		Sgol1					Hmgb1		
		Sgol2a					Hmgb2		
		Sh3bgrl3					Hnrnpa3		
		Sh3gl1					Hoxb5		
		Ska3					Htatsf1		
		Smc1a					Ifitm1		
		Smc2					Iigp1		
		Smc4					Itga8		
		Snrpd1					Kcnk5		
		Snrpg					Kif13b		
		Sorcs2					Kif15		
		Sord					Kif20b		
		Spc24					Knstrn		
		Spc25					Lbh		
		Sprr2a2					P3h4		
		Sprr2a3					Limch1		
		Srrt					Lipa		
		Stmn1					Lrp1		
		Sult1d1					Lrp3		
		Suv39h1					Lrp4		
		Tacc3					Macf1		
		Tacstd2					Mad2l1		
		Tcf19					Maf		
		Tex10					Mapt		
		Tgm2					Matn2		
		Tk1					Mcm4		
		Tmem167					Mdk		
		Tmem194					Mettl21e		
		Tmpo					Mfap2		
		Top2a					Mis18bp1		
		Topbp1					Mki67		
		Tppp					Mme		
		Tpt1-ps3					Mrap		
		Tpx2					Mrc2		
		Trim59					Mrpl27		
		Tuba1c					Mrpl39		
		Tubb5					Mrpl49		
		Tubb6					Mrto4		
		Ube2c					Mycbp		
		Ube2s					Naa50		
		Ube2t					Ncapg		
		Ugt2b34					Ndufa4l2		
		Uhrf1					Ndufs5		
		Upk1b					Nebl		
		Usp1					Neo1		
		Vangl1					Nexn		

		Xpo1					Nrep	
		Ywhah					Nrk	
		Zdhhc2					Nuak1	
		Zfp296					Nusap1	
							Osbpl5	
							Pbk	
							Phex	
							Pkd1	
							Plekha6	
							Pmf1	
							Plpp3	
							Ppm1k	
							Ppp1r14a	
							Prdx6	
							Prex2	
							Prim1	
							Prkg2	
							Psmd6	
							Ptch1	
							Ptges	
							Pygm	
							Rab34	
							Ralb	
							Raver2	
							Rcan1	
							Rfc4	
							Rhno1	
							Rn7sk	
							Rpa1	
							Rps4l	
							Rrm2	
							Rtn2	
							Scnm1	
							Pnisr	
							Sh3bgrl	
							Shcbp1	
							Sin3a	
							Ska2	
							Slc27a6	
							Slc36a2	
							Slc38a5	
							Slit2	
							Smc2	
							Smchd1	
							Snai2	
							Specc1l	
							Spon1	
							Srpx2	
							Stbd1	
							Stmn1	

							Tacc1	
							Tacc3	
							Taf12	
							Tbpl1	
							Tbx3	
							Tgfb1i1	
							Thumpd1	
							Tiam2	
							Tk1	
							Tmed1	
							Tmem119	
							Tmem38b	
							Tnc	
							Tns1	
							Tns3	
							Top2a	
							Tpx2	
							Trim56	
							Trip6	
							Trp53bp1	
							Tshz1	
							Tslp	
							Ttc17	
							Tuba1a	
							Tuba1b	
							Tubb5	
							Ube2c	
							Uqcr10	
							Utp11l	
							Vldlr	
							Vstm4	
							Wdr60	
							Wnt2	
							Xdh	
							Zim1	
							Zranb1	
							Zyx	

Supplementary Table S12B
List of individual marker gene sets

Human immune cell marker

Dendritic	Neutrophil			Macrophage	NK	B cell	Cd4-naïve	Cd4-effect	Cd8-naïve	Cd8-effect
**133**	**1574**			**503**	**168**	**207**	**210**	**179**	**206**	**177**
CD83	CD11b	CRLF3	LYST	CD68	ZNF683	CD79A	CCR7	IL32	CCL5	GZMK
CD141	CD66b	MPPE1	SGK1	CD14	KLRC2	MS4A1	LEF1	CD2	GZMH	CCL5
CD1a	HNL	PCF11	OSBPL11	CCL18	CTSW	CD79B	TCF7	CD3D	CD3D	IL32
CD1c	FCGR3B	KRTCAP2	MYSM1	CD163	KRT81	LINC00926	MAL	CD3E	IL32	CD3D
CD11c	IL1R2	GTF2IP1	STX4	CD11b	KRT86	BANK1	IL7R	CD40LG	NKG7	CD3E
CD209	CYP4F3	PRAM1	CNPY3	CD11c	XCL1	CD37	CD3E	ZFP36L2	GZMB	GZMA
CD303	S100A9	PTK2B	PHF12	CD80	KLRC1	VPREB3	PRKCQ-AS1	IL7R	CD8A	CD8A
MHC Class II	IFITM2	H3F3AP6	NUDT16	CD86	GNLY	TCL1A	RPL30	CD6	CST7	DUSP2
CCR6	S100A8	SFTPB	LRRFIP1	HLA-DR	KLRC3	RCSD1	RPS14	LINC00892	CD8B	FYN
CD11b	S100P	INPPL1	KLF6	CCL2	HOPX	FCER2	CD3D	TRAT1	CTSW	RPL23A
CD197	CSF3R	ECD	ZFAND3	CD11a	XCL2	HVCN1	OXNAD1	CD52	CD3E	RPS3
CD1b	NAMPT	TRIOBP	ING1	CD16	GZMB	BLK	LINC00861	LEPROTL1	PRF1	RPS29
CD205	FPR1	PEAK3	MAP2K4	CD54	CXCR4	CD19	RPL31	LCK	GZMA	CD8B
CD207	MMP25	TADA3	VAC14	CD64	KLRD1	FCRLA	RPS3A	TMEM173	IFITM1	LYAR
CD273	ADGRG3	YIF1B	NSUN4	CD71	CD7	RPS23	RPS23	SPOCK2	CMC1	RPS27
CD304	G0S2	MKLN1	LRSAM1	CD83	NKG7	PAX5	IL32	PLP2	KLRD1	CD2
CD4	BASP1	CCDC57	EP300	CR3	APOBEC3G	RPS5	RPS12	GPR171	GNLY	IL7R
CD40	CXCR1	CELF2	ARID3B	CXCL10	GZMH	EEF2	LDHB	PRDM1	HCST	RPL27A
CD49d	SLC11A1	GTF2IP4	CDIPT	FIZZ1	CD2	FCMR	RPL32	SIT1	GZMM	SH2D1A
CD80	APOBEC3A	BAP1	SYK	HLA class II	TSC22D3	RPL8	RPS29	SARAF	KLRG1	KLRG1
CD86	MME	DPYD	MAML3	IL-1ra	PRF1	ADAM28	TRABD2A	PBXIP1	CD3G	CMC1
F4/80	SLC25A37	RAF1	PPARD	RFD7	FCRL6	MEF2C	RPS6	CXCR3	HLA-B	PIK3R1
HLA-DR	ACSL1	ARFGAP1	ELF2	phthyl-acetate	C12orf75	CD24	RPS27	IL2RG	HLA-A	LCK
NRP1	MXD1	TGFB1	LPGAT1	FABP4	B2M	FCRL1	RPL23A	PTGER4	B2M	RPL13A
PCD1	FTH1P20	CFLAR	QKI	APOC1	CST7	CD74	DGKA	ACAP1	CD7	SPOCK2
PCX3CR1	LUCAT1	NR3C1	MYH10	C1QB	RBPJ	P2RX5	RPS27A	EVL	RPS3	RPL31
PFLT3	CXCR2	ZC3HAV1	MARS	MARCO	IFITM1	RPL18	ABLIM1	GSTK1	LCK	RPLP2
LGALS2	CDA	MIR29B2CHG	AC016831.4	C1QA	ID2	CD52	RPL13	CD5	FGFBP2	IFITM1
SERPINF1	NCF1C	ARID3A	DNASE1	MCEMP1	ITGA1	GNG7	RPLP2	ITM2A	CD2	RPL41
CST3	CMTM2	SLC12A9	FAM160A2	C1QC	TUBA4A	SPIB	RCAN3	RBPJ	LYAR	CLEC2D
ALDH2	MMP9	LIMD2	PCYT1A	ALDH2	MYL12A	IRF8	RPLP1	CCDC167	KLRF1	KLRB1
GSN	AQP9	CLPTM1L	SIRPB2	FABP5	7-Sep	RPL15	RPL34	HLA-A	C12orf75	LAG3
MS4A6A	C5AR1	ZKSCAN1	PQLC1	LGALS3	GPR171	RPSAP58	RPS2	IFITM1	TIGIT	HCST
CPVL	SRGN	AL732372.2	DEDD2	FBP1	EMB	FAM129C	RPS13	TUBA4A	AES	RPS25
C1orf54	ABTB1	TRPC4AP	CHMP1A	VSIG4	IL2RB	CD72	RPS19	EML4	ZFP36L2	RPS21
ENTPD1	CEBPB	BTBD2	PXK	FN1	GLIPR1	CD22	SELL	TXNIP	RPL27A	HLA-A
S100B	S100A12	LRRK1	CHKA	ACP5	HLA-C	QRSL1	RPL27	OCIAD2	MALAT1	PTPRC
NAPSB	FTH1P10	PRPS1	LAT2	FTL	RARRES3	IL4R	IL6ST	TNFRSF25	RPL23A	MALAT1
HAVCR2	TREM1	SULT1B1	UVSSA	GRN	CHST12	TNFRSF13C	RPS20	PTPRC	RUNX3	RPS14
RNASE6	MNDA	HPS5	DAGLB	SERPING1	RABAC1	ORAI2	CD27	CORO1A	RARRES3	CD27
WFDC21P	TRIB1	PAPOLG	TNK2	CTSD	PHLDA1	CYB561A3	RPL36	CTLA4	SAMD3	ETS1
RAB31	CLEC4E	TBC1D15	ATP6V1B2	MSR1	MATK	POU2AF1	PCED1B-AS1	CLEC2D	FYN	CD3G
S100A10	ITGAX	MIR3945HG	ANKRD11	OLR1	PLP2	RALGPS2	ITK	ITGB1	ISG20	GIMAP4

CTSH	AL512646.1	HELZ2	ARSG	S100A11	TBCD	LIMD2	SERINC5	1-Sep	ETS1	RPL30
GPX1	FFAR2	LYPLA1	NCLN	LGALS3BP	ARL6IP5	TXNIP	RPL35A	PIK3IP1	ADGRG1	RPS6
FGL2	AL627309.6	PLEKHA2	SDF2	MGST3	MEAF6	RPLP0	INPP4B	CD96	RPS27	RPL10A
SGK1	GCA	FBXW11	GOLGA1	ANXA2	CLEC2B	TPD52	RPL19	GATA3	APOBEC3G	PARP8
SNX8	RNF149	RETREG2	NEK7	CYP27A1	SNRPB	SELL	NOSIP	ANKRD12	FCRL6	RPS4Y1
TWF2	LRG1	MAGOH	FAR1	TREM1	CORO1A	CD40	RPL37	RPS3	ARPC5L	PRDM1
NLRP3	FCGR2A	CSAD	CORO1C	LPL	CD53	CXCR5	SARAF	ICOS	PRDM1	RPS28
CXCL16	PCBP1-AS1	PRKAR2A	ZBTB37	CSTB	DHRS7	AFF3	RPS28	RPL23A	RPL3	LEPROTL1
CD74	ALPL	ATOX1	LCP1	MRC1	DAD1	PNOC	RPL13A	KDSR	TBX21	TC2N
SYNGR2	ZDHHC18	FAM200B	CPEB4	TSPO	TBCB	EBF1	PIK3IP1	ARHGAP15	PIP4K2A	BCL11B
VIM	PGLYRP1	C19orf38	TMX4	GPNMB	PCID2	GAS5	RPL3	STK17A	APMAP	SYNE2
PKIB	FOS	GAB2	RALBP1	GPD1	ARHGDIA	LAPTM5	RPS16	SUSD3	SYNE2	GIMAP7
PLSCR1	YPEL3	ANO10	COTL1	ANXA5	SLF1	SNHG7	RPS3	RPS29	CXCR4	CNOT6L
AXL	PHC2	FBRSL1	RAB27A	SERPINA1	PRMT1	IFT57	RPL10A	ARID5B	GUK1	SAMD3
RAB32	RGS2	CSGALNACT1	RGL4	APOE	DUSP2	TSPAN13	IFITM1	RHOH	LAG3	GPR171
SPINT2	VNN2	GCLM	EHBP1L1	RETN	SCML4	SNX2	RPL27A	BCL11B	S1PR5	RPL35A
CD86	FTL	LMBRD1	MXI1	CES1	MDH2	BCL11A	GIMAP7	LDHB	MATK	EOMES
KLF4	LRRK2	PPP1R12B	KIAA0040	DEFB1	MBP	TLR10	RPL5	TTC39C	GTF3A	DNAJB1
LAP3	HLA-B	E2F3	MYH9	PPARG	PIK3R1	STRBP	RPL37A	MZT2A	PTPN4	SH2D2A
GSTP1	MIDN	MPP1	SORT1	INHBA	RAC2	TMEM156	RPL35	TRADD	CHST12	CDC42SE2
PLEKHO1	IRS2	CARD8	FAM217B	GLDN	TNFRSF18	SMIM14	RPL18A	G3BP2	ITGB2	TERF2IP
GPR183	SIRPB1	SELENOO	MSN	SCD	RPS26	ZCCHC7	TRAT1	RPL13A	BIN2	TRAT1
SLAMF8	MCL1	HERC3	AKIRIN2	IFI6	MRPS6	HHEX	RPS15A	PRDX2	7-Sep	RPL36
YWHAH	KDM6B	CCNK	TET2	GLIPR2	CD69	CCR7	NELL2	ETS1	EOMES	EML4
RGCC	DYSF	SH3BP5	VAMP3	PDLIM1	GUK1	CD200	RPL18	CAPZB	RPS15A	SNHG6
PPT1	S100A11	SH3GLB1	BAZ2A	BHLHE41	HLA-E	SNX22	RPS21	FNBP1	CLEC2D	SLC38A1
RGS10	PADI2	GADD45B	ARID5A	GSTO1	COMMD7	TNFRSF13B	RPL12	CCDC107	CALM1	GCC2
CLIC2	MEGF9	DENND6A	TUBA1A	SNX10	CD247	CXCR4	EPHX2	FKBP11	CCND3	PBXIP1
KCNK6	VSIR	TRIP12	CYTH4	FAM89A	GYG1	BCL7A	PCED1B	ARHGEF1	SH2D2A	CXCR6
FPR3	KCNJ15	TMEM71	PISD	NUPR1	LEPROTL1	POU2F2	SATB1	ABRACL	TBC1D10C	TOMM7
MTDH	BCL2A1	MAEA	ZNF641	IFI30	COMMD8	BTLA	CHMP7	RASAL3	SPOCK2	SNRPD2
CCDC88A	MSRB1	CDKN1B	USP32	MME	SOD1	BLNK	EEF1A1	GIMAP7	ANXA6	CD6
CLEC10A	ABHD5	TPM4	GALM	MS4A4A	YWHAQ	C16orf74	RPL11	YWHAQ	HLA-F	RPS19
RAB11FIP1	FTLP3	LINC01001	NFKBIZ	ABCG1	11-Sep	PNISR	FHIT	PRKCQ-AS1	AKNA	CLDND1
PEA15	XPO6	ATP6V1A	SAMD4B	ARRDC4	PLA2G16	OSBPL10	FAM102A	LDHA	DUSP2	RPS20
SLC31A2	VNN3	PRKD2	MRPL28	VMO1	RSRC2	TMEM243	AES	KIAA1551	PYHIN1	CLEC2B
ALCAM	FTH1	LYN	RTF2	CTSL	TMCO1	CDCA7L	RPL10	ITK	MFSD10	MYBL1
SMCO4	SPI1	USP15	SIGLEC14	PNPLA6	RASAL3	PLEKHF2	TESPA1	SYNE2	SSBP4	PCED1B-AS1
FILIP1L	TNFRSF10C	SLC15A4	TYK2	CTSC	RPS3	FCRL2	LDLRAP1	OXNAD1	BTG1	RPL35
H2AFY	DUSP1	RIOK3	SLC36A4	HSPB1	CLK1	CD180	1-Sep	TAGAP	PRKCH	TXNIP
ANXA2	ACTN1	MLX	ZNF394	HP	PRMT2	PKIG	LCK	ANXA6	PTP4A2	TTC39C
RAC1	EMP3	SLC25A11	TAGLN2	CD81	STUB1	DRAM2	RPL41	GALM	SUN2	RPL27
SRGAP2B	DGAT2	EXOC1	SIRPA	DAB2	EAPP	SWAP70	ADTRP	CD28	RPSA	IKZF3
HLA-DPA1	ANPEP	SNORA12	PLEKHM1P1	MYL6	GABARAPL1	1-Mar	RPS15	RPS27	RNF167	TBC1D10C
PTMS	R3HDM4	MOB2	STX6	SLCO2B1	HNRNPDL	STX7	RPL14	AAK1	CAPN2	CD48
SNX3	SLC2A3	PNPLA2	CEP63	HNMT	CDK2AP2	GUCD1	RPS7	FAS	C1orf21	SIT1
DPYSL2	RNF24	C16orf72	FAM177A1	MS4A7	NCAM1	SEMA4B	BCL11B	6-Sep	PARP8	GUK1
CTSZ	CHI3L1	DDX23	FGR	TXN	AMZ2	BIRC3	RPL7	SH2D1A	PPP2R5C	CNN2
ATF3	CATIP	TOPBP1	DHTKD1	OASL	ECI2	EAF2	CD6	ATP6V0E2	EML4	PRKCH
DBI	IL2RG	NPEPPS	RPGR	RGCC	LGALS1	SYPL1	TXK	CDC42SE2	SNRPD2	TMA7

RASSF4	MZT2B	NCOA1	SNRK	CXCL3	PTPRA	NCF1	FCMR	PDLIM2	PPP1CA	APMAP
FCGR2B	ARRB2	ANKRD13A	FBXW2	TREM2	GATA3	EZR	RPL15	UBASH3A	ABHD17A	AES
PPA1	TMSB4XP4	MAP3K11	OSBPL2	RBP4	UBC	PRKCB	NPM1	EZR	SYTL3	MIAT
CEBPD	ATG2A	TMEM164	LINC00513	FHL1	IDH2	FAM3C	MYC	TMEM123	ZAP70	CD5
EPSTI1	NEAT1	FAM192A	ENTPD1-AS1	S100A10	SOCS1	SP110	RPS8	CDC14A	FNBP1	SARAF
IFT20	IGF2R	RAP2C	DBNL	SLC11A1	BATF	ADK	RPL23	ICAM3	MYO1G	ACAP1
C12orf45	PELI1	UBE2B	TCF7L2	TIMP2	CPNE1	LY86	RPL4	N4BP2L2	UCP2	SRRT
LILRB4	SAT1	PILRA	PURB	RAB13	FNBP1	LSM7	RPL28	ARHGDIA	TUBA4A	ARHGEF1
CTSB	NDUFA1	CBWD3	ELL2	ITGB8	ITGAE	LINC01215	THEM4	TC2N	RAP1B	RORA
GRN	STEAP4	MMP25-AS1	CLMN	PLBD1	PTPN22	GGA2	GIMAP4	HINT1	CORO1A	NSG1
LGMN	NCF4	SYNE3	GRK2	FTH1	TBC1D10C	DCK	RPS5	SSR2	CASP8	KMT2E
TXN	VASP	ROCK1	GLA	LTA4H	XIST	NAPSB	TC2N	PBX4	RNF125	STK17A
SERPINF1	SOD2	TFEB	FAM49B	ALDH1A1	CITED2	MBD4	RPL6	SOD1	SPN	ANKRD12
PTMS	SMAP2	ZNF292	DENND5A	VAT1	ADGRE5	ARHGAP24	RPL8	DPP4	CCDC107	BIN2
DAB2	CEBPD	BRD9	MKRN1	PTMS	PGK1	MDM4	RPL39	MEAF6	CDC42SE2	7-Sep
CD74	PHOSPHO1	LAMTOR3	DICER1	MMP19	ST3GAL1	RASGRP2	PASK	MAF	RASAL3	IL2RG
TGFBI	COX7A2	LAMP1	DOCK8	CORO1C	SIGIRR	UCP2	TNFRSF25	CMTM7	IL10RA	SNHG16
LAIR1	AC084871.1	AFTPH	ATXN7L3	COMT	TRG-AS1	SP140	SPOCK2	SLAMF1	SLC9A3R1	PIP4K2A
LAP3	PLAUR	MDM2	PITPNM1	CXCL16	OCIAD2	HSPA4	SNHG5	RCAN3	CD53	TNFSF8
RNASE6	CA4	CCDC18	PHF21A	CTSZ	ARID4B	BACH2	TRAF3IP3	SFXN1	DHRS7	GIMAP1
TUBB6	IL17RA	SLC44A2	IFNAR1	NCEH1	ARPC5L	BCAS4	RPLP0	RHOF	MRPL10	PYHIN1
GPX1	PLIN5	LINGO3	MLF2	ADAMTSL4	CRTAM	YBX3	NDFIP1	BUB3	S1PR4	BUB3
MAN2B1	CEACAM3	RNASET2	QSOX1	LRP1	CASP8	HIP1R	PRKCA	PDCD4	PITPNC1	IL10RA
TPP1	IL18RAP	OGA	AGTRAP	SGMS2	IL7R	TMEM154	RPL38	AKTIP	ADGRE5	CRTAM
MS4A6A	CRISPLD2	CMIP	PRKDC	11-Sep	PSIP1	SP100	PIM1	CCR6	CLEC2B	ZBTB38
PLEKHO1	PYGL	FOXO3	TPD52L2	TGM2	TAF7	ANAPC16	PIM2	CRIP2	GNG2	ARHGAP15
NPC2	BEST1	MECP2	LAMP2	NOP10	CD320	SESN1	RPL13AP5	SLF1	MIAT	IL16
DBI	UBALD2	OTULIN	FHL3	FCGR1A	TSPAN2	SEC62	RPL29	TRAF3IP3	CNOT6L	THEMIS
COMT	HCLS1	RN7SL172P	GRK6	FCGRT	PRDX2	EIF2S3	AAK1	HNRNPLL	ICAM3	TUBA4A
DPYSL2	PFKFB3	GINM1	H3F3B	ALOX5AP	MYL12B	TLE1	TXNIP	LEF1	TTC38	C19orf66
UNC93B1	C9orf16	PCGF3	DYNC1LI1	CYB5A	LINC00892	EIF3D	STMN3	RORA	PTGDR	PDCD4
CTSC	ATP5F1E	EIF4G3	ELL	C5AR1	ARHGDIB	PPM1K	RPL24	CCND2	PIK3R1	RNF166
MGST2	DAPK2	PDLIM2	DEF8	SCCPDH	RGS1	STK17A	SUSD3	SKAP1	BTN3A2	ICAM3
CD4	H3F3A	CDK19	JPT1	APIP	SH3BGRL3	AIDA	DPP4	INPP4B	F2R	RNF19A
SSR3	AC072022.2	CEBPZOS	TNRC18	GPCPD1	RPL7L1	SHMT2	TMC8	OPTN	RAB27A	RPS26
VAMP8	TMEM91	PIP4P1	SNX10	NCF2	CXCR3	BLOC1S2	CMPK1	CNN2	MBP	CCSER2
RNF130	LIMK2	LYL1	OAZ2	SLC31A1	SKAP1	P2RY10	AQP3	CRIP1	OPTN	DENND2D
GNAS	VAMP2	NPTN	APPL2	GAA	SLA2	TFEB	SNHG3	TMX4	HERPUD2	TIGIT
MGLL	NDUFB1	ERICH1	GPR132	PRDX1	SH2D1B	CHPT1	RPL26	HSPA8	PRSS23	CD84
TAGLN2	IRAK3	NHSL2	CD82	ACTN1	NAALADL1	FOXP1	6-Sep	CALM1	A2M-AS1	OPTN
	NBEAL2	MXD3	FBXO33	CSTA	WDR86-AS1	POLD4	GIMAP1	SCML4	ZBTB38	GAS5
	RPL7AP30	RBM5	ZFYVE16	QSOX1	ACTR1B	SCIMP	TOB1	CAPN2	TC2N	CD96
	ATG16L2	UBE2M	LRRC57	SIRPA	HMGB1	TRAF5	ZNF101	TBC1D10C	HENMT1	HERPUD2
	PROK2	ZC3H4	COP1	MGST1	LINC00674	SESTD1	LIMD2	C12orf57	LINC00987	EMB
	RASSF3	WDTC1	FBXL20	CYBB	TMEM173	LBH	HSPA8	PCED1B-AS1	CYTIP	PIK3IP1
	CTSS	RBP7	FAM49A	CREG1	PPP2R5C	DAPP1	EIF3E	RASGRP1	ITGAL	PTGER2
	COX5B	WAC-AS1	MCTP2	S100A6	TPST2	TCF4	RPL9	IL10RA	SH2D1A	RASAL3
	TYROBP	CPPED1	AKNA	TUBB6	TBPL1	GABPB1-AS1	ACAP1	RAPGEF6	ITGB7	GALM
	RNF145	MOB3A	RGS14	CD9	SAMD3	CAMK1D	EVL	SUN2	PFN1	LINC00623
	CPD	HK2	JMJD1C	CTSB	SYNGR1	TRIM38	RPS4Y1	RNF19A	TMEM173	ZAP70

	CIRBP	HEBP2	MSRB2	OSCAR	RRAS2	PARP1	GIMAP2	ARL6IP6	TPST2	SYTL2
	CR1	HDAC7	PPCDC	GLRX	FRMD4B	GLO1	TMEM123	GCC2	PCED1B-AS1	RBL2
	FTH1P3	CDK12	CYB5R4	SORT1	MTFP1	6-Sep	EIF3H	MIAT	PLEKHF1	SRSF8
	AC092746.1	ZNF445	GBP2	CAPG	ZFP36	RASGRP3	TOMM7	UBC	BIN1	1-Sep
	C16orf54	EXOC4	AHCTF1	SLC31A2	KLRB1	LMBRD1	ARHGAP15	SH2D2A	CDC25B	APOL3
	TAPBP	ZNF746	ETS2	HLA-DRB1	PTPN7	CXXC5	TTC39C	CCSER2	1-Sep	CALM1
	KLF2	P2RX1	PPM1M	ACVRL1	KLF6	CHMP7	FBLN5	HCST	ARL4C	APOBEC3G
	MX2	ADAR	HSPBAP1	TFRC	CAPN12	GAPT	TSHZ2	KMT2A	TGFBR3	LINC00861
	PGGHG	RAB11FIP1	GNAQ	TUBA1C	CXCR6	STAP1	RPS4X	TNFSF8	MYL12A	SIGIRR
	AC007278.1	SVIL	MARK2	PILRA	LINC00869	RNASE6	EIF4A2	EPC1	ASCL2	F2R
	CICP27	RSBN1L	NUAK2	PLA2G16	CNOT6L	TBC1D22A	LEPROTL1	ADAM19	TRG-AS1	ITM2A
	AC020898.1	NRF1	FOSB	AKR1C3	LPIN1	PLAC8	ADD3	RNF167	LAT	TAGAP
	AL034397.3	PTBP2	HMGB2	MACC1	TBCC	SMC6	EEF2	THEM4	CCSER2	GIMAP2
	JTB	GABARAPL1	CCDC159	SLC7A7	C16orf54	TAF1D	SRSF5	RNF125	SLAMF6	PTPN22
	TXNL4A	DCAF6	HLA-A	PLXDC2	CD8B	RRAS2	C12orf57	GIMAP4	EMB	PITPNC1
	COX8A	NUMB	TNFSF10	CLIC4	F2R	SEL1L3	UQCRB	AQP3	PTGER2	PTPN4
	SOCS3	RLF	UBE2H	RND3	ETS1	CCDC50	SNHG6	GRAP2	C5orf56	LBH
	6-Mar	WBP2	PDXK	SMCO4	SAMSN1	PTPN6	CD5	TTC19	CD320	LY9
	B4GALT1	TLR8	CD14	CD151	PITPNC1	ST6GAL1	PRDX2	CYLD	SIGIRR	CCDC167
	DHX34	LMNB1	GNB4	LYZ	GZMA	ERP29	SNHG25	SLFN5	SYNGR1	LINC00987
	AC015912.3	CHMP2B	PDZD8	AXL	PPP2R2B	ITSN2	RPS25	ZAP70	KIF21A	FCMR
	H3F3AP4	STXBP5	ERN1	ACO1	ITM2A	FGD2	CLEC2D	SLC4A7	SPON2	GIMAP6
	PER1	ZNF787	UPF1	TCF7L2	RGCC	BLCAP	HINT1	DEF6	LINC00623	GBP5
	BCL6	ATP11B	ITPRIP	HCK	FGFBP2	EIF3L	UBA52	SLC38A1	AAK1	PSTPIP1
	BRI3	RTL8A	IFNGR1	PDXK	ITM2C	ELOVL5	CD40LG	ITGB7	C1orf56	OFD1
	AC026462.1	HIVEP2	C11orf68	RAB31		ZNF581	ETS1	MAP4K1	IL12RB1	STAT4
	RNF213	TARDBP	FBXO38	COPRS		PPP1CC	TNFSF8	STAT4	PRKACB	LIMD2
	CKAP4	SHKBP1	AC027290.2	PGD		TRIM13	SIT1	PSIP1	SLFN5	ARAP2
	CLEC4D	ADAP1	STRN4	SLC15A3		CLECL1	TAGAP	SLC9A3R1	SKAP1	C12orf75
	SELL	RNPC3	SNN	CPE		NCOA3	PLEKHB1	GPR183	IKZF1	SYNE1
	WAS	VAV1	TIMP2	GRINA		KLHL5	IPCEF1	TSPYL2	DOK2	TCF7
	FGD3	TOB1	VPS8	FPR1		MYCBP2	FOXP1	CASP8	KLF2	TSEN54
	FPR2	SAMD9	STAT5B	TNNI2		CAMK2D	SH3YL1	SYTL1	TSEN54	SKAP1
	PTGES3P1	ZNF276	ABCA1	FUOM		QARS	FBXO32	PIM2	PTPN22	GATA3
	B2M1	MED13	STK10	PECAM1		HSH2D	SLFN5	PARP8	CRTAM	
	PDLIM7	PPP4R2	YIPF3	DUSP23		FCGR2B	COX7C	TNFRSF4	IKZF3	
	TLR4	RAB1A	PLEK	TRPV2		NUP88	PSIP1		BTN3A1	
	S100A6	SMIM27	WDR26	CXCL5		RB1	COMMD6		RNF19A	
	NCF2	CCDC186	SULF2	HEXB		SMCHD1	ATM		RORA	
	RPL3P7	CHIC2	ZBTB16	UBASH3B		ATP2A3	BEX2		TOB1	
	PAN3	AC114760.2	ALDH3B1	STAC		RPS18P9	LRRC75A-AS1		STOM	
	NFAM1	VPS39	S1PR4	DNASE2		DUSP22	EIF3F		LSP1	
	SEC14L1	JAK3	PLEKHG3	MT1L		NFATC1	PBXIP1		DGKZ	
	JAML	SQOR	HDAC5	MCOLN1		UPF2	RPSAP58		BCL11B	
	HLX	RNF166	PTPN12	FCGR3A		PNN	BTF3		ABI3	
	COX6B1	LINC01002	PSEN1	FCER1G		PDLIM1	LBH		PDE4D	
	CDK2AP2	CUL9	CAMKK2	FPR2		IL16	GAS5		ANXA2R	
	ARPC4	RNF44	TLN1	HLA-DRB5		HDAC1	SRSF8		RPS4Y1	
	SORL1	TNFAIP2	HK3	CNIH4		KIAA0040	SNHG8		OCIAD2	
	AC087343.1	TLK2	GAA	RAC1		CYSLTR1	ST13		GFI1	

	RNA5SP151	TCTA	BNIP3L	MPHOSPH6		PLCG2	OCIAD2		DENND2D	
	VPS281	PACSIN2	BAZ1A	ARHGAP18		ARID5B	EIF2S3		SIT1	
	SLA	RSBN1	CXCL16	LRPAP1		TNFAIP8	EIF3L		PIK3IP1	
	TLE3	ADAT1	RNF10	FCGR2A		PPP3CC	RAPGEF6		CX3CR1	
	CLEC7A	MYO9B	RNF141	TMEM53		GNB5	ANXA2R		STAT4	
	ARHGEF1	PLEKHO2	RAB7A	UBE2E2		ANKRD44-IT1	CD8B		SYNE1	
	NTNG2	DGCR2	CAMK1D	AGPAT2		IKZF3	BCL2		ITK	
	CALM3	NF1	SNX27	TUBA1B		METTL7A	MZT2A		SLA2	
	AC012368.1	PPP4R1	DNTTIP1	TCEAL4		TRAF3IP3	RSL1D1		GTF3C1	
	ANKRD44	ZZEF1	S100A4	CD58		MYC	LPAR6		CCND2	
	DENND3	SIPA1	MEF2A	ANPEP		SRSF8	AP3M2		CD96	
	FAM129A	ODF3B	AGPAT2	SPATS2L		ADAM19	LINC01089		DDIT4	
	MAP1LC3B2	SLAIN2	SCYL2	DPYSL2		ZBTB20	BIN1		CD6	
	KIAA1324	STARD3	APH1B	GPX4		MARCKSL1	DANCR			
	R3HDM2	GMIP	KAT8	COLGALT1			RPL7A			
	STX3	RBM10	SLC35E3	GSN			HNRNPA1L2			
	ATP6V1F	TRAFD1	MAP2K3	ZBTB8OS			CDC14A			
	AC099489.1	MAP4K4	FYB1	LY86						
	WASHC1	AC009226.1	CNOT11	TMSB4X						
	MGRN1	CYTIP	NSFL1C	TYROBP						
	KMT2E-AS1	PPP2R2A	TBC1D1	ATP6V1B2						
	PLXNC1	ZSWIM8	CTBP2	CCL23						
	ARHGAP9	RNF111	NACC2	IGSF6						
	WASH3P	LINC-PINT		STX12						
	RALB	NUP50		TGFBI						
	ALOX5AP	AGTPBP1		PLSCR1						
	PTGS2	RILPL2		ENG						
	MYO1F	RGS19		PDLIM7						
	RAB5C	PRKRIP1		CCRL2						
	LAMTOR4	DDX19A		RHOB						
	GIGYF1	CRCP		BLVRA						
	AC026979.2	CYREN		TMED5						
	JUNB	THBD		SLC43A2						
	LPAR2	PWWP2A		SDC4						
	MRTFA	QPCT		HCFC1R1						
	RGS18	OGFR		HLA-DQA1						
	MTND5P32	ECE1		ATP6V1F						
	CSNK1D	CD55		ZDHHC3						
	LAMTOR2	JMJD6		COA6						
	TMA7	TCF25		AVPI1						
	NPIPB13	MAP3K3		CD59						
	AC027309.2	GRB2		HLA-DQB1						
	TMEM154	SPAST		PTTG1IP						
	AMPD2	KIF1B		RSU1						
	TNFRSF1A	PPP3CB		LACTB						
	AC107956.1	UBE2R2		PLAUR						
	DOK3	MAPK14		ACER3						
	SAMSN1	TRIM21		CD63						
	RASGRP4	ADAMTSL4		HLA-DRB6						
	G6PD	INTS1		LY96						

	IFRD1	JOSD1		CTSH						
	IFITM3	SUPT6H		ATP6V0B						
	DDX17	CYBC1		ATP6V1D						
	POM121	RBM23		AQP9						
	ADAM8	DGKD		HSD3B7						
	NCF1	NCK2		PLEKHB2						
	CORO1A	DNM2		FLVCR2						
	JUND	SEMA4A		VAMP3						
	C4orf3	KAT2B		CYB5R3						
	PREX1	NUP98		CCDC88A						
	CXCL8	WDR48		SIGLEC1						
	SMCHD1	TNPO2		ACAA2						
	CTSD	TRABD		YWHAG						
	NAMPTP1	PSENEN		ATP6AP1						
	RNA5SP207	CAMK2G		H2AFJ						
	RPS19BP1	EXOC3		ACSL1						
	TMCC3	NECAP1		ANXA4						
	EIF4EBP2	INSR		CD300LF						
	SPTAN1	IL13RA1		CD74						
	TWF2	ARF3		VAMP8						
	RPSAP15	AZI2		YWHAH						
	AC016734.1	TECPR2		PHYH						
	B4GALT5	DDX21		MRPL27						
	SUPT16H	AC008533.1		CDCP1						
	CCNI	MIIP		PSMA6						
	RF000191	MEF2D		OAS1						
	IL1RAP	PHF3		LAP3						
	TP53INP2	C1orf162		UPP1						
	KY	PCMTD1		IL1B						
	GLT1D1	MAP3K8		CTNNA1						
	F8A1	NPEPL1		EIF4EBP1						
	ORAI2	PRKACA		NUS1						
	MAPKAPK2	MINDY1		EDEM2						
	NDUFS6	C22orf39		HTATIP2						
	MKNK2	CIR1		RAB10						
	CCDC88B	SAP30		PTPN12						
	FGD4	UBE2O		SPG21						
	TMUB1	ADD1		GLUL						
	LRP10	FCGRT		KYNU						
	TLR2	AGFG1		NQO2						
	MARCKS	EIF4A3		SCARB2						
	SLC6A6	PER2		OTUD1						
	ULK1	FLOT2		DYNLL1						
	MSL1	RANBP9		RTN4						
	MHENCR	PNPLA8		DSE						
	CYTH1	WIPI2		ETHE1						
	UQCRHL	PCNX3		CBR1						
	SLC16A3	SPECC1		C3AR1						
	NBPF10	VPS37B		PAPSS1						
	HAL	WBP4		MYD88						

	SUN2	AC006001.3		BCL2A1						
	LCP2	RAB24		TXNDC17						
	PPP3CA	MAP7D1		DRAM1						
	PSMA6	CTDSP1		PLIN2						
	TMEM259	AC135983.2		FMNL2						
	PKM	CHMP1B		PRDX3						
	FKBP8	RIPOR2		ADAM17						
	NCF1B	USP25		APOL4						
	SIPA1L2	CBL		TUBB4B						
	BCL3	ZMIZ1		KMO						
	FAM107B	HARS2		COLEC12						
	ARHGAP1	TXNDC9		MGST2						
	RPL12P12	CREBRF		ARL4A						
	AC011472.2	SH3BP2		P2RX4						
	CCNJL	TAZ		ACP2						
	PRR13	N4BP1		PSAP						
	METTL26	HIF1A		NANS						
	ZBTB7A	PPM1B		FUCA2						
	BRD4	USP10		APLP2						
	RTN3	WIPI1		PLD3						
	CEBPZ	MCEMP1		RHBDD2						
	ARHGEF40	CASC3		PCBD1						
	AC003681.1	KMT2C		LPXN						
	ADM	GLIPR2		TUBB2A						
	CREB5	NPL		REEP3						
	AP001000.1	SRGAP2B		MSRB2						
	FTH1P8	ZMIZ2		IGFBP2						
	DNAH17	ATP6V0B		TPP1						
	FAM89B	VPS9D1		SLC6A6						
	ZFAS1	ITGB2-AS1		ATP6V0D1						
	FTH1P7	LPIN2		MFSD1						
	MEFV	DESI1		DENND5A						
	BST1	SLC25A44		GK						
	NDUFB3	MAN2A2		TMBIM1						
	RUBCNL	CD300A		RNPEP						
	AC023886.2	RRP12		IL1RN						
	WDFY3	MFN2		DECR1						
	CNPPD1	KMT2D		CLTC						
	TNFAIP3	ACAP2		RNF13						
	PLPPR2	DYNC1LI2		ATP6V1E1						
	MAST3	WWC3		MAN1A1						
	PPP1R35	RABGEF1		PEPD						
	ZNF638	MAU2		AKR1A1						
	BTG2	ZBTB48		RAB1A						
	TKT	PKN2		NAA20						
	PGD	HGSNAT		PSMB5						
	NOP10	AL353625.1		SLFN11						
	POU2F2	WWP2		SUMO3						
	IGF1R	SERPINA1		UBB						
	TBL1X	SMURF2		RBM47						

	UBE4A	CCAR2		ASAH1						
	RPS19P3	SHC1		UROD						
	HERC1	FAM126B		CHMP5						
	HNRNPUL1	PPP2R5A		AKR1B1						
	P2RY13	TUT7		B3GNT5						
	AC245060.5	SIPA1L1		ORMDL2						
	ZNF518A	SOS2		GTF3C6						
	NFE2	SEC22B		HSBP1						
	ASPH	DIP2B		KAT8						
	EPC1	SERPINB1		LAMP1						
	RBMS1P1	CAT		TRIP6						
	TLR6	PRCP		DST						
	CASP2	LNPK		CNDP2						
	CHST15	IFNAR2		C20orf27						
	POLR2A1	R3HCC1L		OAZ1						
	C4orf48	MAP3K5		DOK2						
	REPS2	NKAP		BID						
	STK38	PIK3CD		MRPS15						
	SLC45A4	MCTS1		VDAC1						
	PFKFB2	ANKRD13D		PSMA7						
	SLC35E2B	ZNF592		RNF181						
	CTDNEP1	APH1A		SDCBP						
	MAX	NUP58		SLC16A3						
	KDELR1	EFHD2		SERPINB6						
	ENTPD1	FCHO2		BLOC1S2						
	CDK11B	CCNL1		SECTM1						
	RBM47	DPEP2		BLVRB						
	C5AR2	STXBP2		CST3						
	KIAA1551	ZBP1		VPS29						
	GNB2	FBRS		HCAR2						
	TOP1	SHISA5		RPL26L1						
	CWC15	GSN		MGLL						
	NAP1L4	NCBP3		HSP90AA1						
	TSTD1	CFP		PLEC						
	CHTOP	SPART		ATP6V1A						
	EMB	EGR1		NDUFB6						
	PI4KA	CAMTA2		ECSCR						
	GNAI2	BRMS1		TNFAIP2						
	CCDC130	DNAJC5		MT1F						
	SNHG3	CCNY		PPT1						
	CSTB	CPSF7		STAC3						
	WASH7P	PTS		TYMP						
	ALDH16A1	CPQ		PYCARD						
	SELENOK	PTOV1		LHFPL2						
	UNC13D	PAK1		THBD						
	MYL6	SLC19A1		TFEC						
	CDC42SE1	IDI1		EMC7						
	SMIM25	CSGALNACT2		GBP1						
	FKBP5	UBE2J2		MOB3B						
	PLK3	TSPO		MDH1						

	ARG1	TMEM33		KLF4						
	DOCK11	NFIL3		PFDN2						
	SPAG9	AREL1		ATP2B1						
	AC044849.1	PAK2		AKIRIN2						
	GOPC	TSC22D4		CD300C						
	AP1G1	AGO4		CCT5						
	PLCB2	HECA		UGP2						
	RC3H1	ZNF33A		CLTA						
	PITHD1	ATP6AP1		MT2A						
	MINK1	MTMR3		HADHB						
	KIAA0319L	PNRC1		IL18						
	GLUL	EXOC6		FCGR1B						
	LMBR1	IQGAP1		DESI1						
	BLOC1S6	ATG13		C15orf48						
	SMIM29	TLE4		ISG15						
	AL080243.2	TOX4		GLA						
	DCTN3	GPAT4		SLIRP						
	ECHDC3	PPM1F		IFNGR2						
	RXRA	ACSL3		BATF3						
	CCM2	JAZF1		MX1						
	PITPNA	FAM168A		AP2S1						
	EVI2B	FOSL2		UBE2L6						
	PTAFR	ATP11A		RHEB						
	ARPP19	PHACTR1		CTSS						
	ANP32A	TBC1D10B		VIM						
	IREB2	MARCKSL1		LAMTOR2						
	BOD1L1	WDFY4		GRB2						
	SKI	EPN1		CCL20						
	GABARAPL2	KMT2B		CTSA						
	SNHG9	VMP1		FDX1						
	HIPK1	DEF6		RASGEF1B						
	GAK	MIATNB		EFHD2						
	PTMS	UBAP1		TKT						
	TYMP	CREBBP		GPX1						
	SETX	ETF1		SLAMF8						
	CMC2	NSMAF		POMP						
	CD93	THEMIS2		NR1H3						
	SECTM1	RPS91		DNAJA1						
	SDCBP	TAF12		MYDGF						
	IVNS1ABP	KCNE1		GPX3						
	SMG1	KLHL8		HBEGF						
	CCDC71L	FRMD4B		CXCL2						
	AC005261.1	ATP2B1-AS1		C1orf162						
	BLCAP	YKT6		SYNGR2						
	PPP3R1	GATAD2A		ATP1B3						
	ZNF467	C1RL		REEP5						
	FKBP1C	MSL3		ENY2						
	BTG1	TMUB2		TAGLN						
	FMNL1	NUFIP2		SLC3A2						
	CCDC69	RBCK1		CHMP2A						

	HSPA6	NFE2L1		S100A4						
	CSRNP1	SYS1		MT1E						
	HCK	CABIN1		EIF6						
	ATF4	TRAPPC9		ACADVL						
	GRN	UBE3B		ABL2						
	HLA-E1	TAOK1		LAMTOR1						
	ARHGAP45	KLHL2		TAGLN2						
	ATP2B4	PCNX1		TMED10						
	HIPK3	CYBA		BAX						
	C3orf86	ERV3-1		PSMD8						
	RPS6KA3	UBXN2B		SFT2D1						
	FURIN	ADIPOR1		YBX1						
	CBWD5	SP3		TMEM219						
	NIPBL	SPATA13		COX17						
	ARAP3	ASAH1		IL1A						
	LAMTOR5	NDEL1		GNB2						
	BET1L	USP19		DYNLRB1						
	KREMEN1	HSH2D		RNH1						
	RASSF2	TPRG1L		GNG5						
	LHPP	DDX60L		MT1G						
	RAC2	RNF144B		FKBP1A						
	DDX27	PRKAR1A		LGALS1						
	SRGAP2C	AC018638.5		RPS27L						
	AC078819.1	ARRDC1		SNX3						
	AC004687.1	IER2		BSG						
	VPS4A	HSDL2		ATP6V0E1						
	HECW2	MKNK1		PRDX5						
	GDI1	C1orf56		NPC2						
	RAD21	DIAPH1		PSMB6						
	MBD1	SLC9A8		VDAC2						
	PFKFB4	C3orf62		RAB7A						
	MT-TL1	SRPK1		CXCL8						
	AC015871.3	NRDC		RHOA						
	AL132989.1	PSTPIP2		TALDO1						
	HERC4	KDM5B		TNIP3						
	EMC10	MAP3K2		LRRFIP1						
	KIAA0513	KDM7A		TNFAIP6						
	LGALS8	GGA3		SOD2						
	RBMS1	UPP1		PEBP1						
	SEC23B	LAPTM5		MT1X						
	AC023157.3	ATXN1		SAT1						
	ARHGAP26	UNC93B1		PRR13						
	HRH2	WDR59		ARPC3						
	NABP1	NSUN5		DBI						
	SCAF11	DUSP23		ARPC2						
	SSH2	NCOA2		PGK1						
	LINC02256	PRAF2		GSTP1						
	SLTM	SFXN5		SH3BGRL3						
	PTPA	RB1CC1		LDHA						
	DOCK5	NOTCH1		PFN1						

	PHF20L1	ZNF7		FABP3						
	KDM4B	UGGT1		ENO1						
	ARHGAP35	CLK3		TMEM59						
	CARHSP1	PECAM1		HSP90AB1						
	RAB11B	MMGT1		MT1M						
	APOBR	IRF1		IL6						
	ARAP1	ZFAND2B		CXCL1						
	STAU1	BRAF		MYL12B						
	FBXO21	ERGIC1		PPIA						
	BRD8	SH3GL1		CALM2						
	RTN4	ERLIN1								
	RTF1	LINC-PINT1								
	TGOLN2	ZMAT2								
	NSD3	FES								
	SETD5	NFE2L2								
	HIST1H2AC	USF2								
	NLRP12	MICU1								
	IL1RN	TFDP1								
	AC243960.2	PIK3CA								
	RF000171	GNA13								
	ISY1	NRBF2								
	MTCH1	PAF1								
	AL356273.3	UBXN6								
	SMG1P5	ACSL4								
	H2AFX	LBR								
	AP5Z1	WDR13								
	MAPK3	CYSTM1								
	MT1X	WDR55								
	TNIP1	CCDC9								
	ISG20	GANC								
	BAZ2B	RUNDC1								
	RHOG	FRY								
	TOMM5	ZNF493								
	PPP6R2	TTPAL								
	GLYR1	RNF138								
	CXorf38	ZCCHC2								
	NUDT22	RANBP3								
	JMJD8	IFIT3								
	PDE4B	MBD2								
	PRPF38B	NFIC								
	AFF1	SLMAP								
	LSP1	CUL4B								
	IRF2BPL	SRGAP2								
	NRBP1	GNAI3								
	UBE2A	MYLIP								
	BNIP2	NARF								
	PRDX5	TP53I11								
	PIK3R5	IFIT2								
	OSM	WSB1								
	MIS18BP1	DDX3X								

	KLHDC3	EHD1								
	IRAK1	LINC01410								
	UBN1	DYRK1A								
	YTHDC2	BID								
	PRR14	NCOA4								
	WIPF1	ARSA								
	TXN	FAM193B								
	PPP1R15A	TAF8								
	YY1	RARA								
	PRKAG2	LTBR								
	STK17B	CHMP4B								
	CCNT1	PXN								
	FAM234A	FRMD8								
	AURKAIP1	NEDD9								
	RN7SL368P	RAP1GAP2								
	ADGRE5	SLC7A5								
	STX16	ITPR2								
	MED25	SLCO3A1								
	ORAI3	IP6K1								
	TNFRSF1B	SCAP								
	SLC8A1	NFKB2								
	PDPR	ZNF516								
	SEMA4D	CHD4								
	JDP2	CHD7								
	STAT3	FAM76B								
	EXTL3	ITGAM								
	IFFO1	APMAP								
	NINJ1	ITPRID2								
	ALPK1	STK40								
	UBE2E3	LRMP								
	IQSEC1	FAM120A								
	IDS	UBA1								
	PLEC	DCAF10								
	ZNF281	RPS6KA5								
	IRAK4	MVP								
	TRIM25	COQ7								
	CHCHD2P9	FAM214B								
	FGD5-AS1	MED13L								
	POLR2J	SIRT7								
	HNRNPH2	ANXA11								
	CUX1	PSD4								
	PLBD1	CASP10								
	ERO1A	KIAA0232								
	NLRC5	STK17A								
	CNOT4	GOLGB1								
	SKAP2	MAP1LC3B								
	LRRC47	CASS4								
	STX10	TRAPPC8								
	FAM157C	SPRYD3								
	CRTC2	B3GNT5								

	WDR11	RCOR3								
	CBX4	PLCG2								
	PCGF5	RIN3								
	ARHGAP4	MBP								
	AC093752.1	KCNQ1OT1								
	KIF13A	RNF130								
	FKBP15	TUBA4A								
	PTPRE	RFX3								
	AP000936.3	PGS1								
	IL6R	TOM1								
	OGFRL1	PTBP3								
	TSSC4	CYHR1								
	RAPGEF2	KIF21B								
	TALDO1	CDC34								
	MMP24OS	ABHD2								
	TENT2	IRF2								
	CTDSP2	GPR160								
	TBKBP1	TMEM127								
	YTHDF3	TXNRD1								
	LINC00937	PRKCD								
	ZNF580	ARHGAP30								
	AP5B1	WAC								
	PET100	ADGRE2								
	CSF2RB	CENPBD1P1								
	FKBP1A	AP3B1								
	AL513327.1	IL4R								
	RAB31	NADK								
	NISCH	MCOLN1								
	ASAP1	GK								
	SP110	RERE								
	F5	SPG21								
	FOXN2	MED11								
	FAM168B	ITM2B								
	PPP1R9B	MAFF								
	MAN1B1	SLC12A6								
	GMFG	MTMR6								
	OAZ1	VAPA								
	PYCARD	YPEL5								
	UBE2F	TNFSF13B								
	HK1	ZNF217								
	PGLS	STAG2								
	AL445524.1	EPS15L1								
	SF1	SMG7								
	RUNX1	NQO2								
	LITAF	TLR1								
	MBD6	ZNF672								
	IMPDH1	MED16								
	MED28	GAPT								
	BSG	BCKDK								
	TCIRG1	ATP6V0A1								

	PACS1	IL18R1								
	GRINA	GPCPD1								
	CLDN11	MPZL3								
	LINC02207	PSTPIP1								
	ZFP91	CAPN1								
	MIB2	ABCF3								
	PRRC2B	CLASP1								
	GRB10	LPCAT2								
	STAT6	FRAT1								
	TMEM120A	GTPBP1								
	SERTAD2	CIC								
	TTLL3	KDM5C								
	LGALS9	UBE2D1								
	PNPLA6	PICALM								
	ZER1	CLIP1								
	RELT	MYADM								
	PIKFYVE	UBR2								
	PRPF3	AC020916.1								
	LSM14A	NAA60								
	BAX	CARS2								
	ABCA7	STXBP3								
	ZNF611	SYAP1								
	SMG5	C15orf39								
	H2AFY	SLC8A1-AS1								
	SP2	MAP4K2								
	JARID2	MOSPD2								
	RNPEPL1	ITGA5								
	CLASRP	ADAM19								
	FNDC3B	PJA2								
	TRIM8	BABAM2								
	ZFP36	FRAT2								
	TMCC1	NADSYN1								
	ACOX1	TFE3								
	HAX1	ACTR2								
	ARL6IP6	RAD23B								
	SKIL	PARD6B								

Human pulmonary cell marker

AT1		AT2	Basal	Ciliated	Club	Goblet	Capillary	Fibroblast	Myofibroblast
922		156	520	311	147	224	176	364	203
AGER	CUX1	SFTPC	ACKR3	ACAD11	SCGB3A2	AGR2	IL7R	RARRES2	ASPN
EMP2	RAB25	NPC2	ADH7	AGBL5	CYP2B7P	FAM3D	FCN3	RGCC	CLU
CAV1	DYNC1H1	SFTPA1	ANXA8	AGR3	MGP	S100P	EDN1	A2M	CFH
RTKN2	PMVK	NAPSA	ANXA8L1	AHSA1	RPLP2	CYBA	SLC6A4	GPC3	WIF1
MYL9	C9orf3	SFTPA2	AQP3	AK1	RNASE1	WFDC2	TMEM100	PTGDS	SCARA3
SPOCK2	C5orf38	CTSH	ARL4D	AKAP14	SFTPB	CYP2F1	DUSP6	LUM	VIM
ANXA3	CTNNA1	PGC	ATP1B3	ALDH3A1	CYB5A	VMO1	GPIHBP1	FN1	DPT
S100A4	EIF1B	SFTPD	BMP7	ALDH3B1	VIM	MSMB	CA4	MACF1	TGFBI
IL32	ANG	LAMP3	C10orf99	ALDH9A1	HSD17B11	TSPAN8	NOSTRIN	PMP22	TSPAN8
TIMP3	PMEPA1	ABCA3	C16orf74	AMZ2	CTSE	MDK	CD14	SPINT2	LTBP1
S100A10	TMEM14C	CHI3L2	CAPG	ANG	C16orf89	SPDEF	RNASE1	CFD	CD9
TSPAN13	AAMDC	CA2	CAV1	ANKRD37	RPL27A	CLDN10	BTNL9	LIMCH1	RPS2
RGCC	SCAMP2	DBI	CCND2	ANKRD65	RPL34	CCNO	NTRK2	MFAP4	BCHE
CLDN18	BCAP31	SERPINA1	CD109	ANKRD66	RPL26	SERPINB3	CLEC3B	FMO2	TNC
CAV2	BLVRA	WIF1	CLCA2	AP3M2	RPL21	XBP1	TNFSF10	CYR61	LTBP2
TNNC1	SKIL	LRRK2	CLCA4	APOBEC4	RPS29	FUT6	TPM1	CES1	PAMR1
LMO7	HNRNPA0	C11orf96	CLDN1	APOO	RPS19	MUC16	TXNIP	TIMP3	POSTN
UPK3B	IRS2	NRGN	COL17A1	ARHGAP39	RPS14	LCN2	CD36	FGFR4	FGF18
CLIC3	LPIN2	PLA2G1B	CSTA	ARL3	AADAC	ASS1	CALCRL	FBLN5	LINC00632
SCEL	ARL8A	HHIP	DAPL1	ARMC4	RPS23	ST6GAL1	RGCC	FIBIN	RPL12
SLC39A8	TACC2	PEBP4	DSC3	ATF3	SFTA1P	C15orf48	GATA2	INMT	PCSK1N
KRT7	ATF4	CPB2	DSG3	ATPIF1	RPL24	CTSC	LRRC32	EMP2	RARRES1
CYP4B1	HLA-C	NNMT	DUSP7	AZIN1	RPL35	WFDC21P	ENG	SCN7A	ANGPTL2
AQP4	ERRFI1	MFSD2A	FABP5	B3GNT7	RPS24	CXCL17	HLA-C	MAMDC2	RPS3
RAB11FIP1	MAP2K3	SFTPB	FABP5P3	B9D1	RPL39	CHP2	EPAS1	DST	ANGPTL6
CD55	RCAN1	CHIAP2	FAT2	B9D2	CST3	EPS8L1	MALAT1	WNT2	RPS4X
KLF6	PIM3	CRTAC1	FBLN1	BASP1	RPS28	ERN2	EGFL7	ADH1B	COL3A1
GPRC5A	DERA	MALL	FGFBP1	BBS9	AGR3	BPIFB1	RAMP3	MYH10	ATP1B1
PCYOX1	ADPRHL2	MID1IP1	FYB	BSCL2	RPS15A	DHRS9	HLA-DRB1	CDH11	ITGBL1
KRT18	AVPI1	SLC22A31	GJB3	BTBD3	HOPX	LGALS3	ADGRF5	ZYX	RPL10
GAS6	KIF5B	FTL	GLTP	C10orf107	RPS21	PAQR4	FRMD4A	SRGN	VCAN
CD151	PEX2	P3H2	GM2A	C11orf49	RPS18	S100A16	ADGRE5	GDF10	TYRP1
MYL12A	SH2D4A	AK1	GPC1	C11orf52	RPL32	CXCL6	BAIAP2	SLIT2	TNFRSF19
MGLL	CTSZ	LHFPL3-AS2	GPNMB	C11orf88	RPS27A	FUT2	HSPB8	S100A4	LXN
ACTB	ACTR1A	C4BPA	GPX2	C11orf97	RPL37	ARPC3	KIAA1217	G0S2	TGFBR3
APLP2	PDZK1IP1	C16orf89	HSPB1	C12orf75	KIAA1324	GMDS	ADRB1	SAT1	ROBO1
RDX	UBE2A	CACNA2D2	IFI27L2	C14orf142	STEAP4	BIK	ARHGAP18	TCF21	RPL18A
PLLP	ZDHHC12	HLA-DRA	IGFBP6	C15orf26	MET	CHL1	COL4A2	QSOX1	S100A13
CLIC5	STX3	TMEM243	IL1RN	C16orf71	RPS6	ST6GALNAC1	RALA	MYADM	RPS6
SEMA3B	TNFAIP1	DRAM1	IL20RB	C17orf97	RPS3	MUC4	TIMP3	OLFML3	RPL26
SBDS	TMEM11	LPCAT1	ITGA6	C1orf189	RPS3A	B3GNT3	PDE4B	CTGF	PRELP
CD47	GRAMD1A	TMEM163	JUP	C1orf192	S100A13	NDRG2	MAOA	FHL1	DKK3
ANOS1	KMT2E	CXCL2	KRT13	C1orf194	SLPI	MISP	SMAD6	COL13A1	S100A10
SPARC	EXPH5	HHIP-AS1	KRT14	C20orf26	RPL35A	GAPDH	NDFIP2	LBH	HS3ST3A1
PDLIM2	PEBP4	CD74	KRT15	C20orf85	FOLR1	FUT3	EMP2	HSD11B1	SLITRK6
VEGFA	CCND3	ETV5	KRT16	C21orf59	RPL10	CRABP2	BST2	LTBP4	LGALS3
HPCAL1	TMEM43	SLC34A2	KRT17	C2orf40	RPL31	SLC31A1	CMTM8	ITGA8	VCAM1

LIMCH1	FNTA	DMBT1	KRT4	C2orf81	FABP5	PLEKHS1	CD44	MMP2	RPL13A
CADM1	VKORC1L1	HLA-DRB1	KRT5	C4orf3	TPT1	LY6E	ARGLU1	SLC38A5	TMEM100
CYR61	DDA1	RASGRF1	KRT6A	C5orf15	RPL12	TNFSF10	CFLAR	CCBE1	CYSLTR1
NCKAP5	SQSTM1	NECAB1	KRT6B	C5orf49	RPL5	MARCKSL1	PLLP	SLC1A5	ALDH2
SDCBP	SPAG9	SELENBP1	KRT6C	C9orf116	GDF15	GALNT6	CD58	RGS3	PSTPIP1
EPCAM	DGCR2	SDR16C5	LAD1	C9orf135	RPL13	PSCA	CDH5	SLC40A1	COMP
ICAM1	DNAJA1	TFPI	LGALS7	C9orf171	RPS12	GNE	COL4A1	ZFP36L2	MUC12
FOLR1	SLC3A2	RND1	LGALS7B	C9orf24	RPS8	SORL1	ARHGAP29	CDO1	ARRDC3
HBEGF	KRT19	CD36	LOXL4	C9orf9	RPL13A	MESP1	CD109	ANGPT1	LUZP2
HOPX	SESN1	FABP5	LY6D	CALM1	RPS27	SERINC2	LDB2	USP53	RPS15A
UNC13D	LAMB3	MUC1	LYPD3	CALML4	ARHGDIB	HES4	PRPSAP1	LIMS1	MDFI
MYO1C	WNK1	RGS16	MMP28	CAPS	RPL11	ABCA13	LUC7L3	EMILIN1	RPL10A
MYL6	CTSL	ALPL	MT1X	CAPSL	RPL23A	SLC51A	DUSP1	ANXA1	LINC01436
ARHGEF26	ALS2CL	ALOX15B	NOTCH1	CARS	CMAHP	SLC5A8	FDPS	TMEM176A	EEF1A1
WFS1	NPNT	LRRC36	NRARP	CASC1	SOX4	STEAP1	ITGA6	ALDH1A1	CNN3
SUSD2	NDRG1	KCNJ15	NXN	CAST	RPL37A	SLC9A3R2	NUTM2B-AS1	PGAP2	FN1
B2M	SERPINH1	CSF3R	PGM2	CBY1	MS4A15	OAS1	TMEM165	PRELP	SFTA1P
EFEMP1	ZFYVE21	SCD	PHLDA3	CCDC103	RPL29	SLPI	CD93	TMEM176B	TMEM98
CEACAM6	SLC25A4	LGALSL	PKP1	CCDC104	RPL23	SERPINB4	HLA-F	METTL7A	F2R
PHLDB2	MYO5B	LANCL1-AS1	PKP3	CCDC11	CRACR2B	HS3ST1	WARS	PLEKHH2	TSHZ2
C12orf49	SERTAD3	PPP1R1B	PLAT	CCDC170	RPS13	BACE2	GPR146	LGMN	PDGFC
TNFRSF12A	DNASE2	SLC46A2	PLP2	CCDC176	MRPS25	KRT4	ZNF302	CELF2	ROBO2
PRSS8	SDC4	DCXR	POLR2J3	CCDC181	RPS20	NANS	OSMR	LRRN3	RPSA
CD9	PPP4R1	C3	PPP1R13L	CCDC33	PIGR	IDH1	VIPR1	COL6A3	GREM2
EPB41L5	PGRMC2	NFKBIA	PTTG1	CCDC42B	RPL30	DNAJC12	ACSL3	ZNF106	DIRAS3
DUSP1	SLC7A6	BMP2	PVRL1	CCDC60	RPS5	ATP12A	FOXN3	BMP5	RPS3A
NDNF	PLP2	DUSP6	RAB38	CCDC65	CXCL17	CARHSP1	BMPR2	NPNT	WDR91
EFNA1	RHOG	SLC6A14	RARG	CCDC74B	RPL14	GLIPR2	TEK	SGCG	CCDC146
ARPC5	TP53I3	HLA-DRB5	S100A10	CCDC81	RPS11	RHOV	STX12	EPHX1	RPL3
GGTLC1	FAM129B	AREG	S100A14	CCL15	RPS15	CYP2J2	HMBOX1	ELN	FXYD5
MT1E	MED10	GKN2	S100A16	CD59	FAU	GOLM1	LPAR6	SVEP1	ENC1
C1orf198	YWHAG	CAT	S100A2	CETN2	RPL38	RHOC	ACVRL1	LAMA4	CTHRC1
AHNAK	INF2	EDNRB	S100A8	CIB1	CEACAM6	CRACR2B	FENDRR	ADAMTS8	RPL7
TNS1	RAB9A	KCNJ8	SDC1	CKB	RPL36	VTCN1	ECE1	DUSP1	NTM
COL12A1	TMEM259	MSMO1	SERPINB1	CRIP1	RPL18	CP	PON2	LMCD1	LRRC75A-AS1
FHL1	FAM189A2	KIAA1324L	SERPINB13	CST6	RPL18A	ST14	CLEC14A	DUSP6	FXYD6
VSIG2	RHOBTB2	SNX30	SERPINB2	CTBS	FAM129A	NUCB2	PARP14	CD14	COL1A1
COL4A2	TMEM9	ETV1	SERPINB5	CTF1	RPS7	STX10	RDX	F11R	NPC2
MYADM	FAM167A	PARM1	SFN	CTGF	HSD17B13	TMPRSS4	LAP3	OR7E47P	GAS5
ITGA3	LBH	ZNF385B	SH3BGRL3	CTSS	CIT	GLRX	LIMS2	SPTBN1	COL12A1
NEDD9	SRSF2	FASN	SOX15	CTXN1	GPRC5A	SLC15A2	NXF3	LITAF	GALNT18
DSTN	IRX2	FBP1	SPINK5	CWH43	SSR4	PSMB10	AKAP12	DKK3	FHL1
PLS3	ERGIC1	HMOX1	SPRR1A	CYB561A3	RPS16	B4GALT4	CDC25B	LINC00968	THBD
ITGB1	MPRIP	CITED2	SPRR1B	CYGB	ERP27	DTX2	RNF144B	ABI3BP	COL16A1
FSTL3	CTGF	PLD3	THBD	CYP4B1	SLC22A3	CREB3L4	ARRDC3	NEAT1	TPD52L1
ABCA1	SREK1IP1	PMM1	TIMP1	CYSTM1	RPL27	A4GALT	RASIP1	CARHSP1	FGF14
MYL12B	TNS3	CDC42EP1	TMEM43	DALRD3	DAPK1	BCAS1	FGFR1	PLXDC2	RPL5
CST6	ATP13A4	ODC1	TNS4	DAW1	RPL19	MUC5B	CFDP1	TMEM119	SYNDIG1
TUBB6	RIN2	ORM1	TP63	DMKN	AREG	SLC16A3	NOTCH4	CD82	MMP2
ANXA2	RB1CC1	SPRY4	TRIM7	DNAAF3	RPL15	GALNT7	FOXF1	CYP4B1	CXCL14

CAPN2	KLF10	SMAGP	TUBB6	DNAJB2	LTA4H	RDH10	ADD1	FBP1	MXRA5
NKX2-1	DAB2IP	ACADL	UPK3BL	DNAL4	HSD17B6	GALE	CPNE2	SRGAP1	CIRBP
RNH1	ACAA1	B3GNT8	WNT10A	DNPH1	SERP1	CD82	TMEM88	TSC22D3	RPL4
CXADR	SULT2B1	AGPAT2	ZNF385A	DPCD	SNHG6	PPIC	HLA-DQB1	FEZ1	NOTUM
MS4A15	FSTL1	ESAM	ZYX	DRC1	WSB1	HMGB3	SEC14L1	LSAMP	GEM
FADS3	TMSB4X	ASRGL1	MIR205HG	DYNC2LI1	HNMT	VILL	LY6E	ITGA2	NNMT
EHD2	NIPSNAP3A	LPL	SERPINF1	DYNLL1	BCAM	ECE1	CTSL	S1PR2	USP18
MT1X	UBTD1	QDPR	FHL2	DYNLRB2	AARD	ADGRF1	JAM3	SFTA1P	KCTD12
TAGLN	SMAP1	CISH	IGFBP2	DYNLT1	RPL41	IFI16	OPHN1	ROBO2	DANCR
CPM	LRRN4	MTRR	HNRNPA1	EBNA1BP2	TMSB4X	RARRES3	SPATA13	MME	RPL15
ANKRD29	AP1S3	CHI3L1	RPL3	EEF2K	RNF145	LGALS9	GIMAP8	NAV2	HSBP1
TPM4	BRI3	LGMN	MPZL2	EFCAB10	ATL2	GPX8	ACER3	ABCA6	CTGF
ITLN2	ARPC1B	CD44	IFITM1	EFHC2	TM4SF1	IGFBP3	PODXL	PIEZO2	GABARAPL2
RHOA	HEXA	S100A14	NPM1	ENDOG	ATP1B1	COMTD1	CLEC1A	MCOLN3	HOXB6
COL4A1	TMCC1	MSN	RPLP1	ENKUR	ZFP36L1	PTN	SH2D3C	PRG4	C2CD2
UBC	OSBPL9	MLPH	DLK2	ENPP5	PTGS2	SLC4A11	MAST4	GPM6B	CCDC68
CFL2	GLS	SOCS2	MYC	ERF	CRTAC1	KRT7	C1orf54	MOXD1	RAB34
GPM6A	ABHD2	GSTA4	RPL10A	ERICH2	TIMP1	TIMP1	GALNT18	MAOB	AREG
RAB32	SCNN1B	EP300-AS1	SOD3	ERICH5	VAMP5	AKR1C1	KIAA1549	BAG2	LAMP5
FLNA	NFE2L2	TTN	RPL4	EZR	FXYD5	OAT	IFNGR1	SLC44A1	ARHGDIB
PAPSS2	BAIAP2	ACSL4	NGFR	FAM104B	RGS16	CRELD2	ZBTB8A	KANK4	TSC22D1
CFLAR	ECEL1P2	ZDHHC3	TINAGL1	FAM154B	SOCS2	MANF	SERPINB9	PALLD	EEF2
MYO1B	ANGPTL4	HP	LDHA	FAM166B	FAM20A	PODXL	BTG1	SAMHD1	STXBP6
CRYAB	SEMA3E	PID1	LAMB3	FAM174A	RPL13AP5	SRD5A3	SORBS1	MDFI	HOXB5
SFTA1P	CKB	AQP1	RPS7	FAM183A	SEL1L3	CMAS	LEPR	NRP1	C1QTNF7
PNPLA2	IFI35	HSD17B4	RPL5	FAM216B	FCGR2A	SPINT1	AKR1C3	TGM2	BGN
TSPAN4	PIEZO1	SNX25	RPL14	FAM229B	MGST1	HMGA1	XAF1	CD44	COL1A2
LRRFIP1	GOLGA8A	FGG	GAPDH	FAM81B	SFTA3	RHBDD3	ESYT2	TAGLN2	PBX3
LEPROT	GPRIN2	C1orf21	BTF3	FAM92B	XIST	CXCL1	ISG15	PPM1K	CTSK
ATF3	CD81	SPTSSA	GPC3	FBXO15	FBP1	CTSB	SPNS2	KCNS3	FOXP1
ABLIM1	SRSF3	AKAP13	DKK3	FBXW9	MALL	SORD	GIMAP1	CNN2	CREB5
SULT1A1	H3F3B	FMO5	RPL13A	FOCAD	WWTR1	LINC00342	TTC28	CD8A	WNT5A
RNASE1	JUND	FDPS	RPS6	FUZ	PAEP	MAGED1	THBD	UACA	TCF12
MAP7D1	DUSP3	RAB27A	ETS2	FXYD3	CEBPD	REEP3	RAMP2	SLC51B	TAGLN2
TGFBR2	TPRA1	BLVRB	BCAM	GALC	COMMD6	SLC25A39	STC2	DAAM1	EXPH5
LAMA3	RALA	RBPMS-AS1	PRNP	GJA9-MYCBP	FNIP2	KLF4	ACSL5	ITGBL1	C16orf89
TMOD3	10-Sep	CHCHD7	RPS8	GPR162	FLRT3	TGM2	GPER1	PDLIM2	ENPP2
CALM2	SLC25A37	STC1	RPS5	GPX4	PEG10	CYB561D2	DNAJB1	GYPC	PLSCR4
SGCE	PDCD6IP	TIFA	ERRFI1	GRAMD2	NEDD4L	TPI1	HIF3A	EFCC1	MFAP2
RRAS	ATP2B4	GEM	ZFP36L2	H2AFJ	FMO2	PDIA3	PDGFB	FMO3	EMP3
TBC1D2	LAMA5	SAT2	EEF1A1	HBB	AHR	ARL1	IL18R1	IGSF10	PAM
EPS8L2	CALCOCO1	STEAP4	RPL15	HEATR2	IL6ST	ECHS1	PPARGC1B	AOC3	SPON2
SPRYD7	PRDX6	SLC25A5	RPL7	HIPK1	TOP1	SCNN1B	TLE4	PTGER4	DUSP10
ARL6IP5	NT5DC1	TMEM41A	MACROD2	HIST1H1C	RBPMS	FAM114A1	NEAT1	ENPP2	ST3GAL4
SLC40A1	BCL2L1	POLR2C	ALDH3A1	HIST1H2BD	FAM129B	RND3	MCF2L	LRP1	PRPS2
SEMA5A	FMO2	LTA4H	APOD	HIST2H2AA3	CLIC6	AKR1C3	JADE1	FHOD1	FILIP1L
PTPN1	OPN3	TPD52L1	RPS3A	HIST2H2AA4	KLHL24	MTFP1	LHFPL2	EMID1	RCN3
PON2	SNX14	ENO1	HCAR3	HMGN3	PELI1	FAM129A	HOXA5	TSPAN4	TBX3
MATN3	ITFG1	CKS2	RPL32	HN1L	SGMS2	ZNF428	GBP4	WSB1	CD34
PGM1	ANKS1A	CHP1	NSG1	NRNPUL2-BSC	MMP7	CCDC167	APLNR	SLC29A1	NFIC

DPYSL2	SLC15A2	CD83	YBX1	HRASLS2		MSLN	PALMD	SERPING1	LDLRAD4
SIK1	RANBP17	SERPINB1	ENO1	HS3ST6		SLC12A2	GADD45B	CITED2	COL8A1
GJA1	GADD45B	AZGP1	YBX3	HSP90AA1		TMED3	IL4R	XIST	CD200
UBL3	VEZT	SOD2	SYT8	SPB2-C11orf52		PAM	CLIC5	IL15RA	ALCAM
HCFC1R1	TMEM189	CSF3	ACTG1	HSPBP1		SLC6A14	SLC44A1	NEBL	COX4I1
PDPN	7-Mar	NFKBIZ	PABPC1	IFI27		UAP1	KCNQ1OT1	ENPEP	THY1
HEG1	TUBA1C	CXCL3	IGFBP7	IFT22		LIMA1	KLF4	DOCK4	PDGFRA
TES	HERPUD1	PLIN2	HMGB3	IFT43		WNK2	SULT1A1	ATP6V1B2	CD82
ISG20	HSPB8	MED24	EIF3L	IFT46		GTF3C6	MBOAT2	ARL6IP5	PDLIM4
SESTD1	ARHGAP29		RPL13	IFT52		STAP2	ID1	PLA2G5	USF2
SDC1	DOCK4		PERP	IFT57		SLC4A4	WDR60	FILIP1	CADM3
MALAT1	PLXNA1		EIF3E	IGFBP7		PTGES	CD300LG	PDGFRA	PAPPA
ADRB2	CAT		EEF2	IK		FBXW5	ITGA2	ELMO1	RCN2
LMNA	RBCK1		MT2A	INO80B		FAM3C	ZNF331	VWA5A	PDLIM3
SP100	AHR		CH25H	IQCE		QARS	IFI44L	NTNG1	MYL6B
TSPAN15	CSRNP1		SCPEP1	IQCG		PDLIM5	CD274	BMP4	PTGER1
CCDC107	IFNGR2		RPL8	IQCH		ALG3	STXBP6	COLEC12	STARD10
CYBRD1	DAD1		FBXO32	IQCK		PDHB	SLCO2A1	COL8A1	NEDD9
PARP14	TMEM63B		NACA	ISCA2		SEC13	ERRFI1	CASP12	JAM2
SCD5	MT1M		RPS2	JOSD2		KDELR2	TMEM37	MEOX2	FLRT2
DAPK2	SLC39A13		EIF1	KCNE1		CLINT1	AFF3	HNRNPLL	COTL1
SPINT2	NBEAL2		RPS9	KCNRG		CAPN13	IFIT1	AKAP9	RARRES3
CLTB	RAB6A		F3	KIF19		P4HB	EFNB2	CYP7B1	PHLDA3
F11R	PTPRF		RPS3	KIF9		GPAA1	ADM	S100A16	IMPDH2
RASSF7	APTR		CYP24A1	KLHDC9		GNA15	GIMAP7	MCOLN2	LAYN
COL4A3	LAMC1		RPL26	KLHL13		GSTA1	EFNB1	SERPINE1	FKBP10
FN1	HDAC7		RPL6	LAP3		UGP2	BEX5	VEGFC	PKIG
NEDD4L	EZR		RPS4X	LINC00094		CHMP1B	GJA4	PNISR	FIBP
SNAP23	BSDC1		SERBP1	LOC158960		EHF	RGS5	ADARB1	SNHG8
FARP1	TCEA3		SLC1A5	LOC388780		HDAC1		CHPF	PRDM6
RBP1	CLIP4		RPS24	LOC644172		BPIFA1		UCP2	BASP1
PLEKHA1	PKDCC		RPL7A	LOC645638		COPB2		LMO4	DAP
CYSTM1	TRA2A		RSL1D1	LOC728392		IDH3G		CYB5A	HLF
CXCL16	JOSD1		KCNN4	LRRC10B		FKBP11		FAS	LTBP3
TNFRSF1A	PPA1		IL33	LRRC23		RRBP1		CAMK2N1	ELOVL1
ARAP2	IGSF8		CDH3	LRRC34		FAM173A		MAL	COL15A1
TOR1AIP2	TMEM245		LRRC75A-AS1	LRRC45		CMPK1		TMEM108	MDK
HSD17B6	KPNA3		RPL9	LRRC46		PGD		PTGIR	HSD11B1
WWC2	ABTB1		IFNGR1	LRRC6		RAMP1		JUN	CMTM3
TIMP2	CTDSP2		JUNB	LRRC73		PIGR		MRC2	CXCL16
B3GNT2	CORO1C		RPLP0	LRWD1		SYNGR2		TMEM37	HMGN1
PLEKHO1	KLC1		TSC22D1	LXN		ALDH2		CYP3A5	LOXL1
STX12	SLC6A4		PALLD	LYPLA2		C3		DPT	SEMA3C
CRIP2	RAB5B		RPL11	LYPLA2P2		LRRC26		ABCA8	PID1
PRKCZ	TMEM115		PKM	MAPK8IP1		MGST1		HMCN1	EPDR1
DLC1	NXN		RPL10	MAPRE3		SDF2L1		ZDHHC14	UACA
ARRB1	MGST3		TNFRSF12A	MGMT		ARFIP2		PGM3	EIF3F
SPINT1	MGAT3		RPL29	MIA		PDIA4		TNFRSF1A	CTSB
MAP2	DIRC2		RPS15A	MLF1		HDLBP		HERPUD1	RAB32
ACTG1	HIRIP3		SPOCK3	MORN2		MYDGF		ARHGAP20	HTRA1

KRT8	NAV2		GPR87	MORN5		ANXA1		NCAM2	FKBP7
TMEM50A	IFNGR1		ARG2	MSRB1		SH3GLB1		PKN2	FHL2
TXNDC11	ANKRD37		VSNL1	MTSS1L		RFK		FAP	CALCOCO2
CNN2	TANC1		MCL1	MYCBP		OSTC		FOXO3	MPDU1
TMEM160	TLN1		LGALS1	NAT14		PSMD7		GRIA1	OSBPL9
COL8A1	SUSD6		JAM3	NHLRC4		DNAJC3		LPAR6	SERPINE1
UTRN	WSB1		RPL12	NME5		TMED10		PTPRS	
PDXK	SH3RF1		ISYNA1	NME7		RPN2		RHBDD2	
DST	HYAL1		RPL36A	IPHP3-ACAD11		TPM1		TANK	
PLEKHJ1	RAB24		RPS20	NQO1		SLC26A2		GFOD1	
MYRF	CD40		PDLIM1	NSMCE1		HSPA5		TMEM9	
NEBL	ZDHHC20		RPL18	ODF3B		BZW1		OLFML1	
SPTBN1	SULT1A2		AXL	ORMDL2		AK2		CSTA	
DENND3	RSRP1		PDPN	PACRG		RPL18A		APLP2	
MT1G	SHTN1		UBC	PAIP2		STOML2		AGTRAP	
HSPG2	CLDN4		FOS	PIFO		SAT1		HMGN2	
PARD6B	OFD1		SNCG	PIH1D2		RER1		COMT	
PCBP1	APOL6		SNHG8	PIH1D3		PDIA6		ULK4	
EVA1A	CHCHD6		TGFB1	PITPNM1		NDUFV2		SNHG8	
RAB31	LARP4B		ADH1C	PLA2G16		EIF6		HEG1	
CLDN7	SLC35A1		RAB34	PLAC8		ARF4		ADM	
ABCA7	ZDHHC9		RPSA	PLEKHB1		TMED2		CAB39L	
GKN2	LIMS2		DST	PNMA1		CST3		NABP1	
ORMDL1	C10orf67		RPS14	POLR2I		RBM3		PPFIBP1	
YWHAH	MICAL2		ABI3BP	PPIL6		PKM		MLXIP	
RAB14	NBN		HNRNPA0	PPP1R14C		CSTB		COL6A2	
AGRN	FKBP9		ALDOA	PPP1R7		NTS		CMKLR1	
TMEM9B	C5orf15		RPS16	PRDX1				CTSC	
RTN3	LRRC1		CXCL8	PRDX5				RCAN1	
S100A6	STXBP6		LMNA	PRKAR1A				TNFRSF12A	
TPM3	SDC2		ID1	PRPS1				PHACTR2	
ALCAM	KLF7		SRSF2	PSCA				TTC14	
LPCAT3	CRIP1		RPL21	PSENEN				ISLR	
SFT2D1	CDS2		SLC3A2	PSMB10				IRS2	
HEBP1	CNPPD1		EVA1C	PTPLAD2				PAPSS2	
SERINC1	INSIG2		ITM2B	RBKS				COL6A1	
GRK5	PRR5L		RPL22	RHPN2				EMP1	
ATOH8	PARP12		STK17A	RIIAD1				DPYSL3	
NDST1	WNT7A		LRRC8A	RITA1				COL6A6	
TACSTD2	ITPKC		ITGA2	ROPN1L				LIMA1	
NUDT9	DAG1		CFH	RPA3				LAMC1	
ELOVL5	FOXA2		RPL18A	RRAD				VGLL3	
CELF2	SNX18		RPL24	RRAGA				PLTP	
QKI	RASSF8		GAS5	RSPH1				NUAK1	
ITM2B	ABCA8		TNFRSF21	RSPH4A				KIFC3	
TM4SF1	BTG2		DUSP2	RTDR1				DYNC2LI1	
TSPAN12	B4GALT1		SOCS3	RTP4				ST8SIA1	
STARD7	ATP6V1H		IKBIP	RUVBL1				CYP27A1	
ANXA4	IFNAR2		SGK1	RUVBL2				RSPO1	
CDKN2B	ITSN2		MRPS6	SAA2				HSPB8	

TMEM109	KLK7		LIMA1	SAA2-SAA4				BAMBI	
MRPL14	RORA		RPL19	SAA3P				AKIRIN2	
CSRP1	UBAP1		IMPDH2	SAA4				TM2D2	
MYH9	ARL13B		CD9	SCGB2A1				P4HA2	
OTUD1	TP53BP2		PIM1	SDHC				GJA5	
FXYD3	NDN		TACSTD2	SLAIN2				SERINC5	
COMT	ACOT9		OAT	SMIM22				CHIC2	
PRDX1	SLC44A3		RAN	SMYD2				TSPAN9	
FERMT2	NR4A1		RPL13AP5	SNTN				DAB2	
RHOBTB3	ANKLE2		C1R	SOD1				TRIP10	
CNN3	BIN1		NCL	SPA17				FGF7	
PPFIBP1	VAMP8		RPL22L1	SPAG16				CASP4	
P4HA2	SLCO3A1		RPS11	SPAG6				SH3PXD2A	
FAM107B	ID1		CLEC2B	SPC25				EDNRA	
GALK1	MID1		SERTAD1	SRI				MXRA8	
RNF213	CPEB4		CAPNS2	SSB				LIFR	
RHBDD2	ZBED2		RSL24D1	STMND1				TECR	
CCDC12	FOXO3		SFPQ	STOML3				HM13	
SLC1A1	ANXA11		CNBP	STX2				SYNE1	
DEGS1	CCBE1		CALD1	STYXL1				CCDC102B	
STK17A	KCTD9		WDR43	TCTEX1D2				KCNK6	
PTPRE	PAX8-AS1		TMEM173	TCTEX1D4				AKAP13	
LAPTM4A	MCL1		HSPD1	TCTN1				SGCD	
PTTG1IP	ST6GALNAC2		FAF1	TEKT1				LUC7L3	
CGN	SLC7A7		TMEM237	4SF19-TCTEX1D2				DDX17	
SBSPON	RBMS2		EGR1	TM9SF1				SNCA	
SCARB2	SERINC5		ADRB2	TMEM107				FOS	
PAPSS1	CARD16		ITM2C	TMEM190				CERS2	
ROR1	PPL		PMAIP1	TMEM231				ARL4D	
LLGL2	PER1		CD177	TMEM254				IL6ST	
FBXO7	ZC3HAV1		AHCY	TOMM34				IP6K2	
WBP2	TGIF1		KRT16P3	TP53TG1				PER2	
ASAH1	DUSP7		TPM1	TPPP				LMAN1	
COL4A4	PXDC1		TNIP2	TPPP3				CD36	
TJP1	PEAK1		ARID5B	TRIM32				EPHB6	
EDN1	MPP5		RPS26	TSPAN1				HSPB3	
ANXA5	EDEM1		TPM2	TSPAN19				ST3GAL4	
VPS29	LNX2		LUZP1	TSTD1				DNAJC1	
ALDH3A2	ZFYVE9		JUN	TTC25				ARGLU1	
SLC2A3	LRP5		STOM	TUBA1A				CDH13	
NTM	TMEM246		IFI16	TUBA4B				MMP19	
STX7	ATXN2		NDFIP2	TUBB4B				MAOA	
OSGIN1	ARHGAP24		DUSP23	UCP2				ASPA	
EHD1	EFR3B		HNRNPK	UFC1				AMOTL2	
SRGAP2B	SERTAD1		EFEMP2	UGDH				TNFAIP6	
AUP1	CCND1		EIF3D	ULK4				DNALI1	
TACC1	LTBP4		TRIP6	UNC119B				TMEM246	
C4BPA	TMEM41B		LITAF	VIM				PEMT	
GABARAPL1	RNF128		LPAR6	VRK3				ETV1	
MPC1	LMTK2		RGS12	VWA3B				PTGS2	

RNF13	IDS		EIF3H	VWA5A				ADAM17	
STX11	MYH14		BASP1	WBSCR27				IL16	
UBE2B	ITGA2		CLU	WDR16				KCNQ1OT1	
CTSA	WNT3A		NAP1L1	WDR38				TPM3	
VKORC1	PTPN21		DUSP1	WDR54				GSTM5	
CAPZA2	MEGF9		DDX21	WDR66				CCDC8	
LAMB2	IFRD1		PRMT1	WDR78				LOXL1	
NDFIP1	MPPE1		EMP3	WDR86-AS1				ENG	
UXS1	AKAP11		SLC43A3	WRB				HIVEP3	
HSD17B11	HACD1		LTBP4	ZBED5-AS1				DHRS3	
MSLN	PTGS2		SLC38A2	ZDHHC1				SH3D19	
REEP5	USP54		RPL23	ZMYND12				MCUR1	
TRAM1	APOL1		LPCAT2	ZNF688				SLC38A2	
ATP6V1E1	ADGRF5		NPM3	ZNHIT2				SLC35A1	
C5orf24	SPRED1		SNHG15					NEDD9	
BIRC3	NUMB		TUBA1C					FXYD6	
MTCH1	SNX21		ANXA2					MAP2K3	
LAMC2	TRIP10		C9orf3					ICAM1	
ST3GAL4	TSC22D2		CXCL1					BNIP3	
TMEM125	ZC3H7A		TCF4					LEFTY2	
N4BP2L2	EMP1		HCAR2					AASS	
CHMP1B	ITPR3		LDHB					BOK	
SSR2	DDIT3		CEBPD					TMEM138	
N4BP1	ADAM10		PHLDA1					GRINA	
IL18	TSPAN7		TGIF1					FSTL3	
DNAJB1	RASSF1		TM7SF3					SERPINB9	
IRF1	KLF2		CYCS					CACUL1	
ERBB2	KIFC3		EDN1					FAM3C	
SERPING1	SGMS2		ALDH3A2					ANG	
PDGFA	DHCR24		9-Sep					CFAP20	
C19orf33	TEAD1		ARPC2					C5orf15	
FILIP1	CD63		G0S2					ZFP36	
TMEM179B	ARL4C		TPI1					HBEGF	
PHACTR2	B3GNT7		ITGB4					PPP1R15A	
LGALS3BP	PROS1		CTNNB1					C1orf198	
HYAL2	GRB7		BAG3					SKIL	
CHPT1	COBL		HMGA1					HTRA3	
MT1F	SCAI		PGAM1					SLIT3	
CERS2	TRIM47		CTSB					ADAMTS2	
LSR	ACKR4		EPAS1					STEAP2	
OSGIN2	ACSF2		NAMPT					ARSI	
CAPZB	AGTPBP1		FOSL1					PIK3IP1	
LAMP1	SAMD4A		TRPV4					TPCN1	
GGT1	POLR2L		HNRNPDL					PEAK1	
PFKFB3	FKBP1B		KLF4					EFHC1	
SYNGR2	EYA4		PPA1					TRIM47	
SPTAN1	SMURF1		EFNB1					EPB41L2	
ID4	FGD4		HAS3					ANO1	
GNAQ	TOM1L2		DHRS3					LTBP2	
C3orf58	ANKRD9		RAB4A					ABHD5	

CAP1	ARID4A		H3F3B					CHRDL1	
JADE1	ANKRD1		PLAU					PLK3	
MSN	MISP		NOP16					GBP1	
JAGN1	ANGPTL2		DUSP14					CEP126	
RAB17	RETSAT		RHOC					STK17B	
NFATC3	SLC16A5		RASSF10					ANGPTL4	
SBDSP1	STOM		EIF5A					RAPH1	
CLIC2	PTPN14		SSPN					ANTXR2	
ACTR10	NHSL1		TRIM47					ACP5	
PLEKHB2	B4GAT1		TGFBI					TNFAIP2	
SWAP70	LGALS3		BLCAP					S100A13	
TMEM139	APC		NOB1					ITIH5	
BLVRB	SEMA6D		ARF4					MAFB	
ARHGAP5	ATXN1		KLF5					MDK	
FAS	TMEM59		NIFK					GADD45B	
TRIM16	GSTM3		SNRPB					PTX3	
PFDN1	LURAP1L		NFKBIA					CCL2	
SAP30BP	ZFP36		NXT1						
PIK3IP1	APBB2		ARF6						
YPEL3	GLCCI1		SOX9						
MYO6	NRBP2		NFE2L2						
BTG3	FZD5		EGFR						
MAL2	CNTN6		DNAJB6						
EBPL	TTC7A		MT1E						
TAGLN2	WASF3		CNN2						
ATP1B3	AATK		DCN						
GALNT18	TRIB1		EYA2						
SLC44A2	SNX22		INPP1						
UNC50	PMP22		ILF2						
TMEM30A	INPP5K		EIF3M						
CTNNBIP1	DGKD		CLK1						
PAFAH1B1	ADRB1		RMDN2						
HSPB1	BST2		RBM8A						
SYNPO	EPB41L3		C1S						
FOSB	KCNQ1OT1		ANXA1						
REXO2	RASAL2		PA2G4						
ANO6	TSC22D3		BAZ1A						
RHOF	ATP10A		EFNA5						
ATP11A	APBB1		POLR1D						
OCLN	CCL2		ALDH7A1						
DAPK3	TPPP		EIF3I						
CYB5R3	ARHGEF2		RAP2B						
PARVA	LINC-PINT		RNPS1						
TUBA4A	NGF		C12orf57						
ST6GALNAC5	GBP1		TMEM123						
VCL	RCOR1		ACTR3						
MAPK1IP1L	XPO4		NME1						
CPNE3	MYO9A		PDLIM4						
SMPD1	RICTOR		EIF3F						
C1D	NUPR1		ARID5A						

RBMS1	SMAD7		MAT2A						
ERP44	LATS2		LMO4						
ANKRD12	AQP1		SRSF3						
RBMS3	RAPGEF2		LAPTM4A						
FZD6	ZNF704		BRIX1						
CHMP5	RIMKLA		PRRC2C						
HTATIP2	ZFP36L1		GADD45A						
LMBRD1	UPP1		ACSL3						
PEF1	DOT1L		TSLP						
GRN	METRNL		LRG1						
PPP1R15A	PLAC8		KCTD1						
CDH1	UBE2Q2P1		SLC5A3						
GIPC1	AMOTL1		TKT						
FAM174B	UNC13B		GNL3						
LIMD1	NINJ2		GSTO1						
PCMT1	HIP1		PFN1						
CNIH4	IGSF3		KHDRBS1						
LAMP2	RARRES3		ANP32B						
COMMD2	SELENBP1		CCT4						
LAPTM4B	RIPK2		KLK10						
ITGB6	ISG15		TUSC1						
IL17RE	SCNN1G		TAGLN2						
RARRES2	PDZD2		EIF1B						
NCOR2	SLC16A4		SYTL1						
CRB3	ZC2HC1A		ATF4						
SRSF7	KHDRBS2		RRS1						
DCTN2	PLAUR		BEX4						
RAD21	MVB12B		ALDH1A3						
ELOVL1	ITPRIP		CLNS1A						
FAM177A1	ZFAND2A		CFL1						
ZC3H14	C14orf132		CCT2						
MAGI3	ZNF431		EIF4B						
POR	DOCK11		ZFP36L1						
TMEM98	B3GNT8		TRIP10						
TKT	PLCE1		7-Sep						
MT2A	PEG10		PARP1						
IL6R	NCOA7		HMGN1						
BMPR2	CDKN1A		TMED9						
BCAM	IFIT1		KDSR						
TMBIM1	REPS2		BZW1						
FBLN5	TTLL7		MORF4L1						
LDLR	SHROOM3		CDK4						
ERBB3	P3H2		CTSF						
YPEL5	ABI3BP		UBE2I						
CAPG	IFIT3		HNRNPC						
SAR1A	DAPK1		UGCG						
BDNF	KLK11		PGK1						
SCNN1A	SMAD9		EIF4A3						
PAM	DNAJB4		SSBP2						
JUP	VEPH1		VDAC2						

CDV3	NEAT1		EIF2A						
PNRC2	TMEM47		ST13						
C1orf116	GAB1		EIF3G						
ZNF706	PIM1		TOMM40						
NEXN	CTSE		BNIP3						
MEAF6	IGFBP7		UBE2N						
SFN	HSP90AB1		NAB2						
TMPRSS2	KCNN3		RELB						
RAB18	SFTA3		SNRPD1						
ACAA2	HLA-A		EIF3J						
ADD1	NFKBIA		TM2D3						
	IFI6		MRFAP1						
	ALDH3B1		HEXIM1						
	FOS		TNFRSF10B						
	PDLIM3		TLE3						
	JUN		PXDC1						
	RGS16		FUS						
			ZFAND5						
			FUCA1						
			YTHDF2						
			EIF1AX						
			CCT6A						
			PIM3						
			ZC3H12A						
			CSNK1A1						
			NHP2						
			HSPA9						
			CSRP1						
			NOP58						
			UBE2D3						
			LINC01436						
			NOP56						
			IMP3						
			HNRNPAB						
			ANKRD12						
			RBMX						
			TNFRSF1A						
			TRA2B						
			FKBP4						
			DEK						
			LRRFIP1						
			DUT						
			CCNI						
			ARHGEF35						
			GRINA						
			TFPI						
			SRSF5						
			EML4						
			WWTR1						
			AMD1						

			CIRBP						
			IRF6						
			VDAC1						
			UGDH						
			MARCKS						
			PER2						
			IGFBP4						
			BHLHE40						
			CCNL1						
			RHOB						
			IER2						
			SNHG5						
			FOSB						
			EPHA2						
			SOX4						
			WIPI1						
			MYADM						
			LBH						
			SRSF7						
			TNFAIP3						
			HNRNPH1						
			CBR1						
			ATF3						

Supplementary Table S13
ssGSEA enrichment score for different mouse and human pulmonary cells.

Mouse

	AT1 cell	AT2 cell	Basal	Ciliated	Club	Goblet	Endothelial	Fibroblast	Myofibroblast
Week 1-5	1.09	1.04	0.70	0.24	0.86	0.65	1.13	0.82	0.85
Week 1-5	1.09	1.04	0.68	0.24	0.86	0.61	1.12	0.82	0.85
Week 1-5	1.11	1.07	0.62	0.22	0.86	0.66	1.14	0.79	0.85
Week 1-5	1.10	1.09	0.63	0.26	0.85	0.66	1.14	0.79	0.82
Week 1-5	1.09	1.05	0.66	0.26	0.85	0.67	1.10	0.81	0.82
Week 1-5	1.19	1.12	0.51	0.46	1.04	0.85	1.19	0.74	0.64
Week 1-5	1.19	1.12	0.49	0.45	1.02	0.84	1.18	0.74	0.65
Week 1-5	1.19	1.13	0.47	0.49	1.04	0.84	1.19	0.73	0.61
Week 1-5	1.18	1.11	0.48	0.48	1.04	0.84	1.21	0.74	0.65
Week 1-5	1.19	1.12	0.47	0.51	1.06	0.87	1.19	0.74	0.66
Week 6-26	1.17	1.15	0.46	0.61	1.09	0.87	1.20	0.75	0.71
Week 6-26	1.17	1.15	0.45	0.49	1.08	0.82	1.20	0.74	0.69
Week 6-26	1.16	1.10	0.44	0.56	1.06	0.83	1.20	0.72	0.67
Week 6-26	1.16	1.10	0.46	0.61	1.09	0.88	1.19	0.73	0.69
Week 6-26	1.17	1.12	0.45	0.60	1.08	0.89	1.19	0.72	0.67
Week 6-26	1.18	1.11	0.46	0.61	1.09	0.90	1.21	0.74	0.76
Week 6-26	1.18	1.10	0.47	0.55	1.07	0.87	1.21	0.75	0.73
Week 6-26	1.20	1.12	0.45	0.62	1.09	0.80	1.21	0.73	0.73
Week 6-26	1.17	1.14	0.46	0.45	1.05	0.82	1.20	0.72	0.62
Week 6-26	1.16	1.14	0.46	0.50	1.05	0.87	1.20	0.70	0.62
Week 6-26	1.17	1.14	0.46	0.49	1.04	0.82	1.20	0.70	0.59
Week 6-26	1.15	1.11	0.47	0.41	1.01	0.71	1.19	0.71	0.61
Week 6-26	1.17	1.13	0.47	0.48	1.04	0.80	1.20	0.70	0.58
Week 6-26	1.18	1.14	0.45	0.46	1.07	0.89	1.17	0.71	0.70
Week 6-26	1.15	1.13	0.46	0.54	1.09	0.87	1.16	0.71	0.64
Week 6-26	1.17	1.12	0.45	0.47	1.05	0.83	1.20	0.70	0.67
Week 6-26	1.16	1.14	0.46	0.61	1.09	0.85	1.20	0.71	0.64
Week 6-26	1.16	1.14	0.50	0.58	1.09	0.87	1.19	0.72	0.64
Week 6-26	1.17	1.16	0.49	0.60	1.13	0.90	1.17	0.71	0.64
Week 6-26	1.18	1.14	0.49	0.63	1.11	0.91	1.19	0.73	0.65
Week 6-26	1.18	1.15	0.47	0.61	1.10	0.88	1.20	0.74	0.67
Week 6-26	1.17	1.14	0.46	0.64	1.10	0.89	1.19	0.74	0.69
Week 6-26	1.10	1.18	0.47	0.56	1.08	0.90	1.08	0.63	0.56
Week 6-26	1.16	1.10	0.45	0.62	1.11	0.91	1.17	0.67	0.60
Week 6-26	1.16	1.10	0.45	0.60	1.08	0.86	1.19	0.69	0.61
Week 6-26	1.15	1.10	0.47	0.56	1.08	0.92	1.15	0.68	0.64
Week 6-26	1.15	1.12	0.43	0.59	1.09	0.90	1.15	0.65	0.56
Week 6-26	1.16	1.11	0.45	0.57	1.06	0.91	1.19	0.63	0.54
Week 6-26	1.12	0.99	0.42	0.55	0.93	0.65	1.11	0.65	0.61
Week 6-26	1.15	0.98	0.42	0.36	0.88	0.56	1.16	0.68	0.65
Week 6-26	1.10	0.98	0.43	0.56	0.92	0.66	1.14	0.66	0.65
Week 6-26	1.14	0.98	0.43	0.42	0.95	0.70	1.17	0.67	0.66
Week 6-26	1.09	0.99	0.39	0.46	0.92	0.62	1.16	0.68	0.67
Week 6-26	1.17	1.11	0.46	0.65	1.08	0.88	1.21	0.74	0.68
Week 6-26	1.15	1.10	0.47	0.67	1.07	0.89	1.18	0.72	0.63
Week 6-26	1.14	1.10	0.48	0.59	1.04	0.80	1.15	0.72	0.60
Week 6-26	0.80	0.77	0.54	1.03	1.20	1.12	0.57	0.75	0.77
Week 6-26	0.79	0.75	0.57	1.00	1.20	1.13	0.58	0.76	0.78
Week 6-26	0.78	0.79	0.53	1.06	1.20	1.12	0.58	0.74	0.76
Week 6-26	0.79	0.77	0.51	1.00	1.19	1.12	0.56	0.73	0.75
Week 6-26	1.18	1.15	0.46	0.67	1.10	0.87	1.21	0.73	0.70
Week 6-26	1.17	1.16	0.48	0.70	1.11	0.93	1.19	0.73	0.69
Week 6-26	1.17	1.15	0.46	0.72	1.10	0.89	1.20	0.73	0.67
Week 6-26	1.09	1.06	0.46	0.79	1.09	0.87	1.11	0.70	0.61
Week 6-26	1.11	1.10	0.48	0.76	1.11	0.87	1.14	0.73	0.65
Week 6-26	1.08	1.10	0.45	0.82	1.11	0.88	1.10	0.68	0.56
Week 6-26	1.13	1.07	0.49	0.72	1.08	0.90	1.08	0.72	0.60
Week 6-26	1.12	1.06	0.47	0.79	1.11	0.97	1.07	0.70	0.63
Week 6-26	1.13	1.08	0.51	0.75	1.09	0.96	1.06	0.71	0.63
Week 6-26	1.12	1.10	0.46	0.71	1.09	0.87	1.14	0.71	0.61
Week 6-26	1.11	1.12	0.48	0.75	1.12	0.91	1.16	0.71	0.60
Week 6-26	1.11	1.09	0.47	0.74	1.09	0.85	1.14	0.71	0.60
Week 52-130	1.12	1.08	0.48	0.80	1.10	0.91	1.17	0.71	0.63

Week 52-130	1.07	1.06	0.46	0.81	1.09	0.87	1.10	0.67	0.52
Week 52-130	1.09	1.06	0.49	0.76	1.08	0.88	1.09	0.69	0.58
Week 52-130	0.78	0.76	0.53	1.01	1.20	1.13	0.57	0.72	0.72
Week 52-130	0.78	0.79	0.53	1.06	1.19	1.17	0.57	0.71	0.71
Week 52-130	0.81	0.77	0.55	1.02	1.20	1.13	0.57	0.74	0.74
Week 52-130	0.79	0.78	0.54	1.02	1.20	1.13	0.58	0.73	0.70
Week 52-130	1.12	1.09	0.44	0.77	1.10	0.89	1.16	0.70	0.59
Week 52-130	1.10	1.08	0.50	0.75	1.09	0.87	1.13	0.70	0.58
Week 52-130	1.09	1.10	0.48	0.75	1.10	0.90	1.12	0.69	0.59
Week 52-130	1.07	1.08	0.46	0.79	1.09	0.86	1.12	0.67	0.55
Week 52-130	1.06	1.07	0.50	0.74	1.05	0.83	1.11	0.66	0.54
Week 52-130	1.10	1.10	0.45	0.82	1.10	0.86	1.13	0.67	0.56
Week 52-130	1.07	1.08	0.47	0.93	1.15	0.94	1.12	0.66	0.56
Week 52-130	1.04	1.04	0.51	0.78	1.09	0.85	1.10	0.63	0.48
Week 52-130	1.09	1.06	0.47	0.74	1.08	0.87	1.10	0.70	0.60
	**Dendritic**	**Neutrophil**	**Alveolar M.**	**Interstitial M.**	**Eosinophil**	**NK**	**B cell**	**CD4+ T**	**CD8+ T**
Week 1-5	0.80	0.54	0.78	0.77	0.66	0.66	0.56	0.64	0.80
Week 1-5	0.80	0.54	0.75	0.78	0.66	0.69	0.59	0.67	0.81
Week 1-5	0.81	0.54	0.79	0.80	0.69	0.69	0.59	0.67	0.81
Week 1-5	0.81	0.58	0.82	0.80	0.71	0.68	0.58	0.66	0.81
Week 1-5	0.81	0.54	0.78	0.79	0.69	0.72	0.60	0.69	0.83
Week 1-5	0.88	0.57	0.90	0.88	0.77	0.84	0.73	0.80	0.89
Week 1-5	0.87	0.51	0.86	0.85	0.70	0.79	0.69	0.76	0.86
Week 1-5	0.87	0.53	0.87	0.85	0.74	0.81	0.70	0.76	0.86
Week 1-5	0.88	0.54	0.87	0.86	0.77	0.80	0.71	0.78	0.88
Week 1-5	0.88	0.58	0.91	0.88	0.81	0.82	0.73	0.79	0.88
Week 6-26	0.89	0.63	0.90	0.90	0.81	0.80	0.70	0.79	0.88
Week 6-26	0.90	0.64	0.92	0.91	0.86	0.85	0.71	0.80	0.89
Week 6-26	0.89	0.64	0.88	0.91	0.89	0.85	0.81	0.82	0.92
Week 6-26	0.86	0.50	0.84	0.83	0.73	0.74	0.67	0.76	0.86
Week 6-26	0.86	0.50	0.84	0.82	0.74	0.71	0.67	0.71	0.84
Week 6-26	0.88	0.51	0.81	0.85	0.74	0.76	0.69	0.75	0.85
Week 6-26	0.88	0.54	0.83	0.85	0.77	0.77	0.72	0.79	0.89
Week 6-26	0.88	0.52	0.82	0.84	0.75	0.71	0.62	0.73	0.83
Week 6-26	0.91	0.56	0.91	0.89	0.77	0.84	0.77	0.81	0.90
Week 6-26	0.92	0.56	0.90	0.90	0.79	0.87	0.78	0.84	0.92
Week 6-26	0.91	0.58	0.92	0.90	0.78	0.85	0.74	0.83	0.91
Week 6-26	0.95	0.62	0.94	0.94	0.84	0.93	0.92	0.91	0.97
Week 6-26	0.90	0.51	0.89	0.86	0.73	0.83	0.73	0.78	0.87
Week 6-26	0.88	0.59	0.88	0.88	0.79	0.81	0.68	0.76	0.86
Week 6-26	0.93	0.69	0.93	0.96	0.91	0.93	0.77	0.83	0.92
Week 6-26	0.92	0.64	0.86	0.94	0.83	0.90	0.76	0.83	0.92
Week 6-26	0.88	0.62	0.90	0.88	0.77	0.78	0.68	0.77	0.86
Week 6-26	0.92	0.68	0.93	0.95	0.84	0.89	0.77	0.83	0.91
Week 6-26	0.88	0.59	0.88	0.89	0.76	0.81	0.73	0.80	0.87
Week 6-26	0.90	0.64	0.90	0.91	0.83	0.81	0.72	0.79	0.88
Week 6-26	0.91	0.64	0.92	0.93	0.85	0.83	0.72	0.80	0.88
Week 6-26	0.90	0.65	0.91	0.93	0.89	0.81	0.69	0.79	0.87
Week 6-26	0.97	0.75	0.99	1.03	0.97	0.92	0.89	0.95	0.98
Week 6-26	0.94	0.73	0.92	0.98	0.94	0.91	0.87	0.93	0.99
Week 6-26	0.92	0.66	0.92	0.96	0.88	0.88	0.82	0.89	0.95
Week 6-26	0.94	0.72	0.94	0.99	0.95	0.89	0.84	0.90	0.96
Week 6-26	0.93	0.68	0.91	0.97	0.93	0.91	0.83	0.92	0.97
Week 6-26	0.93	0.81	1.00	1.00	1.08	0.94	0.70	0.96	1.01
Week 6-26	0.71	0.38	0.67	0.58	0.52	0.57	0.62	0.62	0.76
Week 6-26	0.77	0.36	0.67	0.67	0.59	0.61	0.54	0.66	0.79
Week 6-26	0.77	0.45	0.68	0.67	0.65	0.68	0.69	0.71	0.84
Week 6-26	0.76	0.43	0.65	0.67	0.63	0.62	0.69	0.76	0.82
Week 6-26	0.74	0.21	0.66	0.64	0.51	0.58	0.59	0.70	0.78
Week 6-26	0.90	0.55	0.86	0.88	0.77	0.87	0.82	0.88	0.96
Week 6-26	0.91	0.58	0.86	0.89	0.80	0.89	0.84	0.90	0.99
Week 6-26	0.91	0.58	0.89	0.90	0.80	0.90	0.84	0.90	0.99
Week 6-26	0.81	0.42	0.71	0.83	0.60	0.55	0.48	0.64	0.76
Week 6-26	0.83	0.45	0.72	0.85	0.61	0.57	0.49	0.66	0.77
Week 6-26	0.82	0.45	0.72	0.84	0.59	0.56	0.47	0.63	0.76
Week 6-26	0.80	0.43	0.71	0.82	0.61	0.55	0.46	0.63	0.76
Week 6-26	0.92	0.60	0.90	0.93	0.85	0.89	0.83	0.88	0.95
Week 6-26	0.92	0.60	0.94	0.92	0.81	0.85	0.77	0.85	0.91
Week 6-26	0.92	0.63	0.92	0.94	0.84	0.89	0.83	0.86	0.94

Week 6-26	0.90	0.66	0.89	0.93	0.93	0.89	0.78	0.86	0.95
Week 6-26	0.91	0.62	0.86	0.92	0.83	0.85	0.82	0.85	0.94
Week 6-26	0.92	0.62	0.87	0.93	0.84	0.87	0.78	0.86	0.94
Week 6-26	0.90	0.52	0.81	0.89	0.74	0.82	0.76	0.82	0.90
Week 6-26	0.89	0.53	0.82	0.87	0.74	0.82	0.76	0.81	0.91
Week 6-26	0.94	0.63	0.88	0.96	0.82	0.88	0.80	0.89	0.95
Week 6-26	0.90	0.60	0.91	0.91	0.82	0.88	0.83	0.86	0.95
Week 6-26	0.93	0.66	0.90	0.95	0.89	0.93	0.79	0.90	0.96
Week 6-26	0.89	0.55	0.85	0.87	0.79	0.86	0.81	0.86	0.94
Week 52-130	0.94	0.64	0.86	0.95	0.87	0.92	0.97	0.93	0.98
Week 52-130	0.91	0.64	0.88	0.97	0.91	0.89	0.82	0.90	0.96
Week 52-130	0.91	0.61	0.88	0.95	0.85	0.92	0.89	0.91	0.98
Week 52-130	0.87	0.55	0.75	0.91	0.72	0.65	0.57	0.75	0.82
Week 52-130	0.87	0.55	0.75	0.90	0.71	0.68	0.62	0.77	0.84
Week 52-130	0.84	0.50	0.75	0.87	0.66	0.62	0.53	0.71	0.79
Week 52-130	0.85	0.52	0.75	0.89	0.69	0.63	0.53	0.72	0.80
Week 52-130	0.88	0.65	0.87	0.90	0.90	0.81	0.75	0.82	0.89
Week 52-130	0.96	0.73	0.93	1.00	0.94	0.93	0.92	0.93	0.97
Week 52-130	0.95	0.72	0.99	1.03	0.93	0.90	0.92	0.90	0.94
Week 52-130	0.94	0.70	0.95	1.01	0.95	0.90	0.88	0.92	0.97
Week 52-130	0.98	0.75	0.95	1.04	1.00	0.95	1.00	1.00	1.02
Week 52-130	0.92	0.69	0.94	0.98	0.92	0.88	0.86	0.90	0.95
Week 52-130	0.94	1.00	0.95	1.02	1.15	0.92	0.86	0.94	0.96
Week 52-130	0.99	0.91	0.94	1.06	1.09	0.93	1.11	0.99	1.02
Week 52-130	0.91	0.64	0.92	0.96	0.89	0.89	0.88	0.92	0.99

Human test set

	AT1 cell	AT2 cell	Basal	Ciliated	Club	Goblet	Capillary	Fibroblast	Myofibroblast
Y40	0.93	0.92	0.81	0.29	1.04	0.58	0.85	0.79	0.72
Y40	0.95	0.98	0.81	0.26	1.08	0.61	0.88	0.77	0.69
Y40	0.96	0.97	0.77	0.15	1.05	0.53	0.80	0.75	0.63
Y40	0.87	0.92	0.77	0.26	1.06	0.60	0.74	0.70	0.63
Y40	0.94	0.93	0.76	0.21	1.01	0.53	0.88	0.81	0.68
Y60	0.93	0.93	0.78	0.19	1.10	0.55	0.70	0.71	0.67
Y60	0.97	0.94	0.79	0.13	1.03	0.50	0.90	0.80	0.67
Y60	0.92	0.94	0.80	0.41	1.10	0.63	0.63	0.66	0.67
Y60	0.96	0.93	0.84	0.16	1.02	0.52	0.85	0.80	0.69
Y60	0.93	0.93	0.77	0.18	1.06	0.54	0.76	0.71	0.67
Y60	0.95	0.95	0.76	0.19	1.06	0.56	0.91	0.73	0.65
Y60	0.95	0.85	0.74	0.25	1.01	0.52	0.89	0.77	0.68
Y60	0.93	0.93	0.75	0.23	1.07	0.55	0.81	0.71	0.63
Y60	0.94	0.95	0.75	0.12	1.05	0.52	0.79	0.71	0.63
Y60	0.94	0.86	0.77	0.26	1.01	0.54	0.86	0.84	0.73
Y60	0.94	0.97	0.78	0.19	1.06	0.56	0.74	0.76	0.73
Y60	0.91	0.93	0.80	0.33	1.08	0.62	0.82	0.76	0.65
Y60	0.92	0.87	0.78	0.40	1.08	0.64	0.72	0.68	0.72
Y60	0.94	0.83	0.77	0.16	1.02	0.54	0.77	0.81	0.77
Y60	0.90	0.90	0.81	0.24	1.03	0.62	0.83	0.82	0.81
Y60	0.98	0.99	0.76	0.27	1.06	0.57	0.86	0.70	0.65
Y60	0.95	0.96	0.76	0.15	1.05	0.53	0.84	0.79	0.68
Y60	0.97	0.96	0.80	0.44	1.06	0.60	0.83	0.71	0.62
Y60	0.95	0.88	0.76	0.11	1.03	0.48	0.80	0.82	0.72
Y60	0.96	0.94	0.75	0.14	1.02	0.53	0.88	0.75	0.64
Y60	0.89	0.91	0.83	0.42	1.07	0.68	0.77	0.66	0.61
Y60	0.99	0.91	0.75	0.44	1.04	0.57	0.73	0.71	0.61
Y60	0.93	0.98	0.77	0.13	1.08	0.52	0.76	0.77	0.65
Y60	0.92	0.93	0.79	0.16	1.03	0.51	0.91	0.80	0.67
Y60	0.93	0.89	0.74	0.14	1.02	0.51	0.86	0.80	0.72
Y60	0.88	0.94	0.76	0.20	1.04	0.58	0.68	0.67	0.61
Y60	0.91	0.92	0.79	0.32	1.07	0.65	0.81	0.77	0.67
Y60	0.91	0.84	0.74	0.47	1.02	0.60	0.78	0.78	0.69
Y60	0.97	0.99	0.80	0.15	1.02	0.54	0.82	0.85	0.74
Y60	0.92	0.90	0.75	0.21	1.03	0.52	0.85	0.78	0.69

Y60	0.89	0.93	0.81	0.14	1.05	0.60	0.73	0.71	0.66
Y60	0.95	0.90	0.74	0.25	1.03	0.52	0.84	0.79	0.65
Y60	0.94	0.93	0.76	0.14	1.04	0.51	0.84	0.79	0.69
Y60	0.96	0.98	0.83	0.23	1.05	0.58	0.80	0.74	0.67
Y60	0.95	0.87	0.76	0.13	1.04	0.51	0.84	0.78	0.71
Y60	0.88	0.83	0.76	0.22	0.99	0.58	0.77	0.79	0.75
Y60	0.83	0.88	0.83	0.12	1.02	0.60	0.77	0.80	0.77
Y60	0.91	0.81	0.77	0.14	1.02	0.51	0.86	0.82	0.77
Y60	0.86	0.88	0.79	0.12	1.03	0.57	0.70	0.75	0.74
Y60	0.95	0.93	0.79	0.20	1.05	0.54	0.88	0.81	0.72
Y60	0.94	0.91	0.76	0.20	1.04	0.53	0.83	0.77	0.67
Y60	0.92	0.94	0.78	0.25	1.02	0.58	0.78	0.75	0.72
Y60	0.96	0.87	0.75	0.17	1.04	0.51	0.85	0.79	0.69
Y60	0.94	0.96	0.85	0.30	1.08	0.61	0.83	0.75	0.66
Y60	0.92	0.85	0.80	0.39	1.06	0.61	0.80	0.73	0.68
Y60	0.86	0.92	0.81	0.17	1.03	0.59	0.75	0.78	0.68
Y60	0.93	0.95	0.81	0.29	1.06	0.60	0.82	0.74	0.70
Y60	0.87	0.84	0.82	0.15	0.98	0.60	0.78	0.80	0.76
Y60	0.93	0.79	0.77	0.54	1.01	0.68	0.83	0.75	0.65
Y60	0.95	0.94	0.78	0.19	1.02	0.52	0.86	0.79	0.70
Y60	0.95	0.88	0.78	0.38	1.04	0.59	0.85	0.75	0.66
Y60	0.92	0.89	0.76	0.26	1.05	0.56	0.80	0.79	0.73
Y60	0.80	0.79	0.72	0.18	1.01	0.54	0.71	0.64	0.61
Y60	0.89	0.83	0.78	0.47	1.00	0.66	0.76	0.78	0.71
Y60	0.95	0.98	0.84	0.22	1.06	0.56	0.88	0.74	0.66
Y60	0.92	0.90	0.76	0.10	0.99	0.52	0.78	0.82	0.75
Y60	0.91	0.87	0.78	0.30	1.03	0.59	0.82	0.80	0.76
Y60	0.99	0.86	0.75	0.16	1.00	0.50	0.86	0.81	0.67
Y60	0.94	0.92	0.80	0.33	1.03	0.59	0.76	0.80	0.71
Y60	0.92	1.01	0.75	0.15	1.08	0.53	0.71	0.64	0.57
Y60	0.94	0.95	0.86	0.26	1.03	0.63	0.88	0.76	0.64
Y60	0.88	0.94	0.82	0.25	1.06	0.65	0.74	0.75	0.74
Y80	0.91	0.90	0.77	0.22	1.02	0.59	0.81	0.78	0.72
Y80	0.93	0.88	0.78	0.27	1.02	0.60	0.85	0.79	0.72
Y80	1.00	0.98	0.80	0.16	1.03	0.53	0.90	0.74	0.65
Y80	1.00	0.89	0.76	0.36	1.08	0.56	0.71	0.72	0.63
Y80	0.95	0.93	0.79	0.52	1.07	0.61	0.78	0.69	0.68
Y80	0.94	0.77	0.77	0.56	1.02	0.62	0.88	0.77	0.67
Y80	0.92	1.01	0.77	0.32	1.08	0.61	0.66	0.67	0.59
Y80	0.94	0.95	0.80	0.17	1.05	0.55	0.75	0.79	0.74
Y80	0.96	0.88	0.75	0.16	1.01	0.50	0.88	0.81	0.70
Y80	0.95	0.86	0.77	0.29	1.02	0.57	0.83	0.74	0.73
Y80	0.95	0.98	0.83	0.30	1.06	0.57	0.82	0.80	0.65
Y80	0.87	0.69	0.81	0.58	1.05	0.62	0.61	0.71	0.75
Y80	0.89	0.95	0.78	0.23	1.05	0.58	0.72	0.71	0.65
Y80	0.95	0.96	0.77	0.12	1.02	0.51	0.85	0.82	0.72
Y80	0.96	0.86	0.75	0.20	1.01	0.50	0.91	0.77	0.68
Y80	0.94	0.75	0.75	0.28	1.04	0.52	0.83	0.78	0.67
Y80	0.87	0.83	0.74	0.29	1.01	0.63	0.69	0.63	0.56
Y80	0.95	0.88	0.76	0.48	1.03	0.60	0.82	0.75	0.64

Y80	0.96	0.91	0.75	0.17	1.02	0.51	0.93	0.77	0.67
Y80	0.97	0.90	0.79	0.40	1.04	0.54	0.90	0.78	0.69
Y80	0.93	0.94	0.77	0.37	1.06	0.61	0.76	0.74	0.66
Y80	0.89	0.95	0.81	0.26	1.10	0.61	0.77	0.73	0.70
Y80	0.94	0.98	0.80	0.16	1.07	0.54	0.85	0.77	0.65
Y80	0.89	0.90	0.80	0.16	1.03	0.61	0.78	0.75	0.73
Y80	0.94	0.96	0.80	0.28	1.06	0.60	0.80	0.75	0.65
Y80	0.98	0.92	0.76	0.41	1.05	0.60	0.81	0.74	0.64
Y80	0.97	0.99	0.79	0.20	1.07	0.53	0.78	0.78	0.68
Y80	0.93	0.90	0.78	0.36	1.05	0.60	0.84	0.78	0.70
Y80	0.92	0.83	0.77	0.23	0.98	0.56	0.88	0.85	0.77
Y80	0.90	0.82	0.77	0.28	1.01	0.56	0.91	0.85	0.75
Y80	0.90	0.77	0.75	0.23	1.01	0.55	0.71	0.77	0.74
Y80	0.96	0.99	0.78	0.17	1.05	0.54	0.86	0.77	0.69
Y80	0.83	0.81	0.80	0.33	0.97	0.62	0.87	0.77	0.74
Y80	0.97	0.84	0.86	0.25	1.00	0.55	0.88	0.85	0.70
Y80	0.93	0.93	0.84	0.13	1.03	0.55	0.85	0.77	0.70
Y80	0.95	0.89	0.75	0.17	1.03	0.51	0.85	0.75	0.64
Y80	0.93	1.00	0.80	0.16	1.05	0.57	0.73	0.78	0.67
Y80	0.90	0.81	0.76	0.11	1.01	0.53	0.77	0.78	0.73
Y80	0.96	0.93	0.80	0.16	1.03	0.52	0.91	0.77	0.72
Y80	0.95	0.99	0.84	0.45	1.07	0.63	0.75	0.70	0.63
	**Dendritic**	**Neutrophil**	**Macrophage**	**NK**	**B cell**	**CD4+ Naive**	**CD4+ Effect**	**CD8+ Naive**	**CD8+ Effect**
Y40	0.84	0.67	0.92	0.63	0.48	0.80	0.54	0.57	0.60
Y40	0.92	0.74	0.97	0.63	0.49	0.81	0.57	0.60	0.62
Y40	0.83	0.76	0.90	0.53	0.46	0.77	0.51	0.52	0.54
Y40	0.94	0.75	0.94	0.61	0.52	0.82	0.57	0.57	0.64
Y40	0.80	0.77	0.87	0.50	0.48	0.77	0.54	0.49	0.55
Y60	0.93	0.80	0.92	0.57	0.55	0.81	0.62	0.57	0.64
Y60	0.83	0.76	0.87	0.53	0.51	0.79	0.58	0.53	0.61
Y60	0.81	0.72	0.90	0.47	0.46	0.77	0.48	0.44	0.47
Y60	0.82	0.79	0.88	0.54	0.48	0.74	0.54	0.52	0.54
Y60	0.83	0.78	0.87	0.51	0.56	0.80	0.56	0.53	0.58
Y60	0.81	0.74	0.86	0.57	0.47	0.79	0.53	0.56	0.57
Y60	0.79	0.74	0.85	0.54	0.46	0.79	0.53	0.53	0.57
Y60	0.88	0.77	0.92	0.55	0.50	0.80	0.56	0.55	0.61
Y60	0.83	0.76	0.88	0.50	0.47	0.77	0.54	0.49	0.54
Y60	0.78	0.73	0.82	0.50	0.44	0.77	0.49	0.46	0.50
Y60	0.84	0.76	0.90	0.50	0.46	0.76	0.50	0.49	0.51
Y60	0.87	0.75	0.99	0.55	0.46	0.78	0.50	0.50	0.53
Y60	0.87	0.67	0.93	0.61	0.51	0.81	0.57	0.55	0.60
Y60	0.86	0.75	0.86	0.53	0.47	0.79	0.56	0.53	0.58
Y60	0.91	0.71	0.97	0.64	0.47	0.79	0.55	0.56	0.58
Y60	0.78	0.72	0.83	0.50	0.46	0.76	0.50	0.47	0.52
Y60	0.82	0.76	0.88	0.53	0.47	0.78	0.54	0.55	0.57
Y60	0.80	0.75	0.86	0.48	0.44	0.75	0.50	0.47	0.51
Y60	0.83	0.76	0.82	0.55	0.49	0.79	0.58	0.57	0.61
Y60	0.87	0.75	0.89	0.55	0.47	0.79	0.55	0.56	0.59
Y60	0.92	0.79	1.04	0.57	0.47	0.77	0.53	0.53	0.54
Y60	0.75	0.73	0.84	0.45	0.42	0.74	0.48	0.43	0.47

Y60	0.80	0.77	0.84	0.51	0.49	0.79	0.53	0.51	0.55
Y60	0.84	0.80	0.88	0.57	0.51	0.82	0.60	0.58	0.65
Y60	0.78	0.73	0.83	0.49	0.47	0.76	0.51	0.48	0.54
Y60	0.83	0.77	0.93	0.55	0.57	0.83	0.59	0.56	0.61
Y60	0.86	0.77	0.92	0.53	0.50	0.80	0.57	0.53	0.58
Y60	0.78	0.73	0.80	0.50	0.46	0.78	0.53	0.49	0.53
Y60	0.86	0.78	0.89	0.55	0.47	0.77	0.54	0.55	0.55
Y60	0.81	0.77	0.86	0.48	0.47	0.76	0.51	0.48	0.52
Y60	0.91	0.80	0.97	0.53	0.48	0.76	0.53	0.54	0.54
Y60	0.80	0.75	0.82	0.53	0.47	0.80	0.56	0.54	0.59
Y60	0.87	0.78	0.86	0.59	0.55	0.83	0.65	0.61	0.69
Y60	0.83	0.78	0.93	0.54	0.48	0.76	0.54	0.53	0.54
Y60	0.79	0.74	0.85	0.51	0.49	0.79	0.53	0.50	0.56
Y60	0.83	0.74	0.87	0.52	0.46	0.76	0.54	0.54	0.55
Y60	0.91	0.78	0.91	0.57	0.54	0.82	0.60	0.59	0.63
Y60	0.86	0.75	0.82	0.59	0.61	0.84	0.61	0.59	0.65
Y60	0.87	0.77	0.89	0.60	0.64	0.85	0.62	0.63	0.66
Y60	0.81	0.71	0.84	0.60	0.48	0.82	0.57	0.58	0.62
Y60	0.81	0.76	0.86	0.51	0.47	0.78	0.53	0.52	0.55
Y60	0.87	0.77	0.91	0.54	0.49	0.77	0.57	0.55	0.57
Y60	0.84	0.75	0.87	0.61	0.51	0.82	0.62	0.62	0.66
Y60	0.88	0.81	0.98	0.59	0.49	0.79	0.56	0.55	0.59
Y60	0.80	0.74	0.86	0.55	0.47	0.79	0.52	0.56	0.55
Y60	0.89	0.81	0.93	0.59	0.53	0.81	0.61	0.62	0.66
Y60	0.88	0.71	0.97	0.65	0.49	0.82	0.57	0.56	0.61
Y60	0.85	0.77	0.88	0.53	0.47	0.76	0.54	0.54	0.55
Y60	0.87	0.77	0.94	0.54	0.46	0.78	0.54	0.54	0.55
Y60	0.82	0.78	0.88	0.54	0.47	0.76	0.53	0.55	0.55
Y60	0.86	0.77	0.90	0.58	0.50	0.81	0.60	0.61	0.65
Y60	0.87	0.69	0.93	0.65	0.52	0.84	0.63	0.62	0.70
Y60	0.86	0.79	0.87	0.61	0.86	0.93	0.74	0.66	0.77
Y60	0.83	0.75	0.85	0.52	0.48	0.79	0.57	0.55	0.59
Y60	0.85	0.74	0.98	0.62	0.50	0.81	0.57	0.56	0.62
Y60	0.82	0.75	0.85	0.49	0.47	0.76	0.50	0.49	0.52
Y60	0.82	0.74	0.86	0.54	0.51	0.80	0.56	0.54	0.59
Y60	0.79	0.76	0.86	0.53	0.46	0.77	0.53	0.53	0.56
Y60	0.83	0.76	0.89	0.47	0.46	0.75	0.50	0.48	0.52
Y60	0.81	0.76	0.93	0.48	0.47	0.77	0.50	0.46	0.51
Y60	0.85	0.79	0.93	0.56	0.46	0.75	0.52	0.56	0.54
Y60	0.96	0.76	0.94	0.61	0.50	0.82	0.59	0.59	0.63
Y80	0.84	0.75	0.90	0.57	0.52	0.80	0.56	0.57	0.59
Y80	0.88	0.78	0.89	0.55	0.50	0.78	0.57	0.58	0.60
Y80	0.85	0.74	0.89	0.51	0.47	0.77	0.52	0.49	0.54
Y80	0.84	0.74	0.90	0.49	0.45	0.76	0.52	0.46	0.51
Y80	0.85	0.68	0.92	0.59	0.46	0.79	0.53	0.54	0.57
Y80	0.78	0.72	0.80	0.49	0.46	0.77	0.51	0.47	0.52
Y80	0.87	0.76	0.92	0.50	0.48	0.78	0.55	0.51	0.56
Y80	0.83	0.72	0.90	0.55	0.47	0.78	0.53	0.49	0.55
Y80	0.80	0.74	0.85	0.52	0.46	0.77	0.52	0.53	0.55
Y80	0.80	0.73	0.83	0.50	0.46	0.77	0.52	0.52	0.54

Y80	0.83	0.78	0.90	0.49	0.47	0.76	0.52	0.49	0.53
Y80	0.76	0.68	0.76	0.47	0.45	0.77	0.50	0.45	0.50
Y80	0.87	0.77	0.88	0.62	0.74	0.88	0.67	0.64	0.70
Y80	0.83	0.76	0.87	0.47	0.46	0.76	0.50	0.46	0.51
Y80	0.81	0.75	0.82	0.58	0.47	0.79	0.58	0.60	0.62
Y80	0.84	0.76	0.83	0.55	0.50	0.81	0.62	0.60	0.65
Y80	0.89	0.80	0.99	0.51	0.47	0.76	0.51	0.49	0.53
Y80	0.83	0.76	0.85	0.55	0.47	0.78	0.55	0.56	0.58
Y80	0.82	0.76	0.84	0.57	0.47	0.79	0.57	0.59	0.61
Y80	0.78	0.74	0.82	0.50	0.45	0.75	0.49	0.47	0.50
Y80	0.82	0.76	0.91	0.50	0.45	0.76	0.51	0.49	0.51
Y80	0.90	0.79	0.91	0.63	0.64	0.86	0.65	0.63	0.69
Y80	0.83	0.75	0.83	0.56	0.50	0.80	0.58	0.58	0.62
Y80	0.91	0.76	0.94	0.58	0.48	0.79	0.57	0.59	0.60
Y80	0.83	0.76	0.88	0.57	0.50	0.81	0.57	0.57	0.60
Y80	0.84	0.75	0.90	0.52	0.46	0.78	0.53	0.51	0.55
Y80	0.87	0.81	0.90	0.56	0.49	0.79	0.58	0.57	0.60
Y80	0.86	0.78	0.91	0.60	0.49	0.81	0.59	0.63	0.63
Y80	0.84	0.74	0.84	0.57	0.46	0.79	0.56	0.59	0.60
Y80	0.83	0.72	0.80	0.62	0.48	0.83	0.62	0.64	0.67
Y80	0.81	0.74	0.82	0.51	0.51	0.78	0.54	0.51	0.56
Y80	0.79	0.74	0.82	0.56	0.47	0.82	0.55	0.54	0.58
Y80	0.89	0.79	0.92	0.54	0.49	0.76	0.54	0.56	0.56
Y80	0.81	0.79	0.86	0.58	0.48	0.76	0.57	0.57	0.58
Y80	0.87	0.79	0.94	0.55	0.47	0.77	0.57	0.56	0.59
Y80	0.85	0.79	0.90	0.55	0.50	0.79	0.57	0.58	0.61
Y80	0.88	0.75	0.89	0.51	0.49	0.79	0.55	0.52	0.57
Y80	0.83	0.74	0.89	0.52	0.49	0.80	0.55	0.51	0.57
Y80	0.80	0.74	0.83	0.50	0.46	0.75	0.51	0.49	0.52
Y80	0.79	0.74	0.89	0.46	0.43	0.76	0.47	0.44	0.47

Human validation set

	AT1 cell	AT2 cell	Basal	Ciliated	Club	Goblet	Capillary	Fibroblast	Myofibroblast
Y40	0.98	1.10	0.90	0.50	1.27	0.61	0.98	0.80	0.72
Y40	0.98	0.99	0.91	0.61	1.21	0.67	0.99	0.83	0.75
Y40	0.93	0.98	0.89	0.43	1.20	0.60	0.87	0.80	0.77
Y40	0.97	1.05	0.99	0.44	1.23	0.62	0.94	0.82	0.75
Y40	0.99	1.09	0.92	0.51	1.25	0.63	0.93	0.77	0.73
Y40	0.95	1.07	0.96	0.35	1.22	0.60	0.96	0.82	0.79
Y40	0.94	1.01	0.88	0.53	1.26	0.61	0.92	0.70	0.64
Y40	0.98	1.01	0.90	0.44	1.21	0.59	0.93	0.85	0.79
Y40	0.96	1.02	0.96	0.45	1.26	0.57	0.91	0.87	0.77
Y40	0.93	1.02	0.88	0.46	1.25	0.60	0.87	0.74	0.72
Y40	0.95	1.05	0.89	0.39	1.24	0.59	0.93	0.77	0.72
Y40	0.96	1.04	0.90	0.47	1.23	0.61	0.92	0.79	0.72
Y40	0.98	1.15	0.91	0.40	1.29	0.63	0.80	0.75	0.70
Y40	0.98	1.06	0.90	0.38	1.21	0.58	0.96	0.82	0.78
Y40	0.93	1.00	0.87	0.44	1.23	0.60	0.91	0.80	0.79
Y40	0.97	1.03	0.92	0.60	1.22	0.65	0.98	0.78	0.72
Y40	0.98	1.04	0.91	0.44	1.21	0.57	0.93	0.80	0.76

Y40	0.96	0.99	0.90	0.45	1.21	0.58	0.96	0.81	0.76
Y40	0.97	1.01	0.97	0.48	1.23	0.62	0.96	0.82	0.79
Y40	0.98	1.02	0.89	0.45	1.23	0.57	0.98	0.77	0.72
Y40	0.95	0.96	0.88	0.73	1.20	0.66	0.89	0.77	0.75
Y40	0.99	1.02	0.92	0.48	1.23	0.61	0.97	0.84	0.80
Y40	0.98	1.03	0.92	0.47	1.24	0.59	0.94	0.81	0.76
Y40	0.96	1.00	0.90	0.46	1.24	0.59	0.95	0.83	0.76
Y40	0.94	1.01	0.88	0.61	1.25	0.54	0.81	0.75	0.71
Y40	0.95	1.07	0.92	0.56	1.27	0.67	0.90	0.76	0.72
Y40	0.96	1.04	0.91	0.43	1.25	0.63	0.89	0.77	0.75
Y60	0.93	1.00	0.93	0.45	1.21	0.60	0.85	0.82	0.79
Y60	0.94	0.99	0.88	0.62	1.24	0.66	0.95	0.76	0.67
Y60	0.93	0.93	0.88	0.48	1.20	0.56	0.89	0.84	0.84
Y60	0.97	1.01	0.95	0.39	1.23	0.58	0.99	0.90	0.81
Y60	0.97	1.00	0.88	0.57	1.21	0.62	0.97	0.78	0.71
Y60	0.94	1.01	0.86	0.55	1.24	0.64	0.89	0.71	0.69
Y60	0.98	0.98	0.91	0.56	1.22	0.62	0.93	0.83	0.80
Y60	0.98	1.02	0.95	0.54	1.24	0.59	0.88	0.86	0.77
Y60	0.89	0.92	0.92	0.59	1.22	0.61	0.91	0.87	0.75
Y60	0.96	1.04	0.92	0.50	1.26	0.60	0.87	0.84	0.76
Y60	0.96	0.98	0.90	0.67	1.22	0.59	0.99	0.80	0.72
Y60	0.95	1.02	0.92	0.43	1.26	0.63	0.87	0.79	0.80
Y60	0.98	1.05	0.99	0.41	1.24	0.63	0.97	0.84	0.78
Y60	0.97	1.02	0.94	0.55	1.23	0.60	0.90	0.86	0.81
Y60	0.96	1.02	0.87	0.44	1.21	0.61	0.90	0.76	0.75
Y60	0.97	1.00	0.90	0.45	1.22	0.59	0.89	0.83	0.78
Y60	0.99	1.01	0.90	0.52	1.22	0.58	0.96	0.80	0.75
Y60	0.96	1.10	0.94	0.36	1.24	0.64	0.92	0.75	0.73
Y60	0.96	1.03	0.92	0.34	1.23	0.57	0.92	0.85	0.78
Y60	0.94	0.98	0.93	0.58	1.21	0.67	0.91	0.80	0.78
Y60	0.96	0.98	0.86	0.49	1.23	0.58	0.85	0.77	0.73
Y60	0.93	0.98	0.89	0.46	1.23	0.57	0.84	0.82	0.83
Y60	0.96	1.03	1.00	0.69	1.26	0.64	0.84	0.85	0.78
Y60	0.96	0.97	0.86	0.42	1.20	0.55	0.94	0.84	0.79
Y60	0.96	1.01	0.89	0.53	1.22	0.56	0.91	0.81	0.77
Y60	0.97	1.08	0.93	0.39	1.24	0.56	0.94	0.84	0.78
Y60	0.93	0.92	0.89	0.46	1.20	0.67	0.93	0.73	0.69
Y60	1.04	1.05	0.91	0.49	1.26	0.59	0.85	0.74	0.73
Y60	0.94	0.98	0.94	0.45	1.23	0.61	0.89	0.83	0.75
Y60	0.93	1.10	0.92	0.51	1.28	0.64	0.86	0.72	0.72
Y60	0.91	1.00	0.88	0.37	1.23	0.59	0.87	0.80	0.79
Y60	0.98	1.10	0.95	0.53	1.24	0.64	0.90	0.83	0.79
Y60	0.98	0.96	0.89	0.49	1.21	0.59	0.98	0.82	0.75
Y60	0.96	1.07	0.89	0.38	1.22	0.59	0.96	0.80	0.74
Y60	1.00	1.11	0.98	0.36	1.28	0.64	0.93	0.81	0.79
Y60	0.97	1.04	0.93	0.44	1.24	0.59	0.86	0.82	0.76
Y60	0.96	1.01	0.89	0.51	1.24	0.59	0.94	0.82	0.76
Y60	0.92	1.01	0.99	0.36	1.22	0.66	0.87	0.77	0.82
Y60	0.99	1.06	0.95	0.43	1.23	0.61	0.90	0.87	0.83
Y60	0.97	1.03	0.94	0.73	1.25	0.65	0.92	0.79	0.74

Y60	1.01	1.12	0.99	0.39	1.26	0.62	0.91	0.85	0.77
Y60	0.93	0.94	0.91	0.52	1.20	0.57	0.88	0.78	0.74
Y60	0.93	0.92	0.88	0.65	1.23	0.59	0.82	0.77	0.80
Y60	0.94	1.05	0.89	0.39	1.22	0.60	0.91	0.76	0.73
Y60	0.96	0.96	0.88	0.40	1.18	0.55	0.92	0.82	0.77
Y60	0.96	1.02	0.95	0.63	1.20	0.65	0.98	0.83	0.75
Y60	0.92	0.95	0.90	0.51	1.18	0.61	0.89	0.78	0.75
Y60	0.93	0.90	0.86	0.51	1.17	0.58	0.92	0.80	0.75
Y60	0.96	1.01	0.90	0.61	1.24	0.64	0.92	0.81	0.71
Y60	0.98	1.00	0.94	0.53	1.22	0.61	0.87	0.81	0.78
Y60	0.94	1.01	0.84	0.36	1.21	0.57	0.93	0.77	0.72
Y60	0.93	0.96	0.97	0.51	1.23	0.62	0.84	0.87	0.81
Y60	0.94	0.95	0.88	0.38	1.21	0.59	0.87	0.77	0.75
Y60	0.96	1.03	0.89	0.47	1.26	0.60	0.89	0.74	0.75
Y60	0.90	0.98	0.88	0.36	1.22	0.61	0.89	0.74	0.70
Y60	0.98	1.01	0.92	0.42	1.24	0.57	0.95	0.84	0.79
Y60	0.99	1.02	0.93	0.34	1.20	0.57	1.03	0.88	0.79
Y60	0.96	1.04	0.98	0.47	1.26	0.62	0.91	0.87	0.77
Y60	0.97	1.01	0.90	0.54	1.23	0.58	0.94	0.82	0.71
Y60	0.94	0.95	0.90	0.65	1.21	0.64	0.89	0.81	0.83
Y60	0.96	1.03	0.88	0.37	1.21	0.58	0.98	0.77	0.73
Y60	0.97	1.10	0.91	0.37	1.23	0.58	0.92	0.80	0.74
Y60	0.97	1.13	0.95	0.39	1.26	0.60	0.95	0.83	0.80
Y60	0.94	0.94	0.87	0.45	1.22	0.57	0.84	0.74	0.74
Y60	0.96	1.05	0.93	0.41	1.23	0.58	0.92	0.78	0.73
Y60	0.99	0.99	0.89	0.48	1.22	0.58	0.96	0.81	0.74
Y60	0.94	1.03	0.88	0.52	1.25	0.58	0.89	0.80	0.75
Y60	0.95	0.99	0.92	0.44	1.24	0.59	0.91	0.80	0.83
Y60	0.95	1.01	0.92	0.41	1.21	0.60	0.90	0.80	0.80
Y60	0.98	0.95	0.86	0.47	1.17	0.57	1.03	0.81	0.72
Y60	0.93	0.93	0.95	0.37	1.17	0.58	0.98	0.86	0.76
Y60	0.91	0.93	0.90	0.65	1.21	0.65	0.89	0.80	0.78
Y60	0.89	0.92	0.90	0.34	1.17	0.57	0.97	0.84	0.81
Y60	0.99	1.03	0.91	0.39	1.22	0.60	0.98	0.82	0.70
Y60	0.96	1.07	0.91	0.43	1.22	0.59	0.93	0.83	0.77
Y60	0.93	0.95	0.93	0.36	1.22	0.59	0.90	0.79	0.81
Y60	0.95	0.99	0.88	0.56	1.23	0.59	0.91	0.82	0.75
Y60	0.99	1.00	0.90	0.35	1.24	0.58	0.92	0.79	0.73
Y60	0.91	0.90	0.96	0.67	1.23	0.72	0.84	0.72	0.77
Y60	0.95	0.99	0.92	0.38	1.24	0.57	0.91	0.80	0.80
Y60	0.98	1.06	0.97	0.40	1.23	0.61	1.00	0.84	0.85
Y60	0.96	1.00	0.88	0.38	1.18	0.55	1.01	0.85	0.75
Y60	0.94	0.95	0.87	0.72	1.23	0.67	0.95	0.75	0.68
Y60	0.93	1.02	0.94	0.49	1.27	0.64	0.86	0.76	0.81
Y60	0.92	1.06	0.91	0.36	1.24	0.63	0.84	0.74	0.76
Y60	0.97	1.10	0.91	0.39	1.25	0.59	0.93	0.82	0.75
Y60	0.96	1.09	0.96	0.57	1.29	0.63	0.93	0.80	0.70
Y60	0.96	1.03	0.89	0.45	1.21	0.57	1.00	0.80	0.75
Y60	1.02	1.08	0.89	0.54	1.26	0.61	0.91	0.73	0.71
Y60	0.95	1.02	0.99	0.41	1.24	0.64	0.86	0.82	0.86

Y60	1.02	1.13	0.95	0.42	1.28	0.65	0.89	0.81	0.74
Y60	1.02	1.14	0.94	0.52	1.29	0.61	0.90	0.82	0.73
Y60	1.01	1.16	0.91	0.46	1.30	0.62	0.86	0.69	0.68
Y60	0.92	1.00	0.92	0.58	1.20	0.60	0.83	0.85	0.76
Y60	0.91	0.88	0.91	0.45	1.17	0.59	0.91	0.87	0.84
Y60	0.92	0.98	0.91	0.51	1.20	0.63	0.89	0.79	0.80
Y60	0.98	1.08	0.96	0.62	1.26	0.61	0.91	0.84	0.77
Y60	0.98	0.99	0.93	0.57	1.22	0.61	0.98	0.83	0.76
Y60	0.99	1.02	0.92	0.37	1.20	0.60	0.98	0.88	0.81
Y60	0.97	0.98	0.88	0.53	1.22	0.60	0.98	0.80	0.72
Y60	0.98	1.04	0.91	0.44	1.22	0.58	0.94	0.85	0.78
Y60	0.97	1.12	0.91	0.62	1.30	0.65	0.70	0.55	0.54
Y60	0.96	1.03	0.91	0.44	1.22	0.58	0.94	0.81	0.76
Y60	0.98	0.98	0.91	0.38	1.18	0.58	0.94	0.88	0.81
Y60	1.00	1.03	0.88	0.40	1.18	0.57	1.09	0.82	0.75
Y60	0.96	1.02	0.90	0.56	1.21	0.62	0.97	0.81	0.73
Y60	0.95	1.04	0.90	0.43	1.19	0.59	0.97	0.81	0.76
Y60	0.99	1.06	0.92	0.35	1.22	0.58	1.02	0.84	0.79
Y60	0.96	0.94	0.93	0.63	1.23	0.61	0.89	0.80	0.83
Y60	0.99	1.01	0.90	0.42	1.21	0.58	0.94	0.85	0.81
Y60	0.99	1.00	0.92	0.37	1.21	0.59	0.89	0.91	0.79
Y60	0.96	1.03	0.97	0.33	1.22	0.61	0.99	0.86	0.80
Y60	1.01	1.05	0.94	0.37	1.21	0.57	1.01	0.91	0.83
Y60	0.97	0.97	0.87	0.36	1.20	0.59	0.99	0.86	0.78
Y60	0.98	1.01	0.87	0.38	1.23	0.56	0.98	0.82	0.75
Y60	1.00	1.04	0.93	0.42	1.22	0.59	1.02	0.86	0.76
Y60	0.98	1.13	0.89	0.57	1.28	0.67	0.80	0.67	0.66
Y60	0.90	0.98	0.90	0.69	1.21	0.70	0.86	0.73	0.73
Y60	0.96	1.03	0.94	0.49	1.20	0.60	0.96	0.82	0.75
Y60	0.90	0.85	0.91	0.31	1.17	0.57	0.89	0.82	0.83
Y60	0.98	0.99	0.90	0.59	1.20	0.67	0.92	0.78	0.74
Y60	1.00	1.06	0.88	0.65	1.27	0.64	0.85	0.71	0.67
Y60	0.97	1.19	0.91	0.47	1.29	0.64	0.81	0.67	0.67
Y60	0.98	0.99	0.92	0.46	1.21	0.59	0.92	0.86	0.79
Y60	1.02	1.15	0.91	0.40	1.28	0.62	0.87	0.80	0.73
Y60	0.97	0.99	0.89	0.62	1.24	0.64	0.97	0.81	0.73
Y60	0.99	0.96	0.90	0.56	1.19	0.60	1.02	0.88	0.76
Y60	0.97	1.04	0.93	0.46	1.24	0.61	0.86	0.80	0.76
Y60	0.90	0.95	0.95	0.42	1.21	0.62	0.75	0.73	0.78
Y60	0.96	0.94	0.85	0.38	1.21	0.57	0.96	0.78	0.72
Y60	0.98	1.13	0.90	0.36	1.24	0.59	0.93	0.78	0.73
Y60	0.95	0.91	0.89	0.65	1.22	0.65	0.94	0.83	0.78
Y60	0.91	1.02	0.96	0.42	1.22	0.64	0.85	0.77	0.81
Y60	0.95	1.01	0.86	0.55	1.22	0.58	0.91	0.75	0.69
Y60	0.97	0.98	0.89	0.47	1.19	0.57	1.05	0.86	0.76
Y60	0.95	0.97	0.86	0.45	1.19	0.55	0.91	0.80	0.73
Y60	0.96	1.04	0.88	0.40	1.23	0.59	1.01	0.82	0.76
Y60	0.97	1.10	0.94	0.49	1.25	0.65	0.91	0.83	0.76
Y60	0.97	1.00	0.88	0.42	1.23	0.57	0.87	0.80	0.76
Y60	0.95	1.07	0.87	0.44	1.26	0.61	0.80	0.67	0.68

Y60	0.87	0.85	0.87	0.44	1.24	0.60	0.70	0.67	0.70
Y60	0.95	1.00	0.94	0.40	1.25	0.62	0.89	0.82	0.75
Y60	0.92	1.10	0.96	0.47	1.28	0.70	0.81	0.77	0.77
Y60	0.92	0.99	0.87	0.45	1.21	0.59	0.80	0.74	0.68
Y60	0.95	1.01	0.85	0.53	1.21	0.59	0.81	0.75	0.65
Y60	1.00	1.05	0.90	0.62	1.26	0.66	0.81	0.74	0.74
Y60	0.94	1.08	0.91	0.42	1.23	0.61	0.88	0.76	0.70
Y60	0.98	1.01	0.96	0.42	1.22	0.62	0.82	0.81	0.80
Y60	0.94	0.94	0.93	0.57	1.25	0.65	0.82	0.74	0.76
Y60	0.97	1.10	0.90	0.48	1.25	0.63	0.92	0.72	0.70
Y60	0.96	0.89	0.86	0.53	1.21	0.57	0.89	0.81	0.76
Y60	1.00	1.01	0.96	0.36	1.21	0.60	0.97	0.91	0.82
Y60	0.96	0.99	0.90	0.58	1.21	0.61	0.93	0.80	0.76
Y60	0.98	1.02	0.87	0.58	1.24	0.60	0.91	0.74	0.71
Y60	0.98	1.07	0.94	0.60	1.25	0.59	0.91	0.79	0.76
Y60	0.98	1.03	0.91	0.44	1.21	0.58	0.96	0.85	0.79
Y60	0.97	1.02	0.91	0.37	1.21	0.58	0.97	0.82	0.77
Y60	0.97	0.98	0.88	0.52	1.22	0.61	0.93	0.80	0.72
Y60	0.95	1.06	0.91	0.50	1.25	0.64	0.91	0.78	0.73
Y60	0.98	1.03	0.94	0.46	1.23	0.56	0.97	0.86	0.74
Y60	0.96	1.07	0.93	0.50	1.23	0.63	0.97	0.82	0.74
Y60	0.94	0.96	0.91	0.53	1.21	0.60	0.88	0.82	0.79
Y60	0.96	0.99	0.86	0.52	1.20	0.59	0.95	0.83	0.75
Y60	0.91	0.91	0.90	0.67	1.21	0.61	0.93	0.86	0.78
Y60	0.97	1.03	0.91	0.44	1.23	0.58	0.99	0.86	0.76
Y60	0.93	1.05	0.94	0.47	1.22	0.65	0.93	0.79	0.78
Y60	0.99	0.99	0.95	0.47	1.25	0.60	0.94	0.86	0.81
Y60	0.93	0.91	0.86	0.68	1.20	0.57	0.89	0.80	0.77
Y60	0.91	1.02	0.92	0.54	1.22	0.66	0.84	0.82	0.83
Y60	0.96	1.08	0.92	0.41	1.26	0.59	0.89	0.81	0.75
Y60	0.96	1.02	0.93	0.53	1.23	0.60	0.99	0.82	0.79
Y60	0.97	1.01	0.90	0.55	1.21	0.59	0.93	0.83	0.76
Y60	0.92	1.02	0.90	0.44	1.23	0.60	0.84	0.75	0.73
Y60	0.94	1.01	0.96	0.50	1.25	0.62	0.87	0.86	0.76
Y60	0.92	1.05	0.93	0.57	1.25	0.65	0.91	0.80	0.77
Y60	0.97	1.02	0.91	0.36	1.21	0.60	0.93	0.87	0.81
Y60	0.98	1.01	0.93	0.42	1.19	0.60	1.01	0.82	0.78
Y60	0.95	1.00	0.90	0.47	1.21	0.60	0.91	0.76	0.77
Y60	0.95	0.96	0.94	0.44	1.21	0.60	0.87	0.85	0.84
Y60	0.98	1.07	0.93	0.42	1.21	0.62	0.96	0.83	0.80
Y60	0.98	1.05	0.89	0.44	1.28	0.56	0.91	0.76	0.72
Y60	1.00	0.94	0.88	0.46	1.20	0.56	1.01	0.83	0.77
Y60	1.00	1.00	0.92	0.42	1.21	0.56	1.01	0.84	0.76
Y60	0.99	1.04	0.93	0.39	1.23	0.61	0.99	0.86	0.78
Y60	0.98	1.07	0.92	0.45	1.22	0.59	0.94	0.77	0.75
Y60	0.97	1.04	0.91	0.49	1.24	0.58	0.93	0.83	0.80
Y60	0.92	1.00	0.92	0.43	1.17	0.61	0.82	0.79	0.80
Y60	0.98	1.02	0.93	0.45	1.23	0.63	0.96	0.83	0.74
Y60	0.96	1.11	0.96	0.46	1.28	0.64	0.90	0.86	0.80
Y60	0.92	0.98	0.92	0.50	1.20	0.63	0.89	0.83	0.83

Y60	0.96	1.08	0.90	0.46	1.26	0.58	0.83	0.78	0.72
Y80	0.98	1.01	0.97	0.51	1.25	0.63	0.97	0.88	0.80
Y80	0.96	1.03	0.89	0.53	1.25	0.60	0.96	0.80	0.70
Y80	0.99	1.08	0.95	0.39	1.24	0.59	0.92	0.83	0.75
Y80	0.89	0.92	0.97	0.36	1.20	0.63	0.79	0.78	0.84
Y80	0.94	0.96	0.86	0.41	1.22	0.58	0.95	0.78	0.73
Y80	0.97	1.11	0.93	0.37	1.27	0.60	0.82	0.74	0.78
Y80	0.98	0.94	0.95	0.57	1.24	0.58	0.94	0.87	0.80
Y80	0.95	1.01	0.94	0.44	1.21	0.61	0.90	0.83	0.81
Y80	0.97	1.03	0.93	0.56	1.23	0.59	0.97	0.80	0.76
Y80	0.96	0.86	0.87	0.49	1.17	0.56	0.95	0.87	0.75
Y80	0.89	0.96	0.91	0.36	1.19	0.61	0.83	0.74	0.80
Y80	0.94	0.91	0.86	0.71	1.21	0.63	0.95	0.75	0.72
Y80	0.95	1.00	0.94	0.38	1.26	0.58	0.92	0.78	0.77
Y80	1.00	1.11	0.96	0.44	1.23	0.64	0.92	0.83	0.80
Y80	0.98	0.97	0.89	0.60	1.21	0.59	0.91	0.77	0.73
Y80	0.91	1.06	0.97	0.40	1.24	0.62	0.81	0.77	0.77
Y80	0.98	1.14	0.93	0.43	1.25	0.62	0.97	0.84	0.76
Y80	0.93	0.98	0.86	0.57	1.21	0.59	0.87	0.80	0.75
Y80	0.94	1.01	0.89	0.64	1.22	0.61	0.85	0.81	0.75
Y80	0.94	0.97	0.88	0.55	1.22	0.59	0.97	0.79	0.75
Y80	0.97	1.01	0.95	0.54	1.20	0.63	0.94	0.86	0.80
Y80	0.94	0.94	0.87	0.45	1.20	0.58	0.89	0.80	0.74
Y80	0.94	0.95	0.94	0.44	1.21	0.59	0.95	0.84	0.84
Y80	0.96	1.02	0.93	0.53	1.23	0.61	0.94	0.85	0.78
Y80	0.99	1.01	0.92	0.38	1.20	0.59	0.97	0.87	0.78
Y80	0.96	1.01	0.89	0.43	1.22	0.57	0.99	0.85	0.77
Y80	0.96	0.98	0.89	0.46	1.23	0.59	0.93	0.84	0.77
Y80	0.95	0.99	0.89	0.52	1.21	0.60	0.98	0.82	0.80
Y80	0.91	1.01	0.89	0.38	1.21	0.61	0.84	0.76	0.76
Y80	0.97	1.02	0.91	0.53	1.22	0.60	0.93	0.80	0.80
Y80	1.00	0.97	0.90	0.46	1.20	0.57	1.03	0.81	0.73
Y80	0.94	1.03	0.92	0.43	1.22	0.67	0.92	0.80	0.72
Y80	0.95	0.90	0.87	0.58	1.19	0.60	1.01	0.84	0.74
Y80	0.97	0.98	0.94	0.66	1.25	0.61	0.89	0.82	0.79
Y80	0.98	1.08	0.89	0.37	1.25	0.58	0.97	0.79	0.75
Y80	0.93	0.92	0.91	0.39	1.19	0.57	0.84	0.82	0.85
Y80	0.95	0.98	0.91	0.43	1.22	0.58	0.86	0.84	0.76
Y80	0.93	0.92	0.90	0.70	1.21	0.64	0.87	0.79	0.75
Y80	0.93	0.99	0.91	0.49	1.22	0.63	0.90	0.78	0.79
Y80	0.94	0.98	0.92	0.54	1.23	0.61	0.97	0.81	0.82
Y80	0.89	1.07	0.98	0.59	1.25	0.71	0.82	0.77	0.76
Y80	0.93	1.02	0.88	0.32	1.20	0.57	0.91	0.85	0.77
Y80	0.97	1.01	0.91	0.50	1.21	0.60	0.91	0.82	0.79
Y80	0.93	0.91	0.93	0.85	1.19	0.76	0.97	0.82	0.77
Y80	0.96	1.07	0.92	0.49	1.24	0.66	0.93	0.76	0.73
Y80	0.97	0.91	0.89	0.48	1.17	0.56	0.94	0.84	0.83
Y80	1.01	1.08	0.92	0.70	1.27	0.66	0.77	0.74	0.74
Y80	0.95	1.02	0.95	0.35	1.23	0.63	0.88	0.82	0.88
Y80	0.97	1.07	0.93	0.35	1.22	0.58	0.94	0.83	0.77

Y80	1.01	1.11	0.94	0.38	1.25	0.63	0.89	0.81	0.79
Y80	0.92	0.96	0.90	0.42	1.22	0.61	0.75	0.82	0.78
Y80	0.95	1.01	0.96	0.49	1.25	0.59	0.90	0.88	0.80
Y80	0.94	0.99	0.89	0.44	1.21	0.57	0.88	0.82	0.77
Y80	0.93	0.99	0.90	0.55	1.22	0.57	0.84	0.86	0.79
Y80	0.95	1.02	0.89	0.58	1.23	0.61	0.89	0.84	0.80
Y80	0.97	1.00	0.91	0.41	1.20	0.61	0.95	0.86	0.83
Y80	1.03	1.10	0.95	0.53	1.26	0.63	0.82	0.74	0.72
Y80	0.90	0.89	0.94	0.65	1.25	0.68	0.77	0.77	0.83
Y80	0.98	1.12	0.96	0.47	1.25	0.57	0.89	0.85	0.78
Y80	0.95	0.97	0.88	0.50	1.24	0.60	0.90	0.74	0.77
Y80	0.84	0.81	0.95	0.30	1.17	0.61	0.80	0.79	0.84
Y80	0.95	0.98	0.90	0.49	1.22	0.58	0.96	0.80	0.76
Y80	1.00	1.09	0.95	0.45	1.22	0.58	1.03	0.86	0.79
Y80	0.96	1.04	0.94	0.42	1.26	0.63	0.91	0.80	0.77
Y80	0.98	1.00	0.95	0.36	1.24	0.59	0.94	0.88	0.82
Y80	1.00	0.98	0.99	0.36	1.22	0.60	1.04	0.86	0.80
Y80	0.92	0.98	0.90	0.42	1.23	0.57	0.88	0.84	0.79
Y80	0.97	1.02	0.95	0.48	1.25	0.65	0.93	0.86	0.87
Y80	0.94	0.91	1.01	0.71	1.23	0.68	0.87	0.89	0.83
Y80	0.99	1.13	0.94	0.40	1.25	0.60	0.94	0.83	0.77
Y80	0.93	1.01	0.85	0.70	1.22	0.64	0.79	0.70	0.65
Y80	0.95	1.04	0.95	0.55	1.26	0.67	0.83	0.78	0.79
Y80	0.93	0.96	0.97	0.38	1.20	0.64	0.92	0.78	0.82
Y80	0.99	1.07	0.95	0.42	1.22	0.61	0.98	0.88	0.83
Y80	0.96	1.01	0.94	0.40	1.22	0.59	0.88	0.82	0.82
Y80	0.96	0.97	0.93	0.53	1.20	0.61	0.90	0.86	0.83
Y80	0.93	1.00	0.87	0.52	1.22	0.60	0.76	0.67	0.67
Y80	0.99	1.02	0.89	0.38	1.19	0.58	1.03	0.73	0.70
Y80	0.96	1.05	0.93	0.37	1.22	0.62	0.97	0.84	0.80
Y80	0.98	0.96	0.92	0.51	1.21	0.60	1.01	0.84	0.80
Y80	0.90	0.95	0.93	0.37	1.20	0.60	0.83	0.83	0.83
Y80	0.96	1.00	0.94	0.40	1.20	0.60	0.94	0.84	0.77
Y80	0.98	1.03	0.92	0.50	1.22	0.59	0.92	0.85	0.79
Y80	0.97	1.06	0.94	0.43	1.23	0.62	0.87	0.81	0.81
Y80	1.00	1.06	0.93	0.38	1.21	0.57	1.02	0.84	0.79
Y80	1.01	1.04	0.93	0.50	1.23	0.59	0.99	0.82	0.76
Y80	0.97	1.04	0.91	0.47	1.22	0.63	0.95	0.79	0.76
Y80	0.92	0.98	0.95	0.43	1.22	0.64	0.85	0.87	0.85
Y80	0.94	0.98	0.91	0.52	1.24	0.59	0.88	0.84	0.81
	Dendritic	Neutrophil	Macrophage	NK	B cell	CD4+ Naive	CD4+ Effect	CD8+ Naive	CD8+ Effect
Y40	1.02	0.77	1.14	0.75	0.51	0.89	0.62	0.59	0.60
Y40	0.94	0.74	1.03	0.79	0.49	0.90	0.65	0.62	0.63
Y40	0.99	0.76	1.11	0.73	0.49	0.91	0.62	0.60	0.61
Y40	0.99	0.86	1.12	0.78	0.51	0.90	0.68	0.67	0.65
Y40	1.03	0.77	1.16	0.78	0.49	0.91	0.65	0.63	0.65
Y40	1.02	0.78	1.10	0.78	0.52	0.95	0.69	0.64	0.67
Y40	1.04	0.77	1.15	0.72	0.51	0.89	0.60	0.57	0.60
Y40	0.99	0.73	1.07	0.71	0.49	0.89	0.60	0.56	0.58
Y40	1.02	0.78	1.10	0.80	0.53	0.91	0.66	0.61	0.64

Y40	1.02	0.74	1.13	0.77	0.50	0.91	0.65	0.61	0.62
Y40	1.08	0.79	1.18	0.85	0.55	0.92	0.72	0.71	0.73
Y40	1.04	0.77	1.14	0.76	0.52	0.88	0.61	0.59	0.60
Y40	1.06	0.77	1.21	0.74	0.52	0.88	0.62	0.57	0.59
Y40	1.03	0.76	1.11	0.77	0.52	0.91	0.65	0.63	0.66
Y40	1.02	0.73	1.08	0.75	0.57	0.90	0.64	0.58	0.62
Y40	1.03	0.77	1.16	0.74	0.48	0.89	0.62	0.61	0.61
Y40	1.00	0.73	1.08	0.76	0.51	0.92	0.63	0.59	0.63
Y40	1.03	0.74	1.09	0.79	0.50	0.89	0.65	0.62	0.64
Y40	1.01	0.78	1.12	0.76	0.53	0.91	0.64	0.64	0.65
Y40	0.97	0.73	1.06	0.74	0.49	0.91	0.64	0.59	0.63
Y40	0.96	0.76	1.06	0.74	0.50	0.91	0.65	0.62	0.63
Y40	1.02	0.75	1.10	0.77	0.52	0.92	0.66	0.64	0.65
Y40	1.02	0.76	1.11	0.76	0.51	0.90	0.65	0.62	0.64
Y40	1.03	0.76	1.14	0.80	0.52	0.91	0.66	0.64	0.66
Y40	0.96	0.72	1.02	0.71	0.48	0.91	0.63	0.58	0.60
Y40	1.03	0.76	1.12	0.86	0.54	0.93	0.72	0.73	0.70
Y40	1.04	0.76	1.14	0.79	0.53	0.91	0.67	0.64	0.66
Y60	1.05	0.76	1.08	0.81	0.58	0.96	0.71	0.68	0.71
Y60	1.02	0.75	1.14	0.80	0.52	0.90	0.63	0.64	0.63
Y60	0.99	0.69	1.04	0.72	0.51	0.93	0.64	0.58	0.65
Y60	0.99	0.76	1.07	0.81	0.52	0.92	0.67	0.63	0.67
Y60	0.93	0.72	1.04	0.77	0.47	0.89	0.66	0.64	0.64
Y60	1.04	0.76	1.16	0.76	0.51	0.87	0.60	0.58	0.59
Y60	0.99	0.73	1.09	0.74	0.50	0.90	0.61	0.58	0.60
Y60	1.02	0.78	1.11	0.73	0.52	0.90	0.62	0.58	0.61
Y60	0.97	0.74	1.01	0.77	0.60	0.94	0.68	0.61	0.67
Y60	0.98	0.74	1.08	0.72	0.58	0.92	0.65	0.56	0.63
Y60	0.95	0.79	1.08	0.74	0.51	0.90	0.60	0.61	0.62
Y60	1.03	0.76	1.12	0.78	0.51	0.91	0.65	0.62	0.63
Y60	0.93	0.77	1.07	0.69	0.45	0.88	0.61	0.54	0.54
Y60	1.01	0.73	1.07	0.72	0.48	0.90	0.61	0.55	0.59
Y60	1.05	0.74	1.14	0.79	0.52	0.94	0.69	0.69	0.69
Y60	1.01	0.72	1.04	0.74	0.51	0.91	0.63	0.58	0.64
Y60	1.03	0.74	1.08	0.82	0.51	0.93	0.70	0.70	0.72
Y60	1.08	0.83	1.20	0.82	0.53	0.91	0.70	0.69	0.70
Y60	0.97	0.74	1.05	0.78	0.52	0.91	0.64	0.59	0.62
Y60	1.02	0.73	1.09	0.74	0.50	0.90	0.61	0.58	0.60
Y60	1.00	0.73	1.08	0.81	0.51	0.92	0.63	0.66	0.66
Y60	0.99	0.74	1.07	0.78	0.49	0.93	0.66	0.62	0.66
Y60	0.99	0.76	1.11	0.74	0.49	0.91	0.60	0.56	0.57
Y60	0.96	0.71	1.03	0.72	0.49	0.91	0.61	0.56	0.60
Y60	0.99	0.72	1.06	0.74	0.49	0.91	0.62	0.60	0.63
Y60	0.96	0.74	1.02	0.77	0.47	0.93	0.63	0.60	0.62
Y60	1.01	0.72	1.07	0.77	0.49	0.94	0.65	0.64	0.64
Y60	0.98	0.72	1.09	0.73	0.49	0.88	0.59	0.56	0.58
Y60	1.11	0.82	1.14	0.88	0.60	0.99	0.80	0.77	0.81
Y60	1.06	0.75	1.17	0.80	0.56	0.93	0.67	0.66	0.67
Y60	1.05	0.73	1.10	0.75	0.52	0.92	0.63	0.58	0.61
Y60	1.01	0.74	1.12	0.72	0.48	0.90	0.60	0.57	0.57

Y60	0.97	0.74	1.08	0.78	0.48	0.91	0.62	0.66	0.64
Y60	1.03	0.76	1.13	0.77	0.50	0.91	0.64	0.65	0.65
Y60	1.01	0.79	1.15	0.75	0.49	0.87	0.63	0.57	0.56
Y60	1.04	0.77	1.10	0.80	0.49	0.94	0.70	0.66	0.69
Y60	1.00	0.75	1.11	0.79	0.53	0.90	0.64	0.63	0.64
Y60	1.10	0.81	1.18	0.79	0.50	0.90	0.65	0.63	0.62
Y60	1.03	0.79	1.12	0.78	0.51	0.92	0.69	0.64	0.67
Y60	0.97	0.74	1.06	0.76	0.51	0.92	0.64	0.59	0.62
Y60	1.03	0.80	1.13	0.77	0.52	0.92	0.67	0.61	0.62
Y60	1.02	0.76	1.02	0.89	0.51	1.00	0.82	0.79	0.83
Y60	0.99	0.68	1.02	0.75	0.51	0.93	0.65	0.62	0.66
Y60	1.05	0.77	1.15	0.79	0.56	0.91	0.66	0.66	0.66
Y60	1.00	0.74	1.07	0.75	0.48	0.91	0.62	0.61	0.61
Y60	0.97	0.77	1.07	0.78	0.45	0.91	0.63	0.63	0.63
Y60	1.03	0.76	1.11	0.78	0.49	0.92	0.62	0.64	0.63
Y60	0.97	0.71	1.03	0.76	0.46	0.94	0.68	0.64	0.67
Y60	0.98	0.73	1.11	0.71	0.50	0.88	0.58	0.54	0.57
Y60	0.99	0.75	1.06	0.73	0.50	0.90	0.64	0.58	0.61
Y60	0.98	0.73	1.06	0.73	0.56	0.94	0.68	0.63	0.70
Y60	0.90	0.69	0.97	0.73	0.44	0.90	0.60	0.56	0.58
Y60	1.05	0.76	1.14	0.74	0.52	0.91	0.65	0.63	0.66
Y60	1.00	0.73	1.09	0.75	0.53	0.89	0.62	0.57	0.60
Y60	1.01	0.76	1.06	0.81	0.61	0.97	0.79	0.68	0.75
Y60	1.02	0.76	1.09	0.81	0.54	0.93	0.71	0.65	0.69
Y60	1.05	0.80	1.10	0.85	0.54	0.96	0.76	0.73	0.77
Y60	0.94	0.76	1.08	0.75	0.48	0.91	0.63	0.58	0.58
Y60	1.00	0.75	1.09	0.79	0.52	0.94	0.68	0.67	0.71
Y60	1.00	0.73	1.06	0.77	0.48	0.90	0.67	0.61	0.65
Y60	1.02	0.75	1.11	0.82	0.53	0.94	0.67	0.72	0.72
Y60	1.01	0.77	1.14	0.73	0.51	0.90	0.63	0.59	0.63
Y60	1.02	0.81	1.15	0.79	0.55	0.92	0.66	0.64	0.66
Y60	1.00	0.76	1.09	0.78	0.49	0.94	0.66	0.66	0.67
Y60	1.02	0.78	1.12	0.80	0.58	0.94	0.67	0.64	0.68
Y60	0.99	0.72	1.03	0.78	0.52	0.93	0.67	0.66	0.68
Y60	0.99	0.74	1.09	0.75	0.50	0.89	0.61	0.58	0.61
Y60	1.03	0.76	1.11	0.76	0.52	0.92	0.66	0.61	0.64
Y60	1.06	0.76	1.14	0.78	0.52	0.89	0.63	0.62	0.62
Y60	0.97	0.71	1.04	0.81	0.51	0.92	0.66	0.70	0.68
Y60	0.97	0.78	1.08	0.77	0.50	0.89	0.64	0.63	0.62
Y60	1.02	0.72	1.09	0.78	0.54	0.94	0.66	0.66	0.68
Y60	0.98	0.76	1.04	0.80	0.56	0.94	0.70	0.69	0.71
Y60	1.03	0.81	1.17	0.77	0.49	0.90	0.64	0.64	0.63
Y60	1.04	0.76	1.11	0.79	0.51	0.92	0.65	0.65	0.66
Y60	1.05	0.75	1.10	0.79	0.54	0.94	0.70	0.65	0.70
Y60	0.96	0.71	1.05	0.76	0.50	0.90	0.63	0.59	0.62
Y60	1.06	0.75	1.13	0.79	0.50	0.89	0.68	0.64	0.66
Y60	1.00	0.72	1.08	0.71	0.50	0.91	0.62	0.56	0.59
Y60	1.00	0.73	1.08	0.78	0.49	0.92	0.64	0.61	0.63
Y60	1.06	0.79	1.16	0.76	0.50	0.90	0.64	0.60	0.62
Y60	0.95	0.72	1.00	0.79	0.50	0.91	0.63	0.65	0.66

Y60	0.94	0.73	1.04	0.76	0.48	0.91	0.63	0.63	0.63
Y60	1.10	0.75	1.12	0.84	0.60	0.98	0.77	0.71	0.76
Y60	1.09	0.79	1.20	0.73	0.55	0.90	0.63	0.58	0.61
Y60	1.03	0.78	1.14	0.77	0.51	0.88	0.61	0.59	0.60
Y60	1.06	0.81	1.17	0.84	0.58	0.95	0.72	0.68	0.70
Y60	1.01	0.74	1.05	0.83	0.54	0.93	0.73	0.72	0.75
Y60	0.99	0.76	1.13	0.73	0.49	0.86	0.57	0.56	0.56
Y60	1.06	0.77	1.13	0.80	0.53	0.92	0.67	0.62	0.63
Y60	1.09	0.78	1.21	0.78	0.53	0.89	0.64	0.60	0.62
Y60	1.00	0.77	1.13	0.74	0.53	0.88	0.60	0.54	0.57
Y60	1.04	0.77	1.19	0.75	0.53	0.88	0.60	0.60	0.61
Y60	1.03	0.73	1.10	0.81	0.50	0.91	0.61	0.64	0.62
Y60	1.02	0.73	1.06	0.81	0.59	0.94	0.69	0.66	0.70
Y60	1.01	0.70	1.06	0.75	0.50	0.92	0.62	0.60	0.62
Y60	1.02	0.79	1.12	0.80	0.53	0.92	0.66	0.65	0.67
Y60	1.02	0.75	1.10	0.77	0.55	0.89	0.64	0.62	0.63
Y60	1.01	0.74	1.12	0.74	0.50	0.87	0.58	0.56	0.57
Y60	1.01	0.74	1.11	0.83	0.53	0.92	0.67	0.69	0.69
Y60	1.02	0.76	1.09	0.77	0.52	0.92	0.67	0.64	0.66
Y60	1.04	0.80	1.23	0.73	0.49	0.87	0.60	0.58	0.59
Y60	1.05	0.79	1.14	0.76	0.53	0.89	0.62	0.57	0.60
Y60	1.02	0.73	1.12	0.76	0.50	0.88	0.59	0.59	0.58
Y60	0.96	0.74	1.07	0.79	0.50	0.89	0.60	0.65	0.64
Y60	0.97	0.73	1.08	0.75	0.49	0.90	0.62	0.60	0.62
Y60	1.05	0.76	1.14	0.78	0.50	0.91	0.60	0.64	0.62
Y60	1.01	0.76	1.10	0.77	0.51	0.93	0.66	0.63	0.66
Y60	1.01	0.73	1.08	0.78	0.54	0.94	0.67	0.64	0.68
Y60	0.99	0.72	1.04	0.75	0.52	0.91	0.63	0.60	0.64
Y60	1.01	0.75	1.09	0.75	0.52	0.91	0.65	0.60	0.65
Y60	1.09	0.81	1.16	0.85	0.64	0.97	0.75	0.75	0.76
Y60	1.01	0.75	1.08	0.74	0.51	0.88	0.60	0.57	0.59
Y60	0.97	0.75	1.08	0.75	0.49	0.89	0.64	0.63	0.63
Y60	1.03	0.74	1.12	0.83	0.55	0.93	0.70	0.71	0.71
Y60	1.03	0.80	1.13	0.80	0.52	0.92	0.69	0.70	0.70
Y60	1.05	0.75	1.19	0.71	0.50	0.88	0.57	0.55	0.58
Y60	1.02	0.75	1.11	0.78	0.59	0.93	0.66	0.67	0.69
Y60	1.09	0.79	1.12	0.88	0.59	0.99	0.82	0.78	0.82
Y60	1.05	0.75	1.05	0.84	0.76	0.99	0.79	0.74	0.81
Y60	1.04	0.74	1.14	0.79	0.50	0.90	0.61	0.65	0.63
Y60	1.01	0.75	1.14	0.79	0.52	0.88	0.61	0.64	0.64
Y60	1.10	0.78	1.22	0.71	0.53	0.87	0.58	0.54	0.56
Y60	1.02	0.77	1.13	0.72	0.51	0.89	0.60	0.58	0.61
Y60	1.04	0.75	1.15	0.76	0.54	0.88	0.62	0.58	0.60
Y60	0.97	0.74	1.10	0.79	0.51	0.90	0.64	0.66	0.65
Y60	0.97	0.73	1.05	0.77	0.48	0.90	0.62	0.63	0.64
Y60	1.02	0.78	1.14	0.72	0.50	0.89	0.61	0.57	0.59
Y60	1.09	0.82	1.16	0.83	0.69	0.97	0.77	0.71	0.75
Y60	1.03	0.74	1.11	0.77	0.54	0.90	0.66	0.66	0.67
Y60	0.99	0.77	1.13	0.70	0.49	0.86	0.59	0.52	0.55
Y60	0.95	0.69	1.03	0.77	0.49	0.92	0.63	0.61	0.64

Y60	1.04	0.74	1.09	0.71	0.50	0.90	0.60	0.55	0.57
Y60	0.96	0.72	1.07	0.72	0.48	0.88	0.57	0.58	0.58
Y60	0.99	0.74	1.05	0.81	0.51	0.91	0.64	0.67	0.67
Y60	0.92	0.72	0.97	0.68	0.47	0.87	0.57	0.51	0.55
Y60	0.99	0.73	1.07	0.76	0.48	0.90	0.63	0.58	0.62
Y60	1.03	0.77	1.14	0.76	0.53	0.94	0.65	0.63	0.64
Y60	0.95	0.73	1.06	0.71	0.52	0.91	0.63	0.57	0.61
Y60	0.95	0.73	1.06	0.65	0.53	0.88	0.58	0.49	0.53
Y60	1.15	0.80	1.12	0.91	0.67	1.01	0.86	0.82	0.88
Y60	1.08	0.80	1.18	0.81	0.54	0.91	0.69	0.65	0.68
Y60	1.12	0.80	1.14	0.79	0.59	0.95	0.70	0.66	0.70
Y60	1.04	0.77	1.14	0.73	0.52	0.91	0.61	0.61	0.60
Y60	1.02	0.77	1.14	0.82	0.52	0.91	0.67	0.71	0.68
Y60	1.04	0.73	1.15	0.72	0.51	0.89	0.58	0.56	0.58
Y60	1.03	0.77	1.13	0.76	0.57	0.92	0.66	0.63	0.66
Y60	1.04	0.77	1.14	0.77	0.57	0.93	0.67	0.63	0.64
Y60	1.11	0.76	1.13	0.90	0.57	0.99	0.78	0.76	0.77
Y60	1.05	0.76	1.16	0.77	0.56	0.92	0.65	0.63	0.65
Y60	0.91	0.70	0.99	0.70	0.47	0.88	0.59	0.52	0.56
Y60	1.02	0.77	1.13	0.78	0.52	0.91	0.65	0.63	0.66
Y60	1.00	0.76	1.09	0.73	0.50	0.90	0.60	0.59	0.60
Y60	0.97	0.74	1.08	0.78	0.53	0.89	0.65	0.63	0.63
Y60	0.95	0.73	1.06	0.75	0.48	0.89	0.62	0.60	0.61
Y60	1.01	0.76	1.07	0.80	0.52	0.93	0.69	0.68	0.70
Y60	1.00	0.76	1.13	0.75	0.54	0.90	0.64	0.62	0.65
Y60	1.01	0.73	1.09	0.79	0.50	0.91	0.64	0.66	0.66
Y60	1.08	0.78	1.18	0.77	0.53	0.90	0.66	0.63	0.65
Y60	0.97	0.77	1.11	0.81	0.51	0.94	0.69	0.68	0.68
Y60	0.96	0.75	1.06	0.76	0.48	0.91	0.65	0.62	0.64
Y60	1.01	0.72	1.06	0.75	0.51	0.93	0.65	0.62	0.64
Y60	0.97	0.71	1.06	0.74	0.49	0.92	0.62	0.59	0.63
Y60	0.93	0.69	1.01	0.73	0.47	0.91	0.61	0.54	0.61
Y60	1.02	0.76	1.10	0.82	0.55	0.92	0.68	0.67	0.70
Y60	1.06	0.79	1.15	0.80	0.56	0.95	0.72	0.67	0.71
Y60	0.99	0.73	1.06	0.75	0.50	0.90	0.63	0.57	0.61
Y60	0.95	0.72	1.03	0.75	0.48	0.90	0.60	0.60	0.60
Y60	1.04	0.72	1.10	0.76	0.48	0.93	0.64	0.59	0.64
Y60	1.00	0.76	1.12	0.76	0.51	0.91	0.63	0.60	0.63
Y60	1.02	0.79	1.10	0.81	0.52	0.94	0.66	0.66	0.67
Y60	1.00	0.75	1.09	0.78	0.52	0.91	0.63	0.64	0.65
Y60	1.04	0.78	1.17	0.72	0.55	0.91	0.65	0.59	0.62
Y60	1.01	0.73	1.08	0.81	0.50	0.92	0.66	0.67	0.66
Y60	1.06	0.80	1.13	0.80	0.56	0.94	0.70	0.65	0.69
Y60	1.02	0.74	1.11	0.77	0.50	0.90	0.66	0.60	0.63
Y60	1.06	0.77	1.12	0.83	0.50	0.95	0.72	0.69	0.71
Y60	0.97	0.72	1.07	0.72	0.47	0.89	0.61	0.58	0.58
Y60	1.08	0.76	1.11	0.83	0.53	0.96	0.75	0.71	0.75
Y60	1.01	0.74	1.09	0.81	0.50	0.93	0.66	0.67	0.65
Y60	1.02	0.74	1.07	0.80	0.53	0.94	0.71	0.67	0.70
Y60	0.98	0.71	1.05	0.75	0.48	0.90	0.62	0.60	0.63

Y60	1.00	0.76	1.08	0.75	0.52	0.92	0.65	0.59	0.62
Y60	1.00	0.77	1.10	0.76	0.52	0.91	0.64	0.59	0.62
Y60	1.02	0.75	1.09	0.75	0.51	0.90	0.64	0.61	0.63
Y60	0.98	0.77	1.08	0.78	0.52	0.92	0.67	0.67	0.67
Y60	1.05	0.73	1.13	0.75	0.51	0.88	0.61	0.60	0.62
Y60	1.07	0.80	1.15	0.83	0.54	0.95	0.71	0.71	0.74
Y60	1.02	0.76	1.14	0.80	0.53	0.93	0.64	0.59	0.63
Y60	1.04	0.73	1.13	0.77	0.50	0.90	0.64	0.61	0.63
Y60	1.01	0.77	1.12	0.78	0.52	0.92	0.68	0.64	0.65
Y80	1.04	0.79	1.12	0.82	0.54	0.93	0.70	0.67	0.71
Y80	0.97	0.75	1.08	0.76	0.52	0.90	0.65	0.62	0.62
Y80	1.02	0.78	1.13	0.79	0.53	0.94	0.70	0.66	0.67
Y80	1.06	0.79	1.10	0.85	0.62	0.99	0.80	0.74	0.77
Y80	0.96	0.71	1.04	0.77	0.48	0.93	0.65	0.66	0.66
Y80	0.98	0.73	1.07	0.75	0.58	0.92	0.68	0.57	0.62
Y80	0.98	0.75	1.05	0.76	0.51	0.90	0.63	0.58	0.61
Y80	1.04	0.79	1.12	0.79	0.52	0.93	0.67	0.64	0.67
Y80	1.03	0.76	1.11	0.81	0.54	0.94	0.70	0.66	0.68
Y80	0.92	0.71	0.99	0.74	0.47	0.92	0.63	0.62	0.63
Y80	1.08	0.79	1.12	0.82	0.54	0.97	0.78	0.72	0.76
Y80	0.90	0.69	1.00	0.70	0.41	0.87	0.59	0.53	0.56
Y80	1.10	0.80	1.11	0.92	0.61	0.98	0.83	0.81	0.83
Y80	0.99	0.75	1.12	0.74	0.47	0.90	0.61	0.60	0.59
Y80	0.93	0.73	1.03	0.72	0.44	0.88	0.60	0.58	0.57
Y80	1.07	0.78	1.15	0.85	0.57	0.96	0.71	0.70	0.70
Y80	1.03	0.77	1.15	0.77	0.48	0.88	0.62	0.59	0.60
Y80	1.01	0.71	1.07	0.77	0.51	0.95	0.65	0.61	0.67
Y80	0.99	0.71	1.08	0.75	0.48	0.93	0.64	0.59	0.62
Y80	1.00	0.73	1.07	0.80	0.50	0.92	0.70	0.66	0.69
Y80	1.04	0.77	1.13	0.77	0.51	0.92	0.68	0.63	0.65
Y80	0.98	0.74	1.08	0.78	0.54	0.93	0.67	0.63	0.65
Y80	1.03	0.73	1.08	0.80	0.50	0.93	0.71	0.67	0.70
Y80	1.01	0.75	1.11	0.78	0.57	0.94	0.68	0.62	0.66
Y80	1.03	0.78	1.11	0.81	0.58	0.95	0.72	0.69	0.71
Y80	1.02	0.75	1.08	0.79	0.55	0.93	0.68	0.66	0.68
Y80	1.00	0.72	1.04	0.80	0.51	0.93	0.70	0.62	0.67
Y80	1.01	0.72	1.09	0.80	0.51	0.93	0.67	0.67	0.70
Y80	1.07	0.77	1.16	0.73	0.58	0.91	0.66	0.60	0.65
Y80	0.98	0.74	1.07	0.73	0.48	0.90	0.63	0.59	0.61
Y80	0.99	0.74	1.10	0.72	0.50	0.88	0.60	0.61	0.62
Y80	1.03	0.77	1.12	0.77	0.50	0.92	0.65	0.62	0.64
Y80	0.96	0.71	1.02	0.80	0.48	0.91	0.65	0.67	0.66
Y80	1.00	0.76	1.09	0.76	0.50	0.89	0.61	0.58	0.60
Y80	0.98	0.73	1.10	0.73	0.48	0.88	0.63	0.54	0.59
Y80	1.00	0.73	1.04	0.74	0.50	0.90	0.62	0.56	0.60
Y80	1.01	0.73	1.08	0.83	0.51	0.97	0.74	0.71	0.74
Y80	1.01	0.72	1.07	0.79	0.49	0.94	0.66	0.65	0.66
Y80	1.06	0.75	1.13	0.82	0.61	0.96	0.74	0.69	0.73
Y80	1.05	0.74	1.10	0.81	0.54	0.96	0.72	0.67	0.73
Y80	1.06	0.77	1.15	0.81	0.69	0.98	0.76	0.68	0.71

Y80	0.98	0.72	1.06	0.67	0.49	0.87	0.58	0.49	0.55
Y80	1.04	0.73	1.11	0.82	0.51	0.94	0.68	0.71	0.70
Y80	1.02	0.71	1.07	0.83	0.51	0.95	0.70	0.72	0.72
Y80	1.07	0.77	1.19	0.82	0.53	0.91	0.66	0.69	0.66
Y80	0.95	0.70	1.02	0.72	0.47	0.88	0.59	0.57	0.57
Y80	1.03	0.77	1.17	0.72	0.51	0.88	0.60	0.55	0.57
Y80	1.03	0.75	1.10	0.78	0.51	0.91	0.67	0.63	0.66
Y80	1.06	0.77	1.13	0.81	0.55	0.92	0.67	0.67	0.70
Y80	1.03	0.80	1.19	0.74	0.52	0.88	0.60	0.58	0.60
Y80	1.07	0.73	1.15	0.78	0.51	0.92	0.64	0.63	0.66
Y80	1.02	0.77	1.10	0.76	0.55	0.93	0.66	0.59	0.65
Y80	1.02	0.76	1.05	0.79	0.62	0.96	0.74	0.65	0.74
Y80	0.98	0.75	1.05	0.72	0.50	0.89	0.60	0.53	0.58
Y80	1.01	0.72	1.09	0.81	0.52	0.93	0.68	0.65	0.67
Y80	1.04	0.75	1.14	0.76	0.50	0.91	0.63	0.64	0.64
Y80	1.03	0.81	1.19	0.72	0.51	0.88	0.61	0.55	0.57
Y80	1.04	0.71	1.06	0.73	0.54	0.93	0.64	0.58	0.62
Y80	1.00	0.82	1.09	0.71	0.50	0.89	0.60	0.55	0.59
Y80	1.01	0.72	1.09	0.77	0.53	0.91	0.61	0.61	0.64
Y80	1.05	0.79	1.07	0.79	0.67	0.95	0.73	0.66	0.73
Y80	0.98	0.74	1.09	0.80	0.53	0.92	0.66	0.67	0.68
Y80	0.94	0.73	1.03	0.76	0.48	0.90	0.62	0.59	0.62
Y80	1.07	0.76	1.15	0.78	0.58	0.94	0.69	0.64	0.68
Y80	1.09	0.78	1.14	0.77	0.53	0.94	0.72	0.64	0.72
Y80	1.05	0.85	1.16	0.86	0.54	0.91	0.71	0.75	0.73
Y80	1.04	0.74	1.09	0.78	0.61	0.97	0.73	0.66	0.74
Y80	1.06	0.74	1.13	0.80	0.51	0.93	0.64	0.62	0.63
Y80	1.00	0.79	1.10	0.78	0.51	0.93	0.66	0.62	0.64
Y80	1.06	0.78	1.13	0.78	0.53	0.92	0.65	0.64	0.65
Y80	0.95	0.72	1.09	0.69	0.45	0.87	0.53	0.53	0.52
Y80	1.03	0.77	1.08	0.80	0.58	0.95	0.72	0.67	0.68
Y80	1.07	0.82	1.16	0.81	0.60	0.96	0.74	0.68	0.71
Y80	1.03	0.78	1.13	0.78	0.53	0.92	0.65	0.62	0.65
Y80	1.03	0.79	1.12	0.81	0.58	0.96	0.71	0.69	0.69
Y80	0.99	0.75	1.07	0.78	0.54	0.92	0.65	0.64	0.66
Y80	1.03	0.75	1.13	0.74	0.51	0.90	0.66	0.61	0.63
Y80	0.96	0.74	1.07	0.79	0.48	0.89	0.63	0.66	0.66
Y80	1.03	0.75	1.13	0.75	0.51	0.90	0.61	0.59	0.60
Y80	1.05	0.77	1.11	0.82	0.57	0.95	0.68	0.69	0.70
Y80	1.10	0.78	1.16	0.78	0.56	0.93	0.68	0.63	0.67
Y80	1.06	0.79	1.13	0.86	0.59	0.97	0.72	0.73	0.73
Y80	1.00	0.75	1.07	0.74	0.51	0.91	0.63	0.61	0.63
Y80	1.00	0.76	1.14	0.71	0.49	0.88	0.62	0.58	0.59
Y80	1.00	0.76	1.08	0.81	0.51	0.93	0.71	0.67	0.70
Y80	1.03	0.76	1.13	0.79	0.53	0.90	0.67	0.64	0.66
Y80	1.03	0.77	1.13	0.77	0.52	0.91	0.66	0.66	0.65
Y80	1.08	0.76	1.14	0.83	0.62	0.97	0.73	0.68	0.73
Y80	1.06	0.75	1.09	0.81	0.57	0.96	0.72	0.66	0.72

Supplementary Table S14

Commonly regulated DEGs between human and mouse pulmonary genomes

Increased genes:

Dclk1	Cldn2	Rgs5
Fcrla	Cbr3	Cyfip2
Sema4d	Aspa	Cxcl9
Rnase6	Tnfrsf13b	Ccl8
Mst1	Stk33	Col15a1
Poln	Ch25h	
Ssr4	Lck	
Socs3	Irf4	
Col10a1	Lrmp	
Mlana	Cox7a1	
Thbs2	Fmo3	
Cfap70	Cyr61	
Mmp16	Il13ra1	
Fam46c	Casq2	
Acan	Hcar2	
Ctxn1	Ltb	
Ptprt	Thy1	
Prg4	Prg2	
Esr1	Ctsk	
Efhd1	Prkcb	
Clic6	Foxc1	
Cd72	Nr4a1	
Pla1a	Mmp9	
Cd28	Cxcr6	
Cst7	F13a1	
Gpr183	Gzmk	
Rsph10b	Cxcl13	

Decreased genes:

Efnb2
Ednrb
Hecw2
Phex
Wwc2
Col4a4
Rbm25
Gmfb
Ccdc141
Bex1
Wnk1
Cadm1
Hhip
Veph1
Pdzd2
Secisbp2l
Lrp6
Tmem33
Prpf38b
Fibin
Gapvd1
Ttc14
Ostc
Shprh
Edil3
Bmp5
Itm2b
